# Revision of the Ceratocapsine *Renodaeus* group: *Marinonicoris*, *Pilophoropsis*, *Renodaeus*, and *Zanchisme*, with descriptions of four new genera (Heteroptera, Miridae, Orthotylinae)

**DOI:** 10.3897/zookeys.490.8880

**Published:** 2015-03-25

**Authors:** Thomas J. Henry

**Affiliations:** 1 Systematic Entomology Laboratory, Plant Sciences Institute, Agricultural Research Service, United States Department of Agriculture, c/o National Museum of Natural History, Washington, DC 20013–7012

**Keywords:** Insecta, Hemiptera, Heteroptera, Orthotylinae, Ceratocapsini, *Renodaeus* complex, new genera, new species, keys

## Abstract

The *Renodaeus* group, a monophyletic assemblage of genera within the New World orthotyline tribe Ceratocapsini, comprising eight genera, including four new ones, is defined; and 48 species are treated, including 26 described as new and 12 transferred from *Ceratocapsus* Reuter as new combinations. *Ceratocapsidea* **gen. n.** is described to accommodate the new species *Ceratocapsidea bahamaensis* **sp. n.**, from the Bahamas; *Ceratocapsidea baranowskii* **sp. n.**, from Jamaica; *Ceratocapsidea dominicanensis* **sp. n.**, from the Dominican Republic; *Ceratocapsidea rileyi* **sp. n.**, from Texas; *Ceratocapsidea taeniola* **sp. n.**, from Jamaica; *Ceratocapsidea texensis* **sp. n.**, from Texas; *Ceratocapsidea transversa* **sp. n.**, from Mexico (Neuvo León); and *Ceratocapsidea variabilis* **sp. n.**, from Jamaica; and *Ceratocapsus balli* Knight, **comb. n.**, *Ceratocapsus complicatus* Knight, **comb. n.**, *Ceratocapsidea consimilis* Reuter, **comb. n.**, *Ceratocapsus fusiformis* Van Duzee, **comb. n.** (as the type species of the genus), *Ceratocapsus nigropiceus* Reuter, **comb. n.**, and *Ceratocapsus rufistigmus* Blatchley, **comb. n.** [and a neotype designated], *Ceratocapsus clavicornis* Knight, **syn. n.** and *Ceratocapsus divaricatus* Knight, **syn. n.** are treated as junior synonyms of *Ceratocapsus fusiformis* Van Duzee. The genus *Marininocoris* Carvalho and the only included species *Marinonicoris myrmecoides* Carvalho are redescribed. The genus *Pilophoropsis* Poppius is redescribed and revised, *Renodaeus texanus* Knight, **comb. n.** is transferred into it and the three new species *Pilophoropsis bejeanae* **sp. n.**, from Sonora, Mexico; *Pilophoropsis cunealis* **sp. n.**, from Oaxaca, Mexico; *Pilophoropsis quercicola* **sp. n.**, from Arizona, USA, are described. *Pilophoropsidea* **gen. n.** is described to accommodate the 12 new species *Pilophoropsidea brailovskyi* **sp. n.**, from Federal District, Mexico; *Pilophoropsidea cuneata* **sp. n.**, from Chiapas, Mexico; *Pilophoropsidea dimidiata* **sp. n.**, from Durango, Mexico; *Pilophoropsidea fuscata* **sp. n.**, from Durango, Mexico and Arizona and New Mexico, USA; *Pilophoropsidea keltoni* **sp. n.**, from Durango, Mexico; *Pilophoropsidea maxima* **sp. n.**, from Durango, Mexico; *Pilophoropsidea pueblaensis* **sp. n.**, from Puebla, Mexico; *Pilophoropsidea schaffneri* **sp. n.**, from Neuvo León and San Luis Potosi, Mexico; *Pilophoropsidea serrata* **sp. n.**, from Michoacan, Mexico; *Pilophoropsidea touchetae* **sp. n.**, from Mexico (Puebla); *Pilophoropsidea truncata* **sp. n.**, from Mexico (Guerrero); *Pilophoropsidea tuberculata* **sp. n.**, from Mexico (Guerrero); and *Ceratocapsus barberi* Knight, **comb. n.**, *Ceratocapsus camelus* Knight, **comb. n.** (as the type species of the genus), and *Ceratocapsus fascipennis* Knight, **comb. n.** *Pilophoropsita* **gen. n.** is described to accommodate *Pilophoropsidea schaffneri* **sp. n.** from Costa Rica and Mexico (Jalisco, Nayarit, Oaxaca). The genus *Renodaeus* Distant is redescribed and the new species *Renodaeus mimeticus* **sp. n.** from Ecuador is described. The genus *Zanchisme* Kirkaldy is reviewed and the four known species are redescribed. *Zanchismeopsidea* **gen. n.** is described to accommodate *Zanchismeopsidea diegoi* **sp. n.** from Argentina (Santiago del Estero). Provided are habitus illustrations for certain adults (*Pilophoropsidea camelus*, *Pilophoropsis brachyptera* Poppius, *Renodaeus mimeticus*, and *Zanchisme mexicanus* Carvalho & Schaffner), male and female (when available) color digital images and figures of male genitalia of all species, electron photomicrographs of diagnostic characters for selected species, and keys to the genera and their included species. The taxa treated in this paper are arranged alphabetically by genus and species.

## Introduction

The orthotyline tribe Ceratocapsini is a large, diverse New World group of Miridae, currently comprising more than 10 genera (Henry 1994) and nearly 200 species. The tribe has been widely accepted by nearly all early authors subsequent to its establishment (Van Duzee 1916a), including Van Duzee (1916b, 1917b), Knight (1923, 1941, 1966, 1968), and Blatchley (1926). Carvalho (1958), however, in his world catalog, merged Ceratocapsini with the nominate tribe Orthotylini without explanation. Schuh (1974) followed Carvalho (1958) by placing many of the ceratocapsine genera, including *Ceratocapsus* Reuter and *Pilophoropsis* Poppius, and his *Sericophanes* group, a collection of genera he considered held together mostly by myrmecomorphic attributes. Since then, Carvalho et al. (1983) and Henry (1978, 1979, 1985, 1994, 2006) have offered additional support for the tribal status of the group, based largely on a combination of characters, including gentialia. More recently, Schuh (2014) recognized Ceratocapsini in his on-line catalog to include the five genera *Ceratocapsus*, *Pamillia* Uhler, *Pilophoropsis*, *Renodaeus* Distant, and *Schaffneria* Knight, explaining that further study was needed to better define monophyletic groups in the Orthotylinae.

In a preliminary cladistic analysis of the Orthotylinae now in preparation, a minimum of five tribes are recognised: Austromirini (Australian Region), Ceratocapsini (New World), Halticini (worldwide), Nichomachini (Afrotropical), and a paraphyletic Orthotylini (worldwide). The Ceratocapsini are defined by the truncate posterior margin of the head, with a sharp basal carina on the vertex; an endosoma lacking or having only one simple spicule; and the well-developed phallotheca that is visible externally when viewing the genital capsule caudally.

The reduction or lack of spicules in the endomsoma is unique within the subfamily; all other Orthotylinae have multiple endosomal sclerites, with the exception of *Pseudopsallus tiquiliae* Schwartz (2005) that also apparently lacks spicules. The Halticini have a reduced number of spicules (Tatarnic and Cassis 2012), usually with two or more, but they lack the characteristic phallotheca that protrudes externally, among numerous other differences. Several other genus-group taxa (e.g., certain Australian Austromirini and the Argentinian and Chilean genus *Tuxenella* Carvalho) have exposed phallothecae that are distally quadrate, a condition similar to the basal clades in Ceratocapsini. In all of these genera, however, the endosoma has multiple, complex spicules. Cassis (2008) documented that the austromirines are distinct from *Tuxenella* and other New World genera, including the Ceratocapsini, based on endosomal characteristics (e.g., numerous branched spicules) and the tumose peritreme of the metathoracic scent gland system.

In this paper, a monophyletic group of taxa within the Ceratocapsini is defined, herein referred to as the *Renodaeus*-genus group. Included are descriptions of seven genera, four of which are described as new, and 48 species, 26 of which are described as new. I describe the new genus *Ceratocapsidea* to accommodate eight new species and transfer into it *Ceratocapsus balli* Knight (as the type species), *Ceratocapsus complicatus* Knight, *Ceratocapsidea consimilis* Reuter, *Ceratocapsus fusiformis* Van Duzee, *Ceratocapsus nigropiceus* Reuter, and *Ceratocapsus rufistigmus* Blatchley; treat *Ceratocapsus clavicornis* Knight and *Ceratocapsus divaricatus* Knight as junior synonyms of *Ceratocapsus fusiformis* Van Duzee; redescribe and revise the genus *Pilophoropsis*, transfer *Renodaeus texanus* Knight into it, and describe three new species from Arizona and Mexico; describe the new genus *Pilophoropsidea* to accommodate 12 new species from Arizona and Mexico, and transfer into it *Ceratocapsus barberi* Knight, *Ceratocapsus camelus* Knight (as the type species), and *Ceratocapsus fascipennis* Knight; describe the new genus *Pilophoropsita* to accommodate one new species from Mexico; redescribe the genus *Renodaeus* Distant and describe one new species from Ecuador; review the genus *Zanchisme* Kirkaldy; and describe the new genus *Zanchismeopsidea* to accommodate one new species from Argentina. Habitus illustrations are provided for the adults of *Pilophoropsidea camela*, *Pilophoropsis brachyptera*, *Renodaeus mimeticus*, sp. n., and *Zanchisme mexicanus* Carvalho and Schaffner, dorsal adult color digital images and figures of male genitalia of all species, electron photomicrographs of certain diagnostic characters for selected species, and keys to the genera and all included species. The taxa in this paper are arranged alphabetically by genus and species.

## Materials and methods

Male genital capsules were dissected and placed in room-temperature, 10% KOH solution for one to two days or until softened and cleared, after which they were rinsed in water and placed in a depression slide containing glycerol. The endosoma, right and left parameres, and phallotheca were dissected and pencil sketched using a Nikon E400 compound microscrope and drawing tube. Final illustrations were digitally rendered using Adobe Photoshop CS4.

Photomicrographs were taken using either an AMRAY 1810 or a Zeiss EVO/MA15 scanning electron microscope (SEM). Specimens were glued to standard SEM stubs, sputter coated, and examined at 6–10 KV.

Color plates of adults were captured using an EntoVision Imaging Suite that included a JAI Technologies (AT-200GE) digital camera mounted to a Leica Z16 zoom lens via a Leica z-step microscope stand. Multiple focal planes were merged using Cartograph 8.0.6 (Microvision Instruments, France) software.

Color plates of adults (not to scale), SEM photomicrographs, and male genitalia were created using Adobe Photoshop CS4 and numbered in Adobe Illustrator CS4.

Matrix code labels were attached to more than 1,000 specimens examined. These codes, referred to as unique specimen identifers (USIs), are a way to uniquely identify specimens and are stored in a database developed for the NSF Planetary Biodiversity Project awarded 2003–2011 (http://research.amnh.org/pbi/) to R. T. Schuh at the American Museum of Natural History and G. Cassis at the University of New South Wales, Sydney. The full code contains the prefix “AMNH_PBI, an eight-digit number, and the specimen depository, for example (AMNH_PBI 00162206) (USNM)”. USI codes are included in the specimen data listed at the end of each species treatment. To save space, the repetitive “AMNH_PBI” prefix has been omitted. Data for several hundred additional specimens also were recorded, but matrix code labels were not available for these collections (e.g., BNHM) at the time the data were captured and, thus, were not entered into the database and lack USI numbers.

The number of specimens measured equals “n”, in addition to the holotype measurements given in parentheses. For example, “*Male* (n = 4; plus holotype measurements in parentheses): Length to apex of membrane 3.30–3.58 mm (3.50 mm)” means three specimens ranged from 3.30–3.58 mm and the holotype measured 3.50 mm. Structural measurements were taken as follows: Length = from apex of clypeus to apex of hemelytral membrane, or when specified for brachypters, from apex of clypeus to base of cuneus; width = widest width across hemelytra. Head width = greatest width across eyes; interocular width = greatest width between eyes. Labium length = length from base of labrum to apex of segment IV. Antennal segments = actual measurement between articulations. Pronotum length = median length; basal width = width across posterior margin.

### Lending institutions

The following abbreviations are for institutions that kindly lent specimens cited in this paper:

AMNH American Museum of Natural History, New York, NY; Randall T. Schuh

CAS California Academy of Sciences, San Francisco, CA; the late Paul Arnaud, Norman Penny, and Keve J. Ribardo

CDFA California Department of Food and Agriculture, Sacramento; Alan Hardy and Rosser Garrison

CNC Canadian National Collection, Agriculture Canada, Ottawa, Ontario; the late Leonard A. Kelton and Michael D. Schwartz

CUIC Cornell University, Ithaca, NY; E. Richard Hoebeke and James K. Liebherr

FSCA Florida State Collection of Arthropods, Florida Department of Agriculture and Consumer Services, Gainesville, Florida; Susan E. Halbert and Julieta Brambila (the latter now with the U.S. Department of Agriculture)

JTP Colorado State Museum, Englewood, CO; the late John T. Polhemus

BNHM [British] Natural History Museum, London; Mick Webb

IES Instituto de Ecología y Sistemática, La Habana, Cuba; through Luis M. Hernandez (BNHM)

MACN División Entomología, Museo Argentino de Ciencias Naturales “B. Rivadavia”, Buenos Aires, Argentina; Diego L. Carpintero

MNHN Museu Nacional de Historia Natural, Rio de Janeiro; Luiz A. A. Costa

PU Purdue University, West Lafayette, Indiana; Arwin Provonsha

SMNH Swedish Museum of Natural History [Naturhistoriska Riksmuseet], Stockholm, Sweden; Bert Viklund and Gunvi Lindberg

TAMU Texas A & M University, College Station, TX; Joseph C. Schaffner

UNAM Instituto de Biologia, Universidad Nacional Autonoma de Mexico, Mexico, D.F.; Harry Brailovsky

USNM [United States] National Museum of Natural History, Washington, DC; the author, Thomas J. Henry

USU Utah State University, Logan; the late Wilford J. Hanson

ZMUH Zoological Museum, University of Helsinki, Helsinki, Finland; Larry Huldén and the late Antti Jansson

### Misplaced taxa

Schuh (1995, 2014), in his world catalog, inadvertently placed *Zanchismella* Carvalho & Wallerstein (1975) and the only included species *Zanchismella bispinosa* Carvalho & Wallerstein, and *Zanchismisca* Carvalho & Wallerstein (1975) and the only included species *Zanchismella guanabarina* Carvalho & Wallerstein in the nominate tribe Orthotylini (Orthotylinae). Study of type material of both taxa in the USNM collection in preparation for this study indicated that they clearly belong in the deraeocorine tribe Surinamellini where they were correctly placed by Carvalho and Wallerstein (1975).

## Systematics

### *Renodaeus* group

**Included genera.** *Ceratocapsidea* gen. n., *Marinonicoris* Carvalho, *Pilophoropsidea* gen. n., *Pilophoropsis* Poppius, *Pilophoropsita* gen. n., *Renodaeus* Distant, *Zanchisme* Kirkaldy, and *Zanchismeopsidea* gen. n.

**Diagnosis.** The genera belonging in the *Renodaeus* group are all strongly to moderately ant mimetic, both as adults and nymphs. The *Renodaeus* group forms a monophyletic part of the tribe Ceratocapsini based on the shiny, dark brown to fuscous dorsum; the often weakly to strongly constricted or concave hemelytral margins (Figs 56, 92, 100); the presence of scattered scale-like setae usually in the form of dorsal bands or patches of silvery, scale-like setae, often intermixed with erect, bristle-like setae (Figs 8, 130, 144); the glaucous patch in most taxa at the base of abdominal segments II and/or III comprised of tiny spicules (Figs 112, 143); and the common pattern of the complex male genitalia (Figs 159, 162), usually composed of a two- or three-branched left paramere, a variably branched right paramere, and an apically acute, often bifid or otherwise distally complex phallotheca (Fig. 160) The endosoma in all of these taxa has a simple ductus seminus, with a distinct secondary gonopore but otherwise lacks any diagnostic accessary structures (i.e., spicules). All members of the Ceratocapsini possess a well-developed phallotheca (Fig. 146) that protrudes from the genital capsule and is always visible externally when viewed caudally, though ceratocapsines outside the *Renodaeus* group lack the distinctive distal modifications.

The female genitalia are not treated in this study but will be included in a forthcoming phylogenetic analysis of the tribe Ceratocapsini and its position within the Othotylinae. Slater (1950) noted that it was impossible to distinguish members of the Lopidini (now a synonym of Orthotylini), Orthotylini, and Ceratocapsini, based on the female genitalia, though later in the same paper he indicated that *Ceratocapsus modestus* (Uhler) and *Ceratocapsus fasciatus* (Uhler) had specific differences but, apparently, little in the way of generic characters. Interestingly, I have determined that these two species are not congeneric, suggesting that the specific differences noted by Slater (1950) may translate into synapomorphies useful in distinguishing genus-group taxa within the Ceratocapsini, including the genera treated in this paper. Schaffner and Schwartz (2008) [*Ficinus* Distant and *Jornandes* Distant], Forero (2008) [*Hadronema* group], and Schwartz (2011) [*Scalponotaus* Kelton and *Slaterocoris* Wagner] provided additional evidence that female genitalic characters offer broad resolution at the generic level, further suggesting they may be helpful in resolving relationships within the Ceratocapsini.

Some genera belonging in the *Renodaeus* group exhibit considerable sexual dimorphism, with the females of *Pilophoropsidea* and *Pilophoropsis* always strongly brachypterous. Study of other generic groups within Ceratocapsini shows varying degrees of sexual dimorphism (with brachyptery occurring only in females) from the extreme found in *Pilophoropsidea* and *Pilophoropsis* to very little in species of *Ceratocapsidea* and *Zanchisme*. The combination of external characters shared by these taxa, particularly the sharply truncate, basally carinate head, the overall dark coloration, and often constricted hemelytra having patterns of white or silvery scale-like setae creates an overall ant-like appearance that is impressive, even within the Miridae.

#### Key to the genera of the *Renodaeus* group

**Table:** 

1	Anterior pronotal lobe strongly narrowed, almost neck-like (Figs 86, 97)	**2**
–	Anterior pronotal lobe not strongly narrowed or neck-like, gradually tapering from posterior lobe (Figs 24, 73)	**4**
2	Labial segment I extending posteriorly beyond base of head (Fig. 87); hemelytron with silvery, scale-like setae evenly scattered over clavus and corium (Figs 86, 88), rather than forming distinct bands or patches	***Pilophoropsita* gen. n.**
–	Labial segment I not reaching base of head (Figs 18, 108); hemelytron with silvery scale-like setae forming distinct bands and/or dense patches (Figs 49, 74)	**3**
3	Scutellum flat, not rising above level of hemelytra (Figs 96, 103); basal half of clavus always with a band of silvery scale-like setae bordering scutellum; length of posterior pronotal lobe 1.7 times or less the length of anterior lobe	***Zanchisme* Kirkaldy**
–	Scutellum conical, distinctly rising above level of hemelytra (Fig. 106); basal half of clavus lacking a band of silvery scale-like setae bordering scutellum; length of posterior pronotal lobe 2 times the length of anterior lobe	***Zanchismeopsidea* gen. n.**
4	Hemelytron with silvery, scale-like setae scattered over clavus and corium, never forming distinct bands or patches	***Ceratocapsidea* gen. n.**
–	Hemelytron with silvery scale-like setae forming bands and/or distinct patches	**5**
5	Hemelytron with distinct bands and patches of silvery scale-like setae; females always strongly brachypterous	**6**
–	Hemelytron with a dense patch of golden scale-like setae through clavus and middle of corium, in addition to silvery scale-like bands; females macropterous	**7**
6	Hemelytral surface mostly dull, shiny only along costal margin and on cuneus; silvery scale-like setae forming tight shiny bands and small patches	***Pilophoropsis* Poppius**
–	Hemelytral surface uniformly shiny; silvery scale-like setae forming relatively loose bands, one narrow one across base of clavus and a broad loose one through middle of corium and apex of clavus	***Pilophoropsidea* gen. n.**
7	Hemelytron with long, erect, bristle-like setae on clavus and corium; outer edge of costal margin with a distinct row of tiny, black stridulatory spicules	***Renodaeus* Distant**
–	Hemelytron without long, bristle-like setae; outer edge of costal margin without black spicules	***Marinonicoris* Carvalho**

#### Ceratocapsidea

Taxon classificationAnimaliaHemipteraMiridae

Henry gen. n.

http://zoobank.org/E771BEA6-9D04-434D-BE7C-ECF990EDCBA2

##### Type species:

*Ceratocapsus balli* Knight, 1927.

##### Diagnosis.

This genus is distinguished by a combination of an often shiny dorsum, usually polished head and pronotum; the generally thickened antennal segments I–III, especially in males; the weakly rounded, subparallel to weakly constricted hemelytra with distinct, uniformly distributed, usually brown-stained punctures, often with erect, slender to stout bristle-like setae, intermixed with slender, pale or silvery scale-like setae, especially on the clavus and basal half of the corium; the distinctive stridulatory patch ventrally on abdominal segments II and III; and the distinctive male genitalia, with the main body of the right paramere stout with one or more variously shaped lateral arms, the left paramere usually with one or two dorsal spines or variously shaped processes and the left-most process usually forming a hammer- or beak-like process; and the phallotheca usually apically acute, bifid, or hooked.

##### Description.

Males and females macropterous. Length of males 2.82–3.71 mm; length of females 2.27–3.58 mm. *Head*: Broader than long; posterior margin truncate to slightly concave, narrowly carinate, posterior margin of eyes level with base of vertex; eye large, broadly rounded, coarsely faceted, dorsal width of one eye greater than interocular width, lateral height in males equal or subequal (0.90) to height of head, from about one half to slightly less than three fourths (0.70) height in females; frons broad, shiny to dull, smooth to transversely rugose; clypeus moderately acute, visible from dorsal aspect; scattered with semierect and erect, simple setae. *Labium*: Segment I long, extending posteriorly well beyond base of head, with only basal half enclosed within buccula; segment IV extending to middle or hind coxae. *Antenna*: Slender to weakly thickened, especially in males; segment I shortest; segment II longest, slender basally, gradually enlarging to apex, sometimes weakly clavate; segment III slightly longer than IV, both subequal to apical diameter of segment II. *Pronotum*: Trapeziform, lateral margins straight to weakly sulcate, posterior margin weakly rounded; dorsum dull to shiny, finely to coarsely punctate, sometimes alutaceous to weakly rugose; calli indistinct; set with scattered erect and semierect, simple setae, often intermixed with short, silvery, sericeous setae. *Mesoscutum*: Covered by posterior margin of pronotum. *Scutellum*: Equilateral; evenly punctate, mixed with simple and silvery sericeous setae. *Hemelytra*: Weakly rounded to subparallel, sometimes weakly constricted through middle, especially in males; distinctly punctate, shiny to more dull; thickly set with short recumbent simple and silvery sericeous setae, often intermixed with long, erect, sometimes nearly bristle-like setae; all known species fully macropterous. *Ventral surface*: Shiny; clothed with short, recumbent, simple setae; base of abdominal segment III (second visible segment) in most species with a distinct stridulatory patch (Fig. 112) composed of tiny spicules or microspines. *Ostiolar evaporative area*: Pale, knob at end of scent channel relatively small, sometimes more reddish. *Legs*: Slender, unmodified. *Male genitalia*: Aperture large, open, unarmed; stridulatory patch ventrally on abdominal segments II and/or III distinct in most species; generalized left paramere usually with a large hammer- or beak-like process distally and one or two more basal short acute processes; right paramere with a main trunk and one to three variously shaped lateral arms; phallotheca slender with apex broadly pointed or ending in a slender acute or bifid process; endosoma simple, unmodified.

##### Etymology.

The name of this new genus is taken from the generic name *Ceratocapsus* Reuter and Latin suffix “*idea*”, meaning form or appearance, and is used to reflect the overall resemblance of the two genera. The gender is feminine.

##### Discussion.

Some members of this genus group are the least myrmecomorphic of the genera included in the *Renodaeus* group (e.g., *Ceratocapsidea complicata*, *Ceratocapsidea rufistigma*, *Ceratocapsidea texensis*). Nevertheless, the male genitalia hold these less-ant-like species together as a group close to the other seven genera treated in this paper.

The following key includes external morphology when possible to help distinguish species, though male genitalia are necessary to identity very similar species.

##### Key to the species of *Ceratocapsidea*

**Table:** 

1	Pronotum polished, finely punctate, and with only simple setae	**2**
–	Pronotum dull to somewhat shiny, usually coarsely punctate, often rugose, with both simple and silvery sericeous setae	**5**
2	Antennal segment I and II dark brown to fuscous	**3**
–	Antennal segment I and II yellowish to yellowish brown	**4**
3	Left paramere (Fig. 159) with two middle processes, beak-like process curved downward on each side; right paramere (Figs 161, 162) with a deep subapical notch, upper arm relatively short, straight, and slender; phallotheca (Fig. 160) gradually narrowing and curving distally; Jamaica	***baranowskii* sp. n.**
–	Left paramere (Fig. 208) with three middle processes, beak-like process curved upward distally; right paramere (Fig. 210) with a small, indistinct, subapical notch, upper arm long, curving upward; phallotheca (Fig. 209) thickened distally, then narrowing into a long, narrow process; Jamaica	***variabilis* sp. n.**
4	Antennal segment II length subequal to width of head across eyes; right paramere with one lateral arm (Fig. 158); phallotheca (Fig. 157) pointed distally; southeastern United States, west to Texas	***balli* (Knight)**
–	Antennal segment II length much greater than width of head across eyes; right paramere (Fig. 177) with two lateral arms; phallotheca (Fig. 176) truncate distally, with a serrate margin; Dominican Republic	***dominicanensis* sp. n.**
5	Pronotum dark brown to fuscous; hemelytra usually subparallel to weakly constricted through middle	**6**
–	Pronotum pale brown to reddish brown, sometimes with dark lines, spots, or other areas leaving midline pale; hemelytra laterally rounded, more strongly so in females	**11**
6	Antennal segments I and II dark brown to fuscous; left paramere (Fig. 189) with beak-like process broadly rounded; right paramere (Fig. 191) with one, relatively short, upturned lateral arm; phallotheca stout, broadly pointed distally (Fig. 190); United States (Texas)	***rileyi* sp. n.**
–	Antennal segment I and II yellowish to pale brown	**7**
7	Hemelytra pale yellowish brown on basal half, distal half of corium and cuneus contrasting dark brown	**8**
–	Hemelytra uniformly colored, costal margin and cuneus sometimes more reddish.	**9**
8	Left paramere (Fig. 171) with beak-like process blunt distally; right paramere (Figs 173, 174) with one large recurving arm and two smaller, marginally crenulate processes above; phallotheca (Fig. 172) broad with a short, blunt apical process; Cuba	***cubana* (Bergroth)**
–	Left paramere (Fig. 182) lacking a beak-like process apically; right paramere (Figs 184, 185) with two lateral arms, each with a short, marginally crenulate process; phallotheca (Fig. 183) evenly slender with a long, blunt, apical process; Cuba	***holguinensis* (Hernández & Henry)**
9	Left paramere (Fig. 178) with beak-like process blunt apically; right paramere (Figs 180, 181) with one, stout, apically bifurcate lateral arm; phallotheca (Fig. 179) stout, distally bifurcate with the upper-most process the longest; southern and western United States	***fusiformis* (Van Duzee)**
–	Left paramere with beak-like process pointed on either side; right paramere and phallotheca different from above	**10**
10	Left paramere (Fig. 186) with two middle processes, one stout, directed outward, one longer, reclining; right paramere (Fig. 188) with one stout lateral process, recurving at apex; phallotheca (Fig. 187) long, slender, apically bifid; Jamaica	***nigropicea* (Reuter)**
–	Left paramere (Fig. 196) with one stout middle process directed outward; right paramere (Figs 198, 199) with two lateral arms curving upward apically; phallotheca (Fig. 197) with a long, tapered apex and an incised subapical process; Jamaica	***taeniola* sp. n.**
11	Pronotum uniformly yellowish brown to reddish brown	**12**
–	Pronotum yellowish brown to reddish brown but variously marked with lines, spots, or other dark areas	
12	Broadly rounded, uniformly reddishbrown species (Figs 45, 46); legs uniformly yellow; left paramere (Fig. 200) with two middle processes; right paramere (Figs 202, 203) with two stout lateral arms; phallotheca (Fig. 201) with a long, slender apical process; United States (Texas)	***texensis* sp. n.**
–	More slender, yellowishbrown species (Fig. 32); fore and middle femora yellow, hind femur dark brown; left paramere (Fig. 167) with three middle processes; right paramere (Figs 169, 170) with one complex arm with a large bifid process arising at base; phallotheca (Fig. 168) stout, with a curved, blade-like process at apex; Jamaica	***consimilis* (Reuter)**
13	Pronotum (Figs 30, 31) yellowish brown with a dark brown spot behind each callus; left paramere (Fig. 163) with beak-like process long and pointed on either side; right paramere (Figs 165, 166) with one very stout, bifurcate lateral arm; phallotheca (Fig. 164) deeply bifurcate apically; eastern United States	***complicata* (Knight)**
–	Pronotum and genitalia not as above	**14**
14	Pronotum with a broad, dark brown, transverse line through middle; left paramere (Fig. 204) with beak-like process pointed on either side; right paramere (Figs 206, 207) two lateral arms, the upper one shortest and most slender; phallotheca (Fig. 205) with a long, slender apex and a marginally crenulate subapica process; Mexico (Neuvo León)	***transversa* sp. n.**
–	Pronotum brown on either side leaving midline pale, overall color yellowish brown; left paramere (Fig. 192) with beak-like process elongate, rounded behind; right paramere (Figs 194, 195) with one thick lateral arm; phallotheca (Fig. 193) stout on apical half, with a slender, recurved process at apex; United States (Florida, North Carolina)	***rufistigma* (Blatchley)**

#### Ceratocapsidea alayoi

Taxon classificationAnimaliaHemipteraMiridae

(Hernández & Henry) comb. n.

Figs 24 , 148–151

Ceratocapsus alayoi Hernández & Henry, 1999: 207 (orig. descrip.); Hernández and Henry 2010: 105 (diag., habitus, genitalia).

##### Diagnosis.

*Ceratocapsidea alayoi* (Fig. 24) is distinguished by the brownishyellow pronotum, red-tinged vertex, and apically hammer-shaped left paramere (Fig. 148), with a bifid process at the middle, and the right paramere (Fig. 151) with two long, recurving arms and a large crenulate inner process.

##### Description.

*Holotype male* (after Hernández and Henry 1999; abdomen and wings detached): Length about 3.30 mm. *Head*: Length 0.52 mm, width across eyes 0.82 mm, interocular width 0.22 mm. *Labium*: Length (measurement not given). *Antenna*: Segment I, length 0.30 mm; II, 1.05 mm; III, 0.45; IV, missing. *Pronotum*: Length 0.73 mm; basal width 1.06 mm.

*Coloration*: General coloration brownish yellow with dark brown areas on posterior margin of pronotum and hemelytron. *Head*: Castaneous. *Antenna*: Segment I yellow, II and III pale brown, IV missing. *Labium*: Segments I and II red, III and IV missing. *Pronotum*: Shiny, brown anteriorly and darker brown to lateral angles; calli obsolete. *Scutellum*: Yellow, finely punctate. *Hemelytron*: Basal half of corium yellow, clavus and remainder of corium brown, claval commissure brown, cuneus dark brown, membrane dark translucent gray or brown. *Ventral surface*: Mesosternum reddish brown, abdominal segments reddish brown mesally, brown laterally. *Ostiolar evaporative area*: Pale or white. *Legs*: Yellow, procoxae reddish brown.

*Structure, texture, and vestiture*: *Head*: Smooth, impunctate; clypeus weakly produced; eyes prominent, occupying entire lateral height of head. *Labium*: Extending to hind coxa. *Scutellum*: Finely punctate. *Hemelytron*: Punctate, lateral margins subparallel. Body covered with erect brown setae, intermixed with white sericeous or scale-like setae, especially on scutellum and hemelytron.

*Male genitalia*: Left paramere (Fig. 148) slender with two stout, acute processes at middle and a hammer-shaped process at apex. Right paramere (Fig. 151) stout with two long, inward-curving arms and a large crenulate inner process. Phallotheca (Fig. 149): Elongate, gradually tapering to a slender point apically.

*Female*: Length 2.85 mm, width not measured. *Head*: Length 0.60 mm, width across eyes 0.75 mm, interocular width 0.30 mm. *Antenna*: Segment I, length 0.30 mm, yellow, with a red basal dash; remaining segments missing. *Pronotum*: Length 0.60 mm, basal width 0.60 mm.

##### Host.

Unknown.

##### Distribution.

Described and known only from Cuba.

##### Type material examined.

Holotype ♂: **CUBA:** **Guantánamo:** Tortugilla, vii 1975, L. B. Zayas (IES); paratype ♀, same data as for holotype (IES).

##### Discussion.

The above information is based in part on Hernández and Henry’s (1999) diagnosis and description. After preparation of the original description, the holotype was returned to IES in Cuba, where it is presently inaccessible for study. Based on the original description, Figures of male genitalia provided by Hernández and Henry (1999, 2010), and study of two paratypes in the USNM collection, *Ceratocapsidea alayoi* clearly belongs in *Ceratocapsidea*.

#### Ceratocapsidea bahamaensis

Taxon classificationAnimaliaHemipteraMiridae

Henry sp. n.

http://zoobank.org/582896C1-393B-4F15-80B8-839F8C2B62CF

Figs 25 , 152–155

##### Diagnosis.

This species (Fig. 25) is best distinguished by the overall brown coloration, impunctate head and pronotum, evenly punctate hemelytron with erect, scattered, simple setae, intermixed with silvery scale-like setae on the clavus and corium, and the distinctive male genitalia, including the bilobed left paramere (Fig. 152), the complex right paramere (Figs 154, 155) with two arms visible from caudal view, one long and slender, and one bifid apically, and a large flared or fan-like inner process, and the relatively simple, apically acute phallotheca (Fig. 153) with a subapical notch.

##### Description.

*Male* (n = 4; holotype measurements in parentheses): Length to apex of membrane 3.30–3.58 mm (3.50 mm), width 1.12–1.28 mm (1.15 mm). *Head*: Width 0.74–0.77 mm (0.74 mm), interocular width 0.26–0.27 mm (0.27 mm). *Labium*: Length 1.31–1.41 mm (1.38 mm). *Antenna*: Segment I, 0.32–0.35 (0.30 mm); II, 0.91–0.96 (0.94 mm); III, 0.48–0.54 (0.54 mm); IV, 0.48–0.50 (0.50 mm). *Pronotum*: Length 0.66–0.69 mm (0.69 mm); basal width 1.06–1.12 mm (1.12 mm).

*Coloration*: *Head*: Yellowish brown to dark brown. *Antenna*: Segment I yellowish brown, with a red dash near base; segment II yellowish brown, apex red; segment IV yellowish brown on basal half, reddish brown on distal half; segment IV reddish brown. *Pronotum*: Anterior lobe dull yellowish brown to dark brown; posterior lobe shiny dark brown. *Scutellum*: Yellowish brown to dark brown. *Hemelytron*: Clavus and basal half of corium yellowish brown, apical half of corium dark brown; cuneus dark brown to reddish brown, except for paler inner angle (paracuneus); membrane pale or whitish on basal half, dark smoky brown on apical half. *Ventral surface*: Overall yellowish brown; abdomen dark shiny brown, paler basally. *Ostiolar evaporative area*: White. *Legs*: Uniformly yellowish brown to dark brown.

*Structure, texture, and vestiture*: Dorsal surface shiny overall. *Head*: Impunctate, vertex transversely finely granulate. *Labium*: Extending to middle of hind coxae or base of abdomen. *Pronotum*: Impunctate, anterior lobe and calli finely granulate, posterior lobe more polished and smooth; scutellum transversely rugose with fine, brown-stained punctures. *Hemelytron*: Evenly scattered with brown-stained punctures. Head and pronotum with recumbent and semierect simple setae; hemelytron with scattered, long, erect, simple setae, intermixed with white, flattened, scale-like setae on clavus and basal half of corium.

*Male genitalia*: Left paramere (Fig. 152) bilobed, right lobe weakly curved and apically acute (tip broken and blunt in holotype); left lobe strongly curved downward and apically pointed. Right paramere (Figs 154, 155) with elongate main stem and two lateral arms, the shorter one bifid apically, inner surface with a wide fan-like process extending downward into a curved spine. Phallotheca and endosoma (Fig. 153) tapering to a point apicially, with a subapical notch.

*Female*: Unknown.

##### Etymology.

Named after the Bahama Islands where the only four known specimens were collected.

##### Host.

Unknown.

##### Distribution.

Known only from Nassau, New Providence Island, Bahama Islands.

##### Type material.

Holotype ♂: **BAHAMAS:** **Nassau:** ***New Providence Co.*:** 24 Jun 1972, F. D. Bennett, blacklight trap (00286325) (USNM). Paratypes: **BAHAMAS:** **Nassau:** ***New Providence Co.*:** 24 Jun 1972, F. D. Bennett, blacklight trap, 1♂ (00286324) (USNM). 16 Apr 1953, E.B. Hayden, at light, 2♂♂ (00286049, 00286050) (AMNH).

#### Ceratocapsidea balli

Taxon classificationAnimaliaHemipteraMiridae

(Knight) comb. n.

Figs 2 , 3–10 , 26–28 , 156–158

Ceratocapsus balli Knight, 1927: 146 (orig. descrip.); Carvalho 1958: 44; Henry and Wheeler 1988: 392 (cat.); Schuh 1995: 90 (cat.).
Ceratocapsus nigropiceus : Henry and Wheeler 1982: 235 (note, host, distr.).

##### Diagnosis.

*Ceratocapsus balli* (Figs 26–28) can be distinguished from all other species of the genus by the impunctate, shiny, brown head and pronotum; punctate hemelytra with numerous white, scale-like setae on the clavus and basal half of the corium; and the male genitalia, especially the left paramere (Fig. 156), with a beak-like distal process and right (Fig. 158) paramere, with an apically hooked lateral arm.

##### Description.

*Male* (n = 10; holotype measurements in parentheses): Length 0.83–1.03 mm (1.01 mm); width 1.06–1.22 mm (1.24 mm). *Head*: 0.67–0.74 mm (0.74 mm), interocular width 0.22–0.26 mm (0.26 mm). *Labium*: Length 0.93–1.06 mm (1.06 mm). *Antenna*: Segment I, length 0.22–0.27 mm (0.27 mm); II, 0.70–0.85 mm (0.85 mm); III, 0.40–0.48 mm (0.43 mm); IV, 0.38–0.40 mm (0.40 mm). *Pronotum*: Length 0.51–0.64 mm (0.64 mm), basal width 0.93–1.12 mm (1.12 mm).

*Coloration*: Overall coloration dark brown to fuscous. *Head*: Dark brown, eyes dark brown or fuscous. *Antenna*: Segment I and basal half to two thirds of II brown to yellowish brown, segments III, IV, and apical third of II usually red tinged. *Pronotum*: Fuscous. *Hemelytron*: Clavus and basal half of corium brown to yellowish brown, becoming dark brown through middle of corium, apical third of embolium, and on cuneus; membrane pale or whitish on basal third, dark smoky gray or brown distally. *Ventral surface*: Dark brown on thorax to fuscous or nearly black on abdomen. *Ostiolar evaporative area*: White, with central knob red. *Legs*: Fore and middle coxae brown, often red tinged, hind coxa paler brown; fore and middle femora yellowish brown to dark brown, hind femur usually dark brown; fore and middle tibiae reddish, pale on apical third to fourth, hind tibia uniformly reddish; tarsi and claws yellowish brown.

*Structure, texture, and vestiture*: *Head*: Impunctate, shiny. *Labium*: Extending to bases of hind coxae. *Pronotum*: Impunctate, shiny, smooth, slightly transversely wrinkled near collar, calli smooth, impunctate; scutellum finely punctate and transversely rugose. *Hemelytron*: Uniformly and finely punctate. Head with scattered simple, recumbent simple setae; pronotum with numerous, relatively long, semierect simple setae; scutellum with numerous, small, white, scale-like setae, intermixed with a few long, erect, simple setae; hemelytra densely covered with small, white, scale-like setae, mostly on clavus and basal half of corium through paler brown areas, intermixed with scattered, relatively long, semierect, simple setae.

*Male genitalia*: Left paramere (Fig. 156) elongate, with a well-developed beak-like distal process and two short, stout, apically acute processes at middle. Right paramere (Fig. 158) relatively slender, with a long, upward-curving and apically hooked lateral arm. Phallotheca (Fig. 157) relatively stout, weakly curved, and apically acute.

*Female* (n = 3): Length 2.82–2.88 mm, width 1.15–1.20 mm. *Head*: Width 0.62–0.64 mm, interocular width 0.29 mm. *Labium*: Length 0.96 mm. *Antenna*: Segment I, length 0.24–0.26 mm; II, 0.72–0.74 mm; III, 0.45 mm; IV, 0.40 mm. *Pronotum*: Length 0.58 mm, basal width 0.99–1.02 mm.

##### Hosts.

Most specimens studied are males that have been taken at light. Henry and Wheeler (1982) beat a specimen from *Batis maritima* L.[Bataceae] in Upper Key Largo. In Florida, three specimens were taken on live oak, *Quercus virginiana* Mill. [Fagaceae], and three were swept from ferns.

##### Distribution.

This species was described and previously known only from Florida (Knight 1927). New records are Arkansas, Georgia, Louisiana, North Carolina, Tennessee, and Texas.

##### Discussion.

Henry and Wheeler (1982) based their record of *Ceratocapsus nigropiceus* in Florida on comparison of the undissected holotype from Jamaica with locally collected material from South Florida. Re-examination of a male paratype and other specimens of *Ceratocapsus nigropiceus* from Jamaica shows that this species is similar to *Ceratocapsus balli* in overall size, general appearance, and the shape of the left (Fig. 186) and right parameres (Fig. 188). More detailed examination of dissected specimens, however, shows that the two species are quite distinct, especially in the stouter, apically acute phallotheca of *Ceratocapsus balli* (Fig. 157) as compared with a more slender and apically bifid phallotheca in *Ceratocapsus nigropiceus*. As a consequence, reference is made to Henry and Wheeler’s (1982) Florida record of *Ceratocapsus nigropiceus* to *Ceratocapsus balli*.

In addition, a small series of specimens taken at lights in the Gainesville, Florida, area differ in details of the right and left parameres from the typical *Ceratocapsus balli*. The right paramere has a distinct elongate process at the base of the lateral arm, the apex of the lateral arm is less hooked, and the main trunk has a broad subapical flange. The left paramere has a broader beak-like apex and the two processes at the middle of the main trunk are longer, more slender, and sharply pointed apically. Examination of additional material to determine variability of these structures may eventually reveal that this population is distinct from *Ceratocapsus balli*.

##### Type material examined.

Holotype ♂: **USA:** **Florida:** ***Seminole Co.*:** Sanford, 15 May 1926, E. D. Ball (00162209) (USNM). Paratypes: **USA:** **Florida:** ***Alachua Co.*:** Agricultural Research Station, Univ. Florida, Gainesville, 29.64254EN, 82.32289EW, 12 m, 09 May 1918, C. J. Drake, 1♂ (00329539) (CNC), 1♀ (02467780) (TAMU). ***Seminole Co.*:** Sanford, 28.80055EN, 81.27312EW, 01 Jun 1926, E. D. Ball, 1♀ (00329540) (CNC), 3♀♀ (00071407, 00071408, 00071356) (USNM). Sanford, 28.7884EN, 81.2544EW, 12 m, 17 Aug 1927, E. D. Ball, 1♂ (00246779) (TAMU). ***Volusia Co.*:** Orange City, 28.94852EN, 81.29883EW, 8 m, 05 May 1928, E. D. Ball, 1♀ (02467781) (TAMU).

##### Other specimens examined.

**USA:** **Arkansas:** ***Garland Co.*:** Camp Clearfork Ouachita National Forest, ca. 1 km S. of U.S. route 270, area ca. 30 km W. Hot Springs, 34.85EN, 93.65EW, 25 Jun 2008, Brian Baldwin, 2♂♂ (00286327, 00286328) (USNM). **Florida:** ***Alachua Co.*:** Gainesville, 29EN, 82EW, 13 May 1977–15 May 1977, F. W. Mead, 3♂♂ (00071413- 00071415) (USNM); 20 May 1977–22 May 1977, F. W. Mead, 1♂ (00286052), 2♂♂ (00071419, 00071420), 1♀ (00071421) (USNM); 03 Jun 1977–05 Jun 1977, F. W. Mead, 1♂ (00286053) (USNM); 09 Jun 1978–11 Jun 1978, F. W. Mead, 2♂♂ (00071416, 00071417) (USNM); 08 Sep 1978–10 Sep 1978, F. W. Mead, 1♂ (00071418) (USNM). Gainesville, 29.63527EN, 82.37111EW, 24 m, 05 May 1977, F. W. Mead, 1♂ (00071437) (USNM); 22 May 1986–26 May 1986, F. W. Mead, 1♂ (00071439) (USNM); 10 Dec 1997, F. W. Mead, 1♂ (00071438) (USNM). Gainesville, 29EN, 82EW, 20 May 1977–22 May 1977, F. W. Mead, 1♂ (00286013) (USNM). ***Hendry Co.*:** LaBelle, 26.76055EN, 81.43916EW, 16 Jul 1939, Oman, fern, 1♂ (00071429) (USNM). ***Highlands Co.*:** Archbold Biological Station, 27.18833EN, 81.33778EW, 12 Mar 1971, S. W. Frost, 1♂ (00071423) (USNM). Highlands Hammock State Park, 27.47138EN, 81.54138EW, 26 m, 22 Apr 1981–28 Apr 1981, T. J. Henry, swept from fern, 1♀ (00071424) (USNM). ***Marion Co.*:** 9 mi SSW of Ocala, 29.819EN, 82.187EW, 27 Aug 1975, Drummond & Wiley, fern, 1♀ (00071428) (USNM). Lake Eaton, BLT, 29.25861EN, 81.86444EW, 10 m, 08 Aug 1975–25 Aug 1975, J. & S. Knox, black light, 1♂ (00071422) (USNM). ***Monroe Co.*:** Bahia Honda Key, 27.73722EN, 82.73583EW, 1 m, 10 Jun 1968, F. E. Wood, black light, 1♂ (00071425) (USNM). Upper Key Largo, 25.124EN, 80.41502EW, 2 m, 17 Apr 1982, T. J. Henry and A. G. Wheeler, Jr, *Batis maritima* (Bataceae), 1♂ (00286055) (USNM). ***Seminole Co.*:** Sanford; 17 Apr 1927, E. D. Ball, 1♂ (00286051), 1♀ (00071409) (USNM); 05 May 1927, E. D. Ball, 1♂ (00071410) (USNM); 15 May 1927, E. D. Ball, *Quercus* sp. (Fagaceae), det. E.D. Ball, 1♀ (00071360) (USNM). ***Volusia Co.*:** Orange City, 28.94852EN, 81.29883EW, 8 m, 05 May 1928, E. D. Ball, 2♂♂ (00071411, 00071412) (USNM). Rt. 415, 2 miles N. Osteen, 28.87888EN, 81.1525EW, 27 Apr 1984–28 Apr 1984, T. J. Henry, J. T. Polhemus and A. G. Wheeler Jr., *Quercus virginiana* (Fagaceae), 1♀ (00071435), 1♂ (00071436), light trap, 1♀ (00286016) (USNM); 27 Apr 1984, T. J. Henry, J. T. Polhemus and A. G. Wheeler Jr., *Quercus virginiana* (Fagaceae), 1♂ (00286054) (USNM). **Georgia:** ***Peach Co.*:** Peach County, 32.5666EN, 83.8333EW, 12 Jul 1943, Turner, light trap, 1♂ (00071427) (USNM); 12 Jul 1943, Turner, light trap, 1♀ (00071426) (USNM). **Louisiana:** ***Baton Rouge Parish*:** E. Baton Rouge, 24–25 July 1998, E. G. Riley, at UV light (TAMU). **North Carolina:** ***Columbus Co.*:** Lake Waccamaw, 34.32194EN, 78.52194EW, 18 m, 04 Jul 1985–06 Jul 1985, W. Steiner & A. Gerberich, at blacklight in oak & pine scrub sand barren near lake, 1♀ (00071430), 2♂♂ (00071431, 00071432) (USNM). **Tennessee:** ***Hamilton Co.*:** Hamilton County, 35.22111EN, 85.20888EW, 24 Jun 1943–15 Jul 1943, Turner, light trap, 1♀ (00071433), 1♂ (00071434) (USNM). **Texas:** ***Brazos Co.*:** College Station, Riley Estate, 30.58833EN, 96.25333EW, 18 May 2006–19 May 2006, K. Menard, light & sheet, 2♂♂ (00286014, 00286015) (USNM).

Gainesville specimens showing genitalic variation discussed above: **Florida:** 1♂, ***Alachua Co.***, Gainesville [Doyle Conner Building], 5 May 1977, F. W. Mead (USNM); 1♂, same locality, 10 Dec. 1997, no collector (FSCA); 1♂, same locality, 22–26 May 1986, F. W. Mead. (FSCA).

#### Ceratocapsidea baranowskii

Taxon classificationAnimaliaHemipteraMiridae

Henry sp. n.

http://zoobank.org/EC0B6153-7BAA-4E9D-9435-440D6606DBE8

Figs 29 , 159–162

##### Diagnosis.

This species (Fig. 29) is distinguished by the dark brown head; shiny, finely punctate, dark brown pronotum; slightly paler brown hemelytra, with numerous white scale-like setae, intermixed with relatively long, semierect simple setae; and the male genitalia, especially the left paramere (Fig. 159) with a small, beak-like, apical process and two middle processes with the basal one erect and directed outward, and the complex right paramere (Figs 161, 162) with multiple arms and processes.

##### Description.

*Male* (n = 6; holotype measurements in parentheses): Length 3.30–3.65 mm (3.65 mm), width 1.09–1.22 mm (1.15 mm). *Head*: Width 0.75–0.78 mm (0.77 mm), interocular width 0.21–0.22 mm (0.22 mm). *Labium*: Length 1.17–1.23 mm (1.23 mm). *Antenna*: Segment I, length 0.17–0.18 mm (0.18 mm); II, 0.77–0.85 mm (0.85 mm); III, 0.38–0.40 mm (0.40 mm); IV, 0.38–0.42 mm (0.42 mm). *Pronotum*: Length 0.64–0.67 mm (0.67 mm), basal width 1.15–1.20 mm (1.20 mm).

*Coloration*: Overall coloration brown to dark brown. *Head*: Dark brown, eyes grayish brown, weakly tinged with red on some specimens. *Pronotum*: Dark brown; mesoscutum brown, scutellum brown, becoming paler yellowish brown on distal half. *Hemelytron*: Brown to yellowish brown, cuneus dark brown; membrane and vein translucent brown, paler along basal margins. *Ventral surface*: Thoracic segment dark brown; abdomen brown to yellowish brown. *Ostiolar evaporative area*: Uniformly yellowish brown. *Legs*: Coxae yellowish brown; femora pale yellowish brown on basal fourth, fore and middle femora brown distally, hind femur dark brown distally; tibiae dark brown; tarsi and claws brown to yellowish brown.

*Structure, texture, and vestiture*: *Head*: Finely faceted, with scattered, short, recumbent simple setae. *Antenna*: Relatively thickened, segment IV and apical diameters of segments II and III subequal to diameter of segment I. *Labium*: Extending to bases of hind coxae. *Pronotum*: Shiny, evenly and finely punctate, except for finely granulate calli; with scattered, short, recumbent simple setae, intermixed with a few longer, erect, simple setae. *Mesoscutum*: Finely punctate. *Scutellum*: finely punctate and weakly transversely rugose, with scattered, small, white, scale-like setae, intermixed with a few long, erect, bristle-like setae. *Hemelytron*: Shiny, evenly punctate, with numerous small, white, scale-like setae especially on clavus and basal half of corium, intermixed with long, erect, bristle-like setae.

*Male genitalia*: Left paramere (Fig. 159) elongate, with a beak-like distal process, and two middle processes, the basal one erect, apically acute and directed outward, and middle process prostrate, lying flat against the main body. Phallotheca (Fig. 160) elongate, tapering distally to a fine point; tapered point varying from relatively short to long and nearly thread-like. Right paramere (Figs 161, 162) with one large and one small lateral arm arising from stout main stem in caudal aspect, and one short, apically rounded and one short slender process on anterior side.

*Female*: Unknown.

##### Etymology.

This species is named in honor of my good friend and colleague, Dr. Richard M. Baranowski (University of Florida, Homestead), who has done much to advance our knowledge of the Heteroptera with his many collections, especially from South Florida and the West Indies, and his numerous important publications, including *How to Know the True Bugs* (Slater and Baranowski 1978), the Coreidae of Florida (Baranowski and Slater 1986), the Lygaeidae of Florida (Slater and Baranowski 1990), and the Lygaeidae of the West Indies (Baranowski and Slater 2005).

##### Host.

Unknown.

##### Distribution.

Known only from Jamaica.

##### Type material.

Holotype ♂: **Jamaica:** ***St. Andrew Parish*:** Holywell Forest Camp, 4000 ft, Jan 1972–Jul 1972, M. Winegar, Holotype, (00286065) (USNM). Paratypes: ***Portland Parish*:** Green Hills Institute Jamaica Cabin, 23 Nov 1968, R. E. Woodruff, Paratype, 1♂ (00286061) (USNM); 17 Aug 1969, R. E. Woodruff, 2♂♂ (00286059, 00286060) (FSCA); 13 Dec 1969, E.G. Farnworth, 1♂ (00286062) (USNM). ***St. Andrew Parish*:** Holywell Forest Camp, Jan 1972 - Jul 1972, M. Winegar, Holotype, 1♂ (USNM); 19 Jul 1972, M. Winegar, 1♂ (00286064) (USNM); 04 Aug 1972, M. Winegar, 1♂ (00286063) (USNM). Holywell Forest Camp, 1219 m, Sep 1971 - Oct 1971, M. Winegar, 1♂ (00286058) (USNM); 01 Sep 1971, M. Winegar, 1♂ (00286057) (USNM); 16 Dec 1971, M. Winegar, 1♂ (00286056) (USNM).

#### Ceratocapsidea complicata

Taxon classificationAnimaliaHemipteraMiridae

(Knight) comb. n.

Figs 11–16 , 30 , 31 , 163–166

Ceratocapsus complicatus Knight, 1927: 148 (orig. descrip.), 1941: 108 (descrip., distr.); Froeschner 1949: 143 (note); Carvalho and Becker 1959: 44 (cat.); Wheeler et al. 1983: 139 (list); Henry and Wheeler 1988: 393 (cat.); Schuh 1995: 91 (cat.).

##### Diagnosis.

This species (Figs 30, 31) is distinguished by the overall yellowishbrown to orange-brown coloration with a pale embolium (costal margin) and red cuneus; two dark spots on the paler pronotal disc; the pale yellow antenna with only segment IV and the apex of III red; and the distinct male genitalia, especially the right paramere (Figs 165, 166) with two lateral arms in the caudal aspect and one in the anterior aspect and the distally bifid phallotheca (Fig. 164).

##### Description.

*Male* (n = 10; holotype measurements in parentheses): Length 3.17–3.71 mm (3.71 mm), width 1.28–1.47 mm (1.47 mm). *Head*: Width 0.69–0.75 mm (0.75 mm), interocular width 0.24–0.26 mm (0.26 mm). *Labium*: Length 0.93–1.12 mm. *Antenna*: Segment I, length 0.26–0.30 mm (0.30 mm); II, 0.72–0.98 mm (0.98 mm); III, 0.40–0.56 mm (0.56 mm); IV, 0.32–0.43 mm (0.43 mm). *Pronotum*: Length 0.58–0.69 mm (0.69 mm), basal width 1.07–1.23 mm (1.23 mm).

*Coloration*: Overall coloration yellowish to orange brown, some specimens becoming darker brown. *Head*: Yellowish brown to dark brown; eyes dark brown, often tinged with red around margins. *Antenna*: Pale yellow, segment I usually with a red streak on inner basal surface; segment II yellow, sometimes tinged with red at apex; segment III yellow, red on distal half to two thirds; segment IV red. *Pronotum*: Yellowish to orange brown, discal area with two small to large dark brown to nearly fuscous round spots just behind calli; scutellum concolorous with pronotum, often dark brown to fuscous at middle of base. *Hemelytron*: Orange brown to dark brown, costal margin (embolium), cuneus usually red or red tinged except for pale yellowishbrown outer margin and inner angle (paracuneus); membrane smoky brown, paler along basal margin bordering cuneus. *Ventral surface*: Thorax largely dark brown to fuscous, becoming paler ventrally; abdomen brown to darker reddish brown. *Ostiolar evaporative area*: Pale or whitish, often tinged with red through middle. *Legs*: Uniformly yellowish brown, hind femora usually red tinged on apical half.

*Structure, texture, and vestiture*: *Head*: Frons and vertex finely granulate, frons sometimes becoming weakly transversely rugose. *Labium*: Extending to bases on hind coxae. *Pronotum*: Finely punctate throughout except finely granulate area around calli. *Scutellum*: Finely punctate, weakly transversely rugose on basal half. *Hemelytron*: Finely and evenly punctate, except on costal margin and outer margin of cuneus. Ventral surface shiny, with only a few recumbent simple setae. Head and pronotum with numerous recumbent, simple setae, intermixed with a few longer semierect, simple setae; scutellum with three types of setae: short, recumbent and long erect, simple setae, intermixed with small, white, scale-like setae. Hemelytra with numerous short, recumbent simple setae, intermixed with long, erect and semierect simple setae, and numerous short, slender, white, scale-like setae.

*Male genitalia*: Left paramere (Fig. 163) beak-like distally, with two processes at middle, one long, reclining or prostrate and directed apically and one short, tubercle-like basally. Right paramere (Figs 165, 166) with two long, slender, curving arms in caudal aspect and a slightly shorter arm anteriorly. Phallotheca (Fig. 164) elongate and strongly bifid apically.

*Female* (n = 10): Length 3.07–3.42 mm, width 1.38–1.47 mm. *Head*: Width 0.69–0.70 mm, interocular width 0.32 mm. *Labium*: Length 1.09–1.15 mm. *Antenna*: Segment I, length 0.27–0.29 mm; II, 0.57–0.58 mm; III, 0.51–0.54 mm; IV, 0.40–0.43 mm. *Pronotum*: Length 0.59–0.62 mm, basal width 1.06–1.15 mm.

##### Hosts.

A. G. Wheeler and I have beaten this species from various trees (*Fraxinus* sp. [Oleaceae], *Ulmus* sp. [Ulmaceae]), shrubs (*Viburnum* sp. [Caprifoliaceae]), thick vegetation, and vines, especially *Vitis* spp. (grape) and *Parthenocissus quinquefolia* (L.) Plauch. (Virginia creeper) [Vitaceae]). Individuals also have been taken on *Ampelopsis arborea* (L.) Koehne [Vitaceae], *Alnus serrulata* (Aiton) Willd. [Betulaceae], *Aralia spinosa* L. [Araliaceae], *Cannabis sativa* L. [Cannabaceae], *Celtis* sp. [Ulmaceae], *Commelina virginiana* L. [Commelinaceae], *Juglans nigra* L. [Juglandaceae], and *Pluchea purpurascens* ( Sw. ) DC. [Asteraceae], most of which are probably incidental or represent sitting records. These numerous plant records indicate that *Ceratocapsidea complicata* probably is a generalist predator, searching for prey on unrelated plants.

##### Distribution.

Previously known in the United States from Florida, Illinois, Maryland, Mississippi, Missouri, North Carolina, Texas, Virginia, and West Virginia (Henry and Wheeler 1988). New state records are Arkansas, Georgia, Indiana, Louisiana, Oklahoma, Pennsylvania, South Carolina, and Tennessee.

##### Discussion.

I note that the female allotype and one female paratype from Gainesville, Florida, in Knight’s “type series” for his manuscript name “*Ceratocapsus rufistigmus*”, unintentionally validated by Blatchley (1926), actually are specimens of *Ceratocapsus complicatus* as listed below [also see my neotype desigation for *Ceratocapsidea rufistigma* Blatchley based on a male from Knight manuscript name series].

##### Type material examined.

Holotype ♂: [**USA**] **Missouri** [***Taney Co.***]: Hollister, 22 July 1915, H. H. Knight (00162214) (USNM). Paratypes: **USA:** **Florida:** ***Alachua Co.*:** Agricultural Research Station, University of Florida, Gainesville, 29.64254EN, 82.32289EW, 12 m, 09 May 1918, C. J. Drake [as *Ceratocapsidea rufistigma* Knight, ms name], 1♂ (00329557) (CNC). Gainesville, 29.63527EN, 82.37111EW, 24 m, 04 Jul 1918, C. J. Drake [as *Ceratocapsidea rufistigma* Knight, ms name], 1♂ (00246811) (TAMU). **Maryland:** ***Calvert Co.*:** Plum Point, 38.61417EN, 76.5125EW, 09 Aug 1913, W. L. McAtee, 1♂ (00329558) (CNC). **Mississippi:** ***Lowndes Co.*:** Columbus, 33.49556EN, 88.42722EW, 24 Jan 1921, Unknown, 1♀ (00071440) (USNM).

##### Other specimens examined.

**USA:** **Arkansas:** ***Hot Spring Co.*:** 5 mi. S. of Bismark, Rt. 7, 34.2675EN, 93.14583EW, 08 Jun 2004, T. J. Henry and A. G Wheeler, Jr., *Parthenocissus quinquefolia* (Vitaceae), 1♂ (00286010) (USNM). ***Perry Co.*:** Rt. 60, near Conway, Toadsuck Park, 35.07198EN, 92.52377EW, 91 m, 12 Jun 1987, T. J. Henry and A. G. Wheeler, Jr, 1♀ (00286008) *Vitis* sp. (Vitaceae), 1♂ (00286006), 1♀ (00286007) (USNM). ***Pope Co.*:** Russellville, Arkansas Tech University, 35.27842EN, 93.13379EW, 13 Jun 1987, T. J. Henry and A. G Wheeler, Jr., *Fraxinus* sp. (Oleaceae), 1♂ (00286009) (USNM). **Florida:** ***Alachua Co.*:** Newnan’s Lake, Gainesville, 29.65073EN, 82.24006EW, 26 Apr 1952, O. Peck, 1♂ (00167380) (AMNH). ***Liberty Co.*:** 1.5 to 3 mi S of Bristol, County Road 379, 30.40532EN, 84.96591EW, 44 m, 07 May 1982, T. J. Henry, *Pluchea purpurascens* (Asteraceae), 1♂ (00286004) (USNM). Torreya State Park, 30.56889EN, 84.94778EW, 28 Apr 1952, O. Peck, 1♂ (00167378) (AMNH). University of Florida Agricultural Experimental Station, 29.72108EN, 82.41816EW, 53 m, 31 May 1918, C. J. Drake, 1♀ (00286266) (USNM); 04 Aug 1918, C. J. Drake, 1♀ (00286267) (USNM). **Georgia:** ***Peach Co.*:** Peach County, 32.5666EN, 83.8333EW, 13 Sep 1943, Turner, light trap, 1♂ (00071450) (USNM). **Illinois:** ***Pope Co.*:** Herod, 37.58028EN, 88.43611EW, 24 Jul 1930, Knight and Rose, 1♀ (00071441) (USNM). **Indiana:** ***Elkhart Co.*:** Elkhart, 41.68194EN, 85.97666EW, 229 m, 05 Aug 1896, R.J. Weith, 1♂ (00071453) (USNM). **Louisiana:** ***Madison Parish*:** Tallulah, 32.4083EN, 91.1867EW, 16 Jun 1924, E. R. Kalmbach, 1♂ (00286011) (USNM). ***Rapides Parish*:** Alexandria, 31.3125EN, 92.44555EW, 14 m, 08 Jun 1943, Wm. F. Buren, 1♂ (00286003) (USNM); 16 Jul 1943, Wm. Buren, 1♂ (00071442) (USNM). **Maryland:** ***Anne Arundel Co.*:** Odenton, 39.08389EN, 76.70056EW, 29 Jul 1917, W. L. McAtee, *Vitis* sp. (Vitaceae), 1♀(00071449) (USNM). ***Baltimore Co.*:** Butler, 39.03444EN, 76.7275EW, 108 m, 19 Sep 1986–23 Sep 1986, E. J. Ford, light trap, 1♂ (00071454) (USNM). ***Calvert Co.*:** Plum Point, 38.61417EN, 76.5125EW, 09 Aug 1913, W. L. McAtee, 1♂ (00071447) (USNM). ***Prince George’s Co.*:** Beltsville, 39.03472EN, 76.90778EW, 14 Aug 1914, W. L. McAtee, 1♂ (00071446) (USNM). **Mississippi:** ***Issaquena Co.*:** Rolling Fork, 32.90555EN, 90.87972EW, 30 m, 23 Jul 1929, H. G. Johnston, 1♀ (00071444), 1♂ (00071445) (USNM). ***Lafayette Co.*:** University of Mississippi Campus, 34.36667EN, 89.53528EW, 30 Jun 1983, P. K. Lago, *Cannabis sativa* (Cannabaceae), 1♂ (00285999) (USNM). ***Noxubee Co.*:** 33.10472EN, 88.55811EW, 52 m, 06 Aug 1972, M.F. Bonuster, 1♀ (00286002) (USNM). ***Oktibbeha Co.*:** Missississippi State University [A. & M. College], Starkville, 33.4504EN, 88.81839EW, 91 m, 22 Sep 1894, H. E. Weed, 1♂ (00286000) (USNM). ***Sharkey Co.*:** Cary, 32.80583EN, 90.92666EW, 29 m, 27 Jul 1929, H. G. Johnston, 1♂ (00246807) (TAMU), 1♂ (00071443) (USNM). Rolling Fork, 32.90631EN, 90.87811EW, 29 m, 23 Jul 1929, H. G. Johnston, 1♂ (00246806) (TAMU). ***Washington Co.*:** Stoneville, 33.42401EN, 90.9151EW, 18 Aug 1983, G. L. Snodgrass, *Vitis cinerea* (Vitaceae), 1♂ (00285993) (USNM); 08 Sep 1983, G. L. Snodgrass, *Commelina virginica* L. (Commelinaceae), 1♀ (00285992) (USNM); 24 Sep 1983, G. L. Snodgrass, *Ampelopsis arborea* (Vitaceae), 1♀ (00285991), 1♂ (00285994) (USNM). **Missouri:** ***St. Charles Co.*:** Peruque, 38.86833EN, 90.60806EW, 21 Jul 1919, W. L. McAtee, 2♂♂ (00285995, 00285996) (USNM). **North Carolina:** ***Edgecombe Co.*:** 10 km NNE Tarboro, 35.97988EN, 77.49356EW, 08 Jul 1983, W. Steiner, A. Gerberich, A. & J. Clarke, 1 (?sex) (00286001) (USNM). ***Macon Co.*:** Wayah Gap, 35.15516EN, 83.58086EW, 1280 m, 29 Jul 1957, J. G. Chillcott, 1♀ (00167376), 1♂ (00167377) (AMNH). ***Wayne Co.*:** Mill Creek, 35.44656EN, 78.02973EW, 25 m, 14 Aug 1957, L. A. Kelton, 1♀ (00167379) (AMNH). **Oklahoma:** ***Carter Co.*:** Rt. 53 & I-35, near Springer, 34.30388EN, 97.1625EW, 200 m, 13 Jun 1999, T. J. Henry and A. G. Wheeler, Jr., *Juglans* sp. (Juglandaceae), 1♂ (00071459) (USNM). **Pennsylvania:** ***Indiana Co.*:** 4mi W of Strongstown, 40.51682EN, 78.86219EW, 575 m, 14 Aug 1974, A. G. Wheeler, *Aralia spinosa* (Araliaceae), 1♂ (00246809) (TAMU). ***Monroe Co.*:** Sciota, Rt. 33, along Belmont Pike, 40.92722EN, 75.30111EW, 19 Jul 1998, T. J. Henry, *Vitis* sp. (Vitaceae), 1♂ (00286005) (USNM). ***York Co.*:** Manchester, Dauber’s Nursery, 40.06306EN, 76.71861EW, 16 Aug 1974, A. G. Wheeler, *Viburnum* sp. (Caprifoliaceae), 1♂ (00246810) (TAMU). Manchester, Dauber’s Nursery, 40.06388EN, 76.72041EW, 146 m, 01 Aug 1974, T. J. Henry and A. G. Wheeler, Jr., *Viburnum* sp. (Caprifoliaceae), 1♀ (00071455) (USNM); 16 Aug 1974, T. J. Henry and A. G. Wheeler, Jr, *Viburnum* sp. (Caprifoliaceae), 1♀ (00071456), 2♂♂ (00071457, 00071458) (USNM). **South Carolina:** ***Pickens Co.*:** Clemson University, 34.68333EN, 82.8375EW, 14 Jul 1988, T. J. Henry and A. G Wheeler, Jr., 1♀ (00286012), *Alnus serrulata* (Betulaceae), 1♂ (00285998) (USNM). **Tennessee:** ***Hamilton Co.*:** Hamilton, 35.04305EN, 85.36611EW, 231 m, 22 Sep 1943, Turner, light trap, 1♂ (00071451) (USNM). ***Lawrence Co.*:** Lawrence County, 35.22861EN, 87.40472EW, 889 m, 12 Jul 1940, collector unknown, *Prunus* sp. (Rosaceae), 1♂ (00071452) (USNM). **Texas:** ***Brazos Co.*:** College Station, 30.62778EN, 96.33417EW, 04 Jul 1932, H. J. Reinhard, 1♀ (00246808) (TAMU). ***Comal Co.*:** New Braunfels, 29.7028EN, 98.1242EW, 194 m, 22 Jun 1917, H. H. Knight, 1♂ (00246812) (TAMU). ***Lee Co.*:** Rt. 21, West Yegua Creek, at Lincoln, 30.28333EN, 96.95EW, 10 May 2002, T. J. Henry and P. S. F. Ferreira, *Ulmus* sp. (Ulmaceae), 3♂♂ (00071460-00071462) (USNM). ***Llano Co.*:** Ink Lake, Rt. 29 & Jct. to Dam Rd., 30.75EN, 98.46666EW, 312 m, 12 Jun 1997, T. J. Henry & J. C. Schaffner, *Celtis* sp. (Ulmaceae), 3♂♂ (00071463-00071465), 2♀♀ (00071466, 00071467) (USNM). **Virginia:** ***Fairfax Co.*:** Scotts Run to Balts Hill, 38.9375EN, 77.20444EW, 77 m, 12 Aug 1917, W. L. McAtee, 1♂ (00071448) (USNM). ***Loudoun Co.*:** Murrays Ford Farm, Leesburg, 39.11556EN, 77.56389EW, 14 Aug 1997, T. J. Henry & V. N. Power, 1♂ (00285997) *Vitis* sp. (Vitaceae), 1♀ (00285990) (USNM).

#### Ceratocapsidea consimilis

Taxon classificationAnimaliaHemipteraMiridae

(Reuter) comb. n.

Figs 32 , 167–170

Ceratocapsus consimilis Reuter, 1907: 14 (orig. descrip.); Van Duzee 1907: 29 (note); Carvalho 1958: 44 (cat.), 1990: 193 (descrip. type); Schuh 1995: 91 (cat.)

##### Diagnosis.

This species (Fig. 32) is best recognized by the dark brown head and pronotum, paler brown hemelytra with numerous, evenly spaced, brown-stained punctures; the pale antennae, with only segment IV tinged with red; pale yellow legs, with the hind femur reddish brown on the distal two thirds; and the male genitalia, especially the right paramere (Figs 169, 170), with two long, curving arms, the shorter one distally bifid, and the phallotheca (Fig. 168) with a beak-like apex.

##### Description.

*Male* (n = 1): Length 3.65 mm, width 1.47 mm. *Head*: 0.70 mm, interocular width 0.21 mm. *Labium*: Length 1.04 mm. *Antenna*: Segment I, length 0.24 mm; II, 0.67 mm; III, 0.45 mm; IV, 0.38 mm. *Pronotum*: Length 0.53 mm, basal width 0.99 mm.

*Coloration*: General color brown to dark brown. *Head*: Dark brown, eyes reddish. *Antenna*: Segments I–III yellowish brown, segment IV red. *Pronotum*: Dark brown, becoming paler brown on posterior third; scutellum dark brown, apex pale yellowish brown. *Hemelytron*: Overall brown to yellowish brown, slightly darker on basal half of clavus, tinged with red along subcostal vein; membrane smoky brown, paler along base. *Ventral surface*: Thoracic segment dark brown, abdomen brown. *Ostiolar evaporative area*: Pale or whitish, central knob brown. *Legs*: Overall yellowish brown, hind femur dark reddish brown on distal half to two thirds.

*Structure, texture, and vestiture*: *Head*: Finely granulate on vertex, transversely rugose on frons, with a few recumbent and semierect, pale simple setae. *Labium*: Extending to bases of hind coxae nearly to base of abdomen. *Pronotum*: Semishiny (not polished) with evenly distributed brown-stained punctures, except for finely granulate area anteriorly around calli; with numerous small, white, scale-like setae, intermixed with longer, semierect, pale brown, simple setae; scutellum evenly punctate, with numerous small, white, scale-like setae, intermixed with long, erect, pale, simple setae. *Hemelytron*: Evenly distributed with fine brown-stained punctures, with numerous scattered short, recumbent and long erect and semierect simple setae, intermixed with small, white, scale-like setae.

*Male genitalia*: Left paramere (Fig. 167) apically beak-like, with a trifid middle process on dorsal margin, one process prostrate and two recurved forming a C-shape. Right paramere (Figs 169–170) with two lateral arms in caudal view, the upper one shortest, bifid, and the lower one longer and flared apically; each arm crenulate distally along margins. Phallotheca (Fig. 168) uniformly broad, with stout beak-like apex bent down and finely serrate around margins.

*Holotype female*: Length 2.80 mm, width 1.20 mm. *Head*: Width 0.62 mm, interocular width 0.28 mm. *Labium*: Missing. *Antenna*: Segment I, length 0.24 mm; II, 0.72 mm; III and IV missing. *Pronotum*: Length 0.50 mm, basal width 0.96 mm.

##### Host.

Unknown.

##### Distribution.

Described and previously known only from the female holotype from Jamaica.

##### Discussion.

The holotype female deposited at the California Academy of Sciences was examined and have matched to a male (CNC) that compares closely with it. This is important because of the great amount of speciation that has occurred in Jamaica. Most of the Jamaican species treated in this paper can be distinguished with certainty only by the male genitalia. Fortunately, *Ceratocapsidea consimilis* is one of the more distinct species externally, with a paler brown dorsum having dark-stained punctures and two types of pubescence on the both the pronotum and hemelytra. Carvalho (1990) illustrated the feHolotype male and commented that this species has the appearance of a small *Ceratocapus modestus* Uhler [known from eastern United States], which is misleading and inaccurate. The dorsum of the eastern North American *Ceratocapsus modestus* is impunctate and nearly glabrous, whereas *Ceratocapsidea consimilis* is strongly and evenly punctate, with two types of pubscence on pronotum and hemelytra, and the male genitalia differ substantially (see Knight 1941 or Henry 1979 for Figures of *Ceratocapsus modestus* genitalia).

The Nicaraguan record of *Ceratocapsidea consimilis* reported by Cherot et al. (2007) undoubtedly is a misidentification of another ceratocapsine. Because their specimen is a female, it will be difficult to determine the species with certainty.

##### Type material examined.

Holotype ♀: **JAMAICA:** Mande[vil]le, Apr. [19]06, Van Duzee collector [“Spec. typ.”; “Holotype”; “E. P. Van Duzee Collection”] (CAS).

##### Other specimen examined.

**JAMAICA:** 1♂, St. James, Adelphi, 15 Aug. 1966, H. Howden (CNC).

#### Ceratocapsidea cubana

Taxon classificationAnimaliaHemipteraMiridae

(Bergroth) comb. n.

Figs 33 , 171–174

Ceratocapsus punctulatus Reuter, 1876: 87. Preoccupied by *Trichia punctulata* Reuter, 1876; neotype designated by Hernández and Henry 1999: 209.
Ceratocapsus cubanus Bergroth, 1910: 68 (n. name); Carvalho 1958: 45 (cat.); Schuh 1995: 91 (cat.); Hernández and Henry 1999 (descrip.); Hernández and Henry 2010: 106 (diag., color habitus). New name for preoccupied junior secondary homonym *Ceratocapsus punctulatus* Reuter, 1876.

##### Diagnosis.

This species (Fig. 33) is distinguished by the overall dark brown dorsum with only the clavus and basal half of the corium paler yellowish brown, the yellowishbrown antennal segments I and II, and male genitalia, particularly the right paramere (Figs 173, 174) with three lateral arms visible from the caudal aspect, two of which are short and marginally serrate and one long, with the apex slender, apically acute, and bent 90E.

##### Description.

*Lectotype male*: Length 3.32 mm, width 1.18 mm. *Head*: Width 0.80 mm, interocular width 0.24 mm. *Labium*: Embedded in glue. *Antenna*: Segment I, length 0.27 mm; II, 0.80 mm; III, 0.45 mm; IV, 0.43 mm. *Pronotum*: Length 0.69 mm, basal width 1.14 mm.

*Coloration*: Overall coloration dark brown, with paler areas on hemelytra. *Head*: Dark brown. *Antenna*: Pale yellowish brown, segment III and IV reddish. *Pronotum*: Dark brown. *Scutellum*: Dark brown basally, pale yellowish brown on apical third. *Hemelyron*: Clavus and basal half of corium pale yellowish brown, cuneus and apical half of corium dark brown, cuneus weakly red tinged; membrane smoky brown on distal half, paler basally. *Ventral surface*: Thoracic area brown to dark brown, abdomen dark brown. *Ostiolar evaporative area*: Pale or white. *Legs*: Overall yellowish brown to brown, hind femur and tibia dark brown.

*Structure, texture, and vestiture*: *Head*: Granulate. *Pronotum*: Shiny, evenly and finely punctate, except finely granulate around calli. *Labium*: Extending to about hind coxae. *Scutellum*: Finely punctate. *Hemelytra*: Shiny, finely and evenly punctate. Head and pronotum with short, recument, simple setae. Scutellum and hemelytra with numerous, short, recumbent, simple setae and slender, white scale-like setae, intermixed with long, erect, simple setae.

*Male genitalia*: Left paramere (Fig. 171): Slender, with beak-like apex somewhat blunt at tip, and middle processes prostrate against paramere body, forming a shallow C-shaped structure. Right paramere (Figs 173, 174) with three arms visible from caudal aspect, the two shorts arms marginally crenulate, the longer arm bent at a right angle distally. Phallotheca (Fig. 172): Relatively stout, with a peg-like apex.

*Female* (paralectotype): Length to apex of membrane 2.80 mm, length to base of cuneus 2.00 mm, width 1.08 mm. *Head*: Width 0.69 mm, interocular width 0.77 mm. *Labium*: Embedded in glue. *Antenna*: Segment I, length 0.27 mm; II, 0.75 mm; III, 0.37; IV, missing. Pronotum: Length 0. 62 mm, basal width 1.01 mm.

##### Host.

Unknown.

##### Distribution.

Described and known only from Cuba.

##### Discussion.

Hernández and Henry (1999) indicated that they had “searched the Helsinki collection for specimens of *Ceratocapsus punctulatus* without success, even though the type of *Trichia punctulatus* is present there (pers. comm., the late Antti Jansson)”. As a consequence, they designated a neotype from “Pinares de Viñales, Cuba”, which is deposited in the Instituto de Ecología y Sistemática, Ciudad Habana, Cuba (IES).

Since their work (Hernández and Henry 1999), I have visited the Helsinki and Stockholm (2006) museums. I was surprised to find that Stockholm has many of Reuter’s syntypes sent by Reuter to Stål (Bert Viklund, pers. comm.). Although Helsinki does not have original material of *Ceratocapsus punctulatus*, a male and female syntype were found in Stockholm that clearly are part of the original series Reuter had before him when describing this species (Reuter did not indicate a number of specimens). These specimens, which agree with Hernández and Henry’s (1999) interpretation of the species, now should become the name-bearing types, according to Article 75.8 of the ICZN (2000), which states that “if, after the designation of a neotype, the name-bearing types of the nominal species-group taxon that were presumed lost are found still to exist, on publication of that discovery the rediscovered material again becomes the name-bearing type and the neotype is set aside”. Hernández and Henry (2010) followed their 1999 paper, deferring correction of the neotype/lectotype designation to the present work.

##### Type designation.

The following male is designated as the lectotype of *Ceratocapsus punctulatus*: Lectotype ♂: label 1, “Cuba”; 2, “Stål”; 3, red label “Typus”; 4, (white, folded, handwritten label) “Ceratocapsus punctulatus Reut. typ.”; 5 (printed red label here added) “Lectotype ♂ *Ceratocapsus punctulatus* Reuter, desig. by T. J. Henry (SMNH).” The second specimen, a ♀, with the same label data as for the lectotype, is labeled as a paralectotype (SMNH).

#### Ceratocapsidea dominicanensis

Taxon classificationAnimaliaHemipteraMiridae

Henry sp. n.

http://zoobank.org/D9DB545D-1F67-4BA0-9247-1AA03D30DDCC

Figs 34 , 35 , 175–177

##### Diagnosis.

This species (Figs 34, 35) is distinguished by the overall brown coloration, with the dark brown pronotum having a pale brown median line (pale line sometimes flaring wider on the anterior region around calli and area near posterior margin); the numerous, relatively long, simple setae on the dorsum, antennae, and legs; and the unique male genitalia, especially the left paramere (Fig. 175) with a beak-like apex and two large, stout processes at middle, the strongly pubescent right paramere (Fig. 177) with two lateral arms, and the phallotheca (Fig. 176) with a broad, serrate and apically quadrate apex.

##### Description.

*Male* (n = 3; holotype measurements in parentheses): Length 3.49–3.65 mm (3.65 mm), width 1.72–1.86 mm (1.86 mm). *Head*: Width 1.00–1.08 mm (1.08 mm), interocular width 0.30 mm (0.30 mm). *Labium*: 1.46–1.52 mm (1.50 mm). *Antenna*: Segment I, length 0.34–0.36 mm (0.36 mm); II, 1.04–1.10 mm (1.04 mm); III, 0.60–0.64 mm (0.60 mm); IV 0.50 mm (0.50 mm). *Pronotum*: Length 0.84–0.88 mm (0.88 mm), basal width 1.50–1.62 mm (1.62 mm).

*Coloration*: Overall brown with darker areas. *Head*: Brown to yellowish brown, with a few scattered brown-stained punctures; eyes dark brown. *Antenna*: Segments I and II yellowish brown, segment I with a short red streak at base on inner surface; segments III and IV becoming slightly dark brown. *Pronotum*: Dark brown, with median line pale yellowish brown, sometimes with posterior flared area around calli yellowish brown. *Scutellum*: Brown with a few scattered brown-stained punctures. *Hemelytron*: Brown, with costal area and outer margin of cuneus paler yellowish brown; evenly scattered with dark brown punctures; membrane uniformly smoky brown, veins darker brown. *Ventral surface*: Thorax and abdomen dark brown, becoming slightly paler ventrally. *Ostiolar evaporative area*: Evenly pale or whitish. *Legs*: Uniformly brown, hind femur darker brown on apical third, except for extreme apex.

*Structure, texture, and vestiture*: *Head*: Surface relatively smooth, with a few scattered, fine punctures. *Labium*: Extending to bases of middle coxae or slightly beyond. *Pronotum*: Shiny, evenly scattered with distinct, strong punctures. *Scutellum*: Equilateral, weakly transversely rugose, finely punctate. *Hemelytron*: Shiny, with evenly scattered punctures; punctures largely absent along costal area and apical area of cuneus. *Ventral surface*: Shiny, impunctate. Head, pronotum, and hemelytron with numerous short, decumbent simple setae and small, slender, white scale-like setae, intermixed with long, erect, simple setae. Antenna set with numerous short, recumbent setae, intermixed with long, erect setae.

*Male genitalia*: Aperture of male genital capsule with a long, slender tubercle at about 2:00 o’clock. Left paramere (Fig. 175) beak-like apically, with a distally truncate apical process and a small subapical spine, and with two stout, heavily setose, median processes, one much longer than other. Right paramere (Fig. 177) with two slender lateral arms, one recurved at apex, and apical half of main trunk with long, extremely dense setae. Phallotheca (Fig. 176) stout, with curved apex quadrate and distally serrate.

*Female* (n = 5): Length 3.33–3.58 mm, width 1.98–2.00 mm. *Head*: Width 0.94–0.98 mm, interocular width 0.36–0.40. *Labium*: Length 1.48–1.50 mm. *Antenna*: Segment I, length 0.34–0.36 mm; II, 0.94–1.00 mm; III, 0.54–0.56 mm; IV, 0.40–0.44 mm. *Pronotum*: Length 0.82–0.84 mm, basal width 1.52–1.60 mm.

Similar to male in overall coloration, pubescence, and markings. [pubescence?]

##### Etymology.

This species is named *dominicanensis*, after the Dominican Republic where all specimens were collected.

##### Host.

Unknown.

##### Distribution.

Known only from the Dominican Republic.

##### Type material.

Holotype ♂: **DOMINICAN REPUBLIC:** **Santo Domingo:** Santo Domingo City, 18.49397EN, 69.9536EW, 43 m, 22 Jul 1917, H. Morrison (00286070) (USNM).

Paratypes. **DOMINICAN REPUBLIC:** **San Cristobal:** 5 mi N of Haina, 18.47898EN, 70.02059EW, 27 m, 14 Aug 1967, J. C. Schaffner, 1♂ (00286069) (USNM). San Cristobal, 18.41666EN, 70.1EW, 02 Aug 1967, J. C. Schaffner, 1♀ (00286073) (TAMU); 05 Aug 1967, J. C. Schaffner, 2♀♀ (00286071, 00286072), 1♂ (00286075) (USNM). 18.4789EN, 70.16182EW, 26 Jul 1917, H. Morrison, 1♀ (00286067) (USNM); 19 Nov 1967, collector unknown, 1♀ (00286074) (TAMU). **San Pedro de Macoris:** seashore, 18.42679EN, 69.40358EW, 6 m, Jun 1971, J. Maldonado C., 1♀ (00286068) (USNM).

#### Ceratocapsidea fusiformis

Taxon classificationAnimaliaHemipteraMiridae

(Van Duzee) comb. n.

Figs 17 , 18–23 , 36 , 37 , 178–181

Ceratocapsus fusiformis Van Duzee, 1917a: 270 (orig. descrip.); Carvalho 1958: 46 (cat.); Knight 1968: 156 (distr., host); Henry and Wheeler 1988: 394 (cat.); Schuh 1995: 93 (cat.).
Ceratocapsus clavicornis Knight, 1925: 47 (orig. descrip.); Carvalho 1958: 44 (cat.); Henry and Wheeler 1988: 393 (cat.); Schuh 1995: 91 (cat.). **syn. n.**
Ceratocapsus divaricatus Knight, 1927: 145 (orig. descrip.); Carvalho 1958: 45 (cat.); Henry and Wheeler 1988: 393 (cat.); Schuh 1995: 92 (cat.); Henry et al. 2005: 52 (list). **syn. n.**

##### Diagnosis.

*Ceratocapsus fusiformis* (Figs 36, 37) can be distinguished by the striate frons, the brown pronotum often having the base and calli darker brown, the dark or reddish hind femur, silvery scale-like and bristle-like setae on the hemelytron, and the male genitalia, especially the apically bifid phallotheca (Fig. 179) and distally bifid arm on the right paramere (Figs 18, 181).

##### Description.

*Male* (n = 10; holotype measurements of *Ceratocapsus clavicornis*, *Ceratocapsus divaricatus*, and *Ceratocapsus fusiformis* in this sequence in parentheses): Length 2.94–3.39 mm (3.30 mm, 3.42 mm, 3.68 mm), width 1.14–1.38 mm (1.30 mm, 1.26 mm, 1.44 mm). *Head*: Width 0.69–0.82 mm (0.78 mm), interocular width 0.26–0.27 mm (0.32 mm, 0.26 mm, 0.30 mm). *Labium*: Length 1.06–1.15 mm (1.17 mm, 1.12 mm, ca 1.36 mm [apex in glue]). *Antenna*: Segment I, length 0.21–0.26 mm (0.24 mm, 0.26 mm, 0.28 mm); II, 0.64–0.75 mm (0.72 mm, 0.72 mm, 0.88 mm); III, 0.32–0.43 mm (0.38 mm, 0.42 mm, 0.44 mm); IV, 0.32–0.40 mm (0.40 mm, missing, 0.40 mm). *Pronotum*: Length 0.59–0.70 mm (0.70 mm, 0.67 mm, 0.76 mm), basal width 1.07–1.22 mm (1.22 mm, 1.20 mm, 1.28 mm).

*Coloration*: Overall coloration brown with dark brown areas. *Head*: Dark brown, eyes dark brown tinged with red. *Antenna*: Segment I pale yellow, with a red dash on inner basal surface; segment III pale yellow, apex reddish brown; segments III and IV red to reddish brown. *Pronotum*: Brown, often with posterior margin and calli dark brown to fuscous. *Scutellum*: Brown, median line usually dark brown. *Hemelytron*: Yellowish brown, apical two thirds of corium and apical third of clavus darker brown; cuneus and often apical third of embolium (costal margin) red to dark reddish brown; membrane dark translucent brown, except around base bordering cuneus. *Ventral surface*: Brownish red, becoming darker brown to fuscous on abdomen. *Ostiolar evaporative area*: Uniformly pale or white. *Legs*: Forelegs uniformly pale yellow; middle legs pale yellow, apical third of femur and basal fourth of tibia brown; hind femur with only distal half dark brown to almost entirely dark brown to reddish brown, except for base and apex; hind tibia brown to reddish brown, sometime pale on distal half; all tarsi and claws pale yellow.

*Structure, texture, and vestiture*: *Head*: Finely granulate and transversely with relatively short, semierect, simple setae. Labium: Extending to bases of hind coxae. *Pronotum*: Shiny, with fine brown-stained punctures, calli finely granulate, with short, semierect, simple setae, intermixed with a few long, semierect, simple setae and more thickly scattered white or silvery, scale-like setae. *Scutellum*: Finely punctate and weakly transversely rugose. *Hemelytron*: Shiny, with evenly scattered fine, brown-stained punctures; thickly clothed with silvery, scale-like setae, intermixed with long, erect, almost bristle-like brown setae.

*Male genitalia*: Left paramere (Fig. 178) almost S-shaped, apex beak-like, middle process relatively long and rectangular, with a slender spine at base. Right paramere (Figs 180, 181) with one broad, stout, apically bifid arm, with the apex of the dorsal process Y-shaped and the lower process hooked. Phallotheca (Fig. 179)] slender, with, apex bifid, appearing beak-like with upper side of beak about 2 x the length of lower side.

*Female* (n = 10): Length 2.78–3.17 mm, width 1.22–1.34 mm. *Head*: 0.67–0.74 mm, interocular width 0.30–0.34 mm. *Labium*: Length 1.06–1.12 mm. *Antenna*: Segment I, length 0.21–0.24 mm; II, 0.67–0.75 mm; III, 0.35–0.42 mm; IV, 0.32–0.40 mm. *Pronotum*: Length 054–062 mm; basal width 1.01–1.17 mm. Color and vestiture similar to that of male.

##### Hosts.

Knight (1968) reported taking specimens of *Ceratocapsus fusiformis* on *Purshia tridentata* Pursh (DC) [Rosaceae], but most other records for this species are from species of *Quercus* [Fagaceae]. In the eastern United States, it is most frequently taken on *Quercus stellata* Wangenh. In the West, it has been taken on *Quercus arizonica* Sarg., *Quercus emoryi* Torr., *Quercus gambeli* Nutt., *Quercus oblongifolia* Torr., and *Quercus turbinella* Greene. It also has been taken on *Platanus wrightii* S. Watson [Platanaceae], *Purshia glandulosa* Curran, *Purshia stansburiana* (Torr.) Henrickson [Rosaceae], *Symphoricarpos oreophilus* A. Gray [Caprifoliaceae], and *Vauquelinia californica* [Rosaceae].

##### Distribution.

This species was described from California and Colorado (Van Duzee 1917a) and later reported from Nevada (Knight 1968). It is also recorded from Arizona as *Ceratocapsus clavicornis* (Knight, 1925) and Florida and Kentucky as *Ceratocapsus divaricatus* (Knight 1927, Henry et al. 2005). Downes (1935) referred his earlier record of *Ceratocapsus fusiformis* from British Columbia (Downes 1924) to *Ceratocapsus downesi* Knight. New state records are Arkansas, Georgia, North Carolina, Oklahoma, Oregon, South Carolina, Tennessee, Texas, and Utah.

##### Discussion.

Comparison of the male genitalia and external morphology of a paratype from Colorado and the holotypes of *Ceratocapsus divaricatus* Knight and *Ceratocapsus clavicornis* Knight indicates that these two species are clearly conspecific with *Ceratocapsus fusiformis*. The shape of the lower arm of the right paramere varies slightly over the entire range, but otherwise these bugs are indistinguishable.

*Ceratocapsidea fusiformis* has the widest distribution of any North American ceratocapsine. Most species are restricted to particular regions, such as eastern U.S., western U.S., or southwestern U.S., whereas *Ceratocapsus fusiformis* ranges from Oregon and Utah, south to California and Arizona through Colorado and Nevada, and east to Florida and North Carolina.

##### Type material examined.

Holotype ♂ (*Ceratocapsus clavicornis* Knigtht): [**USA**] **Arizona:** Grand Canyon, Grand V., 3 Aug. 1917, H. H. Knight (0016210) (USNM); Holotype ♂ (*Ceratocapsus divaricatus* Knight), Florida [Seminole Co.], Sanford, 15 May 1926, E. D. Ball (00162211) (USNM). Paratypes: **USA:** **Arizona:** ***Coconino Co.*:** Grand Canyon [National Park], Grand V[iew], 35.99877EN, 111.98952EW, 2248 m, 03 Aug 1917, H. H. Knight, 10♂♂ (00286200-00286209), 13♀ (00286222) (USNM). ***Pima Co.*:** Tucson, 32.22167EN, 110.92582EW, 1372 m, 12 May 1929, E. D. Ball, 2♀♀ (00286223, 00286224), 1♂ (00286225) (USNM). **Colorado:** ***El Paso Co.*:** Colorado Springs, 08 Jul 1902, E. P. Van Duzee, 1♀ (00286238), 1♂ (00286239) (USNM).

##### Other specimens examined.

**USA:** **Arizona:** ***Cochise Co.*:** 4 mi W of Portal, 31.91361EN, 109.20895EW, 1700 m, 10 Jun 1980, R.T. Schuh, K & R. Schmidt and B. Massie, 2♂♂ (00138492, 00138493) (AMNH). Ash Canyon Road, 0.5 mi W of Hwy 92, 31.38194EN, 110.22444EW, 1554 m, 26 Jun 1990–29 Jun 1990, N. McFarland, light trap, 1♂ (00286046) (USNM). Ash Canyon Road, nr. Rt. 92, S. Sierra Vista, 31.55454EN, 110.30369EW, 1646 m, 04 Jun 1997, T. J. Henry and A. G Wheeler, Jr., *Quercus oblongifolia* Torr. (Fagaceae), 2♀♀ (00286104, 00286105) (USNM). Cave Creek Canyon, Chiricahua Mountains, Sunny Flat Campground, 31.88333EN, 109.16666EW, 1554 m, 31 May 1982, B. Barientos, 2♀♀ (00138464, 00138465) (AMNH). Hereford, Miller Canyon Rd. at Rt. 92, 31.42929EN, 110.2369EW, 1463 m, 02 Jun 1997, T. J. Henry and A. G. Wheeler, Jr, *Platanus wrightii* (Platanaceae), 2♂♂ (00286143, 00286144) (USNM). Huachuca Mountains, 31.502EN, 110.3994EW, 1839 m, 08 Jul 1905, Unknown, 1♀ (00286141) (USNM); 10 Jul 1905, Unknown, 1♂ (00286140) (USNM); 31 Jul 1905, collector unknown, 1♂ (00286142) (USNM); 03 Aug 1905, collector unknown, 1♀ (00286246), 1♂ (00286247) (USNM). Huachuca Mountains, 5354 Ash Canyon Road, 0.5 mi W of Hwy 92, 31.38194EN, 110.22444EW, 1554 m, 29 Jun 1992, N. McFarland, light trap, 1♂ (00286042) (USNM); 20 Jul 1992, N. McFarland, light trap, 1♂ (00286043) (USNM); 27 Aug 1992, N. McFarland, 1♂ (00286109) (USNM); 19 Sep 1992, N. McFarland, 1♂ (00286145) (USNM); 13 Oct 1992, N. McFarland, 1♂ (00286148) (USNM); 29 May 1993–24 Sep 1993, N. McFarland, light trap, 23♂♂ (00286017- 00286039), 2♀♀ (00286040, 00286041) (USNM); 11 Jun 1993, N. McFarland, 1♂ (00286146) (USNM); 15 Jun 1993, N. McFarland, 1♂ (00286147) (USNM); 24 Sep 1993, N. McFarland, light trap, 1♂ (00286044) (USNM). Huachuca Mountains, Ash Canyon, 31.55454EN, 110.30369EW, 1646 m, 02 Jun 1997, T. J. Henry and A. G. Wheeler, Jr., 1♂ (00286112), 1♂ (00286113) (USNM). Huachuca Mountains, Ash Canyon Rd., 31.3822EN, 110.23143EW, 1554 m, 02 Jun 1997, T. J. Henry & A. G. Wheeler, Jr., 1♂ (00286120), 2♂♂ (00286121, 00286164) (USNM); 03 Jun 1997, T. J. Henry and A. G. Wheeler, Jr., 1♀ (00286106), 7♂♂ (00286114- 00286119, 00286163) (USNM). Huachuca Mountains, Ash Canyon Road, Jct. 92, 31.38938EN, 110.23033EW, 1539 m, 05 Jun 1997, T. J. Henry and A. G Wheeler, Jr., *Quercus oblongifolia* Torr. (Fagaceae), 2♂♂ (00286110, 00286111) (USNM). Huachuca Mountains, Carr Canyon, 2.5 mi. NW of Hwy. 92, Snapp residence, 31.44091EN, 110.28593EW, 1669 m, 13 Jun 1993, N. McFarland, 2♂♂ (00286107, 00286108) (USNM). Pinery Canyon, Chiricahua Mountains, 31.93514EN, 109.27482EW, 2073 m, 17 Jun 1932, D. K. Duncan, 2♂♂ (00286122, 00286123), 1♀ (00286134) (USNM). Texas Pass, 32.06048EN, 110.0791EW, 1524 m, 20 Jul 1917, H. H. Knight, 1♂ (00286231) (USNM). West Turkey Creek (Chiricahua Mts), 31.9986EN, 109.8177EW, 16 Jul 1976, Scott McCleve, 1♀ (00138522) (AMNH). ***Coconino Co.*:** 3.5 mi S of Sedona on Rt 179, T17N R6E S30, 34.8255EN, 111.769EW, 1280 m, 15 Jun 1983, R.T. Schuh and M.D. Schwartz, *Quercus turbinella* Greene (Fagaceae), 8♂♂ (00138513, 0138514, 00138512, 00138511, 00138510, 00138509, 00138508, 00138507), 22♀♀ (00138562) *Quercus* sp. (Fagaceae), 2♀♀ (00138536, 00138537) *Cowania stansburiana* (Rosaceae), 1♂ (00138505) (AMNH). Bright Angel [Trail, Grand Canyon National Park], 36.05748EN, 112.14416EW, 2073 m, 10 Jul 1922, Barber and Schwarz, 1♀ (00286124), 1♂ (00286125) (USNM). Grand Canyon, 36.05444EN, 112.13861EW, 2134 m, 11 Jul 1892, collector unknown, 1♂ (00286136) (USNM). Hermit Rim Road, Grand Canyon National Park, 36.06034EN, 112.21308EW, 2012 m, 01 Aug 1914, J. Ch. Bradley, 1♀ (00286236) (USNM). Long Valley, 34.52123EN, 111.3309EW, 2134 m, 05 Aug 1929, E. D. Ball, 1♀ (00286235) (USNM). Williams, 35.24944EN, 112.19028EW, H. S. Barber, 7♂♂ (00286127- 00286133) (USNM). ***Gila Co.*:** 6 mi S of jct of Rts 87 and 188 (Rt 87 at Forest Service Road 171), 33.9438EN, 111.44946EW, 1006 m, 29 May 1983, R. T. Schuh and G. M. Stonedahl, *Quercus arizonica* Sarg. (Fagaceae), 1♂ (00138491) (AMNH). Old CCC Campground S of Globe on Pioneer Pass Rd, 33.39417EN, 110.78583EW, 1433 m, 30 May 1983–31 May 1983, R.T. Schuh, G.M. Stonedahl and B.M. Massie, *Quercus emoryi* (Fagaceae), 1♀ (00138563) *Quercus emoryi* (Fagaceae), 3♂♂ (00138494-00138496) (AMNH). Pinal Mountains, 33.1656EN, 110.4916EW, 2389 m, 18 Jul 1935, E. D. Ball, 1♀ (00286138) (USNM). ***Graham Co.*:** east end of Aravaipa Canyon, 32.87146EN, 110.37087EW, 1010 m, 09 Jun 1975–10 Jun 1975, McCleve & Topham, 2♂♂ (00138461, 00138462), 1♀ (00138463) (AMNH). ***Greenlee Co.*:** Blackjack Campground, 33.1EN, 109.06666EW, 20 Jul 2001, J. C. Schaffner, 1♀ (00167401) (AMNH). ***Pima Co.*:** 4 mi N of Coronado Natl. Forest boundary on Mount Lemmon Rd, 32.36EN, 110.7EW, 1219 m, 11 Jun 1983, Schuh, Schwartz, and Stonedahl, *Vauquelinia californica* (Rosaceae), 1♂ (00138517), 1♀ (00138535) (AMNH). 7.5 mi S of Coronado Natl. Forest boundary on Mount Lemmon Rd, 32.31EN, 110.72EW, 1433 m, 11 Jun 1983, Schuh, Schwartz, and Stonedahl, *Quercus oblongifolia* Torr. (Fagaceae), 4♂♂ (00138516, 00138518-00138520), 6♀♀ (00138538, 00138539, 00138568-00138571), 1♀ (00138540) (AMNH). NE Tucson, Catalina Hwy, Molina Canyon Outlook, 32.32696EN, 110.70395EW, 1219 m, 01 Jun 1997, T. J. Henry and A. G Wheeler, Jr., *Quercus oblongifolia* Torr. (Fagaceae), 10♂♂ (00286076-00286085), 15♀♀ (00286086-00286099, 00286165), light trap, 1♂ (00286047) (USNM). ***Pinal Co.*:** Oracle, 32.61083EN, 110.77028EW, 1372 m, 05 Jul 1922, E. A. Schwarz, 1♂ (00286149) (USNM); 13 Sep 1925, A. A. Nichol, 1♀ (00286248) (USNM). ***Santa Cruz Co.*:** Santa Rita Mountains, 31.69381EN, 110.93509EW, 1372 m, 09 Sep 1929, A. A. Nichol, 1♂ (00286232) (USNM). ***Yavapai Co.*:** 5 mi N of Wilhoit N of Kirkland, 34.49811EN, 112.58611EW, 1400 m, 19 Jun 1980, R. T. Schuh, *Quercus turbinella* Greene (Fagaceae), 4♂♂ (00138476-00138479), 4♀♀ (00138564-00138567) *Quercus turbinella* Greene (Fagaceae), 1 nymph (00138533) (AMNH). Glen Oaks, 34.44306EN, 112.54778EW, 19 Jul 1929, E. D. Ball, 1♂ (00286233) (USNM). Yarnell Heights, 34.22156EN, 112.75514EW, 1462 m, 21 Jul 1929, E. D. Ball, 1♀ (00286234) (USNM); 12 Jun 1937, E. D. Ball, 1♂ (00286139) (USNM). **Arkansas:** ***Drew Co.*:** Monticello University, 33.58876EN, 91.81259EW, 09 Jun 1987, T. J. Henry and A. G Wheeler, Jr., *Quercus stellata* (Fagaceae), 3♀♀ (00286171, 00286176, 00286177), 4♂♂ (00286172, 00286173, 00286175, 00286174) (USNM). ***Pope Co.*:** Lake Dardanelle State Park, Rt. 64, 13 Jun 1987, T. J. Henry and A. G Wheeler, Jr., *Quercus stellata* (Fagaceae), 2♂♂ (00286178, 00286179), 1 (?sex) (00286180) (USNM). ***Washington Co.*:** Fayetteville, University of Arkansas campus, 36.06258EN, 94.15743EW, 15 Jun 1987, T. J. Henry and A. G. Wheeler, Jr, 1♂ (00286167) *Quercus stellata* (Fagaceae), 3♂♂ (00286168-00286170) (USNM). **California:** ***Sacramento Co.*:** Folsom, 38.67806EN, 121.175EW, 07 Aug 1885, collector unknown, 1♀ (00286150) (USNM). ***Shasta Co.*:** Cayton, 41.06194EN, 121.63278EW, 15 Jul 1918, E. P. Van Duzee, 1♂ (00286249) (USNM). **Colorado:** ***Montezuma Co.*:** Mesa Verde National Park, 37.23333EN, 108.47917EW, 2134 m, 14 Aug 1925, H. H. Knight, 1♂ (00286135) (USNM). **Florida:** ***Marion Co.*:** Dunnellon, 29.04914EN, 82.46093EW, 12 Jun 1939, Oman, 1♂ (00286196) (USNM). ***Volusia Co.*:** Rt. 415, 2 miles N. Osteen, 28.87888EN, 81.1525EW, 27 Apr 1984, T. J. Henry, J. T. Polhemus and A. G. Wheeler Jr., *Quercus virginiana* (Fagaceae), 1♂ (00286166) (USNM). **Kentucky:** ***Calloway Co.*:** Murray, Calloway County Park, 36.60933EN, 88.30716EW, 19 Jun 2001, T. J. Henry and A. G. Wheeler, Jr, *Quercus stellata* (Fagaceae), 3♂♂ (00286153), 2♀♀ (00286154, 00286155) (USNM). **Nevada:** ***Nye Co.*:** 5 mi E of Gabbs off Rt 844, 38.86889EN, 117.82827EW, 1768 m, 01 Jul 1983, R.T. Schuh and M.D. Schwartz, *Purshia glandulosa* (Rosaceae), 1♂ (00138490) (AMNH). Mercury, 36.66056EN, 115.99361EW, 14 Jun 1965, Beck, H. Knight & J. Merino, *Purshia tridentata* (Rosaceae), 2♀♀ (00286240, 00286241), 4♂♂ (00286242-00286245) (USNM). Nevada Atomic Test Site, 2 mi. W. of Tippapah Hwy., on Mine Mt. Rd., 1341 m, 07 Jun 1983, Schuh, Schwartz & Stonedahl, Symphoricarpos oreophilus Gray var. utahensis (Caprifoliaceae), 1♂ (00138521) (AMNH). **North Carolina:** ***Columbus Co.*:** Lake Waccamaw, 34.32194EN, 78.52194EW, 18 m, 06 Jul 1985, W. Steiner & A. Gerberich, 1♂ (00286198) (USNM). ***Harnett Co.*:** Cape Fear, 35.425EN, 78.815EW, 02 Sep 1934, H. S. Barber, 1♂ (00286197) (USNM). ***Mecklenburg Co.*:** Rt. 51, 1mi W of Rt 16, nr Matthews, 04 Jul 1975, A. G. Wheeler, Jr., *Quercus stellata* (Fagaceae), 6♂♂ (00286181-00286186), 1♀ (00286187) (USNM); 02 Jul 1976, A. G. Wheeler, Jr., *Quercus stellata* (Fagaceae), 2♂♂ (00286188, 00286189), 2♀♀ (00286190, 00286191) (USNM). **Oklahoma:** ***Choctaw Co.*:** Hugo Lake, Wilson Point, N. of Sawyer, 16 Jun 1999, T. J. Henry and A. G. Wheeler, Jr, *Quercus stellata* (Fagaceae), 1♂ (00286160) (USNM). Hugo Lake, Wilson Pt., Rt. 147, 34.04EN, 95.37638EW, 95 m, 16 Jun 1999, T. J. Henry and A. G Wheeler, Jr., 4♀♀ (00286156-00286159) (USNM). **Oregon:** ***Jackson Co.*:** Colestin, 42.05323EN, 122.6515EW, 1143 m, 01 Aug 1918, E. P. Van Duzee, 1♂ (00374418) (CNC). **South Carolina:** ***Lancaster Co.*:** Forty Acre Rock, near Taxahaw, 34.66888EN, 80.52694EW, 183 m, 11 Aug 1989, A. G. Wheeler, Jr., *Quercus* sp. (Fagaceae), 1♂ (00286162) (USNM). ***Pickens Co.*:** Clemson University, 34.68333EN, 82.8375EW, 14 Jul 1988, T. J. Henry and A. G Wheeler, Jr., *Quercus stellata* (Fagaceae), 1♂ (00286161) (USNM). **Tennessee:** ***Hamilton Co.*:** collector unknown, 35.17EN, 85.18EW, 23 Aug 1943, Turner, 2♂♂ (00286194, 00286195) (USNM). unknown locality, edge of peach orchard, 24 Jun 1943, Turner, 2♂♂ (00286192, 00286193) (USNM). **Texas:** ***Brazos Co.*:** College Station, 30.62778EN, 96.33417EW, 06 Oct 1928, S. E. Jones, 1♂ (00286199) (USNM); 09 May 1929, H. G. Johnston, 1♂ (00286230) (USNM); 16 May 1929, H. G. Johnston, 3♂♂ (00286226-00286228) (USNM); 22 May 1930, H. G. Johnston, 1♂ (00286229) (USNM). College Station, Riley Estate, 30.58833EN, 96.25333EW, 18 May 2006–19 May 2006, K. Menard, 3♀♀ (00286101-00286103) *Quercus* sp. (Fagaceae), 1♂ (00286100) (USNM). **Utah:** ***Garfield Co.*:** 14.3 mi S of Rt 95 on Rt 276, 3.4 mi N of Starr Springs Campground turnoff, 37.87632EN, 110.56773EW, 1524 m, 19 Jun 1983, R. T. Schuh and M. D. Schwartz, *Quercus turbinella* Greene (Fagaceae), 1♂ (00138515) (AMNH). ***San Juan Co.*:** 3 mi W of Clay Hills Crossing Road on Rt 263, T39S R15E, 37.29533EN, 110.45878EW, 1524 m, 18 Jun 1983, R.T. Schuh and M.D. Schwartz, *Cowania stansburiana* (Rosaceae), 22♂♂ (00138466-00138468, 00138480-00138489, 00138497-00138504, 00138506), 10♀♀ (00138531, 00138572), 1 nymph (00138532) (AMNH). Navajo Mountains (Beaver Creek), 38.35585EN, 109.16314EW, 06 Aug 1936, D.D. Jensen, 1♀ (00286137) (USNM).

#### Ceratocapsidea holguinensis

Taxon classificationAnimaliaHemipteraMiridae

(Hernández & Henry) comb. n.

Figs 38 , 182–185

Ceratocapsus holguinensis Hernández & Henry, 1999: 209 (orig. descrip.); Hernández and Henry 2010: 107 (diag., color habitus, genitalia).

##### Diagnosis.

This species (Fig. 38) is distinguished by the overall shiny, reddishbrown coloration; translucent hemelyral membrane becoming smoky brown on the apical half; the yellowish antenna, with segments III and IV and the apex of II red; and the unique male genitalia, with the bifurcate left paramere (Fig. 182) and the complex right paramere (Figs 184, 185) with two slender arms, each with a crenulate basal process.

##### Description

(in part after Hernández and Henry 1999; holotype measurement not given separately): *Male* (n = 6): Length 2.85–3.45 mm, width 140– ca.1.43 mm [n =2 for width; hemelytra curled in one specimen]. *Head*: Length 0.48 mm, width across eyes 0.60–0.75 mm, interocular width 0.30 mm. *Labium*: Length 1.00 mm. *Antenna*: Segment I length 0.30 mm; II 0.90–1.05 mm; III 0.45–0.54 mm; IV 0.45 mm. *Pronotum*: Length 0.30–0.31 mm, basal width 1.05–1.08 mm.

*Coloration*: General coloration brown. *Head*: Dark brown; eyes reddish black. Labial segments I and II brown; segments III and IV brown to dark brown. *Antenna*: Uniformly yellowish brown. *Pronotum*: Dark shiny brown, sometimes paler brown on anterior margin; scutellum dark brown basally, pale yellow on apex. *Hemelytron*: Brown, with basal half of clavus and corium more yellowish brown, intermixed with fine brown-stained punctures; cuneus dark brown; membrane translucent brown, with cells clear or transparent. *Ventral surface*: Propleura and abdominal segments dark brown. *Ostiolar evaporataive area*: Auricle pale. *Legs*: Uniformly brown to yellowish brown.

*Structure, texture, and vestiture*: *Head*: Slightly rugose, with long, erect, brown setae. *Labium*: Extending between abdominal segments III–IV. *Antenna*: Pubescence short, semierect, yellow. *Pronotum*: Shiny, finely punctate; calli weakly defined, dull, finely granulate. *Scutellum*: Weakly punctate, with scattered pale scale-like setae intermixed with long, erect setae. *Hemelytron*: Subparallel, only slightly constricted on basal half, with distinct, brown, setigerous punctures, especially on clavus and corium. Dorsal vestiture long and erect, intermixed with recumbent scale-like setae, especially on pronotum, scutellum, and hemelytron.

*Male genitalia*: Left paramere (Fig. 182) slender, S-shaped, apical beak-like process slender, weakly pointed distally, middle process broad and distally truncate (rectangular), with a slender basal spine on dorsal side. Right paramere (Figs 184, 185) with two long, recurving arms visible from caudal aspect, the dorsal most arm having a stout, apically rounded, crenulate process at base; and with two processes anteriorly, one short and apically rounded and one long and slender. Phallotheca (Fig. 183): Evenly slender, becoming narrowed and infolded distally.

*Female*: Unknown.

##### Etymology.

This species was named for its occurrence in Holgüin Province, Cuba (Hernández and Henry 1999).

##### Host.

Unknown. Most or all type material was taken at lights (L. Hernández, pers. comm.).

##### Distribution.

Described and previously known only from Holgüin Province, Cuba. Cienfuegos is a new province record.

##### Type material examined.

Holotype ♂: **CUBA:** **Holgüin** [**Prov.**]: Mayari, Loma de l Bandera,, 2.vii.1990, L. F. Armas-V. Beck, charrascal, a la luz, 350 m (IES). Paratypes: 1♂, same data as for holotype (USNM); 1♂, same locality and collectors as for holotype, except with 13.vi.1990, 400 m (USNM).

##### Additional specimen examined.

**CUBA** [**Cienfuegos Prov.**]: Soledad nr.Cienfuegos, 6–20–VIII, N. Banks,1♂ (AMNH).

#### Ceratocapsidea nigropicea

Taxon classificationAnimaliaHemipteraMiridae

(Reuter) comb. n.

Figs 39 , 40 , 186–188

Ceratocapsus nigropiceus Reuter, 1907: 13 (orig. descrip.); Van Duzee 1907: 29 (note); Carvalho 1958: 47 (cat.); Henry and Wheeler 1988: 397 (cat. in part); Carvalho 1990: 193 (descrip., illustr., in part); Schuh 1995: 95 (cat. in part). Lectotype designated by Carvalho 1990: 193.

##### Diagnosis.

This species (Figs 39, 40) is distinguished by the dark brown to fuscous head, pronotum, and scutellum; the paler brown corium and clavus with evenly spaced brown-stained punctures, dark brown femora, the yellowish antenna with segments III and IV tinged with red, and the male genitalia, especially the apically bifid phallotheca (Fig. 187).

##### Description.

*Male* (n = 2; paralectotype measurements in parentheses): Length 2.91 mm (2.94 mm), width 1.00 mm (1.10 mm). *Head*: Width 0.66 mm (0.69 mm), interocular width 0.20 mm (0.22 mm). *Labium*: Length 1.00 mm (1.03 mm). *Antenna*: Segment I, 0.23 mm (0.23 mm); II, 0.70 mm (0.72 mm); III, 0.36 mm (0.40 mm); IV, 0.34 mm (0.36 mm). *Pronotum*: Length 0.56 mm (0.56 mm), basal width 0.98 mm (0.98 mm).

*Coloration*: Overall yellowish brown to darker reddish brown. *Head*: Yellowish brown to dark brown; eyes black. Labium: Yellowish brown, sometimes tinged with red, segment IV often darker brown. *Antenna*: Yellowish brown, segment I with a red dash basally on inner surface, segment III and IV red tinged. *Pronotum*: Uniformly shiny reddish brown to dark brown; scutellum brown to dark brown, paler yellow apically. *Scutellum*: Fuscous to reddish brown, apex slightly paler. *Hemelytron*: Brown to yellowish brown, dark brown on costal area (embolium), cuneus and apical half of corium; membrane translucent brown, narrowly pale across basal margin. *Ventral surface*: Dark brown to dark reddish brown. *Ostiolar evaporative area*: Pale or white, central knob reddish. *Legs*: Coxae pale yellowish brown; fore femur pale yellowish brown, middle femur yellowish brown, dark brown on apical third, hind femur dark brown to dark reddish brown; fore and middle tibiae dark brown, yellowish brown on distal one fourth, hind tibia uniformly dark brown.

*Structure, texture, and vestiture*: *Head*: Dull, finely granulate, with scattered, short, recumbent simple setae, intermixed with a few longer, more erect setae on vertex. *Labium*: Extending to bases of hind coxae. *Pronotum*: Shiny, evenly punctate except for slightly duller granulate calli; evenly set with relatively long, semierect, simple setae. *Scutellum*: Evenly punctate; weakly rugose across basal half, thickly covered with silvery, scale-like setae, intermixed with several long, erect, simple setae. *Hemelytron*: Thickly and evenly covered with brown-stained punctures, less so on cuneus; thickly covered with silvery scale-like setae, especially on clavus and basal half of corium, intermixed with long, semierect, almost bristle-like simple setae.

*Male genitalia*: Left paramere (Fig. 186): Elongate, with a slender neck leading to beak-like apex, middle with two stout, apically acute processes, the longer one parallel with main stem, the basal one shorter and erect. Right paramere (Fig. 188) with one stout, upward-curving arm, having the apex marginally crenulate, and with one short, recurved spine arising from anterior aspect of arm. Phallotheca (Fig. 187) long, slender, with the apex bifid or Y-shaped.

*Female* (n = 5): Length 2.75–3.01 mm, width 1.02–1.21 mm. *Head*: Width 0.62–0.65 mm, interocular width 0.28–0.29 mm. *Labium*: Length 0.98–1.02 mm. *Antenna*: Segment I, length 0.23 mm; II, 0.72 mm; III, 0.39–0.40 mm; IV 0.36 mm. *Pronotum*: Length 0.52–0.56 mm, basal width 0.91–0.99 mm. Very similar to male but with a proportionately broader body and wider vertex.

##### Host.

Unknown.

##### Distribution.

Described and known only from Jamaica (Reuter 1907). Henry and Wheeler’s (1982) report of this species from Florida is a misidentification of *Ceratocapsus balli* (Knight) (which see).

##### Discussion.

Carvalho (1990) designated a male lectotype deposited in the California Academy of Sciences from Mandeville, Jamaica; all other specimens from the original type series are considered paralectotypes. Although the lectotype was not examined, the paralectotypes listed below were studied.

##### Type specimens examined.

Paralectotypes: **JAMAICA:** **Manchester:** Mandeville, 18.03378EN, 77.50012EW, Jan 1906 - Apr 1906, Van Duzee, 1♂ (00286261) (USNM); 3♂♂, 1♀, Mandev’le, Ja, Apr. 06, Van Duzee coll. (1♂ CAS; 1♂ USNM; 1♂, 1♀ ZMUH).

##### Other specimens examined.

**JAMAICA:** **Manchester Parish:** Mandeville, 18.03378EN, 77.50012EW, 618 m, 1700, Unknown, 1♂ (00099697) (MZH). **St. Catherine Parish:** Ferry River, 05 May 1941, Chapin, 3♂♂ (00286257-00286259), 1♀ (00286260) (USNM). Fort Clarence, 07 Dec 1975, O’Brien and Marshall, 3♀♀ (00286253-00286255), 1♂ (00286256) (USNM).

#### Ceratocapsidea rileyi

Taxon classificationAnimaliaHemipteraMiridae

Henry sp. n.

http://zoobank.org/6E4AA477-736D-4E05-A6D8-E5567FD8CD8F

Figs 41 , 189–191

##### Diagnosis.

This species (Fig. 41) can be distinguished by the overall dark brown to fuscous body, antennae, and legs; the weakly constricted hemelytra; the relatively long, nearly erect setae on the hemelytra; and the male genitalia, especially the left paramere (Fig. 189) with two, long middle processes and the right paramere (Fig. 191) with the relatively slender body and a single, slender, upturned process near base.

##### Description.

Holotype *male*: Length 3.39 mm, width 1.31 mm. *Head*: Width 0.80 mm, length 0.35 mm, interocular width 0.24 mm. *Labium*: Length 1.25 mm. *Antenna*: Segment I, length 0.29 mm; II, 0.90 mm; III, 0.48 mm; IV 0.50 mm. *Pronotum*: Length 0.69 mm, posterior width 1.15 mm.

*Coloration*: Overall coloration dark brown or fuscous to almost black. *Head*: black, eyes tinged with red. *Labium*: Dark brown, segments sometimes red tinged. Antenna uniformly fuscous. *Pronotum and scutellum*: Fuscous, nearly black. *Hemelytron*: Dark brown, clavus and cuneus slightly darker brown; membrane translucent smoky brown, becoming paler on basal third. *Ventral surface*: Thorax and propleura dark brown to fuscous; abdomen dark brown. *Ostiolar evaporative area*: Dirty white, tinged with some red; central knob red. *Legs*: Coxae, femora, and tibiae fuscous; tarsi and claws pale brown.

*Structure, texture, and vestiture*: *Head*: Semishiny, strongly rugose, intermixed with recumbent, simple setae; eyes strongly faceted, glabrous. *Labium*: Extending to bases of hind coxae. *Pronotum*: Shiny, shagreened, and finely punctate; calli finely granulate; with evenly spaced recumbent setae, with a few whitish sericeous setae anterior to and between calli. *Scutellum*: Transversely rugose, evenly set with relatively slender, white, scale-like setae, intermixed with long, erect, simple setae. *Hemelytron*: Shiny; clavus finely punctate, corium shagreened on inner half, finely punctate on outer half; evenly set with recumbent simple setae, intermixed with long, erect simple setae and slender, white, scale-like setae on clavus and inner half of corium. Ventral surface and legs shiny; abdominal stridulatory patch distinct.

*Male genitalia*: Left paramere (Fig. 189) relatively stout, distal beak-like process more bulbous with the apex narrowed and truncate and two, long processes at middle, one slender, apically acute, and lying flat against main body and one stouter and slightly extended outward. Right paramere (Fig. 191) relatively slender, with only a small, slender, upturned process near base. Phallotheca (Fig. 190) relatively stout, tapering to a point distally, lower or right apical third finely serrate or crenulate.

*Female*: Unknown.

##### Host.

Unknown. Holotype taken at UV light.

##### Distribution.

Known only from Kenedy County in southeastern Texas.

##### Etymology.

This new species is named after Edward Riley (TAMU), who has collected many new and interesting taxa over the years, including this one.

##### Type material.

Holotype: ♂: **USA:** **Texas:** ***Kenedy Co.*:** Kenedy Ranch, Jaboncillos Pasture, sand dune area, 26.98944EN, 97.66972EW, 21 Apr 2001, Raber, Riley and Yoder, 1♂ (00286262) (TAMU).

#### Ceratocapsidea rufistigma

Taxon classificationAnimaliaHemipteraMiridae

(Blatchley) comb. n.

Figs 42 , 43 , 192–195

Ceratocapsus rufistigmus Blatchley, 1926: 829 (orig. descrip.); Knight 1927a: 103 (note); Carvalho 1958: 49 (cat.); Henry and Wheeler 1988: 398 (cat.); Schuh 1995: 96 (cat.).

##### Diagnosis.

This species (Figs 42, 43) is distinguished by the reddishbrown dorsum with only the costal margin or embolium paler; the evenly distributed brown-stained punctures on the hemelytra; the long, erect setae on the pronotum and hemelytra; the pale antenna with segment III and IV and the apex of II red or tinged with red; and the male genitalia, especially the left paramere (Fig. 192) with the complex middle process with a long, tapering apex and the right paramere (Figs 194, 195) with a broad, curving, apically flared arm and a shorter, crenulate process anteriorly.

##### Description.

*Male* (n = 3): Length 2.94–3.36 mm, width 1.25–1.40 mm. *Head*: Width 0.72–0.75 mm, interocular width 0.22–0.23 mm. *Labium*: Length 0.98–1.01 mm. *Antenna*: Segment I, length 0.23–0.26 mm; II, 0.73–0.83 mm; III, 0.39–0.42 mm; IV, 0.30–0.35 mm. *Pronotum*: Length 0.55–0.59 mm, basal width 1.03–1.12 mm.

*Coloration*: Overall coloration reddish brown. *Head*: Reddish brown; eyes black, frequently tinged with red. *Labium*: pale yellowish brown, often tinged with red, apical half of segment IV dark brown. *Antenna*: Pale yellowish brown, with segment III and IV and apex of II red, segment I with a red mark or dash on inner surface near base. *Pronotum*: Dark reddish brown, with posterior one fourth to one third noticeably paler to almost uniformly reddish brown with only area around calli slightly infuscated; scutellum reddish brown, pale at apex. *Hemelytron*: Reddish brown, cuneus red, costal or embolar margin pale or pale yellowish brown, sometimes tinged with red; membrane translucent brown, narrowly clear or transparent along cuneal margin. *Ventral surface*: Reddish brown, thoracic pleural area and abdomen often becoming more fuscous. *Ostiolar evaporative area*: Pale or white to pinkish, with central knob deep red. *Legs*: Pale yellowish brown, frequently tinged with red, or pale with apices of fore and middle femora, basal one fourth to one half of fore and middle tibiae, and all of hind femur (except base) and tibia red.

*Structure, texture, and vestiture*: *Head*: Frons and vertex strongly granulate, with scattered recumbent and semierect setae, intermixed, with a few long erect setae, especially on vertex. *Labium*: Extending to bases of hind coxae. *Pronotum*: Shiny, with thickly and evenly distributed brown-stained punctures, calli finely granulate, nearly impunctate; set with scattered, long, erect, simple setae, intermixed with silvery, scale-like setae. *Scutellum*: Evenly punctate, weakly transversely rugose, with several long, erect, simple setae, intermixed with silvery, scale-like setae. *Hemelytron*: Evenly distributed with dense, brown-stained punctures; with scattered short, recumbent and long, erect, simple setae, densely intermixed with silvery, scale-like setae.

*Male genitalia*: Left paramere (Fig. 192) with a slender, beak-like apex and a complex middle process having a short basal and dorsal tubercles and a long, slender, tapering apex. Right paramere (Figs 194, 195) relatively stout, with a brown, curving, apically flared arm and short, crenulate tubercle anterior to lateral arm. Phallotheca (Fig. 193) evenly slender on basal half, flaring distally before gradually narrowing into a slender, abruptly curved apical process.

*Female* (n = 5): Length 2.72–3.10 mm, width 1.22–1.42 mm. *Head*: Width 0.65–0.70 mm, interocular width 0.30–0.31 mm. *Labium*: Length 0.99–1.11 mm. *Antenna*: Segment I, 0.25–0.26 mm; II, 0.73–0.91 mm; III, 0.40–0.48 mm; IV, 0.34–0.42 mm. *Pronotum*: Length 0.53–0.65 mm, basal width 0.98–1.14 mm.

##### Host.

One specimen examined was collected on Chinese privet, *Ligustrum sinense* Lour. [Oleaceae], and two others were swept from “ferns”. Both of these plants likely represent incidental occurrences.

##### Distribution.

Previously known only from Florida (Blatchley 1926). North Carolina is a new state record. I note that two PBI database records, a female from Arizona in the AMNH collection and a male in the TAMU collection, were not examined but almost certainly are based on misidentifications.

##### Discussion.

Blatchley (1926) unintentionally validated Knight’s manuscript name of *Ceratocapsus rufistigmus* (Knight 1927a). In studying the unpublished Knight “type series”, his allotype ♀ and one paratype ♀ from Gainesville actually are *Ceratocapsidea complicata* (Knight), clearly recognized by the quadrate black or brown spots on the pronotum. Because of Blatchley’s (1926) unintentional validation, no type was selected by him. In addition, Knight’s own series was mixed. To ensure nomenclatural stability, Knight’s unpublished holotype male is selected as a neotype for *Ceratocapsidea rufistigma* Blatchley.

##### Type designation.

Neotype (here selected) for *Ceratocapsus rufistigma* Blatchley: ♂, label 1) “Gainesville, Fla., 5.5. [19]18” [handwritten date unclear], C. J. Drake”; label 2 (red), “HOLOTYPE by H. H. Knight Ceratocapsus rufistigmus [name handwritten]”; and label 3 (red, here added), “NEOTYPE: ♂ *Ceratocapsus rufistigmus* Blatchley desig. by T. J. Henry” (00286228) (USNM).

##### Other specimens examined.

**USA:** **Florida:** ***Alachua Co.*:** Gainesville, 29.63527EN, 82.37111EW, 24 m, 14 Jul 1918, C. J. Drake, 1♀ (00246624) (TAMU).Gainesville, 29EN, 82EW, 21 Jun 1918, C. J. Drake, 1♀ (00286265) (USNM). Univ. Florida Agricultural Experimental Station, 29.72108EN, 82.41816EW, 53 m, 05 May 1918, C. J. Drake, 1♂ (00286268) (USNM); 31 May 1918, C. J. Drake, 1♀ (00286266) (USNM); 04 Aug 1918, C. J. Drake, 1♀ (00286267) (USNM). ***Duval Co.*:** Jacksonville, 30.33194EN, 81.65583EW, 23 Jul 1926, E. D. Ball, 1♂ (00246625) (TAMU). Jacksonville, 30.33194EN, 81.65583EW, 23 Jul 1926, E. D. Ball, 2♀♀ (00286263, 00286264) (USNM). ***Highlands Co.*:** Highlands Hammock State Park, 27.47138EN, 81.54138EW, 26 m, 22 Apr 1981–28 Apr 1981, T. J. Henry, fern, 2♀♀ (00286272, 00286273) (USNM). ***Liberty Co.*:** Torreya State Park, 30.56889EN, 84.94778EW, 03 May 1976, Joe Schuh, 3♂♂ (00138457-00138459), 1♀ (00138460) (AMNH). ***Marion Co.*:** Rt. 40, 12 mi E of Lynne, 29.18191EN, 81.71137EW, 12 m, 24 Apr 1984, T. J. Henry and A. G. Wheeler, Jr., *Pinus clausa* (Chapm. ex Engelm.) Sarg. (Pinaceae), 1♂ (00286269) (USNM). ***Miami-Dade Co.*:** Westgate, 25.83806EN, 80.25139EW, 11 Apr 1962, J. F. Brimley, 1♀ (00374727) (CNC). ***Pinellas Co.*:** Dunedin, 28.01972EN, 82.77166EW, 9 m, 21 Nov 1920, W. S. Blatchley, 1♂ (00374725) (CNC). ***Seminole Co.*:** Sanford, 28.80055EN, 81.27312EW, 24 Jul 1927, E. D. Ball, 1♂ (00286270), 1♀(00286271) (USNM). ***Volusia Co.*:** South Daytona, 29.16556EN, 81.00472EW, 21 Mar 1960, J. F. Brimley, 1♀ (00374726) (CNC). **North Carolina:** ***New Hanover Co.*:** Carolina Beach, 34.035EN, 77.89389EW, 08 Jun 1950, D. M. DeLong, fern, 1♂ (00286274) (USNM).

#### Ceratocapsidea taeniola

Taxon classificationAnimaliaHemipteraMiridae

Henry sp. n.

http://zoobank.org/9CEF8BFE-F093-4732-B6FE-D787F74F7752

Figs 44 , 196–199

##### Diagnosis.

This species (Fig. 44) is distinguished by the overall dark brown to fuscous coloration, pale brown antennae, distally dark brown hind femora, and male genitalia, especially the apically trifid phallotheca (Fig. 197) with a long, broad, ribbon-like apical process and two shorter, parallel processes below and right paramere (Figs 198, 199) with two upturned arms.

##### Description.

*Male* (n = 2; holotype measurements in parentheses): Length 3.64 mm (3.46 mm), length 1.32 mm (1.20 mm). *Head*: Width 0.73 mm (0.74 mm), interocular width 0.23 mm (0.24 mm). *Labium*: Length 1.31 mm (1.39 mm). *Antenna*: Segment I, length 0.33 mm (0.31 mm); II, 0.94 mm (0.88 mm); III 0.53 mm (0.51 mm); IV, 0.47 mm (0.44 mm). *Pronotum*: Lenght 0.73 mm (0.72 mm); basal width 1.25 mm (1.17 mm).

*Coloration*: Overall coloration dark brown to fuscous or almost black. *Head*: Fuscous, paler laterally below antennal bases. *Labium*: Uniformly brown. *Antenna*: Segments I and II brown; segment III and IV dark brown to fuscous. *Pronotum*: Uniformly shiny fuscous, collar slightly paler brown; scutellum fuscous, paler brown on apical third. *Hemelytron*: Uniformly, shiny dark brown, costal or emboliar margin and cuneus darker brown or fuscous; membrane uniformly translucent brown. *Ventral surface*: Shiny dark brown, becoming darker or fuscous laterally and distally on abdomen. *Ostiolar evaporative area*: Pale brown, central knob of auricle slightly darker. *Leg*: Coxae pale or whitish; fore and middle femora pale or white, with distal third tinged with brown, hind femur pale or whitish on basal half, dark brown on distal half; tibia, tarsi, and claws brown.

*Structure, texture, and vestiture*: *Head*: Finely granulate, becoming somewhat wrinkled on frons; with scattered, short, recumbent, simple setae on vertex and frons, and several long, erect setae at base of vertex. *Labium*: Extending beyond hind coxae to abdominal segment I or II. *Pronotum*: Disk shiny, with evenly distributed punctures; calli finely granulate, becoming finely wrinkled between and anteriorly. *Scutellum*: Evenly punctate, finely, transversely rugose; thickly set with relatively short, recumbent and semierect simple setae, intermixed with long, reclining, almost bristle-like setae. *Hemelytron*: Shiny, evenly and densely punctate, densely set with silvery, scale-like setae, intermixed with long, nearly erect, weakly curved, bristle-like setae.

*Male genitalia*: Left paramere (Fig. 196) slender, with a well-developed, beak-like distal process coming to a point at each end and a stout apically acute middle process, with a short, slender dorsally directed tubercle arising at base. Right paramere (Figs 198, 199) relatively stout, with two upturned, apically crenulate arms. Phallotheca (Fig. 197) evenly slender to trifid apex, ending in a long, apically acute, ribbon-like process and two shorter, more slender, nearly parallel processes just below.

*Female*: Unknown.

##### Etymology.

The specific epithet *taeniola* is taken from the Latin “*taenia*”, meaning ribbon, and the diminutive suffix “ola”, in reference to the small ribbon-like apex of the phallotheca (Fig. 197).

##### Host.

Unknown.

##### Distribution.

Known only from Jamaica.

##### Type material.

Holotype ♂: **JAMAICA:** **St. Andrew Parish:** Holywell Forest Camp, 1219 m, 11 Aug 1971, M. Winegar (00286276) (USNM). Paratype: **JAMAICA:** **St. Andrew Parish:** Holywell Forest Camp, 1219 m, Sep 1971 - Oct 1971, M. Winegar, 1♂ (00286275) (USNM).

#### Ceratocapsidea texensis

Taxon classificationAnimaliaHemipteraMiridae

Henry sp. n.

http://zoobank.org/21EAC2D8-E43F-43E3-8497-425BFF15E75A

Figs 45 , 46 , 200–203

##### Diagnosis.

This species (Figs 45, 46) is distinguished by the small size, oval body form, the uniformly pale yellowishbrown appendages, and the male genitalia, especially the phallotheca (Fig. 201) with a long, slender apex and quadrate, marginally crenulate, subapical process.

##### Description.

*Male* (n = 7; holotype measurements in parentheses): Length 2.82–3.14 mm (2.88 mm), width 1.25–1.37 mm (1.34 mm). *Head*: Width 0.64–0.70 mm (0.68 mm), interocular width 0.26–0.29 mm (0.25 mm). *Labium*: Length 0.94–1.05 mm (0.95 mm). *Antenna*: Segment I, length 0.23 mm (0.25 mm); II, 0.68–0.78 mm (0.73 mm); III, 0.36–0.43 mm (0.42 mm); IV, 0.30–0.35 mm (0.32 mm). *Pronotum*: Length 0.55–0.61 mm (0.59 mm), basal width 1.03–1.16 mm (1.13 mm).

*Coloration*: Overall coloration yellowish brown to pale reddish brown. *Head*: Yellowish brown to darker brown; eyes dark brown. *Labium*: Pale yellowish brown, segment IV darker brown. *Antenna*: Uniformly pale yellowish brown, except for a red dash on inner surface at base of segment I. *Pronotum*: Reddish brown to dark brown, with evenly spaced dark-stained punctures; scutellum reddish brown to brown, apex paler. *Hemelytron*: Yellowish or reddish brown to brown, clavus paler than corium; on paler reddish specimens costal margin and middle of cuneus becoming red, on darker specimens middle of cuneus dark brown; membrane translucent brown, except narrowly pale along cuneal border. *Ventral surface*: Reddish brown to dark brown, ventral area of abdomen sometimes paler brown. *Ostiolar evaporative area*: Pale or white with central knob red or darker than surrounding area. *Legs*: Uniformly pale yellowish brown.

*Structure, texture, and vestiture*: *Head*: Finely granulate, with scattered, relatively long, pale simple setae, especially on frontal area. *Labium*: Extending to bases of middle coxae. *Pronotum*: Shiny, evenly punctate, calli indistinct, finely granulate, with numerous short, recumbent and long erect and semierect, simple setae, intermixed with scattered silvery, scale-like setae. *Scutellum*: Evenly punctate, with a few long, erect, simple setae, thickly intermixed with silvery scale-like setae. *Hemelytron*: Evenly punctate on corium and clavus, less so on cuneus, with numerous long, erect, simple setae, intermixed with silvery scale-like setae, especially on clavus and basal half of corium.

*Male genitalia*: Left paramere (Fig. 200): Slender, with a distinct beak-like process distally, and two short, slender processes at middle, one apically acute, one blunt. Right paramere (Figs 202, 203): Stout, apically tapered, with two lateral arms, the dorsal arm shortest, straight, distally crenulate, the lower arm stoutest, longest, apically hooked with two short lateral spines or processes basally. Phallotheca (Fig. 201): Slender, with a long, slender distal process and a more quadrate, marginally crenulate, subapical process.

*Female* (n = 3): Length 2.27–2.84 mm, width 1.13–1.46 mm. *Head*: Width 0.60–0.68 mm, interocular width 0.33–0.34 mm. *Labium*: Length 0.91–0.99 mm. *Antenna*: Segment I, length 0.21–0.25 mm; II, 0.60–0.72 mm; III, 0.33–0.40 mm; IV 0.33–36 mm. *Pronotum*: Length 0.48–0.59 mm, basal width 0.91–1.12 mm.

##### Host.

Unknown.

##### Distribution.

Known only from four eastern Texas counties (Brazos, Duval, Uvalde, and Victoria).

##### Etymology.

This species is named after the state of Texas where all of the type material was collected.

##### Type material.

Holotype ♂: **USA:** **Texas:** ***Uvalde Co.*:** Concan, 29.49523EN, 99.71255EW, 116 m, 04 Jun 1933, P. W. Oman (00286277) (USNM). Paratypes: **USA:** **Arizona:** ***Santa Cruz Co.*:** Patagonia, 31.53944EN, 110.75556EW, 1219 m, 12 Jun 1928, A. A. Nichol, 1♂ (00286287) (USNM). **Texas:** ***Brazos Co.*:** College Station, 30.62778EN, 96.33417EW, 16 May 1929, H. G. Johnston, 1♂ (00286288) (TAMU). ***Duval Co.*:** San Diego, 27.76391EN, 98.23889EW, 01 May 1900, collector unknown, 1♂ (00286284), 1♀ (00286285) (CUIC). ***Uvalde Co.*:** Concan, 29.49523EN, 99.71255EW, 116 m, 04 Jun 1933, P. W. Oman, 6♂♂ (00286283) (USNM). ***Victoria Co.*:** Victoria, 28.805EN, 97.00333EW, Sep 1900, collector unknown, 1♂ (00286286) (USNM).

#### Ceratocapsidea transversa

Taxon classificationAnimaliaHemipteraMiridae

Henry sp. n.

http://zoobank.org/DAF2752B-9587-4FE2-908E-D453562AE40F

Figs 47 , 204–207

##### Diagnosis.

The species (Fig. 47) is recognized by the oval body form, brown to reddishbrown coloration, long setae on antennal segment II, the distinct transverse brown band on the pronotum, and the male genitalia, especially the left paramere (Fig. 204) with a “wing-like” middle process, the single stout lateral arm on the right paramere (Figs 206, 207), and the relatively stout phallotheca (Fig. 205) with a slender recurved apex.

##### Description.

*Male* (n = 4; holotype measurements in parentheses): Length 3.04–3.36 mm (3.23 mm), width 1.38–1.39 mm (1.34 mm). *Head*: Width 0.70–0.73 mm (0.78 mm), interocular width 0.23–0.25 mm (0.25 mm). *Labium*: Length 1.01–1.05 mm (1.00 mm). *Antenna*: Segment I, length 0.25–0.26 mm (0.26 mm); II, 0.78–0.81 mm (0.75 mm); III, 0.39 mm (0.39 mm); IV, 0.30–0.31 mm (0.35 mm) [see discussion below on an aberrant right antenna in this species]. *Pronotum*: Length 0.62 mm (0.61 mm), basal width 1.14–1.18 mm (1.17 mm).

*Coloration*: Overall coloration brown, often tinged with red. *Head*: Uniformly yellowish brown; eyes dark brown to fuscous, tinged with red around margins. *Labium*: Yellowish brown, segment I and II tinged with red, segment IV darker brown. *Antenna*: Pale yellowish brown, with a narrow red band encircling dorsal half near base and distal half tinged with red; segment II yellowish brown, apex weakly red tinged; segments III and IV slightly darker brown or reddish. *Pronotum*: Brown to yellowish brown, more brown anteriorly, paler brown on posterior third, with a broad dark brown to fuscous band across middle and narrowly fuscous across anterior margin. *Scutellum*: Dark brown, with apex yellowish brown. *Hemelytron*: Brown to dark brown, corium and apical half of cuneus tinged with red; costal margin or embolium pale yellowish brown. *Ventral surface*: Shiny reddish brown; stridulatory area appearing paler or whitish. *Ostiolar evaporative area*: Pale or whitish, with central knob red. *Legs*: Coxae pale; fore and middle femora pale yellowish brown, hind femur yellowish brown with apical third reddish; fore and middle tibiae yellowish brown with a narrow red stripe on basal third to half of anterior face, hind tibiae brown to red on basal half to three fourths; tarsi and claws yellowish brown.

*Structure, texture, and vestiture*: *Head*: Finely granulate on frons and vertex; set with short, recumbent, simple setae, intermixed with 8–10 long, erect setae. *Labium*: Extending to bases of middle coxae. *Antenna*: Segment I with scattered short, recumbent and two long erect, simple setae; segments II–IV with numerous short recumbent and longer, semierect setae, some on segment II equal to or longer than the diameter of the segment. *Pronotum*: Shiny, evenly punctate, except granulate calli; thickly covered with long, semierect, simple setae. *Scutellum*: Evenly punctate, with scattered long, erect and semierect simple setae, intermixed with slender, silvery, scale-like setae. *Hemelyron*: Evenly distributed with numerous brown-stained punctures; set with numerous long, erect, almost bristle-like, simple setae, intermixed with slender silvery, scale-like setae.

*Male genitalia*: Left paramere (Fig. 204): Beak-like apex distally blunt and with a wing-like process at middle. Right paramere (Fig. 206, 207): Main trunk stout, apically tapered, with one stout lateral arm having a slender, upturned, crenulate apical process and a slender process midway between base and apex. Phallotheca (Fig. 205): Relatively stout, with slender apex, recurved.

*Female* (n = 2): Length 3.23–3.32 mm, width 1.35–1.46 mm. *Head*: Width 0.68–0.69 mm, interocular width 0.31–0.33 mm. *Labium*: Length 1.03–1.04 mm. *Antenna*: Segment I, length 0.23–0.25 mm; II, 0.74–0.78 mm; III, 0.36–0.38 mm; IV, 0.30 mm. *Pronotum*: Length 0.59 mm, basal width 1.08–1.11 mm.

Very similar to male in general coloration and texture, differing mainly in the more broadly oval body form, as well as the distinctive long setae (3× or more the diameter of the segment) on antennal segment II that are absent in males.

##### Host.

Unknown.

##### Distribution.

Known only from Nuevo León, Mexico.

##### Etymology.

This species is named “transversa” for the dark brown or black, transverse band across the middle of the pronotum.

##### Discussion.

One paratype (CNC specimen with red label added: “SPECIMEN WITH TERATOLOGICAL RIGHT ANTENNA”) of this species exhibits an aberrant right antenna, with segment II (length 0.88 mm) much longer than the same segment (length 0.78 mm) on the left antenna, and segments III and IV fused (length 0.40 mm) and tapered as in segment IV (missing on this individual) on the left antenna of other specimens. Although antennal abnormalities are not rare in insects, few such teratological cases have been reported in the Miridae. Also, see discussion under *Pilophoropsidea brailovskyi* sp. n.

##### Type material.

Holotype ♂: **MEXICO:** **Nuevo León:** Chipinque Mesa, near Monterrey, N.L., 25.60422EN, 100.36137EW, 5400’ [1646 m], 22 Jul 1963, H. F. Howden, 1♂ (00286296) (CNC). Paratypes: **MEXICO:** **Nuevo León:** Chipinque Mesa, near Monterrey, N.L., 25.60422EN, 100.36137EW, 5400’ [1646 m], 08 Jul 1963, H. Howden & A. Howden, 1♂ (00286289) (USNM); 22 Jul 1963, H. F. Howden, 5♂♂ (00286291-00286295) (CNC), 1♂ (00286290) (USNM).

#### Ceratocapsidea variabilis

Taxon classificationAnimaliaHemipteraMiridae

Henry sp. n.

http://zoobank.org/7C35F88E-F232-453A-B637-BAA46F354FDB

Figs 48 , 208–210

##### Diagnosis.

This species (Fig. 48) is distinguished by the overall dark, shiny dorsum, dark brown antennae and legs, evenly and finely punctate pronotum, silvery sericeous setae confined to the the scutellum, clavus, and inner margin of the corium, and the male genitalia with a very slender beak-like process on the left paramere (Fig. 208), two arms on the right paramere (Fig. 210), and the apically slender phallotheca (Fig. 209).

##### Description.

*Male* (n = 4; holotype measurements in parentheses): Length 3.36–3.68 mm (3.62 mm), width 1.12 mm [hemelytra distorted in other specimens] (1.27 mm). *Head*: Width 0.75–0.81 mm (0.83 mm), interocular width 0.22–0.25 mm (0.26 mm). *Labium*: Length 1.08–1.24 mm (1.27 mm). *Antenna*: Segment I, length 0.26–0.29 mm (0.29 mm); II, 0.73–0.88 mm (0.91 mm); III, 0.39–0.42 mm (0.43 mm); IV, 0.36–0.43 mm (0.40 mm). *Pronotum*: Length 0.61–0.72 mm (0.65 mm), basal width 1.08–1.24 mm (1.22 mm).

*Coloration*: Overall coloration dark brown to fuscous. *Head*: Dark brown; eyes dark brown, sometimes fading to silvery brown. *Labium*: Uniformly brown. *Antenna*: Uniformly dark brown. *Pronotum*: Uniformly shiny dark brown or fuscous; fuscous, paler yellowish brown at apex. *Hemelytron*: Uniformly dark brown to fuscous, sometimes paler dark brown on corium and clavus, leaving costal margin and cuneus darker or fuscous. *Ventral surface*: Evenly dark, shiny brown. *Ostiolar evaporative area*: Somewhat dark pinkish brown, central knob brown. *Legs*: Coxae pale or whitish, narrowly tinged with brown at bases; femora dark brown, except at bases; tibiae, tarsi, and claws uniformly brown.

*Structure, texture, and vestiture*: *Head*: Frons transversely rugose, vertex finely granulate, with scattered short, recumbent simple setae, intermixed with a few longer erect setae. *Labium*: Extending to hind coxae or base of abdomen. *Antenna*: Segment I sparsely scattered with short, recumbent setae and two longer, erect setae at middle of inner surface; remaining segments thickly set with very short, recumbent setae. *Pronotum*: Evenly punctate, except for finely granulate calli; with scattered short, recumbent simple setae, intermixed with longer, nearly bristle-like simple setae and silvery, scale-like setae. *Scutellum*: Very finely punctate and transversely rugose; thickly set with silvery scale-like setae, intermixed with long, erect setae. *Hemelytra*: Shiny, evenly punctate, with scattered short, recumbent simple setae, intermixed with long, erect, bristle-like setae and thickly set silvery scale-like setae along clavus and inner margin of corium.

*Male genitalia*: Left paramere (Fig. 208): Elongate, with distal beak-like process very slender, two middle processes, one stout, lying flat against main body toward apex, one short, erect, and a down-curved acute spine at base. Right paramere (Fig. 210): Main stem stout, tapered at apex, with two lateral arms, upper one shortest, distally curved upward, lower one longest, stoutest, dorsally curved at apex, and a T-shaped process attached behind arms. Phallotheca (Fig. 209): Stout, tapered distally into a slender spine.

*Female*: Unknown.

##### Etymology.

This species is given the name “variabilis” for the somewhat variable antennal and leg coloration, ranging from uniformly dark brown in the holotype to paler yellowish brown in some paratypes.

##### Host.

Unknown.

##### Distribution.

Known only from Jamaica.

##### Type material.

Holotype ♂: **JAMAICA:** **St. Andrew Parish:** Holywell Forest Camp, 4000’ [1219 m], 01 Sep 1971, M. Winegar (00286299) (USNM). Paratypes: **JAMAICA:** **Clarendon Parish:** Alston, 2000’ [610 m], 19 Sep 1972, C. Crickett, 1♂ (00286302) (USNM); 29 Nov 1972, C. Crickett, 1♂ (00286301) (USNM); 11 Dec 1972, C. Crickett, 1♂ (00286303) (USNM); 10 Jan 1973, C. Crickett, 1♂ (00286304) (USNM); 28 Feb 1973, C. Crickett, 1♂ (00286305) (USNM). **St. Andrew Parish:** Holywell Forest Camp, 4000’ [1219 m], 15 Feb 1972, M. Winegar, 1♂ (00286297) (USNM); 29 Jul 1972, M. Winegar, 1♂ (00286298) (USNM). **St. Catherine Parish:** Linstead, 18.13666EN, 77.03166EW, 430’ [131 m], 07 Dec 1970, J. A. Slater and R. Baranowski, 1♂ (00286300) (USNM).

#### Marinonicoris

Taxon classificationAnimaliaHemipteraMiridae

Carvalho

Marinonicoris Carvalho, 1988: 877 (orig. descrip.); Schuh 1995: 141 (cat.). Type species: *Marinonicoris myrmecoides* Carvalho, 1988. Original designation. Monotypic.

##### Diagnosis.

This ant mimetic genus is recognized by the convex, finely punctate, shiny pronotum, the shiny constricted hemelytron with a large patch of golden scale-like setae through the middle of the clavus and corium and bordered on either side by a narrow band of silvery scale-like setae, and the male genitalia, especially the parameres. All known males and females are macropterous.

##### Description.

Myrmecomorphic. Males and females macropterous. Length of males 2.98–3.36 mm, length of females 2.62–2.98 mm. Head broader than long, shiny, alutaceous, posterior margin weakly concave, base finely carinate, posterior margin of eyes level with base of vertex; eyes large, elongate oval, occupying more than half dorsal width of head, laterally occupying nearly 90♂ of height; eyes much smaller in females, occupying little more the one third dorsal width of head; front broadly rounded, clypeus acute. Labium extending to about middle coxae (imbedded in glue); segment I short, not extending beyond buccular sheath. Antennal somewhat thickened, subequal to two times diameter of tibiae; segment I shortest; segment II longest, gradually thickening to apex; segments III and IV subequal, fusiform. Pronotum similar in male and female; shiny, very finely punctate, calli more alutaceous; convex, raised above level of scutellum and hemelytra; lateral margin weakly sulcate, posterior angles flared; with a few short, recumbent simple setae. Mesoscutum hidden by posterior margin of pronotum. Scutellum equilateral, somewhat concave from base to apex; with a broad band of silvery scale-like setae through middle. Hemelytra shiny throughout, weakly constricted through middle; costal margin in males and females with a distinct row of small black spicules along costal margin; each hemelytron with two rows of silvery scale-like setae, one across base of corium and clavus and one through middle of corium and apex of clavus; area between silvery bands densely covered with golden silvery scale-like setae; membrane entire, with two areoles. Ventral surface shiny, impunctate; abdomen with long, semierect, simple setae; glaucous stridulatory patch distinct. Ostiolar evaporative area dark reddish brown, with a distinct shiny red knob on auricle. Legs unmodified; parempodia fleshy, convergent. Male aperture large, unarmed. Left paramere elongate, apical beak-like process slender, middle process short, slender, apically rounded. Right paramere stout with one slender, apically bifid arm. Phallotheca stout, with three comb-like processes.

##### Discussion.

In the original description, Carvalho (1988) compared this genus with *Renodaeus* and said “differs from *Renodaeus* by the absence of long setae on the body”. *Marinonicoris* is superficially similar to *Renodaeus* in overall appearance, including the somewhat swollen pronotum, the constricted hemelytra, dark, shiny brown coloration, and the large patches of golden, scale-like setae on the clavus and corium. It differs from *Renodaeus*, however, in lacking erect bristle-like setae on the hemelytra and the file-like spicules on the costal margin, as well as having quite different parameres, more closely resembling those found in *Pilophoropsis*.

#### Marinonicoris myrmecoides

Taxon classificationAnimaliaHemipteraMiridae

Carvalho

Figs 93 , 94 , 280–282

Marinonicoris myrmecoides Carvalho, 1988: 877 (orig. descrip.); Carvalho and Froeschner 1990: 328 (type data, depository); Schuh 1995: 141 (cat.); Carpintero et al. 2006: 8 (list, distr.).

##### Diagnosis.

This species (Figs 93, 94) is recognized by the generic characters, including the swollen pronotum, constricted hemelytra, the golden and silvery scale-like setae on the clavus and corium, the lack of erect, bristle-like dorsal setae, and the male genitalia, especially the parameres (Figs 280, 282).

##### Description.

*Male* (n = 2): Length 2.98–3.36 mm, width 1.14–1.18 mm. *Head*: Width 0.72–0.77 mm, interocular width 0.30–0.34 mm. *Labium*: Imbedded in glue. *Antenna*: Segment I, length 0.16–0.21 mm; II, 0.51–0.58 mm; III, 0.30–0.37 mm; IV, 0.34–0.40 mm. *Pronotum*: Length 0.51–0.58 mm, basal width 0.91–1.04 mm.

*Coloration*: *Head*: yellow brown to dark brown. *Antenna*: Segment I yellowish brown, with a red streak on inner margin; segments II–IV dark brown. *Pronotum*: Dark brown, area around calli sometimes slightly paler brown. *Scutellum*: Brown. *Hemelytron*: Dark brown, area on basal half of corium and clavus appearing paler brown because of setal coloration. *Ventral surface*: Thoracic area yellowish brown, mesopleural area with a glaucous sheen; abdomen dark brown to fuscous, glaucous stridulatory patch distinct. *Ostiolar evaporative area*: Dark brown, with a red knob at middle of auricle. *Legs*: Dark brown, apices of hind femora and tibiae and all of tarsi and claws pale yellowish brown.

*Structure, texture, and vestiture*: As in generic description. Head, pronotum, and hemelytra shiny. *Labium*: Extending about to hind coxae or base of abdomen. Each hemelytron with two rows of silvery scale-like setae, one across the base of the corium and clavus, and one across the middle of the corium and apex of the clavus; area between densely covered with golden scale-like setae (but lacking erect, bristle-like setae).

*Male genitalia*: *Left paramere* (Fig. 280): Elongate, distal stem-like, with beak-like apex elongate, basal half stouter with a short, slender, apically rounded arm. *Right paramere* (Fig. 282): Stout, with a slender, apically bifid arm near base. *Phallotheca* (Fig. 281): Stout, apex with three comb-like processes.

*Female* (n = 4): Length 2.62–2.98 mm, width 1.02–1.18 mm. *Head*: Width 0.70–0.72 mm, interocular width 0.30–0.35 mm. *Labium*: Imbedded in glue. *Antenna*: Segment I, length 0.16–0.18 mm; II, 0.50–0.53 mm; III, 0.55 (others missing) mm; IV missing. *Pronotum*: Length 1.00–1.03 mm, basal width 0.80–0.91 mm. Similar to male in color, texture, and vestiture.

##### Distribution.

Previously known only from the holotype taken in Paraná, Brazil. Carpintero et al. (2006) reported specimens from Argentina, Paraguay, and Uruguay. Rio Grande Norte is a new Brazilian state record.

##### Host.

Unknown.

##### Discussion.

The holotype, though not borrowed, was examined during a visit to the Museu Nacional collection (MNHN) in Rio de Janeiro. This species is easily distinguished by the structure of the male genitalia.

##### Type material examined.

Holotype ♂, **BRAZIL:** ***Paraná*:** Telêmaco Borba, Reserva Samuel Kalbin, [24.35ES 50.6167EW], 669 m, 02 Nov 1986, Lev. Ent. Profau Par, lâmpada (00175020) (MNRJ).

##### Other specimens examined.

**ARGENTINA:** **Chaco:** Fontana, Unknown, 1♀ (00071514) (USNM); 1♂, Martin Garcia Isl., Buenos Aires Prov., Nov. 96, M. C. Coscarón (MACN); 1♂, Arroya Martires, Misiones, Aug. 1995, D. L.Carpintero (MACN); 1♀, Corrientes Prov., Bella Vista, June 1936 (MACN). **BRAZIL:** **Sao Paulo:** Piracicaba, 22.73931ES 47.655EW, Unknown, 1♀ (00071513) (USNM). 1♂, Natal, Rio Grand Norte, Feb. 1952, M. Alvarenga (MNHN). **PARAGUAY:** 1♂, Arroya Aguapey, Mar. 1994, D. L. Carpintero (MACN). **URUGUAY:** 1♀, Rivera Dept., Sierra and Arroya de al Aurora, 12 Jan. 1971 (MACN).

#### Pilophoropsidea

Taxon classificationAnimaliaHemipteraMiridae

Henry gen. n.

http://zoobank.org/2DDD1CFE-A09D-4B0D-B3D2-A77E4BFA7EAF

##### Type species:

*Ceratocapsus camelus* Knight, 1930.

##### Diagnosis.

Characterized by the relatively long labial segment I that extends beyond the gular sulcus by nearly half its length; the strongly convex pronotum, having nearly straight lateral margins that narrow anteriorly before ending to accentuate a distinct narrow collar; the hemelytra uniformly polished, subparallel to weakly contricted through middle (Figs 51, 56, 62), always having two loose bands of silvery scale-like setae, a narrow one at the base of the corium and clavus and a broader one through the middle of the corium onto the apical third of the clavus, and a single row of long, erect, often bristle-like, simple setae on the clavus; and a distinctive ostiolar area (Fig. 110) having a swollen, usually dull red area or knob at the apex of the scent channel.

##### Description.

Myrmecomorphic. Males macropterous; females brachypterous. Length of males 3.60–5.40 mm. Head broader than long; posterior margin straight or truncate, distinctly carinate, posterior margin of eyes level with base of vertex, hypognathus; eyes large, elongate oval, occupying half or more of dorsal head width, laterally occupying more than 70♂ of height; front rounded, declivent, clypeus acute, visible from dorsal aspect. Labium extending to middle or hind coxae; segment I extending beyond gular sulcus by about half its length, basal half visible below buccula in lateral aspect. Antenna somewhat thickened, subequal to diameters of tibiae; segment I shortest; II longest, gradually enlarging apically to thickness of segments III and IV; III and IV fusiform. Pronotum in males trapeziform, wider at base than long, dull to polished, lateral margins straight or nearly so, anterior margin with a distinct narrow collar separated from calli by a transverse groove; posterior margin weakly rounded; mesoscutum partially visible to hidden by base of pronotum; scutellum equilateral. Pronotum in females more nearly quadrate, disc and calli strongly convex, with hind margin sharply sloping downward posteriorly. Hemelytra shiny to polished, lateral margins subparallel to weakly constricted near level with middle of corium, area at apex of corium and base of cuneus usually depressed; all species with two bands of loosely set, silvery, scale-like setae, one across base of clavus (and across scutellum, but usually disjunct at claval suture) and one through middle of corium and across apical third of clavus, intermixed with a row of long, erect, simple setae through middle of clavus and a few on inner corial margin bordering membrane. Cuneus and membrane fully developed in males. Claval suture absent and apex of cuneus at level across membrane abbreviated in females, exposing apical 3 or 4 abdominal segments, cuneus and membrane entirely wanting in some species. Ventral surface shiny; ostiolar area white, with a distinct knob or raised area at the end of scent channel; second visible abdominal segment with area of dense microsetae (Fig. 112), appearing as a dull or glaucous quadrate patch. Legs unmodified, except the hind tibiae in some species sometimes thickened, somewhat bowed, and laterally compressed; parempodia fleshy and convergent apically. Male aperture of genital capsule large, open, sometimes armed with small to large spines or processes around margins; left paramere variable from beak-like to more slender and apically truncate; right paramere usually with a slender main stem and various lateral arms or spines; phallotheca elongate, slender, variously apically hooked to sometimes more truncate.

##### Etymology.

The name of this new genus is taken from the generic name *Pilophoropsis* Poppius and Latin suffix “*idea*”, meaning form or appearance, and is used to reflect the resemblance of the two genera. The gender is feminine.

##### Discussion.

Twelve of the 15 species included in this new genus are described as new. Most are represented by only one or a few specimens, suggesting that numerous additional species likely will be discovered with more thorough collecting.

The following key includes some external characters to help distinguish species, but male genitalia (viewed caudally, usually without dissection) are required to identify members of this genus with certainty.

##### Key to the males of *Pilophoropsidea*

**Table:** 

1	Pronotum dull black, surface grainy or granulate	**2**
–	Pronotum shiny brown to dark brown, surface polished to finely granulate	**5**
2	Basal half of cuneus thickly clothed with silvery scale-like setae (Fig. 55); left paramere (Fig. 220) distally beak-like with two long dorsal projections; right paramere straight with a slender lateral projection (Fig. 222); phallotheca (Fig. 221) with a long, arched apical process; margins of aperture unarmed; Mexico (Chiapas)	***cuneata* sp. n.**
–	Basal half of cuneus devoid of silvery, scale-like setae (Fig. 68)	**3**
3	Ostiolar auricle, including central knob, uniformly white; left paramere (Fig. 254) globose at base with a long apical process; right paramere (Fig. 256) quadrate; phallotheca (Fig. 255) long with only a short apical hook; aperture with a broad truncate process at one o’clock position; Mexico (Guerrero)	***truncata* sp. n.**
–	Ostiolar auricle white, but with central knob distinctly red	**4**
4	Head, pronotum, and hemelytra black; antennal segment II uniformly black; left paramere (Fig. 257) shallowly C-shaped without lateral processes; right paramere (Fig. 259) stout, bifurcate; phallotheca (Fig. 258) slender with a short acute, apical process; genital capsule with a large tubercle at one o’clock position above aperture rim; Mexico (Guerrero)	***tuberculata* sp. n.**
–	Head and hemelytra reddish brown, only pronotum black; antennal segment II yellow basally, reddish brown on apical third; left paramere (Figs 250, 251) with a stout dorsal process and a smaller anteriorly directed process not visible from caudal aspect; right paramere (Fig. 253) stout with a bifurcate lateral arm; phallotheca (Fig. 252) stout with slender, apical process; aperture unarmed; Mexico (Puebla)	***touchetae* sp. n.**
5	Right paramere C-shaped (Fig. 219) or with a long C-shaped arm (Figs 213, 233)	**6**
–	Right paramere simple, composed of a stout, straight main stem, with only short notches or tubercles laterally (Fig. 209)	**9**
6	Ostiolar auricle uniformly white, right paramere (Fig. 233) with a spine at middle; left paramere, with a long apical process and a large, wing-shaped median process (Fig. 230); phallotheca (Fig. 232) apically blunt; Mexico (Durango) and the United States (Arizona, New Mexico)	***fuscata* sp. n.**
–	Ostiolar auricle with a red knob at middle; right paramere without a median spine; left paramere without wing-shaped process; phallotheca with a short to long, acute apical process	**7**
7	Right paramere (Fig. 213) forming a large open “C”; corium with relatively long, erect simple setae half or more times as long, erect setae on clavus; United States (Arizona)	***barberi* (Knight)**
–	Right paramere (Figs 219, 245) forming a stout, tight “C”; corium with only a few short, reclining, simple setae	**8**
8	General coloration brown to reddish brown; main body of left paramere (Fig. 217) stout and broad; phallotheca (Fig. 218) with a long slender apex; United States (midwestern states)	***camelus* (Knight)**
–	General coloration very dark brown to nearly black; main body of left paramere (Fig. 245) more slender with a smaller, quadrate dorsal process; phallotheca (Fig. 244) with a short, slender apex; Mexico (Nuevo León, San Luis Potosi)	***schaffneri* sp. n.**
9	Phallotheca (Fig. 228) with a long, slender, apical process	**10**
–	Phallotheca (Fig. 241) apically blunt or with only a short apical process	**13**
10	Left paramere (Fig. 227) elongate with a long, slender, basal process or a stout median process; right paramere (Fig. 229) simple, lacking a distinct lateral tubercle or deep notch; United States (Arizona)	***fascipennis* (Knight)**
–	Left paramere relatively stout; right paramere with a distinct lateral tubercle or notch	**11**
11	Left paramere (Fig. 226) with a serrate, triangular-shaped process on side; phallotheca (Fig. 225) lacking a serrate or toothed edge below decurved apical process; Mexico (Durango)	***dimidiata* sp. n.**
–	Left paramere lacking a triangular-shaped process; phallotheca with serrations or a tooth on Edge below apical process	12
12	Right paramere (Fig. 236) with a pointed lateral process directed upward; left paramere (Fig. 234) with an elongate, apically blunt, basal process; phallotheca (Fig. 235) with a tooth and serrations below apical process; Mexico (Durango)	***keltoni* sp. n.**
–	Right paramere (Fig. 239) with a stout, blunt, lateral process; left paramere (Fig. 237) with a stout, apically acute, basal process; phallotheca (Fig. 238) with only a tooth below apical process; Mexico (Durango)	***maxima* sp. n.**
13	Phallotheca (Fig. 241) apically blunt; right paramere (Fig. 242) apically blunt; left paramere (Fig. 240) with two acute basal spines and slender apex notched; Mexico (Puebla)	***pueblaensis* sp. n.**
–	Phallotheca apically acute; right paramere more apically rounded; basal process of left paramere without two acute spines and an apical notch	**14**
14	Dorsal edge of male genital aperture with 12 or 13 short setigerous tubercles; phallotheca (Fig. 248) with a recurved flap opposite apical process; right paramere (Fig. 249) with a quadrate lateral notch; apical section of left paramere (Figs 246, 247) dorsally acute and apically truncate; Mexico (Michoacan)	***serrata* sp. n.**
–	Dorsal edge of male genital aperture without setigerous tubercles, instead with a larger spine over base of left paramere; phallotheca (Fig. 215) strongly serrate or toothed opposite dorsal process; apical section of left paramere (Fig. 214) dorsally rounded and apically acute; Mexico (Federal District)	***brailovskyi* sp. n.**

#### Pilophoropsidea barberi

Taxon classificationAnimaliaHemipteraMiridae

(Knight) comb. n.

Figs 49 , 211–213

Ceratocapsus barberi Knight, 1930: 190 (orig. descrip.); Carvalho 1958: 44 (cat.); Henry and Wheeler 1988: 393 (cat.); Schuh 1995: 90 (cat.).

##### Diagnosis.

This species (Fig. 49) is recognized by the overall reddishbrown color; the shiny pronotum with a fine, grainy surface; erect, relatively long, simple setae on the corium; and the shape of the male genitalia, particularly the right paramere. The right paramere (Fig. 213) is unique in having a globose base and a long arm forming a curved C-shaped structure.

##### Description.

*Holotype male*: Length: 4.10 mm, width 1.32 mm. *Head*: Width 0.83 mm, interocular width 0.30 m. *Labium*: Length about 1.48 mm (embedded in glue). *Antenna*: Segment I, length 0.32 mm; II, 1.00 mm; III, 0.60 mm; IV, 0.56 mm. *Pronotum*: Length 0.68 mm, basal width 1.10 mm.

*Coloration*: *Head*: Dark reddish brown. *Antenna*: Dark reddish brown. *Pronotum*: Dark reddish brown to fuscous. *Scutellum*: Fuscous. *Hemelytron*: Dark reddish brown; cuneus shiny reddish brown; membrane smoky brown. *Ventral Surface*: Shiny reddish brown, abdomen darker brown or fuscous. *Ostiolar evaporative area*: Pale or whitish, central knob or raised area red. *Legs*: Coxae, femora, and tibiae uniformly reddish brown; tarsi and claws pale yellowish brown.

*Structure, texture, and vestiture*: *Head*: Semishiny, finely granulate, frons weakly transversely striate, with scattered semierect pale yellowishbrown setae. *Labium*: extending to about middle coxae. *Pronotum*: Semishiny, finely granulate, with scattered, semierect and erect, pale yellowishbrown setae *Scutellum*: Shiny, finely transversely rugose, with scattered, long, erect, bristle-like, pale setae and a broad band of narrow, white, scale-like setae. *Hemelytron*: Polished, impunctate, except for an irregular double row of shallow, indistinct brown punctures on clavus; clavus with five long, erect, bristle-like pale setae lengthwise through middle; corium with a few short, pale, simple setae, intermixed with longer, erect, pale setae and two bands of silvery scale-like setae, a relatively narrow one across base of clavus (and through scutellum) and a broader one across middle of corium and apex of clavus. *Ventral surface*: Propleura dorsoventrally rugose, remainder of thorax and abdomen shiny, polished.

*Male genitalia*: Left paramere (Fig. 211) elongate, with apex more slender and curved downward. Right paramere (Fig. 213) with a globose base and a long, slender lateral arm forming a broad C-shape. Phallotheca (Fig. 212) stout, apex blunt and slightly curved.

*Female*: Unknown.

##### Distribution.

Known from only the holotype collected in the Huachuca Mountains of Arizona.

##### Host.

Unknown.

##### Discussion.

I have not found any other material that I can associate with the holotype of this species. The male genitalia, however, are quite distinct among the species of *Pilophoropsidea*.

##### Type specimen examined.

Holotype ♂ [**USA**]: **Arizona [Cochise Co.**]: Huachuca Mts., VII–20, H. G. Barber coll., collected by light (USNM) [00162212].

#### Pilophoropsidea brailovskyi

Taxon classificationAnimaliaHemipteraMiridae

Henry sp. n.

http://zoobank.org/D0E177B7-2968-4B0A-B34F-6164B4FD4379

Figs 50 , 214–216

##### Diagnosis.

This species (Fig. 50) is recognized by the relatively large size, reddishbrown coloration, unique male genitalia, particularly the left paramere (Fig. 214) and phallotheca (Fig. 214), and the stout spine on the male genital aperture at about the 10 o’clock position above the left paramere.

##### Description.

*Holotype male*: Length ca. 4.48 mm (hemelytra bent down and twisted apically), width 1.36 mm. *Labium*: Length 1.58 mm. *Antenna*: Segment I, 0.40 mm; II, teratoid left segment 1.26 mm, normal right segment 1.08 mm; III, right 0.56 mm; IV, right 0.54 mm; teratoid left III and IV fused, 0.80 mm, fuscous. *Pronotum*: Length 0.88 mm, basal width 1.36 mm.

*Coloration*: *Head*: Dark brown, frons dark reddish brown. *Antenna*: Segment I pale brown, with a brown to reddishbrown streak on basal half; segment II fuscous; segments III and IV fuscous. *Pronotum*: Reddish brown. *Scutellum*: Reddish brown. *Hemelytron*: Apical half fuscous, slightly paler or very dark brown on basal half; membrane smoky black, pale or whitish through areoles and between. *Ventral surface*: Shiny brown to reddish brown, abdomen more fuscous. *Ostiolar evaporative area*: White with large knob at end of scent channel dull red. *Legs*: Fore coxa brown, more reddish apically, middle and hind coxae pale or whitish, infuscated basally; femora brown to fuscous, with apices pale; tibiae brown, hind tibia more reddish brown, all becoming paler on apical 1/4; tarsi and claws brown.

*Structure, texture, and vestiture*: *Head*: Semishiny, vertex finely granulate with a narrow, smooth, shiny area extending between eyes just before base; frons transversely striate. *Labium*: Extending to bases of hind coxae. *Pronotum*: Disc shiny, smooth to very indistinctly roughened, calli granulate, slightly raised, narrowly shiny around inner margins; collar transversely striate. *Scutellum*: Weakly rugose, with silvery scale-like setae except at middle of base and at apex *Hemelytron*: Polished, smooth except for slightly roughened clavus; with typical bands of silvery scale-like setae, a narrow one at base of clavus and a broader one through middle of corium and apical third of clavus, intermixed with a row of long, erect, simple setae through middle of clavus and a few along inner corial margin near base of membrane.

*Male genitalia*: Aperture with a stout, apically acute tubercle above left paramere Left paramere (Fig. 214): Apex quadrate and extended distally into a long slender process; basally with three processes, the first and third short, apically rounded, the middle one longer, more pointed. Right paramere (Fig. 216) stout, elongate, narrowing apically, without lateral tubercles or processes. Phallotheca (Fig. 215): Stout, distally broad and blunt; finely serrate along one side near apex.

*Female*: Unknown.

##### Etymology.

This species is named in honor of coreoid specialist Dr. Harry Brailovsky, friend, colleague, and collector of the holotype.

##### Distribution.

Federal District, Mexico.

##### Host.

Holotype collected on *Tillandsia bourgari* Baker [Bromeliaceae].

##### Discussion.

The holotype (red label added: “SPECIMEN WITH TERATOLOGICAL LEFT ANTENNA”) and only known specimen of this species exhibits the only case of teratological antennal development observed in *Pilophoropsidea*. The left second antennal segment is much longer than the right and segments III and IV of the same antenna are fused into one. Polhemus and Polhemus (1985) noted that antennal oligomery was not an uncommon phenomenon in the Heteroptera, citing Leston (1952), and described such examples as *Pilophoropsis brachyptera* Poppius treated in this paper and their new genus and species *Phorodendrepulus myrmecomorphus* (Phylinae). They speculated that flightlessness and extensive inbreeding accounted for a higher occurrence of antennal oligomery in *Phorodendrepulus myrmecomorphus* than in *Pilophoropsis bracyptera*, which has fully macropterous, more mobile males [as do species of *Pilophoropsidea*] to increase interdeme gene flow.

##### Type material.

Holotype: ♂: **MEXICO:** **Federal District:** Temascaltepec, 9 Feb. 1979, H. Brailovsky, on *Tillandsia bourgari* Baker (UNAM).

#### Pilophoropsidea camela

Taxon classificationAnimaliaHemipteraMiridae

(Knight) comb. n.

Figs 51–54 , 107 , 108–115 , 116–119 , 217–219

Ceratocapsus camelus Knight, 1930: 187 (orig. descrip.); Knight 1941: 114 (descrip., key, genitalia); Froeschner 1949: 172 (note, key); Carvalho 1958: 44 (cat.); Akingbohungbe et al. 1972: 9 (note); Henry and Wheeler 1988: 393 (cat.); Schuh 1995: 91 (cat.); Henry et al. 2005: 52 (list).

##### Diagnosis.

This species (Figs 51–54) is recognized by the brown to reddishbrown coloration, usually with the anterior half of the hemelytron paler than the posterior half; the smooth, polished pronotum; the uniformly white ostiolar auricle, lacking a red knob; and the male genitalia, particularly the right paramere (Fig. 219) forming a stout C-shaped structure and the phallotheca (Fig. 218) with a long, slender, recurving, apical process.

##### Description.

*Male* (n = 10; holotype measurements in parentheses): Length 3.60–3.96 mm (3.78 mm), width 1.16–1.18 mm (1.06 mm). *Head*: Width 0.76–0.78 mm (0.72 mm), interocular width 0.36-0.40 mm (0.37 mm). *Labium*: Length 1.36–1.42 mm (1.37 mm). *Antenna*: Segment I, length 0.24–0.26 mm (0.24 mm); II, 0.75–0.78 mm (0.75 mm); III, 0.44–0.48 mm (0.46 mm); IV, 0.44–0.46 mm (0.45 mm). *Pronotum*: Length 0.78–0.84 mm (0.80 mm), basal width 1.08–1.12 mm (1.10 mm).

*Coloration*: *Head*: Brown. *Antenna*: Segment I, pale brown; segment II, dark brown, paler on basal half; segments III and IV dark brown to fuscous. *Pronotum*: Shiny dark brown, paler anteriorly; scutellum brown, paler along margins. *Hemelytron*: Uniformly polished, dark brown, paler brown on clavus and basal l/2 of corium; membrane smoky black beyond apex of cuneus, pale across and between areoles. *Ventral surface*: Shiny brown, abdomen becoming dark brown or black. *Ostiolar evaporative area*: Uniformly white. *Legs*: Coxae pale brown, apex of procoxa sometimes reddish, base of middle coxa sometimes infuscated; femora dark brown, hind femur pale at extreme apices; tibiae brown, becoming paler on apical halves; tarsi and claws pale.

*Structure, texture, and vestiture*: *Head*: Shiny, vertex weakly granulate, frons weakly transversely rugose, with numerous long, slender, simple, brown setae. *Labium*: Extending to bases of hind coxae. *Antenna*: Segment II slender basally, gradually enlarging to apex; setae short, recumbent. *Pronotum*: Strongly convex, disc smooth, polished, calli granulate, depressed collar transversely striate, sparsely set with very short, palebrown, recumbent setae; scutellum with a wide band of silvery scale-like setae through middle, intermixed with long, erect, pale brown, simple setae. *Hemelytron*: A band of silvery scale-like setae across base of clavus (and continuous with band on scutellum) and a wider band through middle of corium and across apical l/3 of clavus, sparsely intermixed with very long, pale brown setae on clavus and inner margin of corium. *Ventral surface*: Abdomen sparsely set with short, pale brown, simple setae and a few longer erect setae on genital capsule; second visible abdominal segment with dull alutaceous stridulatory patch on middle half.

*Male genitalia*: Aperture large with a sharp, inwardly directed spine above left paramere. Left paramere (Fig. 217) relatively short and stout, quadrate on apical half, with apex pointed and curved downward; long, erect, basal process stout and bifid apically (apex somewhat variable, with secondary spine sometimes poorly developed). Right paramere (Fig. 219) stout, C-shaped. Phallotheca (Fig. 218) elongate, weakly constricted before widening distally, apex tapered into a long, slender, recurving process.

*Female* (n=3): Coloration and structure similar to that of male, except as noted below. Length to apex of abdomen 2.96–3.05 mm, length to apex of hemelytron 2.25–2.50 mm, width across apex of hemelytra 1.24–1.28 mm. *Head*: Width 1.44–1.46 mm, interocular width 0.48–0.50 mm. *Labium*: Length 1.44–1.46 mm. *Antenna*: Segment I, length 0.26 mm; II, 0.74–0.78 mm; III, 0.42–0.44 mm; IV 0.44–0.46 mm. *Pronotum*: Length 0.72 mm, basal width 0.70–0.74 mm.

*Coloration*: *Head, pronotum, and scutellum*: Brown. *Ventral surface*: Brown, abdomen becoming black, with long, slender, erect, pale setae. *Ostiolar evaporative area*: White. Legs similar to those of male.

*Structure, texture, and vestiture*: *Labium*: Extending to hind coxae. *Pronotum*: Quadrate, shiny brown, strongly convex, sides rounded, disc and calli continuously rounded, disc shiny, not cleft, calli weakly rugose, anterior flattened, collar-like area short, but distinctly transversely striate, downward sloping posterior end of disc rugose, posterior margin flattened, posterior angles flared outward. *Scutellum*: Depressed through middle, apex with a patch of silvery scale-like setae. *Hemelytron*: Brachypterous, polished, narrow on basal half, flaring wider apically, strongly depressed across basal third to half, apical half of corium with a shiny, glabrous swelling, claval suture absent, fusing clavus and corium, cuneal fracture absent, apex truncate, apical margin cutting inward forming a shallow V with both hemelytra together; patch of silvery scale-like setae at base of corium and a semicircular band extending from middle of outer corial margin around inside of hump and back across apex to outer margin of cuneal area, claval area and inner corial margin with long, erect, pale brown setae; apical three abdominal segments exposed beyond hemelytra.

##### Distribution.

Previously known in the United States from Illinois, Kentucky, Missouri, and Wisconsin (Henry and Wheeler 1988, Henry et al. 2005). New state records are Arkansas, Indiana, Nebraska, Oklahoma, and Texas.

##### Hosts.

Henry et al. (2005) reported taking *Campsis camela* from trumpet creeper, *Campsis radicans* (L.) Seem [Bignoniaceae], and poison ivy, *Toxicodendron radicans* (L.) Kuntze [Anacardiaceae]. In addition to these hosts, A. G. Wheeler and I have collected *Pilophoropsidea camela* from a broad range of plants and habitats, including *Celtis* sp. (hackberry) [Ulmaceae], *Fraxinus* sp. (ash) [Oleaceae], *Quercus stellata* Wangenh. (post oak) [Fagaceae], *Parthenocissus quinquefolia* (L.) Planch. (Virginia creeper) [Vitaceae], *Ulmus crassifolia* Nutt. (winged elm) [Ulmaceae], thickets of *Campsis radicans* (L.) Seem. ex Bureau [Bignoniaceae], and from a thick hedge-row of vegetation. Specimens usually are beaten from dense vegetation or from larger, often moss- or lichen-covered branches of trees, particularly species of *Quercus*, such as post oak. This wide assortment of collection sites and hosts suggests that this mirid is an opportunistic predator, probably seeking injured, dead, or dying soft-bodied arthropods as prey.

We also have observed a general association of this mimetic species with ants. In Arkansas, we found *Pilophoropsidea camela* more common when *Crematogaster clara* Mayr was present. A specimen of *Crematogaster lineolata* (Say) in the USNM collection is associated with a male of *Pilophoropsidea camela* collected by R. C. Froeschner in Missouri (23 July 1942).

##### Type specimens examined.

Holotype: ♂: **USA:** **Illinois:** ***Champaign Co.*:** “Urbana, Ill., Aug. 21, 1926, Vera Smith” [00162213] (USNM). Paratypes: **USA:** **Illinois:** ***Champaign Co.*:** Urbana [40.11056EN, 88.20722**E** W], 21 Aug 1926, Vera Smith, 1♀ (allotype) (00071557) (USNM).

##### Other specimens examined.

**USA:** **Arkansas:** ***Perry Co.*:** Rt. 60, near Conway, Toadsuck Park, 35.07198EN, 92.52377EW, 91 m, 12 Jun 1987, T. J. Henry and A. G. Wheeler, Jr, 2♂♂ (00071562, 00071563), 1♀ (00071564) *Campsis radicans* (Bignoniaceae), *Parthenocissus quinquefolia* (Vitaceae), and *Toxicodendron radicans* (Anacardiaceae), 1♂ (00071561) *Populus deltoides* (Salicaceae), 1♂ (00071560) (USNM). ***Pope Co.*:** Lake Dardanelle State Park, Rt. 64, 13 Jun 1987, T. J. Henry and A. G Wheeler, Jr., *Quercus stellata* (Fagaceae), 1♀ (00285689) (USNM). Russellville, Arkansas Tech University, 35.27842EN, 93.13379EW, 13 Jan 1987, T. J. Henry and A. G. Wheeler, Jr, 1♂ (00071574) *Ulmus crassifolia* (Ulmaceae), 1♀ (00071575), 3♂♂ (00071576-00071578) (USNM). **Illinois:** ***Madison Co.*:** Glen Carbon, 38.74833EN, 89.98306EW, 04 Sep 1950, J. A. Slater, 1♂ (00138451) (AMNH). **Indiana:** ***Cass Co.*:** Logansport, Dykeman Park, 1 Jul 1984, T. J. Henry, from thick hedgerow of *Campsis radicans* (Bignoniacaeae), 1♀ (USNM). **Kentucky:** ***Fulton Co.*:** CR-430, nr Mississippi River, 2.1 mi. N. of Sassafras Ridge, 36.55555EN, 89.22388EW, 19 Jun 2001, T. J. Henry and A. G Wheeler, Jr., *Toxicodendron pubescens* (Anacardiaceae), 1♂ (00285685) *Campsis radicans* (Bignoniaceae), 1♂ (00285686), 1♀ (00285687) (USNM). **Missouri:** ***Cole Co.*:** Jefferson City, 38.5584EN, 92.17203EW, 30 m, 23 Jul 1942, R. C. Froeschner, willow, 1♀ (00071559) (USNM); 11 Jul 1944, W. W. Dowdy, 1♂ (00285691) (USNM). ***St. Louis Co.*:** Saint Louis, 38.62722EN, 90.19778EW, 03 Sep 1939, R. C. Froeschner, 1♂ (00071558) (USNM). **Nebraska:** ***Nemaha Co.*:** Peru, Neal Park, 40.46972EN, 95.73277EW, 09 Jul 1986, T. J. Henry and A. G. Wheeler, Jr, beaten from thick hedge of mixed vegetation 1♂ (00285690) (USNM). **Oklahoma:** ***Choctaw Co.*:** Hugo Lake, Wilson Pt., Rt. 147, 34.04EN, 95.37638EW, 95 m, 16 Jun 1999, T. J. Henry and A. G Wheeler, Jr., *Quercus stellata* (Fagaceae), 2♀♀ (00071569, 0071570) (USNM). ***Murray Co.*:** I77, at I-35 W. of Doughtery, 316 m, 34.39444EN, 97.13861EW, 13 Jun 1999, T. J. Henry and A. G Wheeler, Jr., *Ulmus crassifolia* (Ulmaceae), 1 (?sex) (00071565), 1♂ (00071566), 1♀ (00071567) (USNM). ***Sequoyah Co.*:** Applegate Cove, Kerr Lake, 6 mi. S. of Sallisaw, 18 Jun 1999, T. J. Henry and A. G Wheeler, Jr., *Quercus stellata* (Fagaceae), 1♂ (00285688) (USNM). **Texas:** ***Brazos Co.*:** College Station, 30.62778EN, 96.33417EW, 15 May 1933, H. G. Johnston, 3♂♂ (00071571-00071573) (USNM). College Station, 30.62778EN, 96.33417EW, 20 May 1929, H. G. Johnston, 1♂ (02467796) (TAMU); 15 May 1933, H. G. Johnston, 1♂ (02467799) (TAMU); 18 May 1933, H. G. Johnston, 1♂ (02467798) (TAMU); 19 May 1933, H. G. Johnston, light trap, 1♂ (02467800) (TAMU); 23 May 1933, H. G. Johnston, 1♂ (02467801) (TAMU); 24 May 1933, H. G. Johnston, 1♂ (02467797) (TAMU). ***Kerr Co.*:** Kerrville, VI–16–64, M. H. Sweet, 1♀ (TAMU). ***Uvalde Co.*:** 29.20968EN, 99.78617EW, 26 May 1910, F. C. Pratt, 1♂ (00071568) (USNM). **Wisconsin:** ***Jackson Co.*:** no specific locality, 44.43718EN, 90.91126EW, 259 m, 08 Aug 1970, C. F. Koval, 1♂ (00329553) (CNC).

#### Pilophoropsidea cuneata

Taxon classificationAnimaliaHemipteraMiridae

Henry sp. n.

http://zoobank.org/DE2D0A7D-3313-4AFE-812C-C35BA24DCC98

Figs 55 , 220–222

##### Diagnosis.

This species (Fig. 55) is recognized by the overall very dark brown head and hemelytra, contrasting with the dull or, at most, satiny, black pronotum; the field of silvery, scale-like setae across the basal half of the cuneus, and the male genitalia, particularly the left paramere (Fig. 220) with a long basal process.

##### Description.

*Male* (n = 2; holotype measurements in parentheses): Length 4.03 mm (4.10 mm), width 1.10 mm (1.04 mm). *Head*: Width 0.76 mm (0.74 mm), interocular width 0.36 mm (0.32 mm). *Labium*: Length 1.30 mm (1.28 mm). *Antenna*: Segment I, length 0.30 mm (0.27 mm); II, 0.84 mm (0.80 mm); III, 0.54 mm (0.50 mm); IV, 0.52 mm (0.46 mm). *Pronotum*: Length 0.78 mm (0.74 mm), basal width 1.06 mm (1.04 mm).

*Coloration*: *Head*: Dark reddish brown. *Antenna*: Segment I pale brown, basal half with an elongate red mark on inner side; segment II fuscous to black; segment III and IV black. *Pronotum*: Fuscous to black. *Scutellum*: Dark brown to fuscous, apex pale brown. *Hemelytron*: Uniformly dark brown to fuscous; membrane uniformly smoky black. *Ventral surface*: Shiny reddish brown to fuscous. *Ostiolar evaporative area*: White, with knob at end of scent channel dull red. *Legs*: Fore coxa dark brown, middle and hind coxae white, narrowly fuscous at bases; femora uniformly dark reddish brown; tibiae dark reddish brown, hind tibiae noticeably flattened; tarsi and claws brown.

*Structure, texture, and vestiture*: *Head*: Granulate, frons with weak transverse striations. *Labium*: Extending just beyond bases of middle coxae. *Pronotum*: Evenly granulate, dull to weakly shiny, collar paler brown and transversely rugose. *Scutellum*: Finely granulate, smooth, with silvery scale-like setae across apical half except apex, intermixed with long, erect, simple setae. *Hemelytron*: Smooth, polished, with three bands or patches of silvery scale-like setae, one across base of clavus (and through scutellum), a broader one through middle of corium and apical third of clavus, and one across basal half of cuneus, intermixed with sparse, recumbent simple setae and a row of long, erect setae through middle of clavus and a few along inner corial margin at base of membrane. Ventral surface: Abdomen with numerous long, erect setae, especially on genital capsule.

*Male genitalia*: Aperture with a large tubercle along dorsal edge just left of center (approx. at 11 o’clock position) that is thickened on basal half, then abruptly slender apically. Left paramere (Fig. 220): Apex subtriangular or beak-like, middle with a relatively stout, erect process, and base with a very long, forward-curving, slender process. Right paramere (Fig. 222) elongate, with a short, slender process at middle. Phallotheca (Fig. 221) slender, apex with a stout, recurved hook.

*Female*: Unknown.

##### Etymology.

The specific epithet *cuneata* is used to denote the silvery, scale-like patch of setae present across the basal half of the cuneus.

##### Distribution.

Chiapas, Mexico.

##### Host.

Unknown.

##### Type material.

Holotype: ♂: **MEXICO:** **Chiapas**, 27 km SW Teopisca, 18 Sept. 1981, Clark and Coe (TAMU). Paratype: Same data as for holotype, 1♂ (USNM). [00162215].

#### Pilophoropsidea dimidiata

Taxon classificationAnimaliaHemipteraMiridae

Henry sp. n.

http://zoobank.org/6B31D535-D6E8-4C07-AA84-8B454B70A4A1

Figs 56 , 223–226

##### Diagnosis.

This species (Fig. 56) is recognized by the small size; the brown to reddishbrown coloration; the shiny to satiny brown pronotum having a finely granulate surface; and the male genitalia, particularly the stout left paramere (Fig. 223) with a triangular-shaped lateral process.

##### Description.

*Male* (n = 2; holotype mesurements in parentheses): Length 3.76 mm (3.84) mm, width 1.24 mm (1.18 mm). *Head*: Width 0.76 mm (0.74 mm), interocular width 0.40 mm (0.38 mm). *Labium*: Length 1.40 mm (1.44 mm). *Antenna*: Segment I, length 0.30 mm (0.26 mm); II, length 0.94 mm (0.92 mm); III, 0.58 mm (0.53 mm); IV, 0.54 mm (0.52 mm). *Pronotum*: Length 0.83 mm (0.80 mm), basal width 1.06 mm (1.10 mm).

*Coloration*: *Head*: Reddish brown. *Antenna*: Segment I pale brown, with a slender red to brown mark on basal third; segment II brown, darker apically; segment III and IV reddish brown. *Pronotum*: Reddish brown to fuscous. *Scutellum*: Reddish brown to dark brown. *Hemelytron*: Uniformly polished, orange brown on basal half, brown to fuscous on apical third, with a narrow band of silvery scale-like setae at base of clavus and a wider band through middle of corium and apical third of clavus, intermixed with a row of long, erect, pale, simple setae on clavus and inner corial margin bordering membrane; membrane smoky black beyond apex of cuneus, pale or whitish across areoles and between. *Ventral surface*: Shiny reddish brown, becoming fuscous on abdomen; ostiolar area white, with large anterior knob at end of scent channel dull red. *Legs*: Procoxa brown, red apically, middle and hind coxae whitish, base of middle coxa brown; femora brown, apex of hind femur pale on ventral aspect; tibiae reddish brown to dark brown, fore and middle tibiae pale on apical 1/4; tarsi and claws pale brown.

*Structure, texture, and vestiture*: *Head*: Finely granulate, transverse striations on frons absent or extremely vague, pubescence short, recumbent, with a few long, erect, pale setae across base of vertex and basal area around clypeus. *Labium*: Extending to bases of hind coxae or to posterior margin of metasternum. *Antenna*: Segment II slender, only slightly thickening to apex. *Pronotum*: Disc and calli finely granulate, satiny, anterior collar transversely rugose, setae short, sparse, and recumbent.

*Male genitalia*: Aperture with a short, stout spine along margin just above left paramere. Left paramere (Figs 223, 224): stout, truncate apex narrowed and decurved; middle with three processes, one thick and rounded apically and two acute apically (with anterior most one hidden from view in caudal aspect, i.e. *in situ*), and a central marginally crenulate triangular flange at widest part. Right paramere (Fig. 226): stout, with a lateral, hook-like process directed upward. Phallotheca (Fig. 225): stout, with a long, slender, sharply recurved hook.

*Female*: Unknown.

##### Etymology.

The specific epithet *dimidiata* is used to denote the small size of this species.

##### Distribution.

Durango, Mexico.

##### Host.

Unknown.

##### Type material.

Holotype ♂: **MEXICO:** **Durango:** 9 mi. W La Ciudad Durango, 9000’, 1 July 1964, L. A. Kelton (00167381) (CNC). Paratype: **MEXICO:** **Durango:** 24 mi. W. La Ciudad, Durango, 7000’, 21 July 1964, L. A. Kelton, 1♂ (USNM) [00162217].

#### Pilophoropsidea fascipennis

Taxon classificationAnimaliaHemipteraMiridae

(Knight) comb. n.

Figs 57 , 227–229

Ceratocapsus fascipennis Knight, 1930: 189 (orig. descrip.); Carvalho 1958: 46 (cat.); Henry and Wheeler 1988 (cat.): 394; Schuh 1995: 92 (cat.).

##### Diagnosis.

This species (Fig. 57) is recognized by the brown to reddishbrown coloration; shiny to satiny brown pronotum; and the male genitalia, particularly the left paramere (Fig. 227) with an elongate beak-like apex, a long, curving basal process, and a stouter, apically acute, middle process.

##### Description.

*Male* (n = 5; holotype measurements in parentheses): Length 4.16–4.32 mm (4.24 mm), width 1.44–1.52 mm (1.22 mm). *Head*: Width 0.78–0.80 mm (0.80 mm), interocular width 0.38–0.40 mm (0.37 mm). *Labium*: Length 1.36–1.38 mm (1.41 mm). *Antenna*: Segment I, length 0.26–0.28 mm (0.24 mm); II, 0.78–0.86 mm (0.67 mm); III, 0.44–0.48 mm (0.38 mm); IV, 0.42–0.44 mm (0.37 mm). *Pronotum*: Length 0.90–0.92 mm (0.86 mm), basal width 1.18–1.22 mm (1.20 mm).

*Coloration*: *Head*: Reddish brown. *Antenna*: Segment I pale brown, with inner surface having a red line extending along entire length in most specimens; segment II dark brown, pale on basal third; segments III and IV dark brown to fuscous. *Pronotum*: Dark reddish brown to fuscous, sometimes paler on anterior third. *Scutellum*: Dark brown to fuscous. *Hemelytron*: Uniformly orange brown on basal half, cuneus and apical half of corium dark brown; membrane fuscous beyond apex of cuneus, clear whitish basally. *Ventral surface*: Shiny, thoracic sterna orange brown to brown, abdomen fuscous. *Ostiolar evaporative area*: White, with large anterior knob dull reddish brown. *Legs*: Coxae whitish, base of middle coxae and apex of fore coxae brown to reddish brown; femora brown to reddish brown; tibiae brown to reddish brown, fore and middle tibiae becoming pale on apical third; claws and tarsi pale brown.

*Structure, texture, and vestiture*: *Head*: Frons weakly transversely rugose, with long, semierect and erect, pale-brown setae along midline and across vertex. *Labium*: Extending to middle coxae. *Pronotum*: Shiny, disc and calli finely granulate, nearly alutaceous, anterior collar granulate, weakly transversely striate; pubescence short, sparse. *Scutellum*: With loose band of silvery scale-like setae across apical third. *Hemelytron*: Polished; silvery scale-like setae set in a narrow band across base of clavus and a broad band through middle of corium and apical third of clavus. *Ventral surface*: Abdomen with numerous erect to semierect setae. *Legs*: Hind tibiae laterally flattened and weakly bowed and inner margin thickly set with long, erect setae.

*Male genitalia*: Aperture large, without marginal spines or processes. Left paramere (Fig. 227) elongate, with a large, apical, triangular, beak-like process truncated distally; a thickened, apically acute, recurved process at middle; and a long, slender, apically curved, basal process. Right paramere (Fig. 229): stout, thickest on basal half, without lateral arms or branches. Phallotheca (Fig. 228): uniformly slender, with a slender, recurved apical hook, and a short, decurved tubercle on opposite side.

*Female*: Unknown.

##### Distribution.

Known only from Cochise Co., Arizona.

##### Host.

Unknown.

##### Type specimens examined.

Holotype ♂: **USA:** **Arizona:** ***Cochise Co.***, Chiricahua Mts., 6200’, 20 June 1928, A. A. Nichol (00162218) (USNM). Paratypes: **USA:** **Arizona:** ***Cochise Co.*:** Chiricahua Mountains, East Turkey Creek, 31.90956EN, 109.25086EW, 1951 m, 26 Jul 1987, W. F. Barr, *Quercus* sp. (Fagaceae), 1♂ (00374336) (CNC). Huachuca Mountains, 31.502EN, 110.3994EW, 1839 m, 08 Jul 1905, H. G. Barber, 1♂ (00374335) (CNC).

##### Other specimens examined.

**USA:** **Arizona:** ***Cochise Co.*:** Chiricahua Mountains, SWRS, 5 mi. SW Portal, 31.88333EN, 109.20611EW, 1646 m, 22 Aug 2000, J.C. Schaffner, 1♂ (00285693) (USNM). Pinery Canyon, Chiricahua Mountains, 31.93514EN, 109.27482EW, 2073 m, 01 Jul 1919, A. Wetmore, 1♂ (00285692) (USNM). Huachuca Mts., Upper Carr Cyn., 7500 ft., 6–10 Aug 1952, H. B. Leech and J. B. Green, 3♂♂ (CAS). St. Rita Mt., 7-6-33, E. D. Ball, 1♂ (USNM). Southwestern Research Station (SWRS), 5 mi SW of Portal, 31.8825EN, 109.206EW, 5400’ (1646 m), 05 Sep 1965–25 Sep 1965, C. W. Sabrosky, Malaise trap, 1♂ (00285694) (USNM).

#### Pilophoropsidea fuscata

Taxon classificationAnimaliaHemipteraMiridae

Henry sp. n.

http://zoobank.org/6D0985EF-78D3-4A68-917A-761E9CADB75E

Figs 58 , 230–233

##### Diagnosis.

This species (Fig. 58) is recognized by the dark brown coloration; the smooth, highly polished pronotum; the male genitalia, particularly the left paramere (Figs 230, 231) with a wing-shaped median process and the C-shaped right paramere (Fig. 233) with a median spine; and the short, stout spine on the male genital aperture just above the left paramere.

##### Description.

*Male* (n = 2; holotype measurements in parentheses): Length 4.20 mm (3.84 mm), width 1.28 mm (1.24 mm). *Head*: Width 0.84 mm (0.82 mm), interocular width 0.44 mm (0.42 mm). *Labium*: Length ca. 1.50 mm [bent] (imbedded in glue). *Antenna*: Segment I, length 0.26 mm (0.28 mm); II, 0.92 mm (0.91 mm); III, 0.58 mm (0.54 mm); IV, 0.54 mm (0.56 mm). *Pronotum*: Length 0.84 mm (0.80 mm), basal width 1.10 mm (1.16 mm).

*Coloration*: *Head*: Shiny brown to reddish brown. *Antenna*: Segment I pale brown, with a red mark on basal third; segment II brown to fuscous; segments III and IV brown to fuscous. *Pronotum*: Brown to fuscous. *Scutellum*: Brown to fuscous. *Hemelytron*: Uniformly dark orange brown on basal half, dark brown to fuscous on apical half; membrane smoky brown to black, with a pale spot near apex of cuneus and pale at base between areoles. *Ventral surface*: Shiny brown, becoming more fuscous on abdomen. *Ostiolar evaporative area*: White, with enlarged anterior knob at end of scent channel dull red. *Legs*: Uniformly brown to reddish brown, tarsi slightly paler brown.

*Structure, texture, and vestiture*: *Head*: Frons finely transversely rugose, pubescence short, recumbent, with a few long, erect, pale setae along base of vertex and near basal area of clypeus. *Labium*: Extending to hind coxae. Antenna: Segment II slender, gradually enlarging to apex. *Pronotum*: Shiny, polished, disc smooth, calli granulate, weakly defined, collar transversely striate; pubescence short, recumbent, and very sparse. *Scutellum*: With silvery scale-like setae across apical half except apex, intermixed with long, erect, pale, simple setae. *Hemelytron*: Uniformly polished; with a narrow band of silvery scale-like setae across base of clavus and a wider one through middle of corium and across apical third of clavus, intermixed with a row of four long, erect, pale, simple setae on clavus and three or four on inner corial margin bordering membrane.

*Male genitalia*: Aperture with a distinct stout spine along margin just above left paramere. Left paramere (Figs 230, 231): Apex long and slender with a short, decurved, truncate apex; basal process stout, rounded apically; middle process wing-like, dorsally serrate arising from within base of V formed by basal and apical processes. Right paramere (Fig. 233): Somewhat stout, bulbous at middle, apical half forming a recurved C with a short spine arising from middle. Phallotheca (Fig. 232): Thickest basally, narrowing apically, with a stout, slightly curving, truncate apex.

*Female*: Unknown.

##### Etymology.

The specific epithet “*fuscata*”, is given to denote the overall dark brown to fuscous coloration.

##### Distribution.

Known from Arizona and New Mexico in the United States and Durango in Mexico.

##### Host.

Unknown.

##### Type material.

Holotype: ♂: **USA:** **Arizona:** ***Cochise Co.*:** Pinery Canyon, Chiricahua Mts., 4500 to 5000 ft., VIII–21–1932, D. K. Duncan, J. C. Lutz collection 1961 (00162219 (USNM). Paratypes: **MEXICO:** **Durango:** Durango, June 16, 1964, L. A. Kelton, 1♂ (CNC). **USA:** **Arizona** [***Cochise County***]: Chiricahua Mts., (Rustlers Pk.), VII–5–40, D. E. Hardy, 1♂ (KU). **New Mexico:** ***McKinley Co.***, Gallup, VII–14–1977, R. P. Allen, 1♂ (CDFA). ***Santa Fe Co.***, 6 mi. E. Tusuque, VII–4–84, J. T. Polhemus & D. A. Polhemus, 1♂ (JTP).

#### Pilophoropsidea keltoni

Taxon classificationAnimaliaHemipteraMiridae

Henry sp. n.

http://zoobank.org/D1EC94AA-3D3C-4D1F-A509-E8D176282475

Figs 59 , 234–236

##### Diagnosis.

This species (Fig. 59) is recognized by the large size; overall reddishbrown coloration; slender body form; the shiny, finely granulate pronotum; and male genitalia, particularly the left paramere (Fig. 234) with a long, slender, apically blunt basal process, and the phallotheca (Fig. 235) with an elongate apical process and deeply toothed subapical area.

##### Description.

*Male* (n = 5; holotype measurements in parentheses): Length 4.40–4.80 mm (4.80 mm), width 1.52–1.56 mm (1.54 mm). *Head*: Width 0.80–0.84 mm (0.83 mm), interocular width 0.26–0.28 mm (0.29 mm). *Labium*: Length 1.40–1.48 mm (1.52 mm). *Antenna*: Segment I, length 0.28–0.30 mm (0.27 mm); II, 0.96–1.04 mm (1.04 mm), slender, only slightly thickened to apex; III, 0.52–0.60 mm (0.56 mm); IV, 0.44–0.50 mm (0.50 mm). *Pronotum*: Length 0.84–0.86 mm (0.85 mm), basal width 1.28–1.32 mm (1.33 mm).

*Coloration*: *Head*: Brown to dark reddish brown. *Antenna*: Segment I pale brown, with a narrow, sometime indistinct, reddish line on inner surface; segments II–IV dark brown. *Pronotum*: Shiny reddish brown to dark brown. *Scutellum*: Dark brown. *Hemelytron*: Shiny orange brown on basal half, shiny reddish brown to dark brown apically; membrane dark smoky brown beyond apex of cuneus, pale or white basally across and between areoles. *Ventral surface*: Shiny reddish brown, becoming fuscous on abdomen; ostiolar area white with anterior knob dull red. *Legs*: Coxae whitish, apex of procoxa and bases of middle and hind coxae reddish to reddish brown; femora brown to reddish brown; tibiae brown to reddish brown, fore and middle tibiae paler apically.

*Structure, texture, and vestiture*: *Head*: Frons and vertex with transverse, finely granulate striations, with long, erect and semierect, pale, simple setae. *Labium*: Extending to bases of middle coxae. *Pronotum*: Disc shiny but with fine scattered granulations, calli and narrow anterior collar more densely granulate, simple pubescence sparse, recumbent. *Scutellum*: Depressed at middle, granulate, with a wide band of silvery scale-like setae across apical third except apex, intermixed with long, erect, pale, simple setae. *Hemelytron*: With a narrow band of silvery scale-like setae across base of clavus and a wider band through middle of corium and across apical third of clavus, intermixed on clavus with a few erect, pale, simple setae. *Ventral surface*: Mesosternum and abdomen with relatively long, semierect, simple setae. *Legs*: Entire length of hind tibial posterior face with two dense rows of short erect setae.

*Male genitalia*: Aperture. Left paramere (Fig. 234): Apical half stout, elongate, trunk-like with a flared, truncate apex; base stout with two thickened, apically rounded processes. Right paramere (Fig. 236): Mainstem thickened, apically rounded, with a short, thick, upturned lateral process at middle. Phallotheca (Fig. 235): Stout on basal half, narrowing apically into a slender, recurved hook and a deeply toothed, subapical area.

*Female*: Unknown.

##### Etymology.

This species is named in honor of the late mirid specialist Dr. Leonard A. Kelton (retired from the Biosystematics Research Institute, Agriculture Canada, Ottawa), friend, colleague, and collector of all known specimens of this species, as well as numerous other specimens used in this study.

##### Distribution.

Durango, Mexico.

##### Host.

One of the type specimens was taken from mistletoe growing on pine.

##### Type material.

Holotype ♂: **MEXICO:** ***Durango*:** 3 mi. E El Salto, 8400’, 21 June 1964, L. A. Kelton (00167382 )(CNC). Paratypes: **MEXICO:** **Durango:** 3 mi. E El Salto, 8400’, 21 June 1964, L. A. Kelton, 1♂ (00167385) (CNC). 24 mi. W. La Ciudad, Durango, 7000’, 20 June 1964, L. A. Kelton, 2♂♂ (00167384) (CNC), (0016220) (USNM). 10 mi. W. El Salto, Durango, 9000’, 30 June 1964, L. A. Kelton, on pine and mistletoe, 1♂ (00167383) (CNC).

#### Pilophoropsidea maxima

Taxon classificationAnimaliaHemipteraMiridae

Henry sp. n.

http://zoobank.org/69CE0D30-5A5E-4839-83CF-909B368C2860

Figs 60 , 237–239

##### Diagnosis.

This species (Fig. 60) is recognized by the large size; dark brown to reddishbrown coloration; shiny, finely granulate pronotum; and male genitalia, particularly the stout left paramere (Fig. 237) with two apically acute basal spines and a short, truncate, decurved apical process and the phallotheca (Fig. 238) with a slender, recurved apical process.

##### Description.

*Male* (n=3; holotype measurements in parentheses): Length 5.03–5.17 mm (4.99 mm), width 1.36–1.44 mm(1.36 mm). *Head*: Width 0.82-0.84 mm (0.80 mm), interocular width 0.28-0.30 mm (0.29 mm). *Labium*: Length 1.50–1.52 mm (1.49 mm). *Antenna*: Segment I, length 0.28–0.30 mm (0.27 mm); II, 0.94–0.96 mm (0.96 mm); III, 0.52–0.54 mm (0.54 mm); IV, 0.48–0.50 mm (0.50 mm). *Pronotum*: Length 0.88–0.90 mm (0.85 mm), basal width 1.31–1.32 mm (1.31 mm).

*Coloration*: *Head*: Dark brown to fuscous. *Antenna*: Segment I pale brown, with a broad stripe extending entire length on inner side; segments II–IV dark brown. *Pronotum*: Dark brown to fuscous. *Scutellum*: Dark brown to fuscous. *Hemelytron*: Orange brown on basal half, dark brown or fuscous on apical half; membrane mostly smoky dark brown; pale or white through and between areoles. *Ventral surface*: Brown to reddish brown, more fuscous on abdomen. *Ostiolar evaporative area*: White, with anterior knob at end of scent channel dull red. *Legs*: Procoxa pale brown to brown, reddish brown apically, middle and hind coxae white, brown at bases; femora uniformly brown to reddish brown; tibiae brown to reddish brown; tarsi pale brown; claws brown.

*Structure, texture, and vestiture*: *Head*: Shiny, frons with fine transverse granulations. *Labium*: Extending to bases of middle coxae. *Antenna*: Segment II slender, gradually thickened to apex. *Pronotum*: Shiny, disc finely and sparsely granulate, entire surface of calli and collar granulate; pubescence short, sparse, and recumbent. *Scutellum*: Transversely rugose, with a band of silvery scale-like setae across apical half, intermixed with long, erect, pale, simple setae. *Hemelytron*: Uniformly polished; with a band of silvery scale-like setae across base of clavus and a wider band through middle of corium and apical third of clavus, intermixed with a short row of long, erect, pale, simple setae on apical half of clavus and a few along inner corial margin adjacent to membrane. *Legs*: Hind tibiae somewhat flattened, with entire length of inner side having two dense rows of long (subequal to diameter of segment), erect setae.

*Male genitalia*: Aperture with two short spines above left paramere, one simple and acute, one apically trispinose. Left paramere (Fig. 237) short, stout, enlarged apical half with a forward-curving crest and a recurved, truncate, apical process; base with two variable processes, longest one thickened apically, rounded, shorter one apically acute. Right paramere (Fig. 239) relatively slender, with a short, apically blunt process curved upward. Phallotheca (Fig. 238) slender, with a long, recurved apical hook and a subapical notch.

*Female*: Unknown.

##### Etymology.

The specific epithet *maxima* is used to denote the large size of this species.

##### Distribution.

Durango, Mexico.

##### Host.

Unknown.

##### Type material.

Holotype: ♂: **MEXICO:** **Durango:** 10 mi. W El Salto, 9000’, 10-VIII-1964, L. A. Kelton (00167382) (CNC). Paratypes: **MEXICO:** **Durango:** 9 mi. W La Ciudad, 9000’, 1 July 1964, L. A. Kelton, 1♂ (00167386) (CNC). 24 mi. W La Ciudad, 7000’, 21 July 1964, L. A. Kelton, 1♂ (0016221) (USNM).

#### Pilophoropsidea pueblaensis

Taxon classificationAnimaliaHemipteraMiridae

Henry sp. n.

http://zoobank.org/4BD2B524-4507-4ED1-9B56-1F33BDB9CCA0

Figs 61 , 240–242

##### Diagnosis.

This species (Fig. 61) is recognized by the dark brown to fuscous (almost black) coloration; shiny, finely granulate pronotum; and the male genitalia, particularly the simple, apically truncate right paramere (Fig. 242) and phallotheca (Fig. 241).

##### Description.

*Male* (n = 2; holotype measurements in parentheses): Length 4.54 mm (4.54 mm), width 1.32 mm (1.30 mm). *Head*: Width 0.84 mm (0.83 mm), interocular width 0.32 mm (0.34 mm). *Labium*: Length 1.40 mm (1.36 mm). *Antenna*: Segment I, length 0.28 mm (0.30 mm); II, 0.94 mm (0.93 mm); III, 0.48 mm (0.53 mm); IV, 0.50 mm (0.48 mm). *Pronotum*: Length 0.86 mm (0.86 mm), basal width 1.30 mm (1.28 mm).

*Coloration*: *Head*: Dark brown. *Antenna*: Segment I pale brown, with a dark reddish brown line extending entire length on inner side; segment II black, slightly paler dark brown near base; segments III and IV black. *Pronotum*: Fuscous. *Hemelytron*: Fuscous, slightly paler dark reddish brown on basal half; membrane dark smoky brown, paler inside areoles. *Ventral surface*: Shiny brown, abdomen becoming fuscous. *Ostiolar evaporative area*: White, with knob at end of scent channel dull brown. *Legs*: Procoxa brown, middle and hind coxae pale or white with bases fuscous; femora uniformly dark brown, hind femur nearly black with extreme apex pale; tibiae dark reddish brown, hind tibia slender; tarsi and claws brown.

*Structure, texture, and vestiture*: *Head*: Granulate, frons transversely rugose. *Labium*: Extending just to bases of middle coxae. *Pronotum*: Semishiny, finely granulate, pubescence short, sparse, and recumbent; scutellum granulate, with a band of silvery scale-like setae through middle, intermixed with long, erect, simple setae. *Hemelytron*: Polished, smooth, with two bands of silvery scale-like setae, a narrow one across base of clavus and a broader one through middle of corium and across apical third of clavus, intermixed with a row of erect, simple setae through middle of clavus.

*Male genitalia*: Aperture unarmed except for a minute spine just above left paramere. Left paramere (Fig. 240): Apical half enlarged with apex extended and slender, basal half with two processes, the basal one short and acute, the middle one longer, rounded, and apically rounded, acute. Right paramere (Fig. 242): Stout, narrow toward truncate apex, without arms. Phallotheca (Fig. 241): Slender, apically truncate.

*Female*: Unknown.

##### Etymology.

This species is named after the Mexican state of Puebla, from which it was collected.

##### Distribution.

Puebla, Mexico.

##### Host.

Unknown.

##### Type material.

Holotype ♂: **MEXICO:** **Puebla:** Apulco, 13 mi. N. Zacapoaxtla, July 23, 1985, Jones and Schaffner, taken at light (00167706) (TAMU). Paratype: **MEXICO:** **Puebla:** 4.7 mi. SW La Cumbre, elev. 5100’, VII–23–1987, Woolley & Zolnerowich, 87/055, 1♂ (00162222) (USNM).

#### Pilophoropsidea schaffneri

Taxon classificationAnimaliaHemipteraMiridae

Henry sp. n.

http://zoobank.org/D3543ADD-B3B0-4522-B3AF-18E746A8BF7A

Figs 62–65 , 243–245

##### Diagnosis.

This species (Figs 62–65) is recognized by the large size; overall dark brown to fuscous coloration; shiny, finely granulate, fuscous pronotum; and male genitalia, particularly the left paramere (Fig. 243) with a quadrate dorsal process and the stout, C-shaped right paramere (Fig. 245).

##### Description.

*Male* (n = 2; holotype measurements in parentheses): Length 4.48 mm (4.68 mm), width 1.28 mm (1.30 mm). *Head*: Width 0.91 mm (0.85 mm), interocular width 0.26 (0.27 mm). *Labium*: Length 1.54 mm (1.44 mm). *Antenna*: Segment I, length 0.30 mm (0.30 mm); II, 1.02 mm (0.91 mm); III, 0.59 mm (0.53 mm); IV 0.59 mm (0.53 mm). *Pronotum*: Length 0.83 mm (0.82 mm), basal width 1.23 mm (1.20 mm).

*Coloration*: *Head*: Fuscous. *Antenna*: Segment I pale brown, slightly infuscated at base, red mark frequent in other species absent; segment II fuscous, slightly paler at base; segments II and III fuscous. *Pronotum*: Dark brown to fuscous. *Scutellum*: Fuscous. *Hemelytron*: Dark brown to fuscous, apical half slightly darker; membrane smoky black, paler through areoles and basal half of area between. *Ventral surface*: Shiny dark brown, abdomen fuscous or black. *Ostiolar evaporative area*: Uniformly white (one paratype with a tiny red dot at base of auricle). *Legs*: Procoxa brown, middle and hind coxae white to pale brown with fuscous bases; femora uniformly dark brown; tibiae dark brown, fore and middle tibiae paler apically.

*Structure, texture, and vestiture*: Head: Granulate, with a dull sheen; frons weakly transversely striate. *Labium*: Extending to bases of middle coxae. *Pronotum*: Shiny, finely granulate, collar with weak transverse striations, pubescence sparse and very short. *Scutellum*: Transversely rugose except for smooth apex, with a band of silvery scale-like setae across apical half except apex, intermixed with a few long, erect, pale, simple setae. *Hemelytron*: Smooth, polished, with two bands of silvery scale-like setae, a narrow one across base of clavus and a broader one through middle of corium and across apical third of clavus, intermixed on apical half of clavus and inner corial margin bordering membrane with long, erect, pale, simple setae. *Legs*: Hind tibiae straight, slender, diameter subequal to that of fore and middle tibiae, lacking dense row of erect setae along inner margin.

*Male genitalia*: Aperture above left paramere with a slender, dorsally directed spine. Left paramere (Fig. 243) relatively stout, apex slender, curved downward, with a low, quadrate dorsal flange on apical third and a large, stout, apically acute process on basal half. Right paramere (Fig. 245) C-shaped. Phallotheca (Fig. 244) relatively stout, with a slender, decurved apical process.

*Female* (n = 1): Length to apex of abdomen 3.12 mm, length to apex of cuneus 2.48 mm, width 1.24 mm. *Head*: Width 0.88 mm, interocular width 0.48 mm. *Labium*: Length 1.50 mm. *Antenna*: Segment I, length 0.28 mm; II, 0.86 mm; III, 0.50 mm; IV 0.48 mm. *Pronotum*: Length 0.76 mm, basal width 0.74 mm. Similar to male in coloration.

*Structure, texture, and vestiture*: *Labium*: Extending to base of abdomen. *Pronotum*: More quadrate than in males, lateral margins subparallel, basal angles weakly flared. *Hemelytron*: Brachypterous, deeply depressed basally, abruptly lifted upward over abdomen beyond apex of scutellum, claval and cuneal sutures and membrane absent, apical four abdominal segments exposed beyond apices, apical half of corium tumid or with a glabrous hump; pubescence similar to that of male except tumid area surrounded by a semicircular band (modified broad band of males) of silvery scale-like setae beginning at middle of outer corial margin, continuing around toward clavus and across apex to outer margins of cuneus. Ventral surface and legs similar to those of male.

##### Etymology.

This species named in honor of mirid specialist Dr. Joseph C. Schaffner, friend, colleague, and collector of all the known specimens of this species, as well as those for six other new species described in this paper. His collections of Mexican Miridae are unsurpassed.

##### Distribution.

Nuevo León and San Luis Potosi, Mexico.

##### Host.

Unknown.

##### Type material.

Holotype: ♂: **MEXICO:** **San Luis Potosi:** 28.5 mi. S. Huizache, July 4, 1985, Jones and Schaffner (00167507) (TAMU). Paratypes: **MEXICO:** **San Luis Potosi:** 28.5 mi. S. Huizache, July 4, 1985, Jones and Schaffner, 1♂, 1♀ (00167508-00167509) (TAMU). **Nuevo León:** 8 miles south of La Escondida, July 24, 1976, Piegler, Gruetzmacher, R. & M. Murray, and J. C. Schaffner, 1♂ (00162223) (USNM).

#### Pilophoropsidea serrata

Taxon classificationAnimaliaHemipteraMiridae

Henry sp. n.

http://zoobank.org/BDD0CAA4-2ED8-47D0-A510-0E9737CFCD83

Figs 66 , 246–249

##### Diagnosis.

This species (Fig. 66) is recognized by the large size; overall dark coloration; shiny, dark brown pronotum; male genitalia, particularly the left paramere (Figs 246, 247) with a long, slender, apically hooked basal process and a stouter, apically acute median process, and the phallotheca (Fig. 248) with a short hooked apical process and dorsal flap; and the 12 to 13 short, setigerous tubercles on the dorsal edge of the male genital aperture.

##### Description.

*Holotype male*: Length 5.33 mm, width 1.40 mm. *Head*: Width 0.84 mm, interocular width 0.36 mm. *Labium*: Length 1.60 mm. *Antenna*: Segment I, length 0.30 mm; II, 1.00 mm; III, 0.56 mm; IV, 0.54 mm. *Pronotum*: Length 0.90 mm, basal width 1.40 mm.

*Coloration*: *Head*: Dark reddish brown. *Antenna*: Segment I pale brown, with a dark brown streak extending entire length on inner side; segment II dark brown or fuscous; segments II and III fuscous. *Pronotum*: Fuscous, somewhat paler red brown across calli. Scutellum: Reddish brown. *Hemelytron*: Dark brown to fuscous on apical half, more pale reddish to orange brown on basal half; membrane smoky black, paler at base across proximal half of large areole. *Ventral surface*: Shiny dark reddish brown. *Ostiolar evaporative area*: White, with knob at end of scent channel dull brown. *Legs*: Fore coxa brown, middle and hind coxae pale or whitish with bases infuscated; femora uniformly dark brown; tibiae uniformly dark brown.

*Structure, texture, and vestiture*: *Head*: Shiny, granulate, frons transversely rugose, an elongate, glabrous, smooth, shiny spot present on either side of vertex, with each touching inner margin of an eye. *Labium*: Extending to bases of middle coxae. *Pronotum*: Shiny, surface of disc finely granulate, calli and collar uniformly granulate; recumbent pubescence short and sparse. *Scutellum*: Transversely rugose except for smooth apex, with silvery scale-like setae across middle, intermixed with long, erect, pale, simple setae. *Hemelytra*: Polished, smooth, with two bands of silvery scale-like setae, a narrow one across base of clavus and a broader one through middle of corium and across apical third of clavus.

*Male genitalia*: Dorsal edge of aperture with 12 to 13 tiny setigerous tubercles giving a finely serrate appearance, with a slightly larger spine on left side before serrations. Left paramere (Figs 246, 247) relatively elongate, apical part extended and truncate, basal half with two erect, stout processes, the shortest having a small subapical tubercle. Right paramere (Fig. 249) stout, more slender on apical third, middle with a small quadrate flange. Phallotheca (Fig. 248) relatively stout, apically hooked, with a small, recurved flap.

*Female*: Unknown.

##### Etymology.

The specific epithet *serrata* is used to denote the finely serrate dorsal edge of the male genital aperture.

##### Distribution.

Michoacan, Mexico.

##### Host.

Collected on *Pinus* sp. [Pinaceae].

##### Type material.

Holotype ♂: **MEXICO:** **Michoacan:** 6 mi. N Cheran, July 7–8, 1985, Jones and Schaffner, from pine (00167510) (TAMU).

#### Pilophoropsidea touchetae

Taxon classificationAnimaliaHemipteraMiridae

Henry sp. n.

http://zoobank.org/ED841EA7-B7D6-41CD-8700-04AD2E1CECDB

Figs 67 , 250–253

##### Diagnosis.

This species (Fig. 67) is recognized by the fuscous head and hemelytra, the dull black, granulate pronotum; and male genitalia, particularly the left paramere (Figs 250, 251) with a long, stout basal process and short lateral process (visible anteriorly only) and the right paramere (Fig. 253) with a slender, apically crenulate, lateral arm.

##### Description.

*Holotype male*: Length 3.78 mm, width 1.24 mm. *Head*: Width 0.81 mm, interocular width 0.43 mm. *Labium*: Length 1.52 mm. *Antenna*: Segment I, length 0.29; II, 0.95 mm; III, 0.52 mm; IV 0.47 mm. *Pronotum*: Length 0.91 mm, basal width 1.26 mm.

*Coloration*: *Head*: Dark reddish brown. *Antenna*: Segment I yellowish brown; segment II yellowish brown, becoming darker reddish brown on apical third; segments III and IV dark reddish brown. *Pronotum*: Black. *Scutellum*: Fuscous, paler brown apically. *Hemelytron*: Basal third of clavus fuscous or nearly black, remainder of hemelytron, including membrane, dark brown. *Ventral surface*: Shiny dark reddish brown. *Ostiolar evaporative area*: White, with knob at middle of scent channel dull red. *Legs*: Fore coxae and trochanters dark brown, middle and hind coxae and trochanters white; femora, tibiae, tarsi, and claws uniformly dark brown.

*Structure, texture, and vestiture*: *Head*: Finely granulate, satiny, clothed with short, simple recumbent setae, intermixed with longer, more erect, simple setae on frons and clypeus. *Labium*: Extending to middle coxae. *Pronotum*: Finely granulate, surface luster dull or at most satiny; mesoscutum hidden by base of pronotum. *Scutellum*: With 8 or more long, scattered, erect setae and a wide field of silvery, scale-like setae through middle. *Hemelytron*: Clavus, basal half of embolium, and corium dull to satiny, cuneus, apical half of embolium, and corium shiny; with two transverse fields of silvery, scale-like setae, one across base of clavus and one across middle of corium and apex of clavus, intermixed with a row of five or more long, erect, simple setae along middle of clavus and a row on corium bordering membrane.

*Male genitalia*: Left paramere (Figs 250, 251) trifurcate, with a long apical process, a shorter dorsal process, and one short process extending anteriorly and hidden in caudal aspect (i.e., in situ). Right paremere (Fig. 253) with a slender, curved lateral arm with a marginally crenulate apex and a tiny subapical process. Phallotheca (Fig. 252) stout, with a short, curved apical process.

*Female*: Unknown.

##### Etymology.

This species is named in honor of Michele A. Touchet (Systematic Entomology Laboratory, ARS, USDA, Washington, D. C.) for her enthusiastic and professional assistance, including her time-consuming specimen data entry and providing all color habitus photographs in this paper.

##### Distribution.

Known only from the Mexican state of Puebla.

##### Host.

Unknown.

##### Type material.

Holotype ♂: **MEXICO:** **Puebla:** 5.8 miles northwest Telixtlahuaca, July 21, 1987, Kovarik, Schaffner (00167511) (TAMU).

#### Pilophoropsidea truncata

Taxon classificationAnimaliaHemipteraMiridae

Henry sp. n.

http://zoobank.org/56CE627A-72C0-4460-BC02-7D44A61F3931

Figs 68 , 69 , 254–256

##### Diagnosis.

This species (Figs 68, 69) is recognized by the overall fuscous coloration; black, granulate pronotum; uniformly white ostiolar auricle; male genitalia, particularly the basally globose and apically slender left paramere (Fig. 254) and quadrate right paramere (Fig. 256) with a serrate dorsal ridge; and the broad, truncate process on the male genital aperture.

##### Description.

*Holotype male*: Length 4.48 mm, width 1.48 mm. *Head*: Width 0.96 mm, interocular width 0.38 mm. *Labium*: Length 1.50 mm. *Antenna*: Segment I, length 0.32 mm; II, 0.96 mm; III 0.52 mm; IV, 0.46 mm. *Pronotum*: Length 0.96 mm, basal width 1.26 mm.

*Coloration*: *Head*: Fuscous to black. *Antenna*: Segment I pale brown, inner side with a dark brown mark extending entire length; segment II fuscous to black; segments III and IV black. *Pronotum*: Black. *Scutellum*: Fuscous, transversely rugose, covered with silvery scale-like setae except apex, intermixed with long, erect, simple setae. *Hemelytron*: Uniformly fuscous; membrane fumate or black. *Ventral surface*: Fuscous. *Ostiolar evaporative area*: Uniformly white. *Legs*: Fore coxa brown, middle and hind coxae white and narrowly infuscated at bases; tibiae fuscous, hind tibia slender.

*Structure, texture, and vestiture*: *Head*: Weakly shining, granulate, frons transversely rugose, with a shiny spot on either side of vertex adjacent to eye. *Labium*: Extending to middle coxae. *Pronotum*: Dull to semishiny, strongly granulate, collar transversely rugose; pubescence short, sparse, and recumbent. *Hemelytron*: Polished, smooth, with two bands of silvery scale-like setae, a narrow one across base of clavus and continuing onto scutellum and a broader one through middle of corium and across apical third of clavus; also with a few scattered scales on clavus between bands, intermixed with a row of long, erect, simple setae through middle of clavus and a few along inner corial margin near base of membrane.

*Male genitalia*: Aperture with a short, wide, truncate process on dorsal edge just right of center (position about 1:00); dorsal edge also with numerous long, inwardly directed setae. Left paramere (Fig. 254): Simple, most thickened at base, apex extended into a long, slender, decurved process. Right paramere (Fig. 256): Stout, broad, with a thick, wide process arising from each side. Phallotheca (Fig. 255): Long, stout, with a short, pointed apical tubercle.

*Female*: Unknown.

##### Etymology.

The specific epithet “*truncata*” is given to denote the truncate tergal process on the male aperture.

##### Distribution.

Guerrero, Mexico

##### Host.

Unknown.

##### Type material.

Holotype ♂: **MEXICO:** **Guerrero:** 6.2 mi. SW Xochipala, elev. 5670 ft., July 6, 1987, Kovarik and Schaffner (00167512) (TAMU).

#### Pilophoropsidea tuberculata

Taxon classificationAnimaliaHemipteraMiridae

Henry sp. n.

http://zoobank.org/40A38645-37DF-4655-8985-3FA98C73D3B2

Figs 70–71 , 72 , 257–259

##### Diagnosis.

This species (Figs 70–72) is recognized by the overall fuscous coloration; dull black, granulate pronotum; male genitalia, particularly the simple, C-shaped left paramere (Fig. 257), the right paramere (Fig. 259) with a stout, apically acute lateral arm, and the long, slender phallotheca (Fig. 258); and the large, stout spine on male genital aperture above the right paramere.

##### Description.

*Male* (n = 3; holotype measurements in parentheses): Length 3.80–4.20 mm (4.16 mm), width 1.20–1.22 mm (1.20 mm). *Head*: Width 0.74–0.78 mm (0.78 mm), interocular width 0.38–0.40 mm (0.40 mm). *Labium*: Length 1.39–1.44 mm (1.39 mm). *Antenna*: Segment I, length 0.29–0.32 mm (0.29 mm); II, 0.90–1.02 (0.90 mm); III, 0.50–0.52 mm (0.51 mm); IV, 0.42–0.45 mm (0.45 mm). *Pronotum*: Length 0.92–0.99 mm (0.99 mm), basal width 1.20–1.28 mm (1.28 mm).

*Coloration*: *Head*: Fuscous to black. *Antenna*: Segment I pale brown, with a small red mark inside near base; segment II black; segments III and IV fuscous. *Pronotum*: Black. *Scutellum*: Fuscous to dull black, paler apically. *Hemelytron*: Uniformly black; membrane uniformly dark smoky brown, narrowly shiny around base bordering cuneus. *Ventral surface*: Uniformly shiny reddish brown to black. *Ostiolar evaporative area*: White, with knob at end of scent channel dull red. *Legs*: Fore coxa dark reddish brown to black, middle and hind coxae pale or whitish, narrowly fuscous at bases; femora uniformly dark reddish brown to black; tibiae reddish brown to black; tarsi and claws brown to fuscous.

*Structure, texture, and vestiture*: *Head*: Uniformly granulate, weakly shiny. *Labium*: Extending to apices of middle coxae or bases of hind coxae. *Antenna*: Segment II gradually thickened to apex, apical width greater than diameter of segment I. *Pronotum*: Dull, strongly granulate, collar transversely striate, with scattered recumbent setae. *Scutellum*: Granulate, apex smooth and shiny, with a wide band of silvery scale-like setae across apical half, except shiny apex. *Hemelytron*: Rugose or roughened, shiny but not polished, with two bands of silvery scale-like setae, a narrow one across base of clavus and a broader one through middle of corium and apical third of clavus; thickly set with short, recumbent, pale, simple setae and a few longer, erect setae on clavus and inner corial margin bordering membrane. *Legs*: Hind tibia strongly flattened and bowed, broad side nearly two times diameter of fore tibia, inner side with two rows of long (0.04–0.06 mm), erect setae.

*Male genitalia*: Right side of aperture with a large stout spine or tubercle. Left paramere (Fig. 257) simple, slender, without flanges or basal processes. Right paramere (Fig. 259) somewhat C-shaped, apex blunt, with a stout, apically pointed, lateral process arising at middle. Phallotheca (Fig. 258) relatively stout, curving, apex blunt with a short spine on one side.

*Female* (n = 2): Length to apex of abdomen 3.04–3.14 mm, length to apex of cuneus 2.48–2.60 mm, width 1.30–1.32 mm. *Head*: Width 0.78–0.80 mm, interocular width 0.46–0.50 mm. *Labium*: Length 1.44–1.58 mm. *Antenna*: Segment I, length 0.30–0.32 mm; II, 0.92–1.02 mm; III, 0.52–0.60 mm; IV, 0.46–0.56 mm. *Pronotum*: Length 0.74–0.76 mm, basal width 0.76–0.80 mm. Similar to male in coloration, differing structurally as noted below.

*Structure, texture, and vestiture*: *Labium*: Extending past hind coxae to base of abdomen. *Pronotum*: Much more quadrate than in males, lateral margins subparallel, humeral angles weakly flared. *Scutellum*: Basal half sloping downward posteriorly, apical half flattened, level with hemelytra. *Hemelytron*: Brachypterous; coloration and surface texture much as in males; claval and cuneal sutures and membrane absent, apex of cuneus cut inward, forming a deep apical V; last four or five abdominal segments exposed beyond hemelytra. Ventral surface and legs much as in males, including flattened and bowed hind tibiae.

##### Etymology.

The specific epithet “tuberculata” denotes the large, stout tubercle on the right side of the male genital aperture.

##### Distribution.

Guerrero, Mexico.

##### Host.

Unknown.

##### Type material.

Holotype ♂, **MEXICO:** **Guerrero:** 6.2 mi. SW Xochipala, elev. 5670 ft., July 6, 1987, Kovarik and Schaffner (00167513) (TAMU). Paratypes: **MEXICO:** ***Guerrero*:** 6.2 mi. SW Xochipala, elev. 5670 ft., July 6, 1987, Kovarik and Schaffner, 2♂♂ (1 damaged), 2♀♀ ((00167514) 1♂, (00167515) 1♀ TAMU; (00162224) 1♂, (00162225) 1♀ USNM).

#### Pilophoropsis

Taxon classificationAnimaliaHemipteraMiridae

Poppius

Pilophoropsis Poppius, 1914: 249 (orig. descrip.); Carvalho 1952: 82 (cat.); 1955a: 80 (key); Carvalho 1955b: 227 (note); 1958: 141 (cat.); Knight 1968: 158 (key); Carvalho et al. 1983: 3 (note); Henry and Wheeler 1988: 399 (cat.); Henry 1994: 702 (note); Schuh 1995: 181 (cat.). Type species: *Pilophoropsis brachyptera* Poppius, 1914. Original designation.
Renodaella Knight, 1927: 306 (orig. descrip.); Carvalho 1952: 83 (cat.); 1955b: 227 (note, syn.). Synonymized by Carvalho 1955b: 227. Type species: *Renodaella nicholi* Knight, 1927. Original designation.

##### Diagnosis.

Characterized by the recessed labial segment I that does not extend beyond the gular sulcus (Fig. 121); the shiny, strongly convex pronotum that narrows anteriorly and has the lateral margins sulcate, with the disc and calli evenly rounded; the mostly dull or satiny hemelytron having only the cuneus, embolium, and basal area of the membrane polished, distinct bands and patches of tightly arranged, silvery scale-like setae, and stout, erect, black, bristle-like setae on the clavus and corium. Males are fully macropterous. Females are always brachypterous, with the apex of the cuneus and membrane greatly abbreviated, and the hemelytral setal pattern modified; and the pronotum is more quadrate, with the convexity of disc sulcate through the middle.

##### Description.

Myrmecomorphic. Males macropterous; females brachypterous. Length of males 2.80–3.52 mm; length of females 2.36–3.12 mm. Head broader than long; posterior margin truncate, distinctly carinate, posterior margins of eyes level with base of vertex; eyes large, elongate oval, occupying more than half of dorsal head width, laterally occupying nearly three fourths of height; front broadly rounded at level from eye to eye, clypeus moderately acute, partially visible from dorsal aspect; segment I of labium arising from and completely enclosed within oval gular sulcus, segment not or hardly visible below buccula in lateral aspect; labium extending to middle or hind coxae. Antenna with segment I shortest, II longest, most slender on basal half, gradually enlarging to apex that is subequal to diameter of segment I, sometimes swollen or clavate apically; segments III and IV thickest, usually fusiform, III sometimes more slender on basal half. Pronotum trapeziform, lateral margins weakly sulcate, narrowing anteriorly to obscure narrow, transverse, collar-like area, posterior angles often broadly flared, posterior margin weakly rounded; mesoscutum covered by base of pronotum, scutellum equilateral, base sometimes covered by base of pronotum. Hemelytron dull or satiny, with cuneus, embolium, and basal area of membrane polished, lateral margins shallowly constricted between bases of cuneus and corium; with distinct patches and bands of tightly arranged silvery scale-like setae; intermixed on clavus and corium with stout, erect, black, bristle-like setae. Cuneus and membrane fully developed in males; claval suture absent and apex of membrane at that level across cuneus usually abbreviated in females, exposing apical 3 or 4 abdominal segments. Ventral surface shiny; ostiolar area white, without raised knob at end of scent channel; second visible abdominal segment with a dull or glaucous, quadrate patch ventrally. Legs unmodified; parempodia fleshy, convergent apically. Male aperture large, open, unarmed; generalized left paramere elongate, with a subtriangular, beak-like, apical process, with variable processes arising basally to about the middle of the main trunk; right paramere roughly C-shaped, main stem stout, with a large recurving, sometimes bifurcate, lateral arm; phallotheca generally slender, with a distinct apical hook; endosoma unmodified.

##### Etymology.

I follow Steyskal (1973), who considered the suffix “opsis” feminine.

##### Discussion.

Prior to this study, only three species of *Pilophoropsis* were recognized. Strong sexual dimorphism in this genus makes it difficult to associate males and females when collected separately. As a consequence, the male of *Pilophoropsis brachyptera* Poppius (1914), previously known only from the brachypterous female, was described by Knight (1968) as *Pilophoropsis balli*. Polhemus and Polhemus (1985), however, showed that these two species are synonyms based on a series of males, females, and nymphs they collected together in Arizona, thus emphasizing the importance of male genitalic characters for separating species in the Ceratocapsini. Females can be identified only by their association with males at this time.

The following key relies primarily on male genitalic structures, nearly all of which may be viewed caudally without dissecting specimens.

##### Key to the males of *Pilophoropsis*

**Table:** 

1	Posterior band of silvery, scale-like setae on corium bordering cuneal fracture; lateral arm of right paramere flared apically (Figs 268, 279)	**2**
–	Posterior band of silvery, scale-like setae level with apex of clavus, never bordering cuneal fracture; lateral arm(s) of right paramere not flared at apex	**3**
2	Left paramere with a basally stout middle process (Fig. 266) extending outward into along slender, thread-like apex; main trunk of right paramere (Fig. 268) lacking a short spine, apex of lateral arm T-shaped; phallotheca (Fig. 267) with a relatively long, slender, recurved, apical process; Mexico (Oaxaca)	***cunealis* sp. n.**
–	Left paramere with only smaller, relatively thick basal process (Fig. 276) extending upward into a more thickened apex; main trunk of right paramere (Figs 278, 279) with two short, stout spines, lateral arm broad, bifid or V-shaped apically; phallotheca (Fig. 277) with a stout, decurved apical process; Mexico (Coahuila) and United States (Texas)	***texana* (Knight)**
3	Right paramere (Figs 262, 265) with lateral arm simple apically	**4**
–	Right paramere (Figs 271, 274) with lateral arm divided or bifurcate apically	**5**
4	Apical process of phallotheca (Fig. 261) short and decurved; middle of left paramere (Fig. 260) with two processes, one slender and curving outward and one longer, stouter, apically curved, and with a stout spine at base; Mexico (Sonora)	***bejeanae* sp. n.**
–	Apical process of phallotheca (Fig. 264) long and decurved; middle base of left paramere (Fig. 263) with three subequal processes forming a comb-like shape; United States (Arizona)	***brachyptera* Poppius**
5	Apex of phallotheca (Fig. 273) beak-like; right paramere (Figs 274, 275 )with apex of lateral arm bifurcate or V-shaped; United States (Arizona)	***quercicola* sp. n.**
–	Apex of phallotheca (Fig. 270) slender and decurved; right paramere (Fig. 271) with apex of lateral arm separated into widely diverging processes; United States (Arizona)	***nicholi* (Knight)**

#### Pilophoropsis bejeanae

Taxon classificationAnimaliaHemipteraMiridae

Henry sp. n.

http://zoobank.org/515BB5B4-CC51-497B-8B96-A1C76449DC96

Figs 73 , 260–262

##### Diagnosis.

This species (Fig. 73) is distinguished by the small size and the male genitalia, particularly the left paramere (Fig. 260) with two middle processes, the sharply C-shaped right paramere (Fig. 262) similar to that of *Pilophoropsis brachyptera*, and the short, stout, decurved apical process of the phallotheca (Fig. 261).

##### Description.

*Male* (n = 2; holotype measurements in parentheses): Length 3.08 mm (2.80 mm), width 0.96 mm (0.94 mm). *Head*: Width 0.66 mm (0.64 mm), interocular width 0.30 mm (0.28 mm). *Labium*: Length 0.86 mm (0.82 mm). *Antenna*: Segment I, length 0.20 mm (0.18 mm); II, 0.58 mm (0.54 mm); III, 0.34 mm (0.32 mm); IV, 0.34 mm (0.30 mm). *Pronotum*: Length 0.62 mm (0.60 mm), basal width 0.90 mm (0.88 mm).

*Coloration*: *Head*: Pale reddish brown to fuscous, holotype paler, with small red marks on vertex, frons, clypeus, and bucculae. *Antenna*: Segment I pale brown, with two red marks, one across basal third extending distally and ending before apex and one short mark on opposite side of apical third; segment II slender, gradually enlarging apically, brown, paler on apical half to apical two thirds, pubescence short, pale, recumbent; segments III and IVreddish brown. *Pronotum*: Reddish brown to fuscous. *Scutellum*: Reddish brown to fuscous. *Hemelytron*: Pale brown, darker brown on outer two thirds of clavus and apical third of corium, cuneus red to reddish brown; membrane brown. *Ventral surface*: Reddish brown to fuscous. *Ostiolar evaporative area*: Uniformly white. *Legs*: Coxae whitish, bases of middle and hind coxae and apex of fore coxae reddish brown; femora brown, basal half of middle femur pale, base of remaining hind femur obscured by body and glue; tibiae brown to reddish brown, pale on apical half; tarsi and claws pale brown.

*Structure, texture, and vestiture*: *Head*: Shiny, frons weakly rugose. *Labium*: Extending to middle coxae. *Pronotum*: Anterior third narrowed nearly half as wide as base, convex, disc strongly shining, calli granulate, depressed and weakly punctate between, flattened anterior collar-like area seen in other species indistinct or absent; with only scattered, short, recumbent simple setae. *Scutellum*: Dull, with a broad band of silvery scale-like setae across middle. *Hemelytron*: Dull, cuneus and embolium polished; with bands of silvery, scale-like setae, one across base of clavus (and continuous with that on scutellum) and a broader one across apical third of corium, three narrow, short bands on apical third of clavus, and two short, narrow, somewhat broken patches on basal third of corium adjacent to embolium; intermixed with widely set, erect, stout, black, bristle-like setae on clavus and corium. *Ventral surface*: Shiny; abdomen with long erect and semierect pale setae.

*Male genitalia*: Aperture large, without marginal spines. Left paramere (Fig. 260) with a large, triangular apical process and two middle processes, one apically acute, one stout and decurved, and one slender. Right paramere (Fig. 262) C-shaped with a large, dorsally directed, apically serrate, lateral arm. Phallotheca (Fig. 261) slender, with a short, decurved, apical hook.

*Female*: Unknown.

##### Etymology.

I name this new species after my late mother, Betty Jean Vitello Walker, who always provided strong support for my interests in natural history that developed at an early age. The specific epithet comes from her affectionate account of my Italian grandfather’s accented pronunciation of her name, “Be-Jean”.

##### Distribution.

Known only from Sonora, Mexico; also intercepted in commerce at Nogales, Arizona [a border city across from the Mexican state of Sonora], from an unspecified locality in Mexico.

##### Host.

Unknown.

##### Discussion.

The male genitalia of this species are most similar to those of *Pilophoropsis brachyptera*, but they differ in the shape of the middle processes on the left paramere (Fig. 262), certain fine details in the right paramere (Fig. 262), and the much shorter, stouter, apical process on the phallotheca (Fig. 261).

##### Type material.

Holotype ♂, **MEXICO:** Intercepted at Nogales, Arizona, from Mexico, XII-29-41, coll. unknown, on tomato (00162226) (USNM). Paratype: **MEXICO:** Intercepted at Nogales, Arizona, 11 Apr 1966, coll. unknown, on tomato, 1♂ (00285719) (USNM). Mexico, Sonora, sand dunes at Huatabampito, 22 Apr. 1974, Derham Giuliani, 1♂ (CAFA).

#### Pilophoropsis brachyptera

Taxon classificationAnimaliaHemipteraMiridae

Poppius

Figs 74 , 75 , 120 , 121–126 , 127–132 , 263–265

Pilophoropsis brachypterus Poppius, 1914: 250 (orig. descrip.); 1952: 82 (as type); Carvalho 1955b: 227 (note); Polhemus and Polhemus 1985: 29 (note); Carvalho 1958: 142 (cat.); Schuh 1995: 182 (cat.).
Pilophoropsis balli Knight, 1968: 159 (orig. descrip.). Synonymized by Polhemus and Polhemus 1985: 29.
Pilophoropsis brachyptera : Steyskal 1973: 207 (etymology); Henry and Wheeler 1988: 399 (cat.).

##### Diagnosis.

This species (Figs 74, 75) is recognized by having the posterior silvery band on the hemelytra level with the apex of the clavus and well separated from the cuneal fracture, a comb-like basal process of the left paramere (Fig. 263), a long, recurving arm on the right paramere (Fig. 265), and a stout, decurved apical hook on the phallotheca (Fig. 264).

##### Description.

*Male* (n = 5; holotype measurements of *Pilophoropsis balli* in parentheses): Length 3.32–3.52 mm (3.46 mm), width 1.08–1.16 mm (1.12 mm). *Head*: Width 0.76–0.80 mm (0.78 mm), interocular width 0.30–0.32 mm (0.30 mm). *Labium*: Length 1.04–1.10 mm (1.09 mm). *Antenna*: Segment I, length 0.22–0.24 mm (0.22 mm); II, 0.58–0.60 mm (0.62 mm); III, 0.36–0.38 mm (0.37 mm); IV, 0.36–0.38 mm (0.35 mm). *Pronotum*: Length 0.76–0.78 mm (0.80 mm), basal width 1.02–1.06 mm (1.01 mm).

*Coloration*: *Head*: Dark shiny brown. *Antenna*: Segment I yellowish brown, with a red mark running dorsally across basal l/3, then distally along inside before ending subapically; segment II, brown to dark brown; segments III and IV dark brown. *Pronotum*: Dark brown to fuscous. *Scutellum*: Dark brown. *Hemelytron*: Dull, satiny tan to pale brown, outer half of clavus and apical fourth of corium darker dull brown; cuneus, inner margin of corium bordering membrane, and embolium polished, cuneus dark reddish brown; membrane dark brown, with a transparent area bordering cuneus. *Ventral surface*: Thorax dark brown; abdomen fuscous to nearly black. *Ostiolar evaporative area*: Pale or white. *Legs*: Coxae pale or whitish, all bases and apex of procoxae brown to reddish brown; femora brown to reddish brown, with basal l/3 of middle and hind femora pale; tibiae brown to reddish brown, pale on apical l/3 to l/2; tarsi and claws pale brown. *Ostiolar evaporative area*: Uniformly white.

*Structure, texture, and vestiture*: *Head*: Shiny, frons finely, transversely rugose. *Labium*: Extending to bases of middle coxae. *Antenna*: Segment I nearly glabrous, with a few long, erect setae; segment II slender basally, gradually enlarging toward apex to thickness equal with segment I, pubescence dense, short, and recumbent. *Pronotum*: Disc smooth, polished, strongly convex; calli rugose, narrowed anterior part transversely striate; clothed with sparsely set, recumbent, simple setae. *Scutellum*: Rugose, weakly convex, with a row of silvery scale-like setae across base. *Hemelytron*: Clothed with three bands of silvery, scale-like setae, a narrow one across base of clavus (and continuous onto scutellum), a short narrow one across apical third of clavus, and a broad one across apical half of corium near apex of clavus, intermixed with several irregular patches of silvery scale-like setae on basal half of corium along embolium and apical half of clavus, intermixed with scattered, stout, erect, black, bristle-like setae on clavus and corium. *Ventral surface*: Shiny dark brown to fuscous, with a dull, alutaceous patch across middle l/2 of 2nd visible abdominal segment; glabrous except for a few scattered, long, erect, simple setae on abdomen.

*Male genitalia*: Male aperture large, lacking spines or processes. Left paramere (Fig. 263) with apex twisted, beak-like; basal process three-pronged, comb-like process varying from that Figured to slightly longer and more slender. Right paramere (Fig. 265) with main stem stout, lateral arm long, slender, dorsally directed, apically crenulate, base a short, minor process arising near middle. Phallotheca (Fig. 264) with apex ending in a recurved hook.

*Brachypterous* *female* (n = 10; holotype measurements of *Pilophoropsis brachyptera* in parentheses): Length to apex of abdomen 2.60–3.12 mm (2.75 mm), length to base of cuneus 2.00–2.21 mm (2.00 mm), width 0.92-1.08 mm (1.07 mm). *Head*: Width 0.76–0.84 mm (0.80 mm), interocular width 0.40–0.44 mm (0.40 mm). *Labium*: Length 1.02–1.10 mm (1.04 mm). *Antenna*: Segment I, length 0.20–0.22 mm (missing); II, 0.56–0.60 mm (missing); III, 0.36 mm (missing); IV, 0.36 mm (missing). *Pronotum*: Length 0.62-0.70 mm (0.64 mm), basal width 0.60–0.66 mm (0.58 mm). Coloration and structure similar to that of male, except as noted below.

*Structure, texture, and vestiture*: *Labium*: Extending to bases of hind coxae. *Pronotum*: Much more quadrate than male, disc strongly convex with a deep cleft through middle; flattened, collar-like, anterior area transversely striate. *Hemelytron*: Brachypterous, claval suture absent, except apically in some specimens, clavus defined only by darker brown coloration on outer half; cuneus abbreviated on apical third, membrane reduced, not exceeding apex of abbreviated cuneus; cuneus, membrane, and embolium polished; abdomen broadly rounded, with apical four segments exposed beyond hemelytron. *Ventral surface and legs*: Much as in males.

##### Distribution.

Described and known only from Arizona.

##### Hosts.

Polhemus and Polhemus (1985) associated *Pilophoropsis brachyptera* with the mistletoe *Phoradendron californicum* Nutt. [Viscaceae] growing on *Prosopis* spp. [Fabaceae] in Arizona. A. G. Wheeler and I, however, have collected numerous specimens on mesquite (*Prosopis* sp.) lacking mistletoe. Our observations and the numerous other records given below, some with nymphs, indicate that *Prosopis* spp. are the primary hosts of *Pilophoropsis brachyptera*, rather than mistletoe. This species also has been taken on *Acacia greggii* A. Gray (Fabaceae), *Celtis ehrenbergiana* (Klotzsch) Liebm. (Ulmaceae), *Lycium pallidum* Miers (Solanaceae), and *Phoradendron californicum* Nutt. (Santalaceae). *Pilophoropsis brachyptera* is frequently taken on the stouter twigs and branches of its host tree, where it probably preys on various soft-bodied arthropods.

##### Type specimens examined.

Holotype (*Pilophoropsis brachyptera*): &, “Hot Spr[in]gs, 24–6, Ar., H. S. Barber Collector” (00162227) (USNM). Holotype (*Pilophoropsis balli*): ♂, “Tucson, Ar., 9–22–28, E. D. Ball” (00162226) (USNM). Paratype: **USA:** **Arizona:** ***Pima Co.*:** Rincon Mountains, 32.07611EN, 111.91722EW, 1006 m, 02 Sep 1928, A. A. Nichol (Paratype of *Pilophoropsis balli*), 1♂ (00374969) (CNC). Catal[ina] Springs, 15-4, E. A. Schwarz (paratype of *Pilophoropsis balli*), 1♂ (00285695) (USNM).

##### Other specimens examined.

**USA:** **Arizona:** ***Maricopa Co.*:** Fort McDowell, 33.63667EN, 111.67389EW, 13 Oct 1982, J. T. Polhemus, on mistletoe growing on *Prosopis* sp., 2♀♀ (00138474, 00138475) (JTP). Verde R. at Hwy. 87, CL1632, vi–2–81, J. T. Polhemus, taken on mistletoe growing on *Prosopis* sp., 1♂ (1 nymph) (JTP). Ft. McDowell, VIII–10–82, J. T. Polhemus, 3♀♀ (JTP). ***Pima Co.*:** 4 mi S of Interstate 10, along Rt 82 [sic; Rt 83], 31.95003EN, 110.66844EW, 1143 m, 02 Jun 1997, T. J. Henry and A. G Wheeler, Jr., *Prosopis* sp. (Fabaceae), 1 nymph (00285703), 3♀♀ (00285705-00285707) (USNM). 6 mi SE of Continental, 31.79796EN, 110.89233EW, 1084 m, 03 Jul 1958, W. F. Barr, 1♂ (00374970) (CNC). 14 mi W of Three Points, 32.03542EN, 111.54426EW, 948 m, 15 Aug 1988, W. F. Barr, *Celtis ehrenbergiana* (Klotzsch) Liebm. (Ulmaceae), 1♀ (00374971), 1 nymph (00374972) (CNC). Arivaca Lake, 2.7 mi SE of Arivaca, 31.52551EN, 111.2546EW, 1067 m, 19 Apr 1982, M. D. Schwartz, *Prosopis juliflora* (Fabaceae), 1♀ (00138472) (AMNH). Lower Santa Rita Range, near Sahuarita, 31.79705EN, 110.77413EW, 1463 m, 11 Apr 1989, T. J. Henry and A. G Wheeler, Jr., *Prosopis* sp. (Fabaceae), 1♀ (00285714), 1♂ (00285715) (USNM). NE Tucson, foothills of Sta. Catalina Mts., trail to Lower Sabino Dam East Picnic Area, 32.31408EN, 110.81149EW, 823 m, 27 Sep 1988, M. D. Schwartz, *Celtis ehrenbergiana* (Klotzsch) Liebm. (Ulmaceae), 1♂ (00138452), 3♀♀ (00138453-00138455), 1 nymph (00138456) (AMNH). Sabino Canyon, Tucson, 32.31011EN, 110.82122EW, 832 m, 16 May 1953, A. and H. Dietrich, 1♂ (00286347) (CUIC). Santa Catalina Mountains, Finger Rock Canyon, 32.3419EN, 110.90947EW, 985 m, 07 May 1988, W. A. Jones, *Acacia greggii* (Fabaceae), 2♀♀ (00285712, 00285713) (USNM). Santa Catalina Mountains, Ventana Canyon Trail, 32.33194EN, 110.86388EW, 30 Aug 1998, M. D. Schwartz, *Lycium pallidum* (Solanaceae), 1♂ (00374979), 2♀♀ (00374977, 00374978), 1 nymph (00374976) (CNC). Santa Catalina Mountains on Finger Rock Canyon Trail, T12S R14E, 32.35091EN, 110.9015EW, 1128 m, 05 Apr 1981, M. D. Schwartz, *Lycium* sp. (Solanaceae), 1♂ (00138473) (AMNH). Santa Rita Exp[erimental]. Range, 31.88166EN, 110.86569EW, 985 m, 16 Apr 1990, W. A. Jones, *Phoradendron californicum* (Santalaceae), 1 nymph (00285708), 3♀♀ (00285709-00285711) (USNM). Santa Rita Mountains, Madera Cyn., 31.74251EN, 110.88533EW, 1341 m, 22 Aug 1989, W. A. Jones, 1♂ (00285718) (USNM). Tortolito Mountains, 32.4266EN, 111.08935EW, 762 m, 22 Apr 1982, M. D. Schwartz, *Prosopis juliflora* (Fabaceae), 2♀♀ (00138469, 00138470) (AMNH). Tortolito Mountains, 32.46648EN, 111.06777EW, 914 m, 22 Apr 1982, M. D. Schwartz, *Prosopis juliflora* (Fabaceae), 1♂ (00286348) (CUIC). Tucson, Santa Catalina Mountains, Bear Creek near Saddleback Dr, 32.325EN, 110.79083EW, 26 Feb 1995, M. D. Schwartz, *Phoradendron* sp. (Santalaceae), 1♂ (00374974), 1♀ (00374973), 1 nymph (00374975) (CNC). USDA Lab., Tucson, 32.27611EN, 110.94019EW, 720 m, 08 Apr 1989, T. J. Henry and A. G Wheeler, Jr., *Prosopis* sp. (Fabaceae), 1♀ (00285716), 1♂ (00285717) (USNM).

#### Pilophoropsis cunealis

Taxon classificationAnimaliaHemipteraMiridae

Henry sp. n.

http://zoobank.org/3DB7C288-C010-4747-9678-AB4AC7E79467

Figs 76–78 , 266–268

##### Diagnosis.

This species (Figs 76–78) is distinguished by the posterior band of silvery scale-like setae bordering the cuneal fracture and the male genitalia, particularly the long, slender middle process of the left paramere (Fig. 266), the expanded, T-shaped apex of the lateral arm on the right paramere (Fig. 268), and the long, slender apical process of the phallotheca (Fig. 267).

##### Description.

*Male* (n = 2; holotype measurements in parentheses): Length 2.85 mm (2.98 mm), width 0.99 mm (1.01 mm). *Head*: Width 0.69 mm (0.70 mm), interocular width 0.30 mm (0.31 mm). *Labium*: Length 1. 04 mm (1.09 mm). *Antenna*: Segment I, length 0.21 mm (0.21 mm); II, 0.59 mm (0.61 mm); III, 0.30 mm (0.33 mm); IV, missing (0.35 mm). *Pronotum*: Length 0.72 mm (0.72 mm), basal width 0.86 mm (0.91 mm).

*Coloration*: *Head*: Dark brown, clypeus, jugum, lorum, and area around base of antenna paler brown. *Antenna*: Segment I pale brown with a dark reddishbrown band on anterior and inner surface near base; segments II– IV dark brown; apex of II, apical half of III, and IV swollen. *Pronotum*: dark brown, anterior area around collar more yellow or yellowish brown. *Scutellum*: Pale yellowish brown. *Hemelytron*: Brown, with inner margin of clavus and basal half of corium inside radial vein pale yellowish brown, cuneus reddish; membrane uniformly brown;. *Legs*: Coxae pale yellow or whitish, with a few red marks apically; femora reddish brown, middle and hind femora pale on basal fourth; tibiae reddish brown on basal third to half, pale yellow beyond; tarsi pale yellow; claws pale brown. *Ventral surface*: Uniformly reddish brown, abdominal patch paler brown. *Ostiolar evaporative area*: White, weakly tinged with red dorsally.

*Structure, texture, and vestiture*: *Labium*: Extending to apices of middle coxae. *Pronotum*: Shiny, impunctate; narrowed anterior area around calli granulate, disk smooth. *Scutellum*: Base partially hidden by base of pronotum; lacking scale-like setae. *Hemelytron*: Embolium and cuneus polished, remainder of corium dull; corium and base of cuneus with numerous stout, black, bristle-like setae, clavus with six or more short, transverse bands of silvery scale-like setae, outer margin of corium bordering embolium with two short transverse bands, one on basal third and one at middle, apex of corium bordering cuneus with a large transverse band of silvery scale-like setae disjunct at middle (Figs 76, 77).

*Male genitalia*: Aperture of capsule smooth, lacking spines or processes. Left paramere (Fig. 266) with beak-like process more quadrate; basal process with a long, slender apex. Right paramere (Fig. 268) with lateral arm T-shaped apically. Phallotheca (Fig. 267) with a long, curved, apically acute process.

*Macropterous female* (n = 4): Length to apex of abdomen 2.46–2.85 mm, length to base of cuneus 1.98–2.18 mm, width 1.04–1.10 mm. *Head*: Width 0.69–0.75 mm, interocular width 0.35–0.42 mm. *Labium*: Length 1.06–1.14 mm. *Antenna*: Segment I, length 0.19–0.22 mm; II, 0.58–0.61 mm; III, 0.32–0.35 mm; IV, 0.30–0.35 mm. *Pronotum*: Length 0.61–0.72 mm, basal width 0.74–0.85 mm. Color and structure much as in males, including the structure of the pronotum and hemelytra, with the membrane only slightly more abbreviated than fully developed males.

##### Etymology.

Named “cunealis” to denote the posterior band of silvery scale-like setae along the cuneal fracture, a character unique among species of the genus.

##### Host.

*Acacia cochliacantha* Humb. & Bonpl. ex. Willd. [Fabaceae].

##### Distribution.

Known only from Oaxaca, Mexico.

##### Discussion.

unlike the strongly modified females of other species in the genus, such as *Pilophoropsis brachyptera*, females of *Pilophoropsis cunealis* are almost fully macropterous as in males, except for the slightly abbreviated hemelytral membrane and, in one case, a slightly more shortened cuneus.

##### Type material.

Holotype ♂: **MEXICO:** **Oaxaca:** Puerto Escondido, 15 Jul 1985, Jones, Schaffner, *Acacia cochliacantha* (Fabaceae) (00286346) (TAMU). Paratypes: **MEXICO:** **Oaxaca:** Puerto Escondido, 15 Jul 1985, Jones, Schaffner, *Acacia cochliacantha* (Fabaceae) 1♂, 3♀♀ (1 5th instar) (00167516-00167517) 2♀♀ TAM; (0016228) 1♂, (00162229) 1♀ USNM). 2.1 mi. NW Totolpan, July 11–17, 1981, Bogar, Schaffner, and Friedlander, 1♀ (00167518) (TAMU).

#### Pilophoropsis nicholi

Taxon classificationAnimaliaHemipteraMiridae

(Knight)

Figs 79–81 , 269–271

Renodaella nicholi Knight, 1927: 307 (orig. descrip.); Carvalho 1952: 83 (as type).
Pilophoropsis nicholi : Carvalho, 1958: 142 (cat., new comb.); Knight 1968: 159 (descrip., key); Henry and Wheeler 1988: 399 (cat.); Schuh 1995: 182 (cat.).

##### Diagnosis.

This species (Figs 79–81) is recognized by a narrow band of silvery scale-like setae across the base of the clavus, the more irregular, scattered patches on the apical half of the clavus, and another broken, transverse band on the basal half of the corium about level with the apex of the scutellum; and the male genitalia, particularly the stout basal processes of the left paramere with an adjacent slender spine (Fig. 269), the three lateral arms of the right paramere (Fig. 271), and the long, recurved, somewhat flattened, apical process of the phallotheca (Fig. 270).

##### Description.

*Male* (n = 2; holotype in parentheses): Length 3.20–3.32 mm (3.20 mm), width 1.01–1.04 mm (1.06 mm). *Head*: Width 0.68–0.78 mm (0.72 mm), interocular width 0.28–0.35 mm (0.32 mm). *Labium*: Length 1.08–1.20 mm (1.20 mm). *Antenna*: Segment I, length 0.20–0.23 mm (0.22 mm); II, 0.56–0.61 mm (0.62 mm); III, 0.36–0.38 mm (0.35 mm); IV, 0.36 mm (0.38 mm). *Pronotum*: Length 0.68–0.73 mm (0.72 mm), basal width 0.92–1.00 mm (0.96 mm).

*Coloration*: *Head*: Shiny dark brown to reddish brown. *Antenna*: Segment I pale brown, with a red U-shaped mark at base; segments II–IVdark brown. Pronotum: Shiny dark brown to reddish brown. *Hemelytron*: Corium dull yellowish brown, darker on distal half; clavus dark yellowish brown, paler along scutellar margin; cuneus and apex of embolium shiny reddish brown *Ventral surface*: Shiny reddish brown; glaucous abdominal stridulatory patch paler; nearly glabrous. *Ostiolar evaporative area*: Pale or white. *Legs*: Coxae whitish, fore coxae red apically, hind coxae brown at base; femora brown to reddish brown, middle and hind femora pale on basal halves; tibiae brown, fore and midddle tibiae pale on apical thirds; tarsi and claws pale brown.

*Structure, texture, and vestiture*: *Head*: Shiny, weakly rugose on frons. *Labium*: Extending to hind coxae. *Antenna*: Segment II slender, gradually thickening to apex, weakly pubescent on basal half, more thickly set with recumbent pale setae on apical half. *Pronotum*: Shiny, disc impunctate, calli weakly granulate and weakly depressed between, narrow anterior collar-like area weakly transversely striate; sparsely set with widely separated, recumbent, pale setae. *Scutellum*: Dark brown, with a narrow band of silvery scale-like setae through middle. *Hemelytron*: Clothed with bands and patches of silvery scale-like setae, including a band across base of clavus (and continuous onto scutellum), four short bands on apical half of clavus, three short patches on basal third of corium adjacent to embolium, and a large patch across apical third of corium ending before claval suture; intermixed on corium and clavus with widely set, stout, erect, black, bristle-like setae; membrane smoky brown, shiny along base (and beneath rubbed pubescent areas). *Ventral surface*: Thorax glabrous; abdomen with a few erect and semierect setae.

*Male genitalia*: Aperture large, without spines or processes. Left paramere (Fig. 269) with a triangular beak-like apical process and two basal processes, one stout and recurved (with a short basal spine) and the other long, slender, and needle-like. Right paramere (Fig. 271) with stout C-shaped trunk and three lateral arms, two relatively short, distally acute, and one larger, recurving behind main trunk. Phallotheca (Fig. 270) slender, ending in a long, slender, somewhat flattened, recurving spine.

*Brachypterous* *female* (n = 2): Length to apex of abdomen 2.96–3.12 mm, length to base of cuneus 2.08–2.11 mm, width 1.02–1.16 mm. *Head*: Width 0.80–0.82 mm, interocular width 0.40–0.42 mm. *Labium*: Length 1.10 mm (1 specimen obscured in glue). *Antenna*: Segment I, length 0.20–0.22 mm; II 0.62–0.66 mm; III, 0.38 mm (missing on 1 specimen); IV, 0.38 mm (missing on 1 specimen). *Pronotum*: Length 0.62–0.66 mm, basal width 0.66–0.70 mm.

*Coloration*: Similar to male.

*Structure, texture, and vestiture*: *Labium*: Extending to bases of middle coxae. *Pronotum*: Quadrate, disc smooth, shiny, transversely rugose along decurved posterior edge, calli shiny, cleft between, flattened, anterior, collar-like area transversely striate. *Scutellum*: Transversely rugose, with a band of silvery scale-like setae across base. *Hemelytron*: Narrowed basally, widening toward truncate apex; claval suture absent, fusing clavus and corium; cuneus abbreviated on apical third, membrane reduced to level of cuneus, truncate apex shallowly emarginate, cuneus, membrane, and embolium polished; lateral margins and apical three segments of abdomen exposed beyond hemelytron; silvery scale-like bands and patches much as in male, including three distinct patches on basal third of corium along embolium.

##### Distribution.

Described and previously known only from Arizona. Mexico (Jalisco) is a new country record.

##### Host.

One specimen taken on desert willow, *Chilopsis linearis* (Cav.) Sweet. [Bignoniaceae].

##### Discussion.

The genitalia illustrated for *Pilophoropsis nicholi* are based on a male from Jalisco, Mexico. Although these Figures are very similar to Knight’s (1968) illustrations, they differ slightly because of the angle they were drawn. The left paramere in Knight’s (1968) drawing shows the needle-like basal process appearing to originate from the main trunk, but it actually arises from the base of the stouter process, as in my illustration. Also, the right paramere in Knight’s (1968) illustration shows only two lateral arms or spines. If the right paramere is turned up slightly and counter clockwise, three processes can be seen; the lowermost arm curls back behind the main trunk when viewed caudally, making it difficult to see. The apex of the phallotheca, as illustrated by Knight (1968), and my illustration are similar, though the recurved apex in the holotype appears even more flattened than shown in Knight’s (1968) or my illustration. At this time, I consider all of this material conspecific.

##### Type material.

Holotype ♂: **USA:** **Arizona:** Santa Rita Mtns., alt.4500 ft., Sept. 9, 1925, A. A. Nichol (00285699) (USNM).

##### Other specimens examined.

**MEXICO:** **Jalisco:** Puerto Vallarta, 8 Dec. 1984, G. E. Bohart, 1♂ (USNM). **USA:** **Arizona:** Santa Rita Mts., 16 May 1928, A. A. Nichol, 1♀ (USNM). Santa Rita Mts., 9 May 1929, E. D. Ball, 1♀(00285702) (USNM). Pena Blanca [Santa Cruz Co.], 2 July 1949, L. A. Lindsay on desert willow, 1♂ (00285701) (USNM).

#### Pilophoropsis quercicola

Taxon classificationAnimaliaHemipteraMiridae

Henry sp. n.

http://zoobank.org/253D210F-8B88-4480-BC68-12BFE73563D0

Figs 82 , 83 , 272–275

##### Diagnosis.

This species (Figs 82, 83) is recognized by the general dark color, the wide band of silvery scale-like setae transversing the corium level with the apex of the clavus, and the male genitalia, particularly the left paramere (Fig. 272) with three middle processes, the stout, bifurcate lateral arm of the right paramere (Figs 274, 275), and the unique recurved apical process of the phallotheca (Fig. 273).

##### Description.

*Male* (n = 2; holotype measurements in parentheses): Length 3.16 mm (3.30 mm), width 0.96 mm (1.04 mm). *Head*: Width 0.74 mm (0.75 mm); interocular width 0.33 mm (0.34 mm). *Labium*: Length 1.14 mm (1.18 mm). *Antenna*: Segment I, length 0.20 mm (0.20 mm); II, 0.55 mm (0.57 mm); III, 0.34 mm (0.35 mm); IV, 0. 38 mm (missing). *Pronotum*: Length 0.69 mm (0.70 mm); basal width 0.91 mm (0.94 mm).

*Coloration*: *Head*: Dark brown to fuscous, paler brown along inner margin of eyes and around antennal bases. *Antenna*: Segment I brown, with a narrow fuscous stripe and a red spot on inner surface, segments II–IV uniformly dark brown or fuscous. *Pronotum*: Dark brown. *Scutellum*: Dark brown to fuscous. *Hemelytron*: Clavus and corium dull yellowish brown, becoming darker posteriorly; embolium and cuneus shiny reddish brown. *Ventral surface*: Reddish brown to fuscous; glaucous abdominal stridulatory patch pale. *Ostiolar evaporative area*: Uniformly white. *Legs*: Fore coxa white with base narrowly infuscated and apex red tinged, middle coxa white with base infuscated, hind coxa uniformly white; fore femora dark brown, middle and hind femora dark brown with basal thirds whitish; tibiae dark brown, becoming paler on apical thirds; tarsi and claws brown.

*Structure, texture, and vestiture*: *Labium*: Extending to bases of middle coxae. *Pronotum*: Anterior third, granulate, narrowed, sloping toward head, calli indistinct, flattened; posterior two thirds strongly convex, polished, impunctate, basal angles flared. *Scutellum*: With a band of silvery scale-like setae across base. *Hemelytra*: Dull area with two distinct bands of silvery, scale-like setae, a narrow one across base of clavus and through scutellum and a broad one across corium at level with apex of clavus, also with 6 patches of silvery scale-like setae, two at emboliar margin on basal half of corium and four on clavus; intermixed with scattered, stout, erect, black, bristle-like setae on dull areas of clavus and corium. *Ventral surface*: Shiny, with scattered erect and semierect simple setae on abdomen.

*Male genitalia*: Left paramere (Fig. 272) with three apically acute and one slender basal processes; right paramere (Figs 274, 275) with a bifurcate lateral arm having two small processes arising at base; phallotheca (Fig. 273) stout, with a thick, decurved, beak-like apical process.

*Macroperous female*: (n = 1): Length 3.00 mm, width 1.11 mm. *Head*: Width 0.78 mm, interocular width 0.39 mm. *Labium*: Length 1.24 mm, extending to middle coxae. *Antenna*: Segment I, 0.21 mm; II, 0.61 mm; III, 0.38 mm; IV, 0.36 mm. *Pronotum*: Length 0.62 mm, basal width 0.88 mm.

*Brachypterous* *female* (n = 3): Length to apex of abdomen 2.65–2.75 mm, length to base of cuneus 2.14–2.24 mm, width 1.00–1.05 mm. *Head*: Width 0.78–0.79 mm, interocular width 0.40 mm. *Labium*: 1.18–1.20 mm, extending nearly to bases of hind coxae. *Antenna*: Segment I, length 0.20 mm; II, 0.86–0.87 mm; III, 0.31–0.35 mm; IV, 0.33–0.36 mm. *Pronotum*: Length 0.53–0.59 mm, basal width 0.70–0.74 mm.

##### Etymology.

This species is named for the generic name of its host.

##### Distribution.

Known only from Arizona.

##### Host.

All specimens of the type series were collected on Mexican blue oak, *Quercus oblongifolia* Torr. (Fagaceae).

##### Type material.

Holotype ♂: **USA:** **Arizona:** ***Cochise Co.*:** Huachuca Mountains, Ash Canyon Road, nr. Rt. 92, S. Sierra Vista, 31.55454EN, 110.30369EW, 1646 m, 04 Jun 1997, T. J. Henry and A. G Wheeler, Jr., *Quercus oblongifolia* Torr. (Fagaceae) (00285722) (USNM). Paratypes: **USA:** **Arizona:** ***Cochise Co.*:** Huachuca Mountains, Ash Canyon Road, Jct. 92, 31.38938EN, 110.23033EW, 1539 m, 05 Jun 1997, T. J. Henry and A. G Wheeler, Jr., *Quercus oblongifolia* Torr. (Fagaceae), 1♂ (00285723) 2♀♀ (00285724, 00285725) (USNM).

#### Pilophoropsis texana

Taxon classificationAnimaliaHemipteraMiridae

(Knight) comb. n.

Figs 84 , 85 , 276–279

Renodaeus texanus Knight, 1926: 56 (orig. descrip.); Carvalho 1958: 150 (cat.); Henry and Wheeler 1988: 445 (cat.); Schuh 1995: 190 (cat.).

##### Diagnosis.

This species (Figs 84, 85) is recognized by the band of silvery scale-like setae bordering the cuneal fracture and the male genitalia with the unique basal process of the left paramere (Fig. 276), the short, bifid lateral arm of the right paramere (Figs 278, 279), and decurved apical hook on the phallotheca (Fig. 277).

##### Description.

*Male* (n = 1): Length 3.20 mm, width about 1.20 mm (hemelytra spread). *Head*: Width 0.72 mm, interocular width 0.26 mm. *Labium*: Length 1.06 mm. *Antenna*: Segment I, length 0.20 mm; II, 0.62 mm; III, 0.34 mm; IV, 0.34 mm. *Pronotum*: Length 0.70 mm, basal width 0.98 mm.

*Coloration*: *Head*: Brown, darker reddish brown across vertex and red on clypeus. *Antenna*: Segment I brown, with a large red blotch on basal third and a more narrow red mark on apical third touching apex; segment II reddish brown; segments III and IV reddish brown. *Pronotum*: Reddish brown. *Hemelytron*: Dull tan to brown, darker brown outside radial vein, on outer half of clavus, and apical third of corium, cuneus and apex of embolium reddish brown. *Ventral surface*: Reddish brown. *Ostiolar evaporative area*: White, with central knob at end of scent channel red tinged. *Legs*: Coxae pale to whitish, fore coxae red on apical half, bases of middle and hind coxae brown to reddish brown; all legs missing except one hind leg, femur reddish brown, pale at base; tibia reddish brown, pale on apical half; tarsus and claw pale brown.

*Structure, texture, and vestiture*: *Head*: Shiny, rugose on frons, with relatively, short pale, recumbent, brown setae. *Labium*: Extending to bases of middle coxae. *Antenna*: Segment II slender, only slightly thickened out to clavate apex, apex 2X diameter of base; segment III slender on basal half, thickened apically; segment IV uniformly thickened, fusiform, diameter equal to apical thickness of III. *Pronotum*: Disc polished, narrow anterior area across calli granulate, narrow anterior collar-like area merged with calli, posterior angles flared, with a small depressed area just inside posterior margin, setae pale, recumbent, sparse; scutellum dark brown, only two individual scale-like setae present. *Hemelytron*: Cuneus, embolium, and base of membrane polished; clothed with two bands of silvery scale-like setae, one across base of clavus and a broken one bordering cuneal fracture, and also with two patches on basal half of corium adjacent to embolium, and one narrow band and several (3-4) small patches on apical third of clavus, intermixed on clavus and corium with widely set, stout, erect, black, bristle-like setae. *Ventral surface*: Shiny, abdomen with long, erect and semierect pale brown setae.

*Male genitalia*: Genital aperture large, unarmed. Left paramere (Fig. 276) with triangular, beak-like apical process and a broad basal process with long slender arm extending outward. Right paramere (Figs 278, 279) with a slender mainstem and a large, apically flared, lateral arm. Phallotheca (Fig. 277) slender, with a short, sharply recurved, apical hook.

*Female* (n = 3; holotype measurements in parentheses): Length 2.36–2.64 mm (2.55 mm), width 1.04 mm [hemelytra spread on one specimen] (1.04 mm). *Head*: Width 0.74–0.78 mm (0.77 mm), interocular width 0.40-0.44 mm (0.43 mm). *Labium*: Length 0.96–1.02 mm (0.96 mm). *Antenna*: Segment I, length 0.20 mm (0.19 mm); II, 0.50–0.56 mm (0.54 mm); III, 0.30 mm (0.29 mm); IV, 0.32 mm (missing). *Pronotum*: Length 0.62–0.70 mm (0.66 mm), basal width 0.74–0.80 mm (0.80 mm). Coloration and structure similar to that of male, except as noted below.

*Coloration*: *Pronotum*: Reddish brown, paler brown anteriorly. *Scutellum*: Dark brown to fuscous. *Hemeytron*: Dull tan to brown.

*Structure, texture, and vestiture*: *Labium*: Extending to hind coxae. *Pronotum*: Much more quadrate than in male, disc convex, shiny, finely punctate or granulate, anterior half across calli narrowed into a thickened, rugose, neck-like area; pubescence on disc sparse and short, more thickly set and longer setae on anterior half. *Scutellum*: Scale-like pubescence absent or rubbed. *Hemelytron*: Apex extending to end of abdomen; silvery scale-like patches and bands, intermixed with stout, bristle-like setae as in male; cuneus shortened but appearing fully developed (triangular), membrane shortened, extending to apex of abdomen; cuneus, embolium, and membrane polished.

##### Discussion.

Males and females in this genus are difficult to associate without field-associated specimens. Comparison of the holotype female’s hemelytral setal pattern and distinctive antennal shape with a male intercepted from San Pedro [Coahuila or Vera Cruz?], Mexico, makes me reasonably certain they are conspecific. One male from Rio Hondo, Oaxaca (listed below), is in poor condition (hemelytra detached; one missing) and the genitalia are embedded in glue on a separate card. This specimen also is very similar externally to the feHolotype male, although there is some subtle variation in the parameres. Nevertheless, I am tentatively labeling it as *Pilophoropsis texana* as well. Externally, *Pilophoropsis texana* is almost indistinguishable from *Pilophoropsis cunealis*, but the male genitalia are quite different, especially the basal process of the left paramere and the distinctive apex of the phallotheca.

##### Distribution.

Described and previously known only from Brownsville, Texas (USA). Coahuila, Oaxaca, and Vera Cruz represent the first state records for Mexico.

##### Hosts.

No definite host known. Intercepted specimens on Bromeliaceae and Orchidaceae.

##### Type specimen examined.

Holotype ♀: **USA:** **Texas:** ***Cameron Co.*:** “Esp[e]r[an]za, R[an]ch, Brownsville, Tex.” (CU).

##### Other specimens examined.

**MEXICO:** [**Coahuila**?]: San Pedro, #1997, with Orchidaceae, 1♂ (00285721) (USNM). **Oaxaca:** 3.3 mi E of Rio Hondo, 16.43332EN, 95.76663EW, 05 Aug 1980, Schaffner, Weaver, Friedlander, 1♂ (00286356) (USNM). **Veracruz:** intercepted in Texas, 20 Mar 1962, ex bromeliads, 1♀ (00286355) (USNM). unknown, 20 Mar 1962, APHIS port inspector, bromeliads, 1♂ (00285720) (USNM).

#### Pilophoropsita

Taxon classificationAnimaliaHemipteraMiridae

Henry gen. n.

http://zoobank.org/872459AD-5C23-476F-B6E9-80BC51373660

##### Type species:

*Pilophoropsita schaffneri* Henry, sp. n.

##### Diagnosis.

This new genus is distinguished by the large eyes occupying nearly 80% of the dorsal head width in males (and more than 50% in females), the short labial segment I extending only slightly beyond the base of the head, the modified pronotum having a shiny swollen disc and a short neck-like anterior lobe, and the mostly dull hemelytron having only the embolium and cuneus polished and evenly distributed, scale-like setae intermixed with long, erect, simple setae. Females, unlike those of *Pilophoropsis* and *Pilophoropsidea*, are fully macropterous.

##### Description.

Male and females macropterous. Length of males 2.66–2.99 mm; length of females 2.56–2.88 mm. Head broader than long; posterior margin truncate, distinctly carinate, basal margin of vertex weakly indented medially; eyes large, grainy, more so in males, occupying more than 80♂ of dorsal width in males, more than 50♂ in females, laterally occupying 100♂ of height in males, nearly 100♂ in females with only vertex narrowly visible; frontal area relatively narrow and weakly rounded, clypeus moderately acute, short, partially visible in dorsal aspect; buccula narrow, labial segment I readily visible, extending very slightly beyond base of head, labium extending to posterior margin of sternum or bases of middle coxae. Antennal segment I shortest and thinnest, II longest, III and IV subequal, segment II–IV subequally thick. Pronotum narrowed anteriorly across area of calli into a slender, unarmed, neck-like area, calli smooth; posterior lobe trapeziform, shiny, impunctate or very finely and sparsely punctate to alutaceous, swollen above level of head and hemelytra, lateral margins and base weakly convex; mesoscutum hidden by base of pronotum; scutellum flat, equilateral, base narrowly covered by base of pronotum, with a thick band of silvery, scale-like setae across basal half, intermixed with long, erect, simple setae. Hemelytron generally dull or satiny, with the embolium and cuneus shiny or polished, lateral margins parallel, evenly clothed with silvery, scale-like setae, intermixed on clavus and corium with scattered, long, erect simple setae; membrane entire, with two closed cells. Ventral surface shiny; ostiolar auricle mostly red tinged, bordered by white, without raised knob on channel; second visible abdominal segment with a dull or glaucous patch ventrally. Legs unmodified; parempodia fleshy, convergent apically. Male genital aperture moderately large, round, unarmed around margins. Parameres complex as most members of *Renodaeus* group; left paramere with an elongate, beak-like apical process and a short, grooved process at middle; right paramere with a long, stout, lateral arm curved upward at apex; phallotheca elongate, with a long, slender, recurved, apical process; endosoma unmodified.

##### Etymology.

The name of this new genus is formed from the combination of the generic name *Pilophoropsis* and the suffix “ita”, meaning little or small one in Spanish, and is used to imply a close relationship of this new genus to members of the *Pilophoropsis*-*Renodaeus* group. The gender is feminine.

##### Discussion.

This new genus superficially resembles members of the genus *Zanchisme* in having a narrow anterior neck-like area on the pronotum. In *Pilophoropsita*, the neck-like area is short, smooth, and unarmed, whereas in *Zanchisme* it is more extensively raised, especially on the anterior margin and is armed with a pair of tubercles (though sometimes only weakly developed) or spines. Also, in *Pilophoropsita*, the head is broader than long, with proportionately large eyes, and the hemelytra have evenly scattered scale-like setae; in *Zanchisme* the head is elongate and rounded and the hemelytra have distinct transverse bands of scale-like setae. *Pilophoropsita* is most similar to *Ceratocapsidea*, *Pilophoropsidea*, and *Renodaeus* in having scattered scale-like setae on the hemelytra.

#### Pilophoropsita schaffneri

Taxon classificationAnimaliaHemipteraMiridae

Henry sp. n.

http://zoobank.org/82663C74-2D39-4880-A517-FCCDBE3C7CE6

Fig. 86–88 , 283–285

##### Diagnosis.

This species (Figs 86–88) can be recognized by the small size and characters given under the generic description, including the narrowed anterior lobe of the pronotum, scattered scale-like setae on the hemelytra, and the male genitalia (Figs 283–285).

##### Description.

*Male* (n = 8; holotype measurements in parentheses): Length 2.66–2.99 mm (2.85 mm); width 0.94–1.05 mm (0.96 mm). *Head*: Width 0.75–0.77 mm (0.75 mm); interocular width 0.14–0.16 mm (0.14 mm). *Labium*: Length 0.90–1.02 mm (0.98 mm). *Antenna*: Segment I, length 0.20–0.21 mm (0.20 mm); II, 0.56–0.60 mm (0.57 mm); III, 0.31–0.35 mm (0. 35 mm); IV, 0.33–0.35 mm (0.33 mm). *Pronotum*: Length 0.59–0.62 mm (0.59 mm), basal width 0.90–0.96 mm (0.91 mm); anterior width 0.42–0.43 mm (0.43 mm).

*Coloration*: Overall coloration dark reddish brown. *Head*: Dark reddish brown. *Labium*: Brown, segment I more reddish brown. *Antennae*: Segment I pale brown, with a nearly U-shaped red mark arising from base; segments II–IV dark brown. *Pronotum*: Dark reddish brown. *Scutellum*: Dull reddish brown. *Hemelytron*: Dull yellowish brown on clavus and inner half of corium, shiny reddish brown on outer half of corium, embolium and cuneus; membrane smoky brown. *Ventral surface*: Shiny reddish brown; glaucous stridulatory patch on abdomen pale, with a bluish luster. *Ostiolar evaporative area*: Reddish, narrowly white along posterior margin. *Legs*: Fore coxa pale brown, tinged with red, middle and hind coxae pale or whitish; femora reddish brown, basal third of middle and hind femora pale; tibiae reddish brown, apical half of fore and middle tibiae pale; tarsi pale brown, claws darker brown.

*Structure, texture, and vestiture*: *Head*: Shiny, impunctate, frons weakly rugose. *Labium*: Extending to bases of middle coxae. *Pronotum*: Shiny; posterior lobe finely punctate; anterior lobe weakly transversely rugose. *Scutellum*: Thickly covered with silvery scale-like setae on basal half and with 6 or more long, erect, pale, simple setae. *Hemelytron*: Uniformly clothed with silvery scale-like setae on clavus, corium, embolium, and paracuneus, intermixed with long, erect, pale brown, simple setae on clavus and corium.

*Male genitalia*: Left paramere (Fig. 283) with an elongate beak-like apical process and a short, apically grooved or cupped middle process. Right paramere (Fig. 285) with an elongate main stem and straight lateral arm curving upward at apex. Phallotheca (Fig. 284) elongate, with a long, slender, recurved, apical process.

*Female* (n = 10): Length 2.56–2.88 mm, width 0.96–1.09 mm. *Head*: Width 0.66–0.73 mm, interocular width 0.26–0.27 mm. *Labium*: 0.94–0.98 mm. *Antenna*: Segment I, length 0.20–0.21 mm; II, 0.51–0.56 mm; III, 0.33–0.34 mm; IV, 0.33–0.34 mm. *Pronotum*: Length 0.57–0.65 mm, basal width 0.86–0.94 mm, anterior width 0.43–0.46 mm. Similar to male in coloration and vestiture.

##### Etymology.

This new species is named after Dr. Joseph C. Schaffner (TAMU), who along with the late Dr. José C. M. Carvalho, first recognized it as probably representing a new genus.

##### Distribution.

Described from Costa Rica and the Mexican states of Jalisco, Nayarit, and Oaxaca.

##### Host.

*Acacia cochliacantha* Humb. & Bonpl. ex. Willd. [Fabaceae].

##### Type material.

Holotype ♂: **MEXICO:** **Oaxaca:** Puerto Escondido, 15 Jul 1985, Jones, Schaffner, *Acacia cochliacantha* (Fabaceae) (00285726) (TAMU). Paratypes: **COSTA RICA:** **Guanacaste:** 14 km S of Canas, 10.318EN, 85.045EW, 16 Mar 1991–23 Mar 1991, F. D. Parker, 1♀ (00286351) (USU). **MEXICO:** **Jalisco:** Estacion de Biologia, Chamela, 19.5333EN, 105.0666EW, 16 Apr 1992, A. Gomez & A. Rodriguez, 1♂ (00286323), 1♀ (00286326) (UNAM). Puerto Vallarta, 20.62201EN, 105.22845EW, 07 Dec 1986, G. E. Bohart, 1♀ (00286350) (USU). **Nayarit:** Lake Station Maria del Oro, 20 mi. E., 14 mi. N. Tepic, 28 Dec 1971, R.R. and M.E. Murray, 1♀ (00286344) (TAMU). **Oaxaca:** 3.3 mi. E. Rio Hondo, 05 Aug 1980, Schaffner, Weaver, Friedlander, 2♂♂ (00286364, 00286365), 1♀ (00286366) (TAMU), 2♀♀ (00285736, 00285737) (USNM). Puerto Escondido, 15 Jul 1985, Jones, Schaffner, *Acacia cochliacantha* (Fabaceae), 2♂♂ (00285727, 00285728), 7♀♀ (00285729-00285735) (USNM); 15 Jul 1985, J. B. Woolley and G. Zolnerowich, 1♂ (00286345), 3♀♀ (00286367-00286369) *Acacia cochliacantha* (Fabaceae), 1 (?sex) (00286307), 15♀♀ (00286308-00286322) (TAMU); 11 Jul 1987–12 Jul 1987, Kovarik and Schaffner, 2♀♀ (00286362, 00286363) (TAMU).

##### Other specimen examined.

**MEXICO:** **Quintana Roo:** San Isidro, Puerto Morelos, 09 Aug 1982, M. Garcia, 1♀ (00286329) (UNAM).

#### Renodaeus

Taxon classificationAnimaliaHemipteraMiridae

Distant

Renodaeus Distant, 1893: 461 (orig. descrip.); Knight 1926: 57 (notes, diag., n. tribe); Carvalho 1952: 83 (list); 1955: 80 (key); 1958: 150 (cat.); Schuh 1974: 285 (note, moved to Orthotylini); Henry and Wheeler 1988: 445 (cat.); Schuh 1995: 190 (cat.). Type species: *Renodaeus ficarius* Distant. Monotypic.

##### Diagnosis.

This genus is recognized by the constricted, punctate hemelytron with patches of golden scale-like setae and bands of silvery scale-like setae on the clavus and corium, the punctate pronotum (at least on posterior half), the heavily punctate clavus and basal half of corium, the finely punctate cuneus, and the row of file-like, tiny black spicules (stridulitrum) along the edge of the costal margin in males and females.

##### Description.

Length 2.60–3.30 mm. Myrmecomorphic. Males and females macropterous. Head declivent, subtriangular in dorsal aspect, ventrally flattened in lateral aspect, basal margin truncate, vertex with a distinct smooth carina, basal margin of eyes level with base of vertex, frons granulate or punctate, clypeus not or hardly visible in dorsal aspect; eyes moderately large, combined widths occupying about half dorsal width of head in males, about two thirds in females, oblong oval in lateral aspect; segment I of labium extending beyond gular sulcus by about half its length, visible below buccula in lateral aspect, extending to middle coxae or beyond. Antenna generally slender, segment I shortest, II longest, slender basally, gradually enlarging to apex which is sometimes clavate, apex subequal to diameter of segments III and IV; III and IV fusiform. Pronotum strongly convex, with a narrow but distinct collar, middle of disc higher than level of head in lateral aspect; in males trapeziform, lateral margins sulcate, humeral angles flared and depressed adjacent to posterior angles, basal margin rounded; in females subquadrate, lateral margins nearly straight, humeral angles weakly flared; scutellum equilateral, base covered by posterior margin of pronotum. *Hemelytron*: polished, constricted at middle of corium, widest across level of cuneus; corium and clavus with predominately golden, scale-like setae, sometimes with silvery and black scale-like setae, intermixed with long, erect, pale or black, bristle-like setae on clavus, corium, and along inner margin of cuneus; deeply punctured on clavus and middle area of corium, with very fine, scattered punctures on cuneus; costal edge in both sexes with a row of tiny black spicules, possibly serving as a stridulatory file (stridulitrum); membrane entire. Ventral surface shiny; ostiolar area pale to whitish, with a raised knob at end of scent channel; glue obscuring base of abdomen (i.e., cannot determine presence of dull or glaucous patch). Legs unmodified, except hind femur in female sometimes thickened on apical half and narrowed at apex; hind tibiae roughened and armed with two rows of tiny spicules, possibly serving as the plectrum during stridulation. Male aperture large, unarmed, genitalia as illustrated (Figs 286–291).

##### Discussion.

As previously noted by Knight (1926), this genus was originally described by Distant (1893) as an aberrant pyrrhocorid. Knight’s (1926) description of *Renodaeus* as having *Ceratocapsus*-like antennae and a head and pronotum like those of *Pilophorus*, convinced him that it did not belong in either Ceratocapsini or Pilophorini; thus, he established the new tribe Renodaeini. Carvalho (1952, 1955, 1958), however, treated Knight’s tribe as a synonym of Pilophorini and Schuh (1995) listed it in synonymy under Orthotylini, along with Ceratocapsini. It is now clear that *Renodaeus* belongs in Ceratocapsini based on the male genitalia.

*Renodaeus* is similar to *Marinonicoris* in overall appearance, including the golden patches and silvery bands of scale-like setae on the hemelytra. It differs, however, in having erect, bristle-like setae on the hemelytra, a row of file-like spicules on the costal margin, and considerably different male genitalia. The left paramere (Figs 286, 289) in species of *Renodaeus* is C-shaped, sometimes with a process arising from the middle, the right paramere (Figs 288, 291) is broad with two diverging lobes, and the phallotheca (Figs 287, 290) is relatively simple with the apex blunt or moderately pointed, whereas in *Marinonicoris myrmecoides*, the left paramere (Fig. 280) is slender with an apical beak-like apex and a slender, apically rounded process at the middle, the right paramere (Fig. 282) is stout and elongate, with a slender, apically bifid arm basally, and the phallotheca (Fig. 281) is broad with three, apical, comb-like processes.

The lack of males precludes positive identification of *Renodaeus ficarius*. Based on the examination of the lectototype and paralectotype females, *Renodaeus ficarius* has a more extensive covering of scale-like setae on the hemelytra, as noted in the key.

##### Key to the species of *Renodaeus*

**Table:** 

1	Clavus and corium entirely covered with golden, scale-like setae; Guatemala	***ficarius* Distant**
–	Only basal half of corium and clavus with golden, scale-like setae	**2**
2	Left paramere (Fig. 289) with apex of right arm curved downward; right paramere (Fig. 291) with three distinct processes; Ecuador	***mimeticus* sp. n.**
–	Left paramere (Fig. 286) with apex of right arm straight; right paramere (Fig. 288) C-shaped; British Guiana, Trinidad	***gibbicollis* Carvalho & Becker**

#### Renodaeus ficarius

Taxon classificationAnimaliaHemipteraMiridae

Distant

Fig. 89

Renodaeus ficarius Distant, 1893: 462 (orig. descrip.); Knight 1926: 56 (note); Carvalho 1952: 83 (as type); Carvalho 1958: 150 (cat.); Carvalho and Becker 1959: 117 (note); Schuh 1974: 285 (lectotype designation); Carvalho and Dolling 1976: 797 (unnecessary lectotype desig.); Schuh 1995: 190 (cat.).

##### Diagnosis.

This species (Fig. 89), known only from females, is distinguished from *Renodaeus gibbicollis* Carvalho and Becker by the larger size, dense covering of golden scale-like setae (intermixed with silvery and black scales) over the entire hemelytra, except the cuneus, the granulate or rugose head, and pronotum that has distinct punctures only on the posterior half.

##### Description.

*Female* (n = 2; lectotype measurements in parentheses): Length 3.28 mm (3.24 mm), width ca. 1.04 mm [one hemelytron broken and glued to card] (1.02 mm). *Head*: Width 0.82 mm (0.78 mm), interocular width 0.44 mm (0.42 mm). *Labium*: Length ca. 1.20 mm (obscured under body on card). *Antenna*: [missing on paralectype; left segments II-IV missing on lectotype]: Segment I, length (0.30 mm); II, (1.00 mm); III and IV fused, teratoid, length (0.60 mm), fusiform. *Pronotum*: Length 0.82 mm (0.76 mm), basal width 0.90 mm (0.88 mm).

*Coloration*: *Head*: Dark reddish brown. *Antenna*: Segment I pale brown (without indication of a red mark); segment II reddish brown. *Pronotum*: Dark reddish brown to nearly fuscous. *Hemelytron*: Brown; cuneus dark reddish brown; membrane translucent, dark, smoky brown. *Ventral surface*: Reddish brown to fuscous; ostiolar area white. *Legs*: Coxae pale, fore coxa more brown; femora dark reddish brown, hind femora pale at apex; tibiae dark reddish brown to fuscous; tarsi and claws brown.

*Structure, texture, and vestiture*: *Head*: Granulate, more transversely rugose along inner margin of eyes and on frons just above clypeus. *Labium*: Extending to ca. middle coxae (obscured under card on paralectotype). *Antenna*: Segment I slender to clavate apex. *Pronotum*: Shiny to semishiny, subquadrate, convex, basal angles weakly flared, basal margin weakly rounded, collar narrow, anterior half across calli granulate, posterior half thickly punctate, posterior angles becoming almost rugose; setae short, recumbent. *Scutellum*: Equilateral, depressed on basal half, apical half weakly convex. *Hemelytron*: Shiny, but obscured by heavy setal covering, strongly constricted on basal half, gradually flared to widest point across cuneus, costal margin with a row of fine, black spicules, clavus and corium densely covered (surface not showing) with tight-fitting, flattened, golden, scale-like setae with smaller patches of silvery scale-like setae at base of clavus, middle of corium (one inside and one outside radial vein), along cuneal fracture and inner corner of cuneus (paracuneus), intermixed with long, erect, bristle-like setae on clavus and corium; at least basal third of clavus and corium deeply punctate (obscured by heavy scale-like covering); cuneus with a few tiny, widely scattered punctures.

*Male*: Unknown.

##### Host.

Unknown.

##### Distribution.

Known only from Guatemala.

##### Discussion.

The female types of this species have the scales on the outer edge of the corium (basal area outside radial vein) rubbed. This triangular area coincides with the position of the peculiar stridulatory spicules along the emboliar margin and may be the result of the hind tibiae rubbing against this surface. Because the male is unknown, I cannot be certain of the identity of this species. Study of males from or near the type locality in Guatemala should help determine if this species is distinct or the senior synonym of *Renodaeus gibbicollis* or *Renodaeus mimeticus*.

##### Type specimens examined.

Lectotype ♀: **GUATEMALA:** Label 1 (circular, with purple margin), “Lectotype”; 2, “Cerro Zunil, 4–5000 ft, Champion” “; 3, “Sp. Figured.”; 4 (handwritten), “Renodaeus ficarius Dist.”; 5 (red, handwritten), “Lectotype Renodaeus ficarius Distant det. R. T. Schuh” (00085459). Paralectotype: 1♀, S. Geronimo, 3000 ft, Champion (both specimens in BNHM).

#### Renodaeus gibbicollis

Taxon classificationAnimaliaHemipteraMiridae

Carvalho & Becker

Figs 286–288

Renodaeus gibbicollis Carvalho & Becker, 1959: 116 (orig. descrip.); Carvalho and Froeschner 1987: 207 (type data and depository); Schuh 1995: 190 (cat.).

##### Diagnosis.

No reliable external characters have been found to distinguish *Renodaeus gibbicollis* from the other species of the genus. The left paramere (Fig. 286, after Carvalho and Becker 1959), lacking a hook at the apex of the right process, and the C-shaped right paramere (Fig. 288, after Carvalho and Becker 1959) will separate it from *Renodaeus mimeticus*.

##### Description.

*Male* (measurements after Carvalho and Becker 1959): Length 2.40 mm, width 0.75 mm. *Head*: Width 0.66 mm, interocular width 0.24 mm. *Labium*: Length (not given). *Antenna*: Segment I length 0.18 mm, II, 0.55 mm, III, 0.35 mm, IV 0.30 mm. *Pronotum*: Length (not given), basal width 0.60 mm.

*Female* (n = 1): Length 2.45 mm, width 0.93 mm. *Head*: Width 0.77 mm, interocular width 0.30 mm. *Labium*: Length 0.77 mm. *Antenna*: Segment I, length 0.19 mm; II, 0.53 mm; III, 0.32 mm, IV missing. *Pronotum*: Length 0.53 mm, basal width 0.67 mm.

*Coloration*: *Head*: Dark brown. *Antenna*: Segment I pale brown, with a red streak on outer margin; segments II and III dark brown; segment IV missing. *Pronotum*: Uniformly dark brown. *Scutellum*: Dark brown. *Hemelytron*: Dark brown; membrane dark smoky brown. *Ventral surface*: Thorax dark brown; abdomen dark reddish brown. *Legs*: Fore and middle coxa reddish brown, hind coxa pale; femora dark reddish brown, apex of hind femur pale; tibiae dark brown, with apical thirds pale yellowish brown; tarsi and claws pale yellowish brown.

*Structure, texture, and vestiture*: *Head*: Shiny, smooth, impunctate. *Labium*: Extending to bases of middle coxae. *Pronotum*: Polished, impunctate; weakly swollen, hind margins flared; with scattered, short, recumbent simple setae. *Scutellum*: With a thick band of silvery, scale-like setae through middle, intermixed with a few long erect simple setae. *Hemelytron*: Shiny throughout; sparsely and finely punctate on apical half of corium; densely and coarsely punctate on clavus and basal half of corium; with two bands of silvery, scale-like setae, one across base of corium and clavus (and extending across scutellum) and one across middle or corium and apex of clavus; area between silvery bands densely covered with golden, scale-like setae; corium (one or two scattered) and clavus (one row of three or four) with few, pale, bristle-like setae. *Ventral surface*: Shiny, with a few scattered erect and semierect, simple setae.

*Male genitalia* (redrawn after Carvalho and Becker 1959): Left paramere (Fig. 286) with a broad quadrate main body with a curving right arm. Right paramere (Fig. 288) C-shaped, upper arm more slender than basal one. Phallotheca (Fig. 287) relatively simple, cone-shaped.

##### Distribution.

Described and known only from British Guiana (now Guyana).

##### Host.

Unknown.

##### Discussion.

Although the female of *Renodaeus gibbicollis* is similar to *Renodaeus mimeticus*, the male genitalia illustrated by Carvalho and Becker are considerably different from those of this new species.

##### Type specimens examined.

The holotype of this species is missing from the USNM collection. Data as given by Carvalho and Becker (1959): Botanical Garden, Georgetown, British Guiana, September 26, 1918, Harold Morrison coll. Allotype ♀ (in poor condition; all parts missing except head and pronotum): Demerara River Bank, +1 mi. from Georgetown, British Guiana, A605, H.M. Morrison Sep. 22 ‘18 (00285739) (USNM).

##### Other specimen examined.

**Guyana** [as British Guiana]: Swept along seashore, 4 mi. east Georgetown, A744, Sept. 30 ‘18, H. M. Morrison, 1♀ (00162230) (USNM).

#### Renodaeus mimeticus

Taxon classificationAnimaliaHemipteraMiridae

Henry sp. n.

http://zoobank.org/8BF9EFEF-F181-455F-B466-6C6126527177

**Figure 1.**
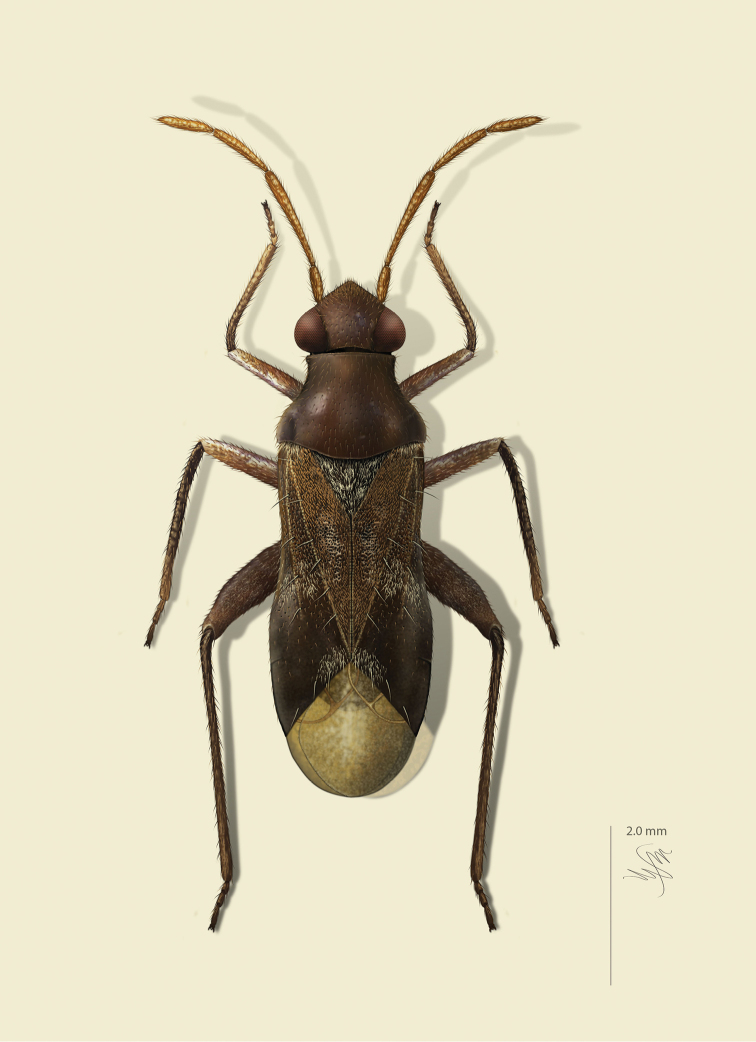
*Renodaeus mimeticus*, adult male.

Figs 1 , 90–92 , 289–291

##### Diagnosis.

At this time, *Renodaeus mimeticus* can be distinguished from *Renodaeus gibbicollis* only by the male genitalia. In *Renodaeus mimeticus*, the left paramere (Fig. 289) has two diverging lobes, the left broader and marginally crenulate, the right paramere (Fig. 291) is C-shaped with a large marginally crenulate process arising from the middle, and the phallotheca (Fig. 290) is apically blunt, whereas in *Renodaeus gibbicollis*, the left paramere (Fig. 286) has a much broader left lobe, the right paramere (Fig. 288) is C-shaped without a process at the middle, and the phallotheca (Fig. 287) is apically acute.

##### Description.

*Male* (n = 3; holotype measurements in parentheses): Length 2.59–2.72 mm (2.72 mm), width 0.86 mm (0.93 mm). *Head*: Width 0.67–0.69 mm (0.69 mm), interocular width 0.24 mm (0.22 mm). *Labium*: Length 0.77–0.80 mm (0.78 mm). *Antennae*: Segment I, length 0.18–0.21 mm (0.19 mm); II, 0.56–0.59 mm (0.56 mm); III, 0.35—0.40 mm (0.35 mm); IV, 0.30–0.32 mm (0.32 mm). *Pronotum*: Length 0.58–0.59 mm (0.59 mm), basal width 0.80 mm (0.83 mm).

*Coloration*: *Head*: Yellowish brown. *Antenna*: Segment I pale brown, with a broad red stripe on dorsal side; segment II brown; segments III and IV reddish brown. *Pronotum*: Dark brown to fuscous, paler anteriorly between calli. *Scutellum*: Fuscous. *Hemelytron*: Dark brown to fuscous; membrane translucent smoky brown. *Ventral surface*: Dark brown, abdominal segments reddish brown. *Ostiolar evaporative area*: Pale brown. *Legs*: Coxae pale brown, fore and hind coxae tinged with red; remainder of legs missing, except two detached, possibly middle legs, femur brown, paler on apical l/3, with a red mark at apex; tibiae reddish brown; tarsi and claws brown.

*Structure, texture, and vestiture*: *Head*: Smooth, shiny, frons with widely scattered, fine, black punctures. *Labium*: Extending to hind coxae. *Antenna*: Segment II slender, gradually thickening to apex; segments III and IV fusiform. *Pronotum*: Polished, smooth, with widely scattered, fine, black punctures, basal angles widely flared, somewhat turned upward and distinctly depressed posteriorly near outer angle, nearly glabrous, with a few scattered, erect, simple setae. *Scutellum*: punctate, with a thick patch of white scale-like setae through middle, intermixed with a thick bunch of long, erect, white, simple setae. *Hemelytron*: Distinctly constricted through middle, widest across cuneus, polished, deeply black punctured on clavus and middle of corium, with scattered, fine punctures on cuneus and apical area of corium; densely set with golden scale-like setae on clavus and middle of corium, with patches of white scale-like setae at base of clavus and through middle of corium (along posterior edge of golden setae) and across apical third of clavus, intermixed with several long, erect, nearly bristle-like setae on clavus and corium and along inner margin of cuneus, cuneus and apical third of corium glabrous.

*Male genitalia*: Left paramere (Fig. 289) with a beak-like left process and a more slender, apically bent right process. Right paramere (Fig. 291) with three large, marginally crenulate processes, the right one most slender (in one specimen, this process is shorter than the stout middle process). Phallotheca (Fig. 290) relatively simple, cone-shaped.

*Female* (n = 4): Length 2.59–2.75 mm, width 0.86–0.93 mm. *Head*: Width 0.66–0.67 mm, interocular width 0.32–0.34 mm. *Labium*: Length 0.78–0.82 mm. *Antenna*: Segment I, length 0.19 mm; II, 0.51–0.54 mm; III, 0.34–0.37 mm; IV, 0.30 mm. *Pronotum*: Length 0.58–0.59 mm, basal width 0.78 mm. Similar to male in coloration, structure, texture, and vestiture.

##### Etymology.

This species is named “*mimeticus*” for its strong ant-like appearance.

##### Host.

Unknown; all specimens were collected by insecticidal fogging of forest canopy.

##### Distribution.

Known only from Orellana Province, Ecuador.

##### Type material.

Holotype ♂: **ECUADOR:** **Orellana Prov.** [as Napo Prov. on label]: Tiputini Biodiversity Station, 216 m, 00E37'55"S, 70E08'39"W, 27 October 1998, T. L. Erwin et al. collectors, insecticidal fogging of mostly bare green leaves, some with covering of lichens or bryophytic plants, Lot 1955, Transect # T-6 (00162231) (Held in trust at USNM). Paratypes: same data as for holotype 5♂♂ (00162232-00162235, 00162240), 4♀♀ (USNM). (00162236-00162239)

#### Zanchisme

Taxon classificationAnimaliaHemipteraMiridae

Kirkaldy

Schizonotus Reuter, 1892: 401 (orig. descrip.). Preoccupied. Type species: *Schizonotus dromedarius* Reuter. Monotypic.
Zanchisme Kirkaldy, 1904: 280 (new name); Carvalho 1958: 153 (cat.); Maldonado 1966: 21 (descrip., key); Carvalho and Schaffner 1974: 58 (review, key); Schuh 1974: 319 (note); Schuh 1995: 204 (cat.). New name for *Schizonotus* Reuter, 1892.
Schistonotellus Reuter, 1905: 32 (new name). Unnecessary new name for *Schizonotus* Reuter, 1892.

##### Diagnosis.

Members of this genus are distinguished by the short labial segment I that does not extend beyond the posterior margin of the head; the round or bulbous head; the distinctly bilobed pronotum, with a distinct constriction separating the strongly convex posterior lobe and the greatly narrowed anterior lobe, latter lobe unarmed or with one to three tubercles; the distinct patches and/or bands of silver, scale-like setae on the hemelytra; and the male genitalia, particularly the left paramere with a distinct beak-like apex, the right paramere with one lateral arm or lobe, and the apically acute phallotheca.

*Zanchisme* is superficially similar to *Zanchismeopsidea* from Argentina in sharing a similarly bilobed pronotum. *Zanchismeopsidea* can be distinguished from *Zanchisme* by the broad, deep gular groove on the undersurface of the head, the distinctly conical scutellum, the proportionately longer posterior lobe of the pronotum (2 times length of anterior lobe versus 1.5 times as long in *Zanchisme*), the lack of a silvery scale-like band at the base of the clavus, and the male genitalia.

##### Description.

Myrmecomorphic. Male and females macropterous. Length of male 3.10–3.78 mm, length of females 2.98–3.17 mm. Head broader than long; posterior margin truncate, distinctly carinate, posterior margins of eyes level with base of vertex; eyes proportionately large, elongate oval, occupying more than three fourths of dorsal head width in males, about half dorsal width in females, laterally occupying nearly entire height in males and about half the height in females; in males, frons flattened from eye to eye, clypeus weakly acute, slightly visible dorsally, in females frons broadly rounded, clypeus rounded, not visible dorsally. Labium extending to middle or hind coxae; segment I short, not extending beyond posterior margin of head, entirely enclosed within oval gular sulcus, buccula enclosing basal half of segment. Antenna with segment I shortest; segment II longest, slender basally, gradually enlarging to apex, diameter subequal to diameters of segments I, III, and IV; segments III and IV evenly thickened, fusiform. Pronotum strongly bilobed; posterior lobe strongly convex, shiny, impunctate, lateral margins straight, posterior margin evenly convex; anterior lobe greatly narrowed, separated from posterior lobe by a distinct constriction, anterior margin with a narrow but distinct collar, ranging from unarmed to armed with one, two, or three blunt to strongly pointed tubercles. Mesoscutum distinct, obliquely angled downward to scutellum; scutellum equilateral, flattened. Hemelytra weakly constricted or concave across middle, widening across apical half of corium and cuneus; dorsum either evenly shiny or shiny on cuneus and outer half of corium and dull on clavus and inner half of corium; with a tight band of silvery scale-like setae across base of corium and cuneus and through scutellum and a loose, wider band of scale-like setae through middle of corium and apex of clavus, intermixed with long, erect, bristle-like setae on scutellum, clavus, and corium; cuneus and membrane fully developed in males and females. Ventral surface shiny; ostiolar area dark with limited evaporative area surrounding auricle, most of metapleural area covered by tiny spicules giving a glaucous sheen; abdomen polished, side of second visible segment with a large, quadrate glaucous patch. Leg slender, unmodified. Male aperture large, open, unarmed; generalized left paramere slender, with a beak-like apex and with a single, sometimes apically bifid process at middle; right paramere stout, with one lateral process; and phallotheca with the apex hooked; endosoma simple, unmodifed.

##### Discussion.

Only four species of this interesting genus are known. No new species were discovered even though numerous collections were examined. Nevertheless, the distribution of several species has been extended significantly. Males and females of the species in this genus are quite similar except for the head, which is much more rounded or bulbous in females. Specimens of *Zanchisme* typically are collected at light, and host associations and feeding habits are unknown.

##### Key to the Species of *Zanchisme*

**Table:** 

1	Hemelytra uniformly shiny on clavus and corium; erect bristle-like setae pale	**2**
–	Hemelytra dull on clavus and at least inner half of corium; erect bristle-like setae black	**3**
2	Anterior lobe of pronotum lacking tubercles, sometimes with a tiny, indistinct median tubercle (Figs 97, 98); Honduras, Mexico	***inermis* Carvalho & Schaffner**
–	Anterior lobe of pronotum with three tubercles, including a more prominent median tubercle (Figs 95, 96); Colombia, Panama, Venezuela	***dromedarius* Reuter**
3	Anterior lobe of pronotum with two large, pointed tubercles (Figs 103, 140); scutellum with a distinct band of silvery, scale-like setae across middle; Mexico to Panama	***mexicanus* Carvalho & Schaffner**
–	Anterior lobe of pronotum with two short, blunt tubercles (Figs 100, 101, 140, 141); scutellum lacking a band of silvery, scale-like setae, or at most, with a few scattered setae; Jamaica	***illustris* Reuter**

#### Zanchisme dromedarius

Taxon classificationAnimaliaHemipteraMiridae

(Reuter)

Figs 95–97 , 292–294

Schizonotus dromedarius Reuter, 1892: 401 (orig. descrip.).
Zanchisme dromedarius : Kirkaldy 1904: 280 (comb. n.); Carvalho 1952: 83 (as type); Carvalho 1958: 153 (cat.); Maldonado 1966: 21, 23 (diag.); Carvalho and Schaffner 1974: 55, 58 (key); Schuh 1995: 204 (cat.).
Schistonotellus dromedarius : Reuter 1905: 32 (comb. n.).

##### Diagnosis.

*Zanchisme dromedarius* (Figs 95–97) is distinguished by the three short tubercles across the anterior lobe of the pronotum, the uniformly shiny hemelytra, the golden scale-like setae on basal half of the corium, and apically pale antennal segment II. This is the only species of the genus with three tubercles on the anterior pronotal lobe.

##### Description.

*Male* (n = 1): Length to apex of membrane 3.20 mm, width 1.02 mm. *Head*: Width 0.82 mm, interocular width 0.29 mm. *Labium*: Length 0.93 mm. *Antenna*: Segment I, length 0.22 mm; II, 0.62 mm; III, 0.40 mm; IV, 0.37 mm. *Pronotum*: Length 0.83 mm, basal width 0.85 mm, anterior width 0.45 mm, width at constriction 0.30 mm.

*Coloration*: Overall yellowish brown. *Head*: Yellowish brown; eyes black. Pronotum: Anterior lobe yellowish brown; posterior lobe yellowish brown to dark brown. *Mesoscutum*: Dark brown. *Scutellum*: Dark brown. *Hemelytron*: Yellowish brown to slightly darker brown at base of corium and on cuneus; membrane smoky brown, paler at base and along apical cuneal margin. *Ventral surface*: Brown to reddish brown. *Ostiolar evaporative area*: Reddish brown. *Legs*: Femora reddish brown, basal fourth of hind femur pale; tibiae reddish brown, apical thirds of middle and hind femora becoming pale yellowish brown; tarsi and claws pale yellowish brown.

*Structure, texture, and vestiture*: *Head*: Impunctate; with scattered semierect simple setae. *Labium*: Extending to middle of mesosternum just before middle coxae. *Pronotum*: Bilobed, narrowed anterior lobe with three, short tubercles, the middle one most prominent; posterior lobe strongly convex; anterior lobe impunctate; posterior lobe very finely punctate, appearing impunctate in certain light; with only scattered semierect simple setae. *Scutellum*: Finely transversely rugose. *Hemelytron*: Shiny, finely punctate, punctures heavier across pubescent patches and on cuneus; with two loose bands of silvery, scale-like setae, one across base of clavus and through middle of scutellum and one across middle of corium and onto adjacent clavus, intermixed on clavus and apical margin of corium with long, erect and semierect pale, nearly bristle-like setae and more generally scattered, shorter, pale simple setae. *Ventral surface*: Shiny.

*Male genitalia*: Left paramere (Fig. 292) with slender beak-like apex and a quadrate process at middle. Right paramere (Fig. 294) with main stem and primary arm forming a shallow C-shape, and with one accessory spine behind arm. Phallotheca (Fig. 293) with a relatively stout, right-curving apical process and a more narrow, left-curving process on opposite side.

*Female* (n = 1): Length to apex of membrane 2.98 mm, width 1.06 mm. *Head*: Width 0.78 mm, interocular width 0.40 mm. *Labium*: Length 0.93 mm, extending to middle of mesosternum. *Antenna*: Segment I, length 0.24 mm; II, 0.56 mm; III, 0.40 mm; IV, 0.38 mm. *Pronotum*: Length 0.80 mm, basal width 0.83 mm, anterior width 0.48 mm, width at constriction 0.27 mm.

Similar to male in overall shape and development of tubercles on anterior pronotal lobe, but slightly shorter and stouter, posterior pronotal lobe in lateral view more compressed from front to back, and hemelytral membrane slightly less elongate.

##### Host.

Unknown.

##### Distribution.

Described from and previously known only from Venezuela. Colombia and Panama are new country records.

##### Type material examined.

Holotype ♀: *Zanchisme dromedarius* Reuter (♀): “Caracas”; [handwritten, white] “Zanchisme dromedarius sp. n. [printed] O. M. Reuter det.”; [pale blue label, printed] “Mus. Zool. H:fors Spec. typ. No. [handwritten] “10025 Zanchisme dromedarius Reut.” (00099731).

##### Other specimens examined.

**COLOMBIA:** **Magdalena:** Santa Marta, Darlington, 03 May 1900, collector unknown, 1♀ (00286333) (AMNH). **PANAMA:** **Panama:** El Cermeno, Apr 1939–May 1939, Zetek, 1♀ (00285959) (USNM). **VENEZUELA:** **Distrito Federal:** Caracas, 10.5EN, 66.9EW, 1700, collector unknown, 1♀ (00099731) (MZH). **Guanare:** R. Portuguesa, 3 Jan. 1965, C. Bordan, 1♂ (UNAM).

#### Zanchisme illustris

Taxon classificationAnimaliaHemipteraMiridae

Reuter

Figs 100 , 101 , 295–297

Zanchisme illustris Reuter, 1907: 11 (orig. descrip.); Van Duzee 1907: 28 (note); Carvalho 1958: 153 (cat.); Maldonado 1966: 22 (descrip., key); Carvalho and Schaffner 1974: 55, 58 (key); Carvalho 1990: 218 (descrip.); Schuh 1995: 204 (cat.).

##### Diagnosis.

This species (Figs 100, 101) is distinguished by the two short tubercles on either side of the anterior lobe of the pronotum, the dull hemelytron with only the costal (emboliar) margin and cuneus shiny or polished, and the male genitalia.

##### Description.

*Male* (n = 5): Length to apex of membrane 3.10–3.78 mm, width 0.90–1.02 mm. *Head*: Width 0.70–0.75 mm, interocular width 0.18–0.21 mm. *Labium*: Length 0.98–1.10 mm. *Antenna*: Segment I, length 0.21–0.24 mm; II, 0.77–0.83 mm; III, 0.35–0.45 mm; IV, 0.37–0.49 mm. *Pronotum*: Length 0.72–0.80 mm, basal width of posterior lobe 0.86–0.91 mm, width of anterior lobe 0.34–0.35 mm, width of constriction 0.30–0.35 mm.

*Coloration*: *Head*: Yellowish brown to dark brown; eyes silvery, tinged with red. *Antenna*: Segment I pale yellow, with a narrow red (sometimes brownish) line on ventral surface and a short line on apical half of dorsal surface; segment II yellowish brown, apex more whitish, dorsal surface at base dark brown; segment III brown, apex more whitish; segment IV uniformly brown. *Pronotum*: Posterior lobe reddish brown to dark brown; anterior lobe yellowish brown to dark brown. *Mesoscutum*: Yellowish brown. *Scutellum*: Dark brown, with apex yellowish. *Hemelytron*: Yellowish brown to brown; costal margin (embolium) and cuneus reddish brown to dark brown; membrane dark brown, pale within areoles. *Ventral surface*: Thoracic area yellowish brown; abdomen yellowish brown, becoming darker brown distally toward genital capsule. *Ostiolar evaporative area*: White, central knob slightly embrowned. *Legs*: Fore coxae brown, middle and hind coxae white; fore and middle femora yellowish brown, darker dorsally, hind femur dark brown, pale at base and extreme apex; tibiae brown to reddish brown, pale on apical one third to one fourth; tarsi and claws yellowish.

*Structure, texture, and vestiture*: *Head*: Shiny, impunctate; interocular area smooth, frons becoming transversely rugose. *Labium*: Extending to middle of mesosternum, not quite reaching bases of middle coxae. *Pronotum*: Bilobed, narrowed anterior lobe with two, short, blunt tubercles, one on either side, middle smooth, shiny; posterior lobe strongly convex, polished, impunctate; anterior lobe shiny, alutaceous; with only sparsely scattered, recumbent, simple setae. *Mesoscutum*: Shiny, impunctate. *Scutellum*: Impunctate, weakly rugose; with four or five long, erect, setae. *Hemelytra*: Dull over most of corium and clavus, polished along costal margin (embolium) and cuneus; clavus with three patches of silvery scale-like setae, longest one across base, one at middle, and a small one on apex; corium with two patches of silvery scale-like setae, larger one at middle and smaller one more basal; intermixed with sparsely placed, long, erect, dark brown, bristle-like setae. *Ventral surface*: Shiny; abdomen with short, semierect, simple setae.

*Male genitalia*: Left paramere (Fig. 295) with a beak-like apical process and a short, slender, apically rounded process at middle. Right paramere (Fig. 297) with a slender main stem and one long, curved, marginally crenulate, lateral arm. Phallotheca (Fig. 296) evenly slender, with a slender, curved apical hook.

*Female* (n = 2): Length to apex of membrane 2.98–3.14 mm, width 0.90–0.91 mm. *Head*: Width 0.66–0.67 mm, interocular width 0.30–0.32 mm. *Labium*: Length 0.98–0.99 mm, extending slightly past middle of mesosternum. *Antenna*: Segment I, length 0.19–0.20 mm; II, 0.56–0.59 mm; III, 00.29–0.35 mm; IV, 0.35 (missing) mm. *Pronotum*: Length 0.64–0.69 mm, basal width of posterior lobe 0.78–0.80 mm, width of anterior lobe 0.32–0.34 mm, width of constriction 0.30–0.32 mm. Similar to male in coloration and general appearance, with head more rounded and the interocular area proportionately wider.

##### Host.

Unknown.

##### Distribution.

Known only from Jamaica.

##### Discussion.

Maldonado (1966) noted the association of this species with an ant (*Crematogaste* r sp.) in St. Catherine Parish, Jamaica, and reported it from *Ipomoea fistulosa* Mart DC ex Choisy [Convolvulaceae] in Kingston.

##### Type material examined.

Holotype: *Zanchisme illustris* Reuter (abdomen missing, but appears to be a ♂ [slender appearance]): “Mandev’le, Ja., Apr. 06”; “VanDuzee Collector”; “48”; [white, handwritten] “Zanchisme illustris sp. n. [printed] O. M. Reuter det.”; [pale blue label, printed] Mus. Zool. H:fors Spec. typ. No. [handwritten] 10026 Zanchisme illustris Reut”; (00099732) (ZMUH). Specimen in poor condition; abdomen missing, both hemelytra were detached and loose in unit tray [I glued both to point adjacent to specimen].

##### Other specimens examined.

**JAMAICA:** **Clarendon Parish:** Alston, 2000’ (610 m), 25 Apr 1972–29 Jul 1972, C. Crickett, 7♂♂ (00285975-00285981) (USNM). **Kingston Parish:** Kingston, Jul 1961, J. Maldonado Capriles, 1♂ (00285960) (USNM). **Manchester:** 4 mi. south of Christiana, Jul 1961, J. Maldonado Capriles, 1 (?sex) (00285961) (USNM). Mandeville, 18.03378EN, 77.50012EW, 23 Aug 1960, J. Howard Frank, light trap, 1♂ (00285965) (USNM). Mandeville, 18.03378EN, 77.50012EW, 618 m, 1700, collector unknown, 1 (?sex) (00099732) (MZH). Mandeville, 18 May 1969, R. E. Woodruff, 1♂ (FSCA). **Portland Parish:** Green Hills Inst. Jamaica, 13 Dec 1969, E.G. Farnworth, light trap, 1♂ (00285964) (USNM). Port Antonio, 18.17435EN, 76.45018EW, 1900, A. E. Wight, 1♂ (00286332) (AMNH). **Saint Andrew:** Hardwar Gap, 18.08774EN, 76.71017EW, 1219 m, 25 Aug 1966, H. F. Howden, 1♂ (00375003) (CNC). **Saint Catherine:** 1 mile N of Old Harbour, 17.95551EN, 77.10789EW, 21 Oct 1957, T. H. Farr, taken in association with *Crematogaster* sp., 1♂ (00285973) (USNM). Guanaboa Vale, 01 Aug 1958, T.H. Farr, 1♂ (00285974) (USNM). **St. Andrew Parish:** Halfway Tree, 22 Feb 1959, T. H. Farr, light trap, 2 nymphs (00285969, 00285970), 1♂ (00285971), 1♀ (00285972) (USNM); Jul 1968, J. Maldonado Capriles, light trap, 2♀♀ (00285966, 00285967), 1♂ (00285968) (USNM). Holywell Forest Camp, 1219 m, 16 Dec 1971, M. Winegar, light trap, 1♂ (00285963) (USNM); 20 Jun 1972, M. Winegar, light trap, 1♂ (00285962) (USNM). Irishtown, 06 Jul 1971, J.A. Slater, R. M. Baranowsk, & J. E. Harrington, 2♂♂ (00286330, 00286331) (AMNH). **St. Catherine Parish:** near Fort Clarence, 07 Dec 1975, G. F. Hevel, 1♂ (00285982) (USNM). **St. Thomas Parish:** Whitfield Hall, 18.04882EN, 76.62191EW, 1234 m, 27 Jul 1966, Howden and Becker, 1♀ (00375006) (CNC). **Westmoreland:** Cornwall Mountain, 18.28583EN, 78.03493EW, 283 m, 18 Aug 1966, Howden and Becker, 2♀♀ (00375004, 00375005) (CNC).

#### Zanchisme inermis

Taxon classificationAnimaliaHemipteraMiridae

Carvalho & Schaffner

Figs 98 , 99 , 298–300

Zanchisme inermis Carvalho & Schaffner, 1974: 57 (orig. descrip.); Carvalho and Froeschner 1987: 219 (type list); Schuh 1995: 204 (cat.).

##### Diagnosis.

This species (Figs 98, 99) is distinguished by the lack of tubercles on the anterior lobe of the pronotum, the uniformly shiny hemelytra, with a band of silvery, scale-like setae across the base of the clavus and corium and a loose patch of silvery scale-like setae through the middle of the corium and distal two thirds of the clavus, and the male genitalia, especially the left paramere (Fig. 298).

##### Description.

*Male* (n = 2; holotype measurements in parentheses): Length to apex of membrane 3.42 mm (3.30 mm), width 0.98 mm (0.99 mm). *Head*: Width 0.74 mm (0.77 mm), interocular width 0.18 mm (0.24 mm). *Labium*: Length 0.88 mm (0.94 mm). *Antenna*: Segment I, length 0.22 mm (0.24 mm); II, 0.66 mm (0.61 mm); III, 0.40 mm (0.40 mm); IV, 0.42 mm (0.35 mm). *Pronotum*: Length 0.74 mm (0.75 mm), basal width of posterior lobe 0.94 mm (0.91 mm), width of anterior lobe 0.35 mm (0.37 mm), width of constriction 0.34 mm (0.32 mm).

*Coloration*: *Head*: Brown to fuscous. *Antenna*: Segment I yellowish brown, with a narrow red line on dorsal and ventral surface; segment I brown, with a short dark brown line dorsally at base; segment III and IV dark brown. *Pronotum*: Dark brown, base of anterior lobe sometimes slightly paler brown. *Mesoscutum*: Brown. Scutellum brown, apex slightly paler. *Hemelytron*: Uniformly dark brown. *Ventral surface*: Thoracic segments dark brown; abdomen dark brown, glaucous stridulatory patch pale. *Ostiolar evaporative area*: Dark reddish brown. *Legs*: Fore coxae brown, middle and hind coxae whitish; fore femur dark brown, middle and hind femora dark brown with bases pale yellow or whitish; tibiae dark brown, with apices paler yellow; tarsi and claws yellowish.

*Structure, texture, and vestiture*: *Head*: Shiny, impunctate, frons weakly alutaceous; with scattered erect and semierect, simple setae. *Labium*: Extending to posterior margin of mesosternum, nearly to bases of middle coxae. *Pronotum*: Bilobed, narrowed anterior lobe lacking tubercles, anterior margin with a somewhat shallow, transverse ridge; posterior lobe strongly convex; shiny, polished, impunctate; with scattered, short, recumbent simple setae. *Mesoscutum*: Shiny, impunctate. *Scutellum*: Shiny, impunctate; with a brown band of silvery, scale-like setae through middle. *Hemelytron*: Uniformly shiny; with a band of silvery, scale-like setae across base of corium and clavus and a wide patch of less dense scale-like setae through apical two thirds of clavus and inner middle half of corium, sparsely intermixed with short, recumbent, simple setae and long, erect, bristle-like setae. *Ventral surface*: Shiny; abdomen with scattered and irregular rows of semierect, simple setae.

*Male genitalia*: Left paramere (Fig. 298) with an elongate beak-like process with opposite side extended into a long slender process; middle with a small, bilobed, wing-like process. Right paramere (Fig. 300) with a slender mainstem and one short lateral arm curved upward. Phallotheca (Fig. 299) stout, with a long, slender apical process and a shorter, more stout, recurved, subapical process.

*Female* (n = 2): Length to apex of membrane 2.94–3.10 mm, width 1.02–1.06 mm. *Head*: Width 0.70–0.74 mm, interocular width 0.32–0.34 mm. *Labium*: Length 0.98–1.02 mm, extending slightly past middle of mesosternum. *Antenna*: Segment I length 0.22–0.24 mm; II, 0.56 mm (one missing); III, 0.38 mm (one missing); IV, 0.42 mm (one missing). *Pronotum*: Length 0.69–0.70 mm, width of posterior lobe 0.85–0.91 mm, width of anterior lobe 0.30–0.32 mm, width of constriction 0.34–0.35 mm.

##### Host.

One specimen from Mexico was taken on *Mimosa pigra* L. [Fabaceae].

##### Distribution.

Previously known only from Oaxaca, Mexico. Guerrero is a new Mexican state record. Costa Rica and Honduras are new country records.

##### Type material examined.

Holotype ♂: **MEXICO:** **Oaxaca:** La Ventosa, 72 mi. E. Oaxaca, VII–21–63, J. Doyen collector (CAS).

##### Other specimens examined.

**COSTA RICA:** **Guanacaste:** South Canas, 25 Feb 1989–08 Mar 1989, F. D. Parker, 1♀ (00286353) (USU). Com., 13 k N Comayagua Hwy. 1, 18 Jul. 1977, O’Brien and Marshall, 1♂, 1♀(USNM). Guanacoste, 14 km S Cañas, 1–7 Feb. 1991, F. D. Parker, 1♂ (USU). **MEXICO:** **Guerrero:** Barra Vieja, 05 Mar 1986, H. Miranda, *Mimosa pigra* (Fabaceae), 1♀ (00285983) (USNM). **Oaxaca:** Puerto Escondido, 22 Aug 1975, H. Brailovsky, 1♂ (00286357) (UNAM).

#### Zanchisme mexicanus

Taxon classificationAnimaliaHemipteraMiridae

Carvalho & Schaffner

Figs 102–104 , 301–303

Zanchisme mexicanus Carvalho & Schaffner, 1974: 55 (orig. descrip.); Carvalho and Froeschner 1987: 219 (type list); Schuh 1995: 204 (cat.).

##### Diagnosis.

*Zanchisme mexicanus* (Figs 102–104) is recognized by the two well-developed, sharp spines on the anterior lobe of the pronotum, the partially dull and partially shiny hemelytra, the black bristle-like dorsal setae, and the male genitalia.

##### Description.

*Male* (n = 5; holotype measurements in parentheses): Length to apex of membrane 3.20–3.62 mm (3.17 mm); width 0.98–1.17 mm (0.96 mm). *Head*: Width 0.74–0.77 mm (0.69 mm), interocular width 0.30–0.32 mm (0.32 mm). *Labium*: Length 0.96–0.98 mm (0.88 mm). *Antenna*: Segment I, length 0.21–0.24 mm (0.21 mm); II, 0.64–0.67 mm (0.52 mm); III, 0.40–0.42 mm (0.32 mm); IV, 0.35–0.40 mm (0.32 mm). *Pronotum*: Length 0.80–0.85 mm (0.75 mm), basal width of posterior lobe 0.91–1.07 mm (0.93 mm), width of anterior lobe 0.48–0.53 mm (0.48 mm), width of constriction 0.34–0.38 mm (0.34 mm).

*Coloration*: *Head*: Reddish brown to dark brown. *Antenna*: Pale yellowish brown, with a narrow red line along ventral surface and short, apical red line on dorsal surface; segment II yellowish brown to brown, with a short, narrow red to brown line dorsally at base; segments III and IV dark brown. *Pronotum*: Yellowish brown, dark brown, to dark reddish brown, anterior lobe sometimes paler than posterior lobe. *Mesoscutum*: Brown to dark reddish brown. *Scutellum*: Brown to dark reddish brown. *Hemelytron*: Corium inside radial vein and clavus yellowish brown; area outside radial vein and cuneus brown to dark brown; membrane dark smoky brown, paler or whitish inside areoles. *Ventral surface*: Thoracic area brown to reddish brown; abdomen dark reddish brown to fuscous. *Ostiolar evaporative area*: Largely white, with raised central area of auricle red. *Legs*: Fore coxa brown, middle and hind coxae pale or whitish; fore femur dark brown, middle and hind femora dark brown on apical half, pale or yellowish brown basally; tibiae dark brown, pale or yellow at apices; tarsi and claws pale yellow.

*Structure, texture, and vestiture*: *Head*: Shiny, impunctate. *Labium*: Extending to middle of mesosternum. *Pronotum*: Bilobed, narrowed anterior lobe with two, long, sharply pointed tubercles, one on either side, middle smooth, shiny; posterior lobe strongly convex; shiny, impunctate, polished; with scattered, short, recumbent, simple setae. *Mesoscutum*: Shiny, impunctate, with a few short, simple setae. *Scutellum*: Dull, narrowly shiny along base, impunctate; with a broad band of silvery scale-like setae across middle. *Hemelytron*: Dull on clavus and corium inside radial vein, shiny on cuneus and corium outside radial vein; with wide band of silvery, scale-like setae across base of clavus and a wider patch of less dense silvery scale-like setae across middle of corium and apical third of clavus, intermixed on dull surface, with evenly placed, long, erect, bristle-like setae. *Ventral surface*: Shiny; abdomen with scattered and irregular rows of short, recumbent, simple setae.

*Male genitalia*: Left paramere (Fig. 301) with an elongate, beak-like process apically, and a smaller beak-like process at middle facing opposite direction. Right paramere (Fig. 303) with a slender main stem and one marginally crenulate lateral arm. Phallotheca (Fig. 302) evenly slender, with a slender, curved apical hook.

*Female* (n = 3): Length to apex of membrane 2.94–3.17 mm, width 1.02–1.04 mm. *Head*: Width 0.70–0.75 mm, interocular width 0.42–0.43 mm. *Labium*: Length 0.83–1.01 mm. *Antenna*: Segment I, length 0.19–0.24 mm; II, 0.50–0.59 mm; III, 0.32–0.37 mm; IV, 0.27–0.35 mm. *Pronotum*: Length 0.70–0.75 mm, basal width of posterior lobe 0.84–0.86 mm, width of anterior lobe 0.48–0.50 mm, width of constriction 0.32–0.37 mm.

##### Host.

Unknown.

##### Distribution.

Previously known only from Oaxaca, Mexico. New state records for Mexico are Chiapas, Michoacan, and Puebla. Costa Rica, El Salvador, Honduras, and Panama are new country records.

##### Type material examined.

Holotype ♂: **MEXICO:** **Oaxaca:** 11.6 miles west of Jalapa del Marques, July 12, 1971, taken at light, Clark, Murray, Hart, Schaffner (00071405) (USNM). Paratypes: **MEXICO:** **Oaxaca:** 11.6 mi W of Jalapa del Marques, 16.49992EN, 95.6426EW, 12 Jul 1971, Clark, Murray, Hart, Schaffner, light trap, 1♀ (00285984) (USNM).

##### Other specimens examined.

**BELIZE:** **Cayo:** Rio On, 22 mi. S. Georgeville, 20 Aug 1977, O’Briens & Marshall, 1♂ (00138580) (AMNH). **COSTA RICA:** **Guanacaste:** 14 km S of Canas, 10.318EN, 85.045EW, 09 Sep 1989–15 Sep 1989, F. D. Parker, 1♂ (00286334) (USNM); 11 Jan 1990–31 Jan 1990, F. D. Parker, 1♂ (00286337) (USU); 15 Feb 1990–24 Feb 1990, F. D. Parker, 1♀ (00286343) (USNM); 23 Feb 1990–28 Feb 1990, F. D. Parker, 2♀♀ (00286339) (USU) (00286340) (USNM); 13 Mar 1990–21 Mar 1990, F. D. Parker, 1♀ (00286373) (USU); 23 Mar 1990–31 Mar 1990, F. D. Parker, 1♀ (00286341) (USU); 29 Aug 1990, F. D. Parker, 1♂ (00286336) (USU); 08 Feb 1991–15 Feb 1991, F. D. Parker, 1♀ (00286370), 1♂ (00286371) (USNM); 16 Mar 1991–23 Mar 1991, F. D. Parker, 1♂ (00286335) (USNM); 01 Mar 1992–15 Mar 1992, F. D. Parker, 1♂ (00286338) (USU). South Canas, 22 Feb 1989–24 Feb 1989, F. D. Parker, 1♀ (00286372) (USU). **San Jose:** Escazu, 9.9166EN, 84.133EW, 22 Jul 1989–05 Aug 1989, F. D. Parker, 1♂ (00286349) (USU). **EL SALVADOR:** **La Libertad:** La Pena, Sep 1961, B. Caillejas, 1♂ (00285985) (USNM). **Santa Ana:** Santa Ana, 13.9942EN, 89.5597EW, 650 m, 16 Jul 1975, N. L. H. Krauss, 1♂ (00286358) (USNM). Santa Tecla la Pena, Sept. 1961, B. Calliejes, 1♂ (USNM). **HONDURAS:** **Atlantida:** La Ceiba, 100 m, Jun 1981, N. L. H. Krauss, 1♂ (00138579) (AMNH). **MEXICO:** **Oaxaca:** 28 mi E of La Ventosa, 16.53555EN, 94.54598EW, 58 m, 25 Jun 1969, Bright and Campbell, 1♀ (00375018) (CNC). Huajuapan, 17.8EN, 97.76667EW, 1633 m, 25 Aug 1969, L. A. Kelton, 5♂♂ (00375007-00375011), 6♀♀ (00375012- 00375017) (CNC). Chiapas, 4 mi. E. Cintalapa, July 11, 1991, at light R. W. Jones, 1♂ (TAMU). Michoacan, 10 mi. South Uruapan, July 29, 1988, Ferreira and Schaffner, 1♂ (TAMU). Michoacan, Coahuayana, 11–II–83, E. Barrera, 1♀(UNAM). Oaxaca, Puento Escondido, 22–VII–75, W. Brailovsky, 1♀(UNAM). Puebla, Acatlan, 16–IX–80, H. Brailovsky, 5th instar (TAMU). **PANAMA:** **Chiriqui:** Palm Beach near San Carlos, 07 Sep 1952, F. S. Blanton, 1♂ (00285987) (USNM). **Cocle:** Agua Dulce, 07 Aug 1951, Blanton, 1♂ (00285986) (USNM). none, 20 Nov 1952, F. S. Blanton, 1♂ (00286354) (USNM). **Panama:** Farfan Beach, 15 Jul 1976, Wayne E. Clark, 1 (?sex) (00285988), 1♀ (00285989) (USNM).

#### Zanchismeopsidea

Taxon classificationAnimaliaHemipteraMiridae

Henry gen. n.

http://zoobank.org/E101C0D7-F8AA-4475-8419-F035175FF7DE

##### Type species:

*Zanchismeopsidea diegoi* Henry, sp. n.

##### Diagnosis.

*Zanchismeopsidea* is distinguished by the distinctly narrow anterior lobe of the pronotum, armed with two large, apically blunt tubercles; a distinct collar; strongly convex posterior pronotal lobe; conical scutellum; the polished hemelytron with a loose band of narrow, white, scale-like setae across the base of the corium and onto the middle of the clavus; and the male genitalia.

##### Description.

Length 3.62 mm. Overall elongate, slender, subparallel, macropterous. Head triangular, 1.5 times broader than long; basal margin truncate; eyes prominent, coarsely faceted; frons flattened, transversely rugose. Pronotum bilobed, shiny; anterior lobe narrowed, width about half width of posterior lobe, armed with two, stout, bluntly pointed tubercles, collar wide, flattened; posterior lobe strongly convex, about two times wider than long, impunctate (or only very finely and indistinctly so). Mesoscutum broadly exposed, flat, shiny. Scutellum equilateral, shiny, bluntly conical. Hemelytron shiny, impunctate, with a broad loose band of silvery, scale-like setae across base of corium and clavus, intermixed with a few long, erect, bristle-like setae on clavus and apical margin of corium; membrane with two closed cells. Genitalia relatively simple; left paramere bilobed with left lobe apically truncate and right lobe more slender; right paramere with simple trunk and one slender, recurved arm and a small spine on backside; and phallotheca with a distinct beak-like apical process.

##### Etymology.

The name of this new genus is formed from the combination of the generic name *Zanchisme* and the suffix from the generic name *Pilophoropsidea*, and is used to denote the close relationship of the three genera. The gender is feminine.

##### Discussion.

This interesting new genus shares characteristics with both *Zanchisme* and *Pilophoropsidea*. The distinctly constricted anterior lobe of the pronotum is shared with *Zanchisme*, whereas the shiny hemelytron with a loose patch of white, scale-like setae across the base of the pronotum and through the middle of the clavus is most similar to the setal plan of species of *Pilophoropsidea*. It can be distinguished from both genera by the combination of characters listed above and the structure of the male genitalia.

#### Zanchismeopsidea diegoi

Taxon classificationAnimaliaHemipteraMiridae

Henry sp. n.

http://zoobank.org/030FF2D1-7B42-4083-9E11-49AEBC8A5D93

Figs 105 , 106 , 304–306

##### Diagnosis.

*Zanchismeopsidea diegoi* (Figs 105, 106) is recognized by the elongate, subparallel body; bilobed pronotum with the anterior lobe narrowed and armed with two large tubercles, the strongly convex posterior lobe; the exposed mesoscutum, the prominent scutellum, and the shiny hemelytron with a broad, loose band of silvery, scale-like setae across the basal half of the clavus and corium.

##### Description.

*Male* (n = 2, holotype measurements in parentheses): Length to apex of membrane 3.42 mm (3.62 mm), width 1.05 mm (0.94 mm). *Head*: Length 0.34 mm (0.48 mm), width 0. 74 mm (0.78 mm), interocular width 0.26 mm (0.27 mm). *Labium*: Length about 1.00 mm (1.25 mm), broken and imbedded in glue. *Antenna*: Segment I, length 0.21 mm (0.24 mm); II, 0.58 mm (0.62 mm); III 0.37 mm (0.49 mm); IV, 0.42 mm (0.43 mm). *Pronotum*: Length 0.63 mm (0.75 mm), length of anterior lobe 0.21 mm (0.27 mm), length of posterior lobe 0.42 mm (0.48 mm); anterior lobe width 0.47 mm (0.48 mm), posterior lobe width at base 0.95 mm (1.02 mm).

*Coloration*: *Head, pronotum, mesoscutum, and scutellum*: Shiny brown. *Antenna*: Segments I and II dark brown; segments III and IV nearly black. *Hemelytron*: Shiny brown, paler yellowish brown on inner half of cuneus; membrane fumate or blackish gray, white at base between bases of cunei and narrowly along apical half of cuneal margin. *Ventral surface*: Shiny brown on thorax to more reddish brown on abdomen. *Ostiolar evaporative area*: White. *Legs*: Fore coxae brown, middle coxae brown at base, paler yellowish brown apically; hind coxae yellowish brown, darker brown at base; remaining segments missing.

*Structure, texture, and vestiture*: *Head*: Surface shiny to semishiny, frons transverely rugose, vertex finely granulate; with a few scattered semierect pale setae, interspersed with a few short, recumbent setae. *Labium*: Extending to middle coxae or slightly beyond. *Pronotum*: Bilobed; anterior lobe with two distinct conical tubercles; posterior lobe shiny, impunctate; clothed with short, sparse, recumbent simple setae. *Mesoscutum*: Broadly exposed, shiny, impunctate. *Scutellum*: Shiny, impunctate, bluntly conical, with a few long, erect, simple setae. *Hemelytron*: Shiny, impunctate; with a broad, loose band of silvery, scale-like setae across base of corium and basal half of clavus, intermixed with three or four long, erect, white bristle-like setae on clavus and four more along apical margin of corium, and very short, sparse, fine, simple setae on clavus, corium, and cuneus. *Ventral surface*: Shiny, with scattered, recumbent, simple setae on abdomen.

*Male genitalia*: Genital capsule aperture with one long slender spine arising at 2:00 o’clock position. Left paramere (Fig. 304) bilobed, with left lobe apically blunt and right lobe more tapered. Right paramere (Fig. 306) with simple main trunk tapering apically, with one down-curved lateral arm and a short spine on back side. Phallotheca (Fig. 305) with an apical beak-like process curving downward caudally.

*Female*: Unknown.

##### Etymology.

I am pleased to name this new species in honor of my good friend and colleague, Diego Carpintero, author of numerous publications on Argentinian Heteroptera, especially Anthocoridae and Miridae, and collector of the only two known specimens of *Zanchismeopsidea diegoi*.

##### Host.

Unknown; holotype and paratype taken at light.

##### Distribution.

Known only from the state of Santiago Del Estero, Argentina.

##### Type material.

Holotype ♂: **ARGENTINA:** S[antiago] D [el] Estero, Añatuya (Luz), III–99, D. J. Carpintero (MACN). Paratype: Same data as for holotype, 1♂ (USNM).

**Figure 2.**
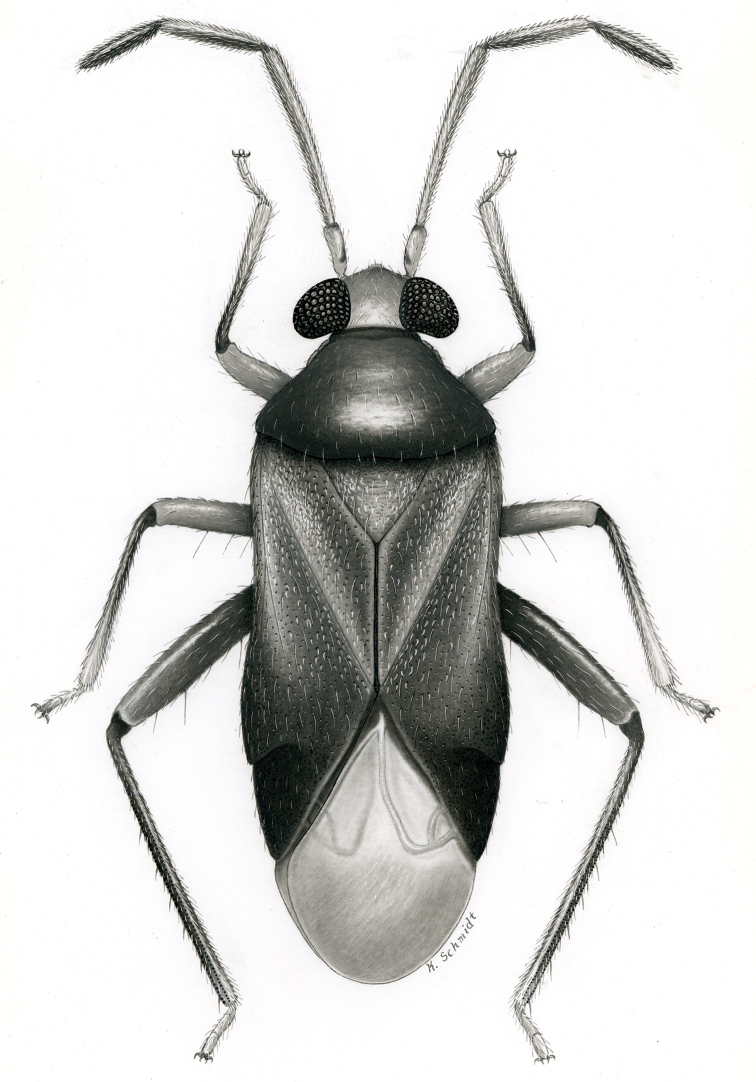
*Ceratocapsidea balli*, adult male.

**Figures 3–10.**
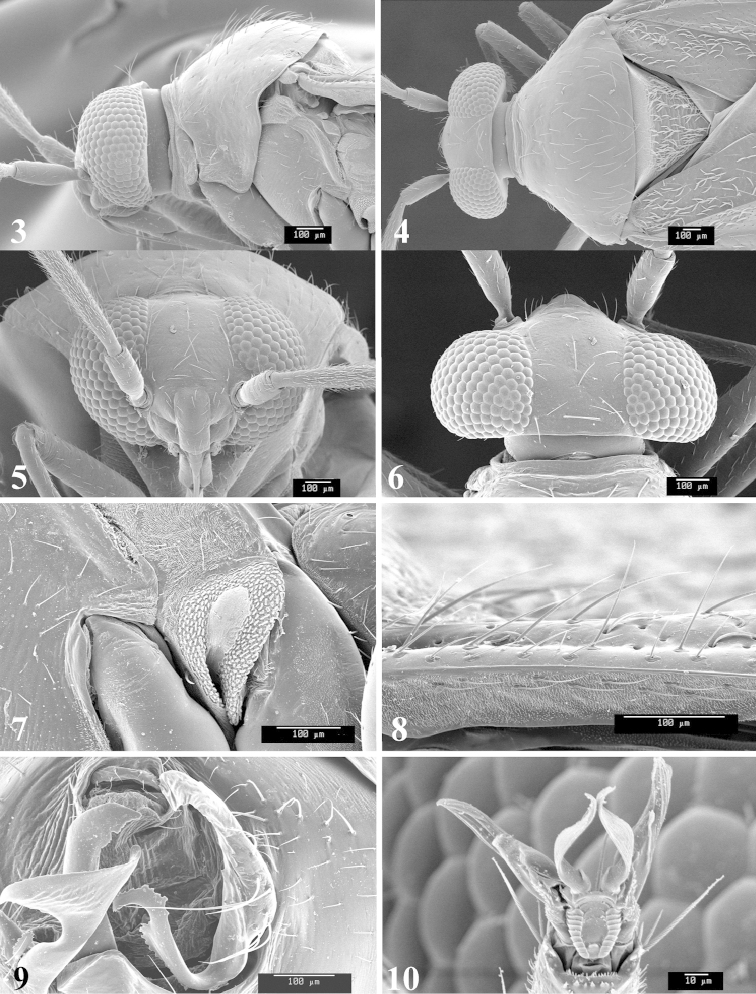
SEM photomicrographs of *Ceratocapsidea balli*, male. **3** head and pronotum, lateral aspect **4** head and pronotum, dorsal aspect **5** head, frontal aspect **6** head, dorsal aspect **7** ostiolar evaporative area **8** lateral margin of hemelytron **9** genital capsule, caudal aspect **10** pretarsus.

**Figures 11–16.**
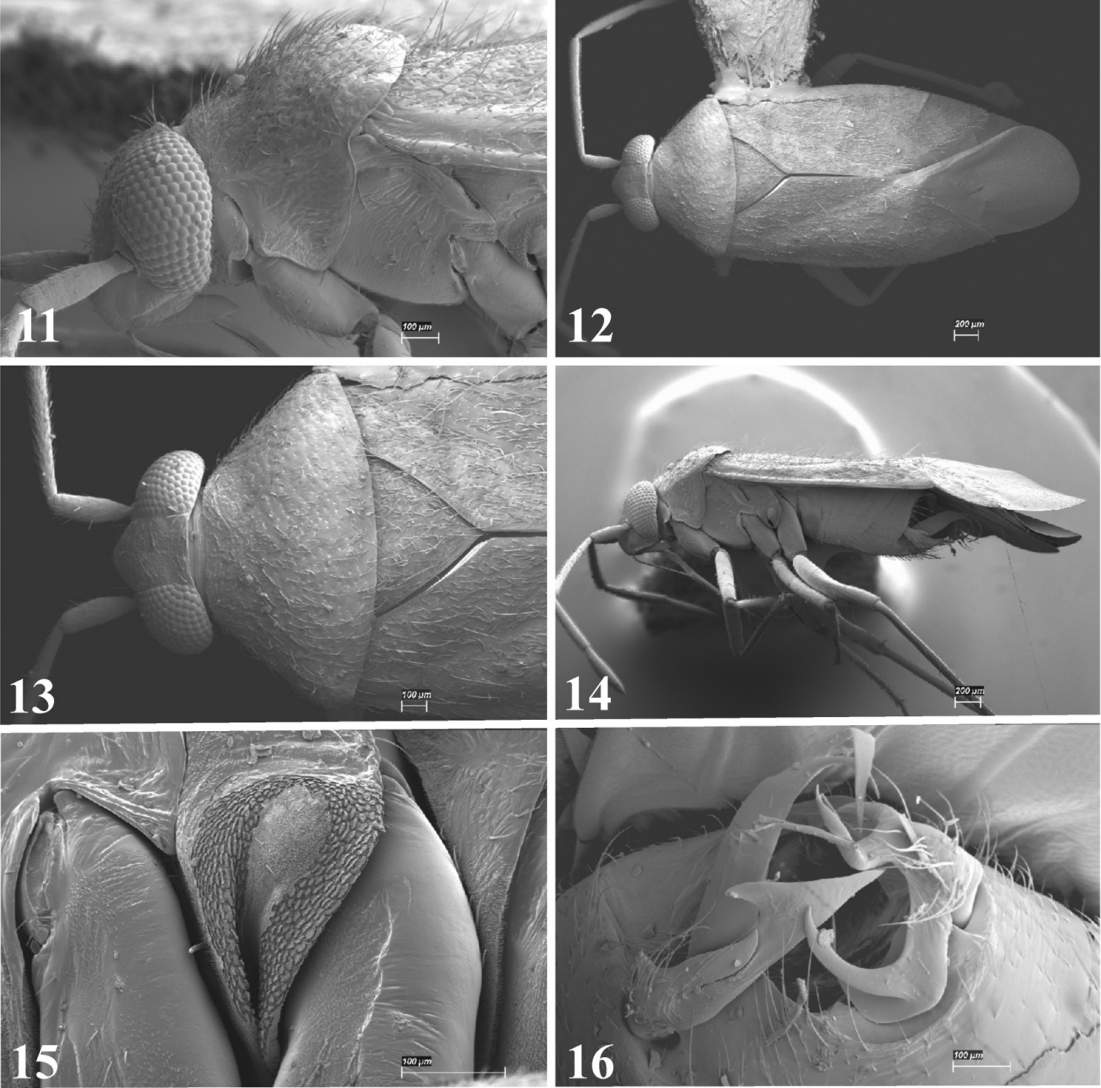
SEM photomicrographs of *Ceratocapsidea complicata*, male. **11** head and pronotum, lateral aspect **12** full dorsal aspect **13** head and pronotum, dorsal aspect **14** full lateral aspect **15** ostiolar evaporative area **16** genital capsule, caudal aspect.

**Figure 17.**
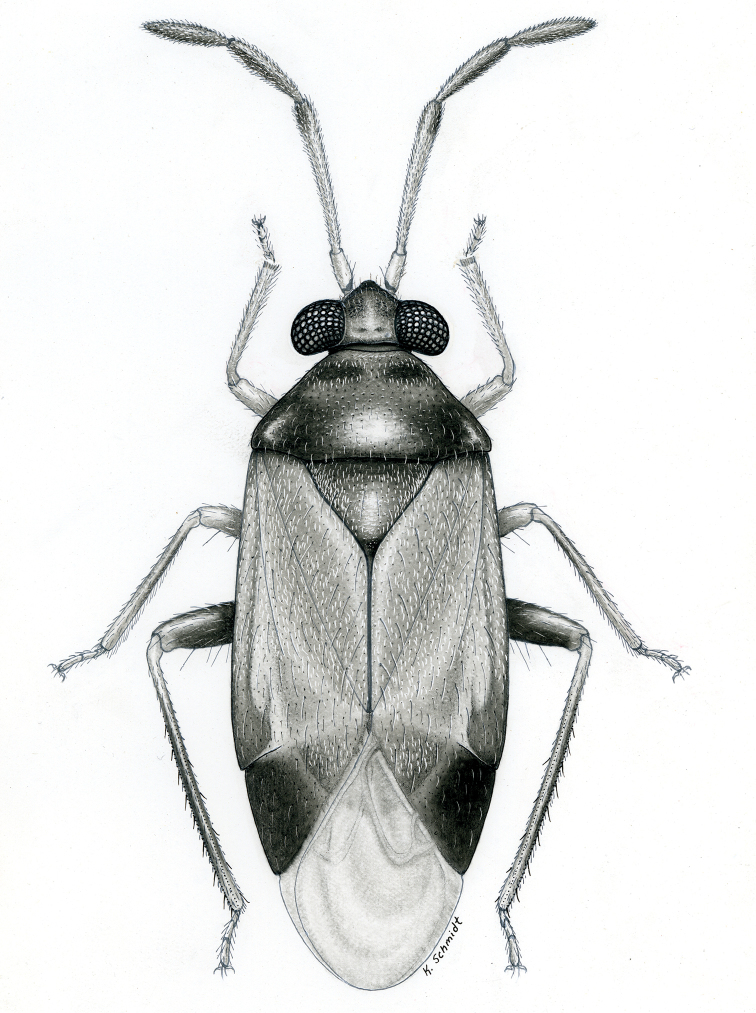
*Ceratocapsidea fusiformis*, adult male.

**Figures 18–23.**
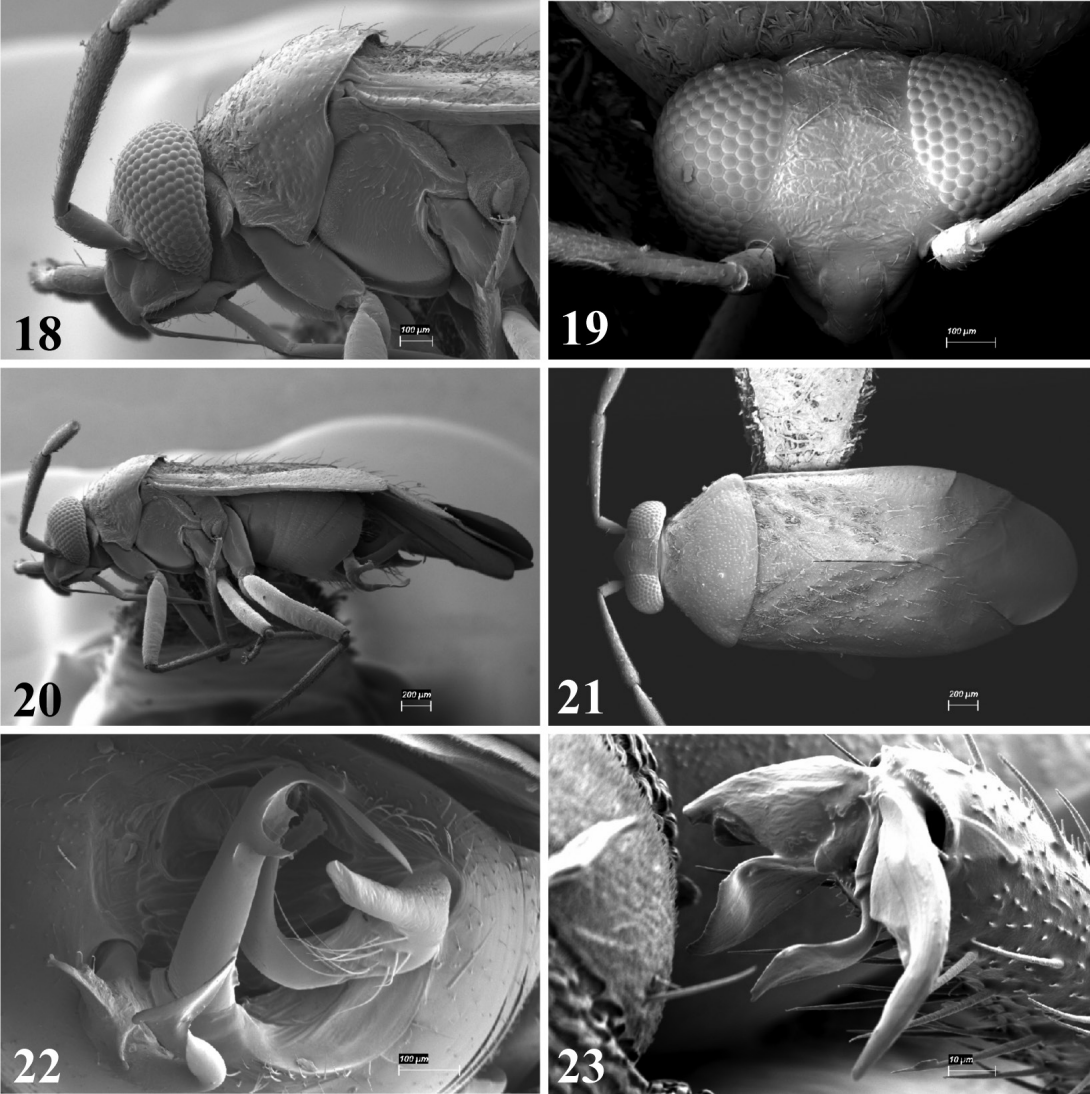
SEM photomicrographs of *Ceratocapsidea fusiformis*, male. **18** head and pronotum, lateral aspect **19** head, frontal aspect **20** full lateral aspect **21** full dorsal aspect **22** genital capsule, caudal aspect **23** pretarsus.

**Figures 24–35.**
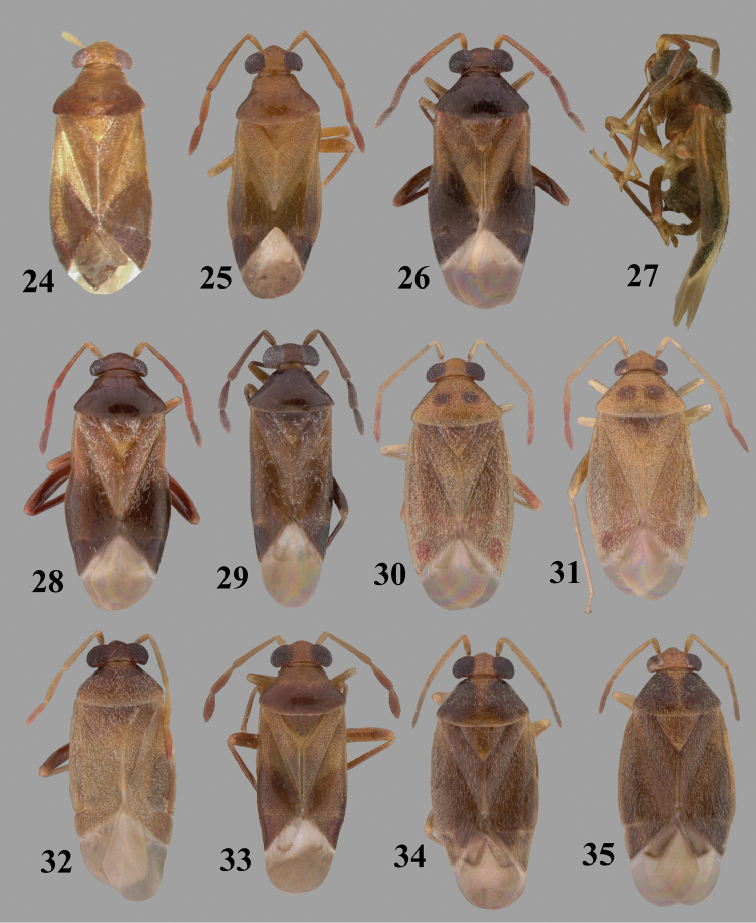
*Ceratocapsidea* spp., dorsal aspects, except as noted (see Appendix I for locality information). **24** *Ceratocapsidea alayoi*, ♂ **25** *Ceratocapsidea bahamaensis*, ♂ (paratype) **26** *Ceratocapsus balli*, ♂ **27** *Ceratocapsus balli*, ♂ (lateral aspect) **28** *Ceratocapsus balli*, ♀ **29** *Ceratocapsidea baranowskii*, ♂ (holotype) **30** *Ceratocapsidea complicata*, ♂ **31** *Ceratocapsidea complicata*, ♀ **32** *Ceratocapsidea consimilis*, ♂ **33** *Ceratocapsidea cubana*, ♂ (lectotype) **34** *Ceratocapsidea dominicanensis*, ♂ (holotype) **35** *Ceratocapsidea dominicanensis*, ♀ (paratype).

**Figures 36–47.**
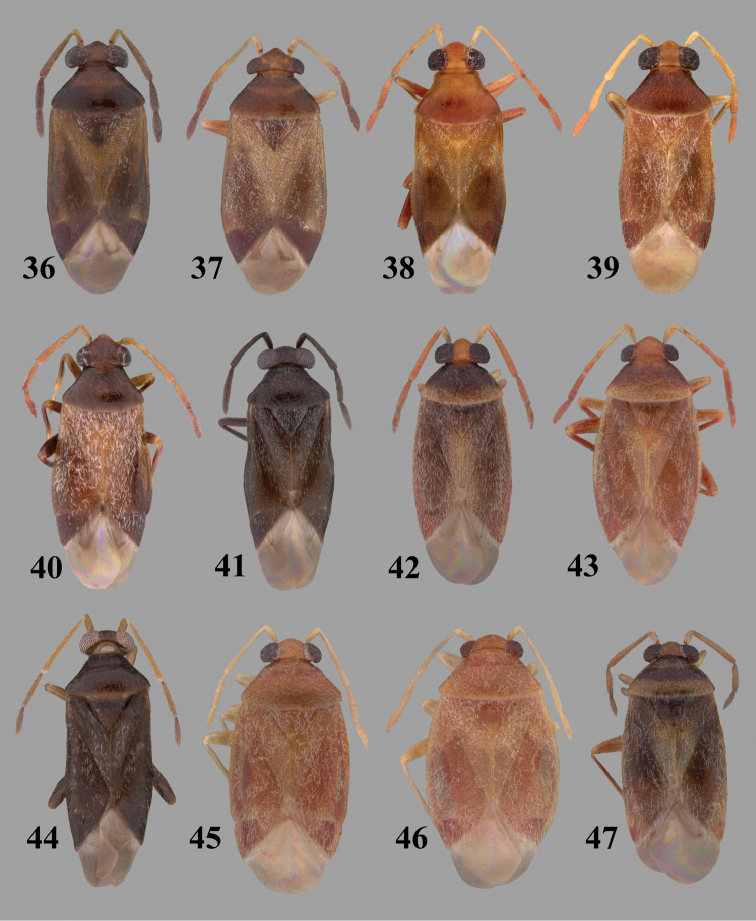
*Ceratocapsidea* spp., dorsal aspects (see Appendix I for locality information). **36** *Ceratocapsus fusiformis*, ♂ **37** *Ceratocapsus fusiformis*, ♀ **38** *Ceratocapsidea holguinensis*, ♂ **39** *Ceratocapsidea nigropicea*, ♂ **40** *Ceratocapsidea nigropicea* **41** *Ceratocapsidea rileyi*, ♂ (holotype) **42** *Ceratocapsidea rufistigma*, ♂ **43** *Ceratocapsidea rufistigma* **44** *Ceratocapsidea taeniola*, ♂ (holotype) **45** *Ceratocapsidea texensis*, ♂ (holotype) **46** *Ceratocapsidea texensis*, ♀ (paratype) **47** *Ceratocapsidea transversa*, ♂ (holotype).

**Figures 48–59.**
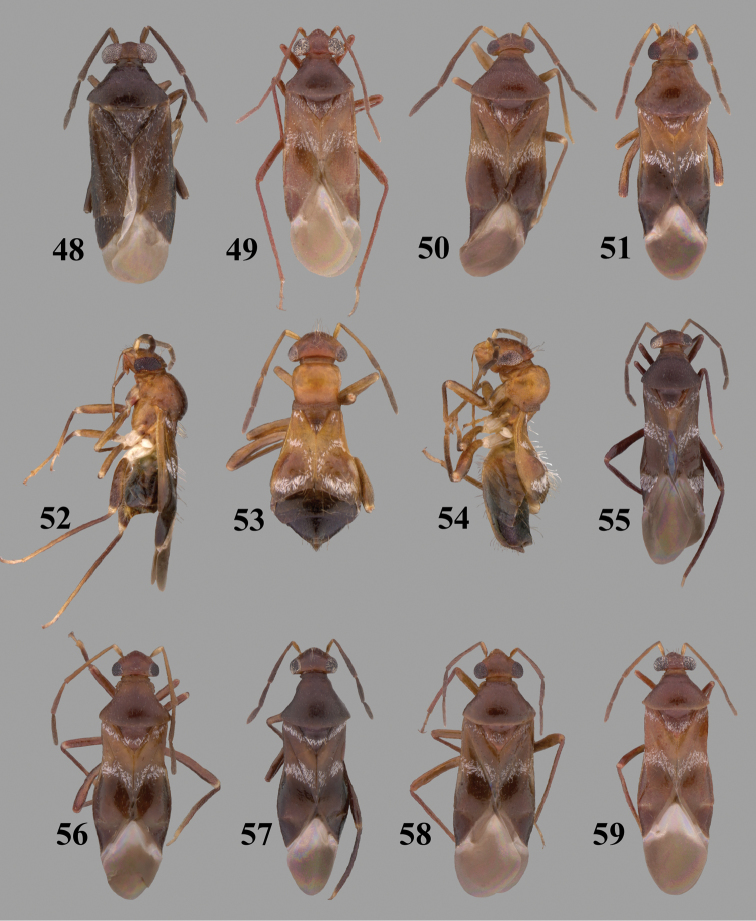
*Ceratocapsidea* and *Pilophoropsidea* spp., dorsal aspects, except as noted (see Appendix I for locality information). **48** *Ceratocapsidea variabilis*, ♂ (holotype) **49–59:** *Pilophoropsidea* spp. **49** *Pilophoropsidea barberi* (holotype), ♂ **50** *Pilophoropsidea brailovskyi*, ♂ (holotype) **51** *Pilophoropsidea camela*, ♂ **52** *Pilophoropsidea camela*, ♂ (lateral aspect) **53** *Pilophoropsidea camela* **54** *Pilophoropsidea camela*, ♀ (lateral aspect) **55** *Pilophoropsidea cuneata*, ♂ **56** *Pilophoropsidea dimidiata*, ♂ (holotype) **57** *Pilophoropsidea fascipennis*, ♂ (holotype) **58** *Pilophoropsidea fuscata*, ♂ (holotype) **59** *Pilophoropsidea keltoni*, ♂ (holotype).

**Figures 60–71.**
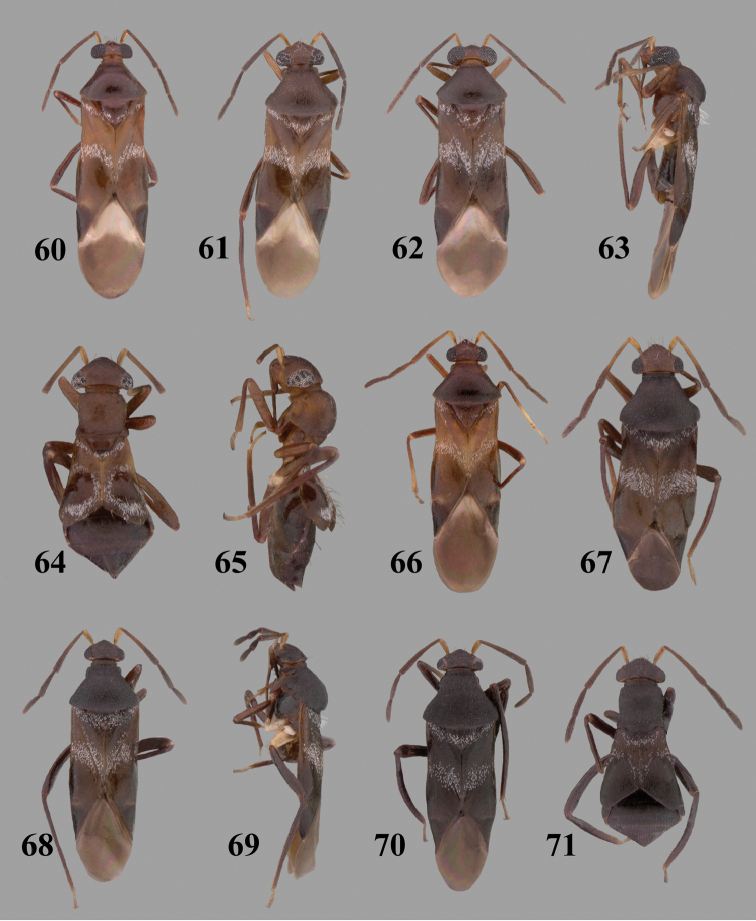
*Pilophoropsidea* spp., dorsal aspects, except as noted (see Appendix I for locality information). **60** *Pilophoropsidea maxima*, ♂ (holotype) **61** *Pilophoropsidea pueblaensis*, ♂ (holotype) **62** *Pilophoropsidea schaffneri*, ♂ (holotype) **63** *Pilophoropsidea schaffneri*, ♂ (lateral aspect) **64** *Pilophoropsidea schaffneri* (paratype) **65** *Pilophoropsidea schaffneri*, ♀(lateral aspect) **66** *Pilophoropsidea serrata*, ♂ (holotype) **67** *Pilophoropsidea touchetae*, ♂ (holotype) **68** *Pilophoropsidea truncata*, ♂ (holotype) **69** *Pilophoropsidea truncata*, ♂ (lateral aspect) **70** *Pilophoropsidea tuberculata*, ♂ (holotype) **71** *Pilophoropsidea tuberculata*, ♀ (paratype).

**Figures 72–83.**
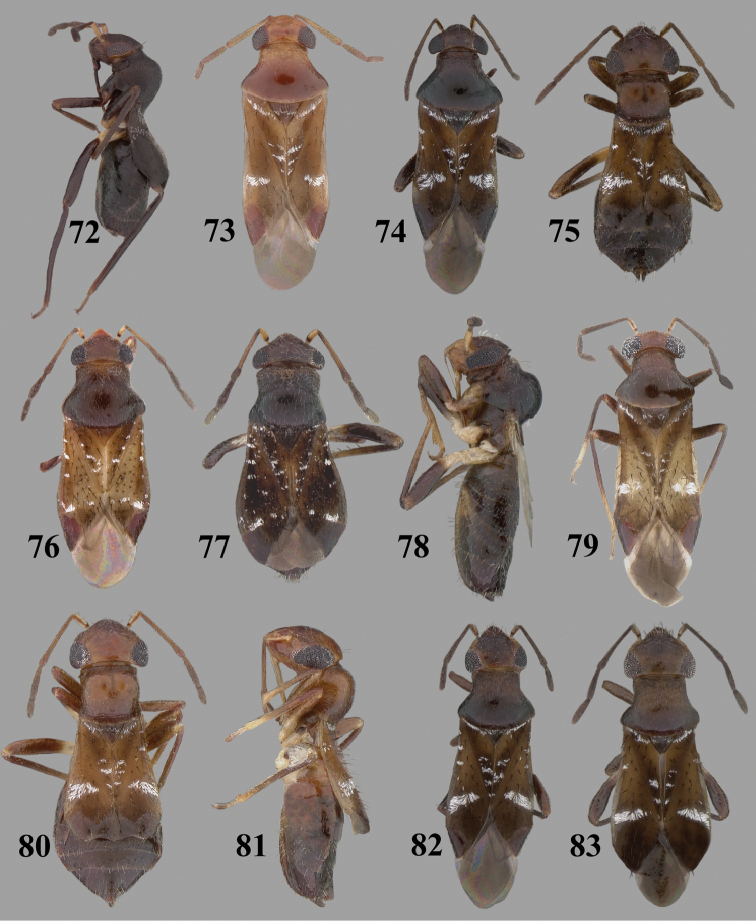
*Pilophoropsidea* and *Pilophoropsis* spp., dorsal aspects, except as noted (see Appendix I for locality information). **72** *Pilophoropsidea tuberculata*, ♀ (lateral aspect). *Pilophoropsis* spp. **73–83:** **73** *Pilophoropsis bejeanae*, ♂ (holotype) **74** *Pilophoropsis brachyptera*, ♂ **75** *Pilophoropsis brachyptera* **76** *Pilophoropsis cunealis*, ♂ (holotype) **77** *Pilophoropsis cunealis*, ♀ (paratype) **78** *Pilophoropsis cunealis*, ♀ (lateral aspect) **79** *Pilophoropsis nicholi*, ♂ **80** *Pilophoropsis nicholi*, ♀ **81** *Pilophoropsis nicholi* (lateral aspect) **82** *Pilophoropsis quercicola*, ♂ (holotype) **83** *Pilophoropsis quercicola*, ♀ (paratype).

**Figures 84–94.**
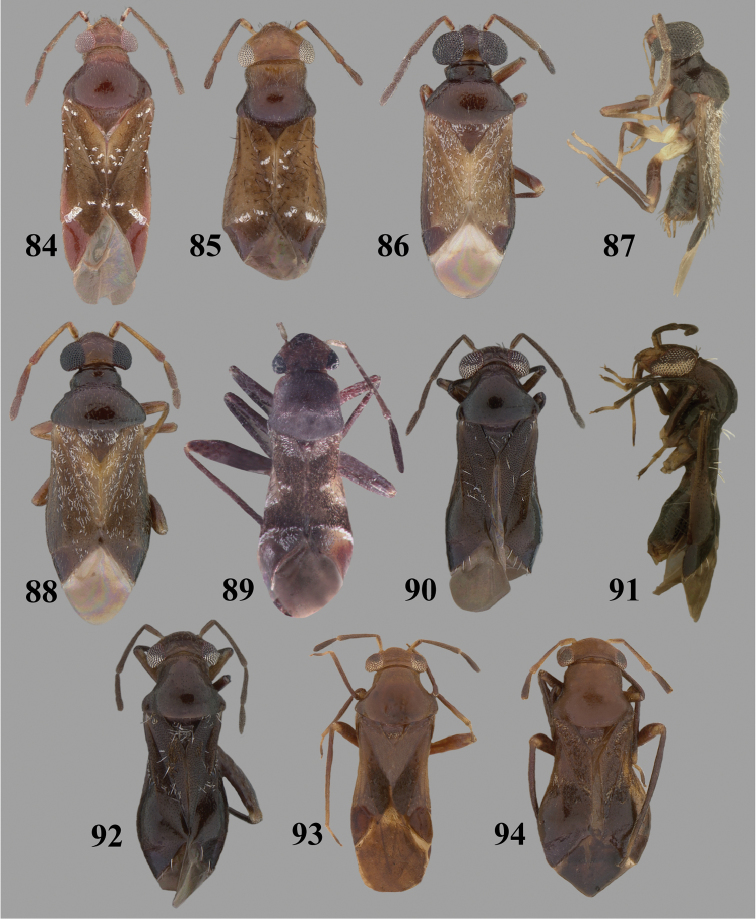
*Pilophoropsis*, *Pilophoropsita*, *Renodaeus*, and *Marinonicoris* spp., dorsal aspects, except as noted **84–85:** **84** *Pilophoropsis texana*, ♂ **85** *Pilophoropsis texana*, **86–88:** **86** *Pilophoropsita schaffneri*, ♂ **87** *Pilophoropsidea schaffneri*, ♂ (lateral aspect) **88** *Pilophoropsidea schaffneri*, ♀ **89–92:** *Renodaeus* spp. **89** *Renodaeus ficarius* ♀ **90** *Renodaeus mimeticus*, ♂ **91** *Renodaeus mimeticus*, ♂ (lateral aspect) **92** *Renodaeus mimeticus* **93–94:** **93** *Marinonicoris myrmecoides*, ♂ **94** *Marinonicoris myrmecoides*, ♀.

**Figures 95–106.**
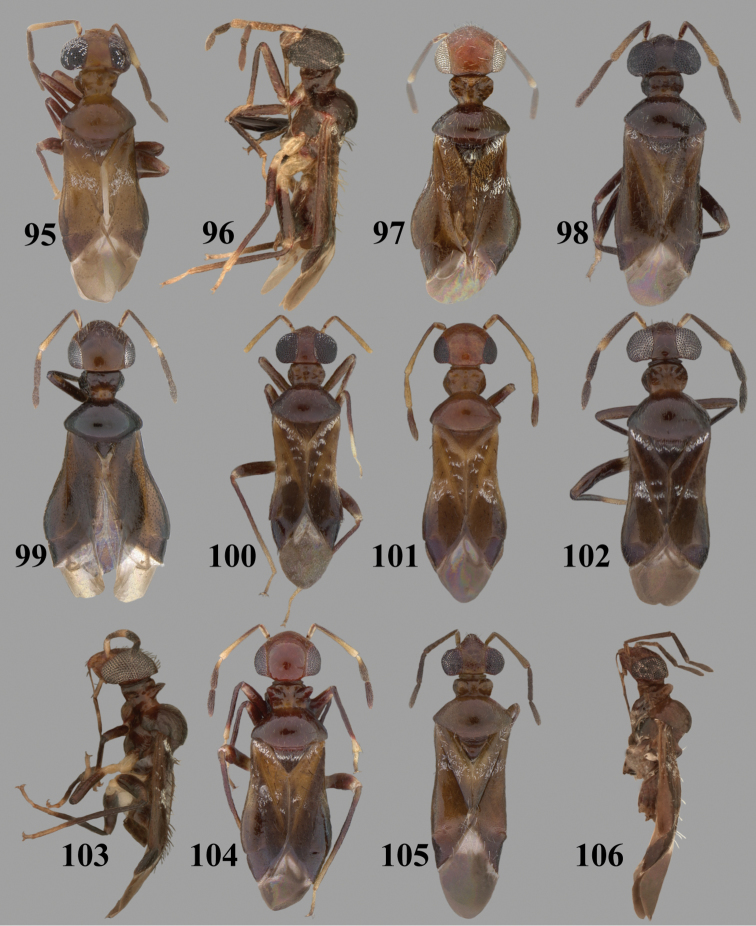
*Zanchisme* and *Zanchismeopsidea* spp. **95–104:** *Zanchisme* spp. **95** *Zanchisme dromidarus*, ♂ **96** *Zanchisme dromedarius*, ♂ (lateral aspect) **97** *Zanchisme dromedarius*, ♀ **98** *Zanchisme inermis*, ♂ **99** *Zanchisme inermis* **100** *Zanchisme illustris*, ♂ **101** *Zanchisme illustris*, ♀ **102** *Zanchisme mexicanus*, ♂ **103** *Zanchisme mexicanus*, ♂ (lateral aspect) **104** *Zanchisme mexicanus*, **105–106:** *Zanchismeopsidea* sp. **105** *Zanchismeopsidea diegoi*, ♂ **106** *Zanchismeopsidea diegoi*, ♂ (lateral aspect).

**Figure 107.**
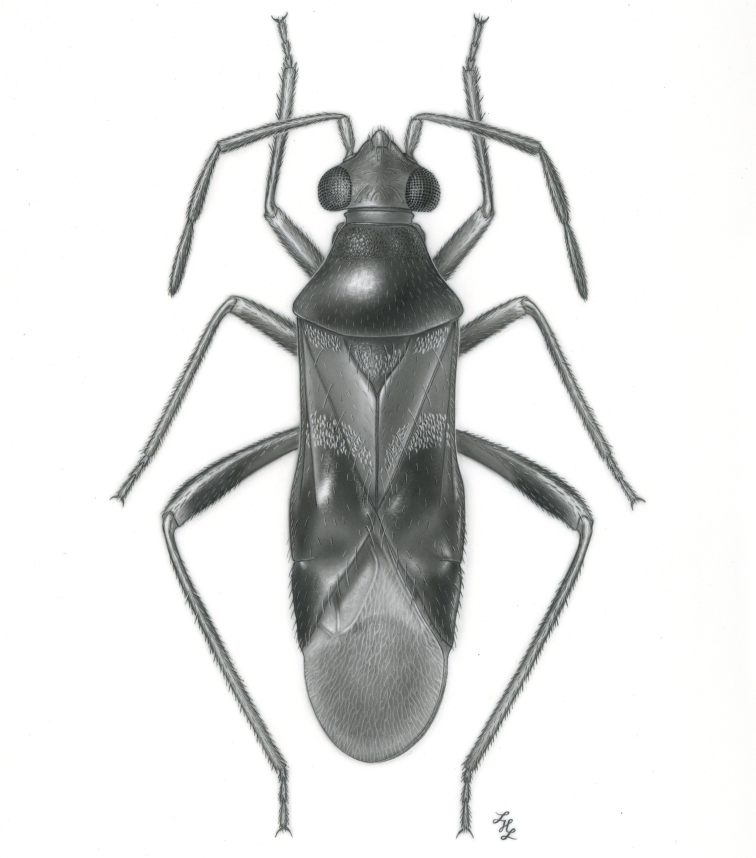
*Pilophoropsidea camela*, adult male.

**Figures 108–115.**
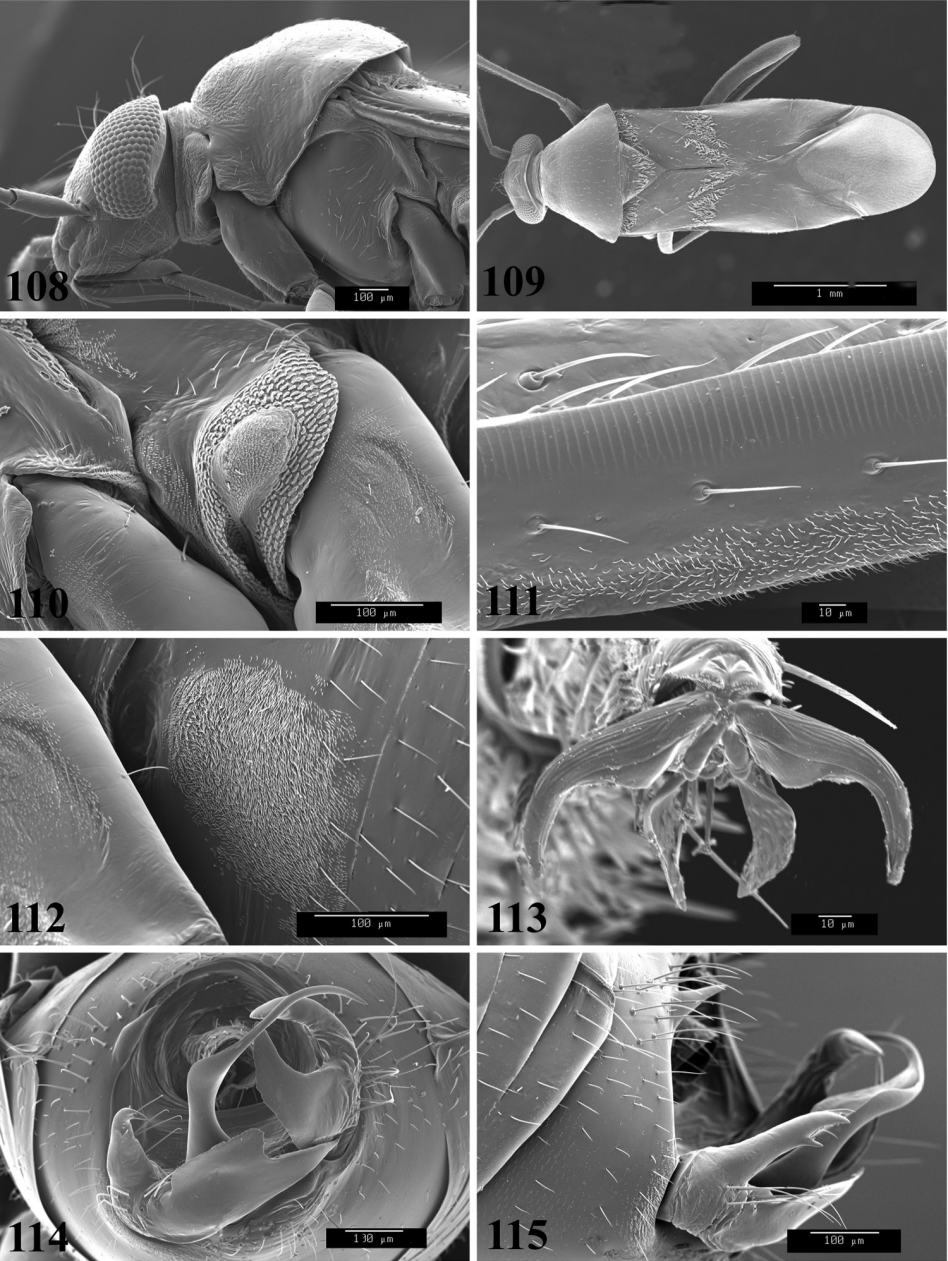
SEM photomicrographs of *Pilophoropsidea camela*, male. **108** head and pronotum, lateral aspect **109** head and pronotum, dorsal aspect **110** ostiolar evaporative area **111** stridulitrum on costal margin of hemelytron **112** glaucous patch (made of many tiny trichomes) at base of abdomen **113** pretarsus **114** genital capsule, caudal aspect **115** genital capsule, lateral aspect.

**Figures 116–119.**
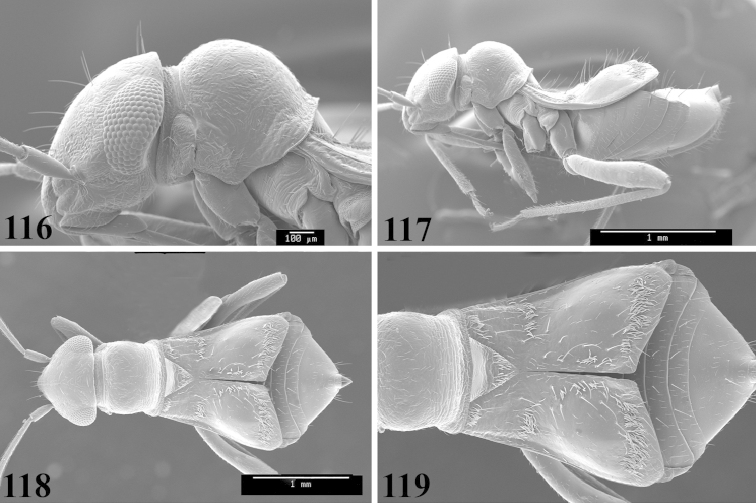
SEM photomicrographs of *Pilophoropsidea camela*, female. **116** head and pronotum, lateral aspect **117** full lateral aspect **118** full dorsal aspect **119** dorsal aspect of hemelytra and abdomen.

**Figure 120.**
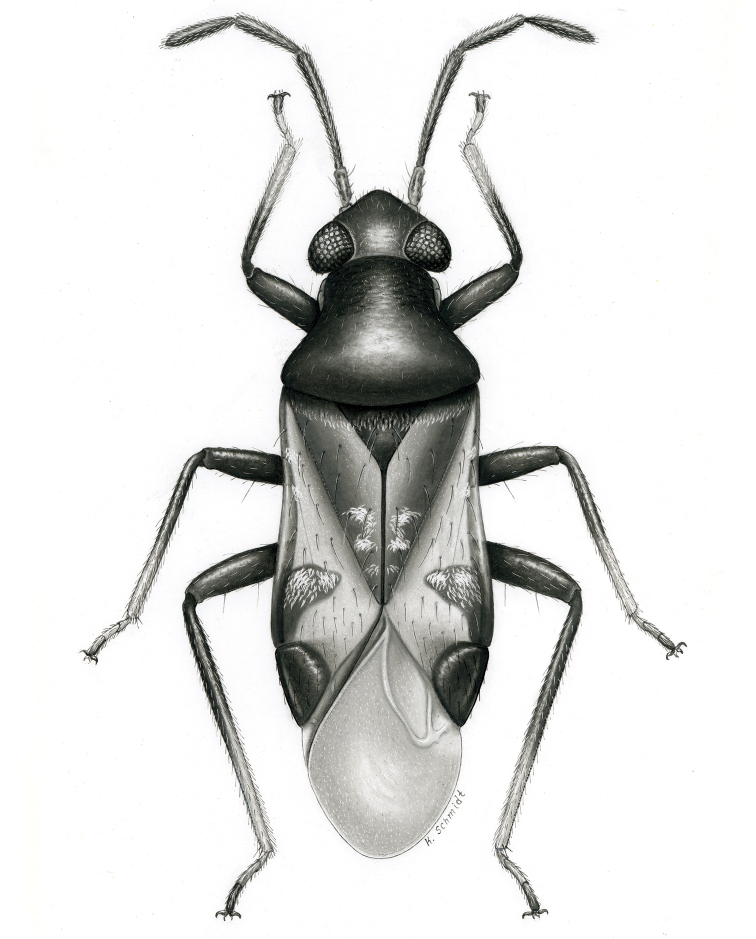
*Pilophoropsis brachyptera*, adult male.

**Figures 121–126.**
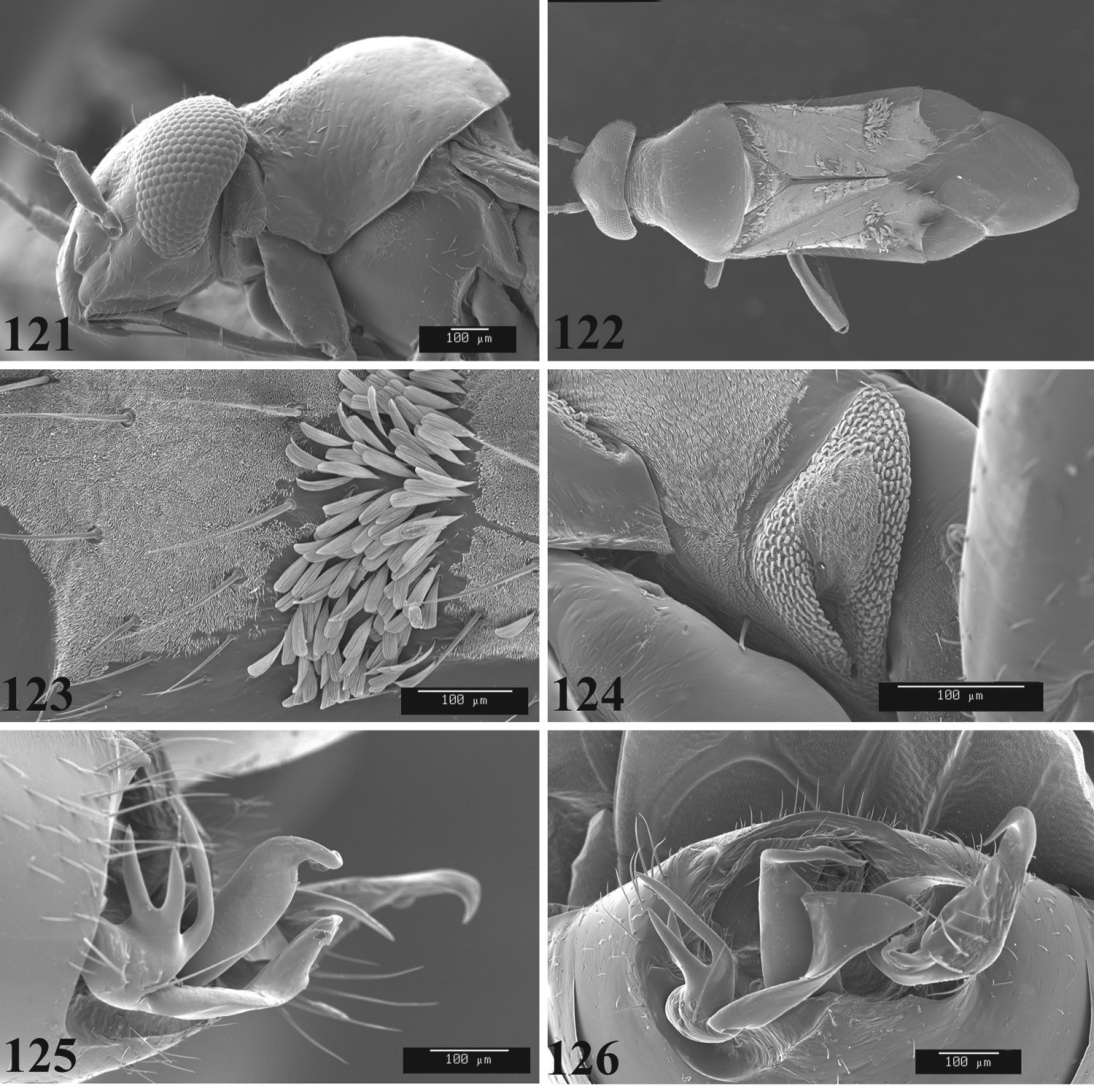
SEM photomicrographs of *Pilophoropsis brachyptera*, male. **121** head and pronotum, lateral aspect **122** full dorsal aspect **123** scale-like setae on hemelytron **124** ostiolar evaporative area **125** genital capsule, lateral aspect **126** genital capsule, caudal aspect.

**Figures 127–132.**
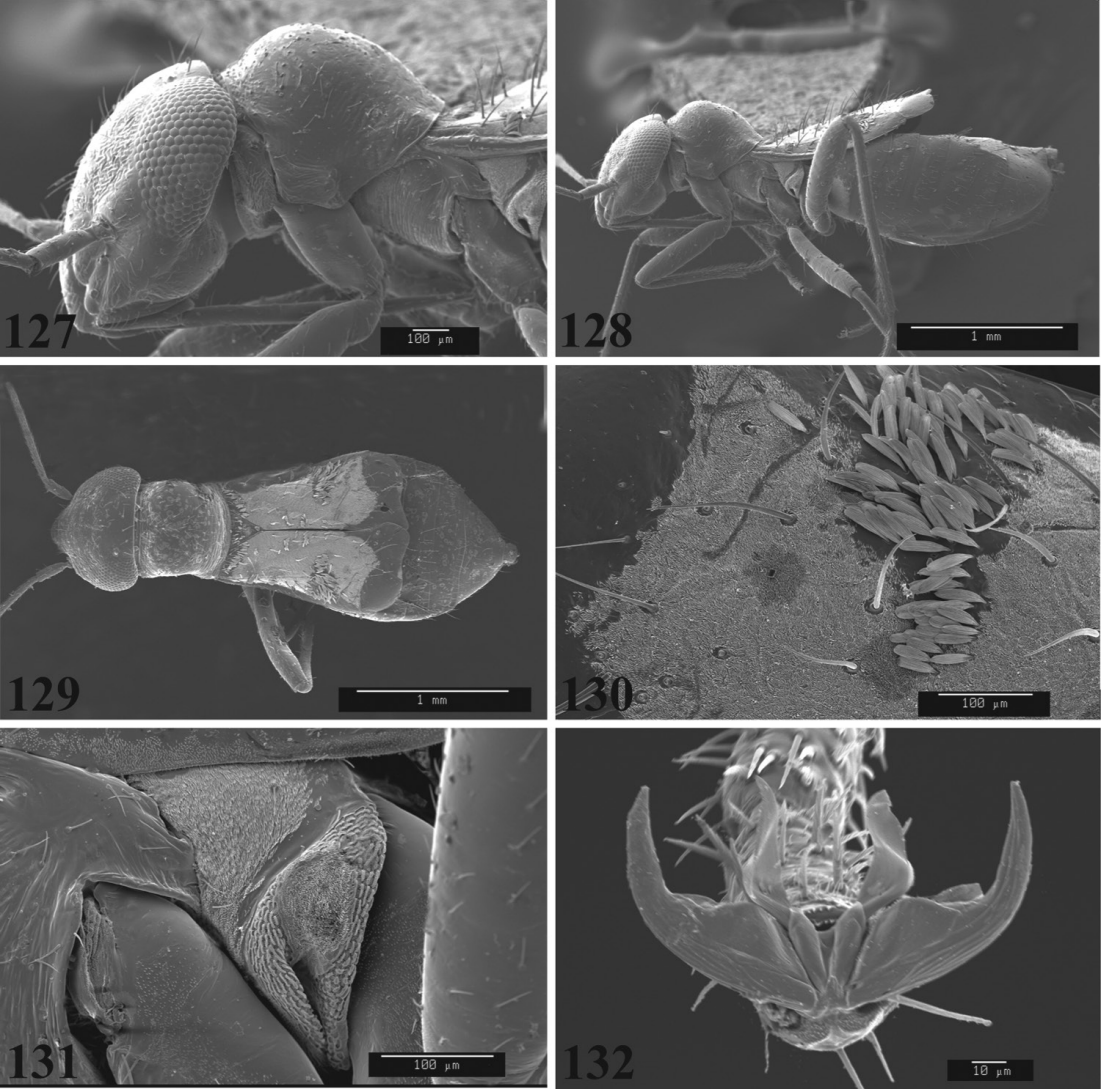
SEM photomicrographs of *Pilophoropsis brachyptera*, female. **127** head and pronotum, lateral aspect **128** full lateral aspect **129** full dorsal aspect **130** scale-like setae on hemelytron **131** ostiolar evaporative area **132** pretarsus.

**Figures 133–138.**
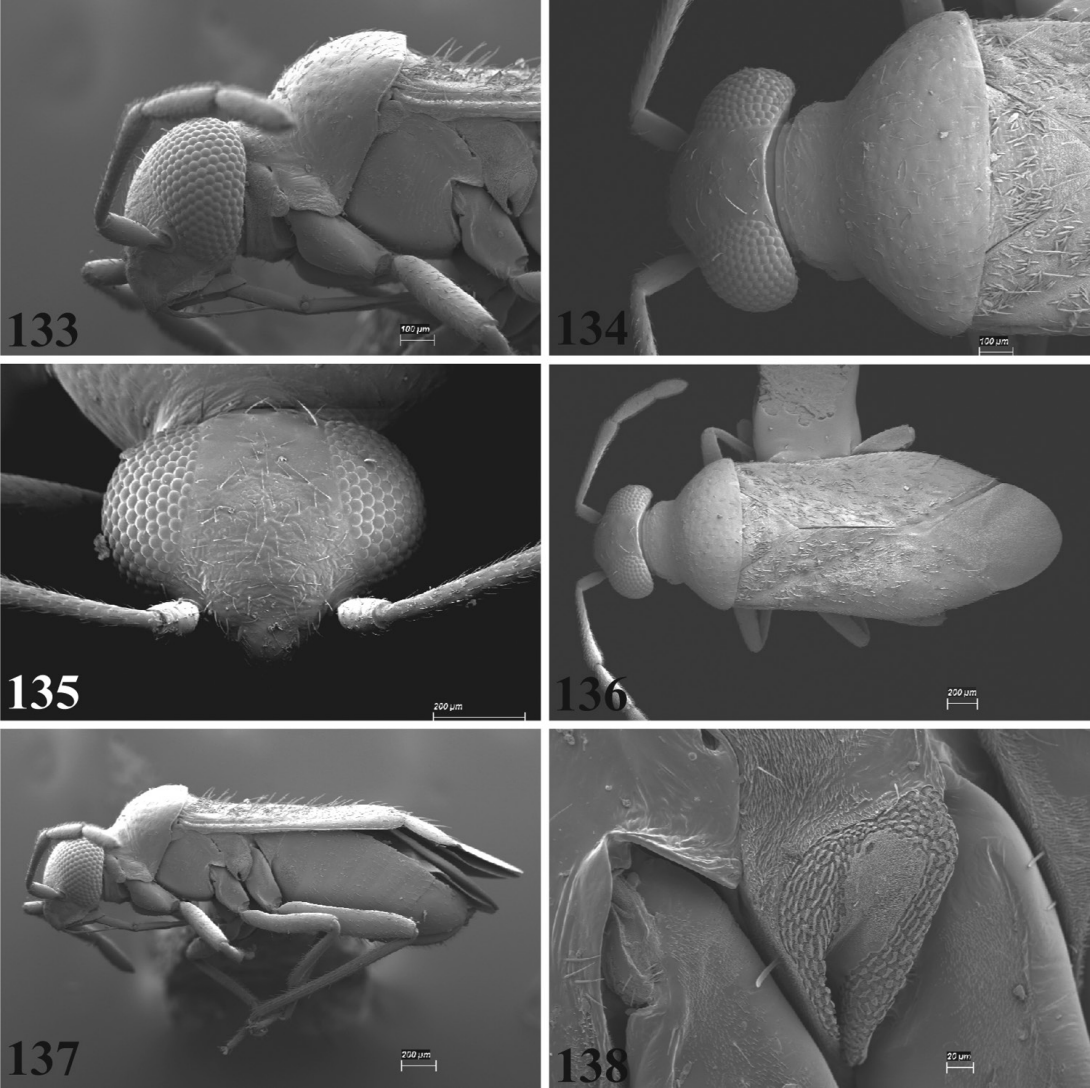
SEM photomicrographs of *Pilophoropsita schaffneri*, female. **133** head and pronotum, lateral aspect **134** head and pronotum, dorsal aspect **135** head, frontal aspect **136** full dorsal aspect **137** full lateral aspect **138** ostiolar evaporative area.

**Figure 139.**
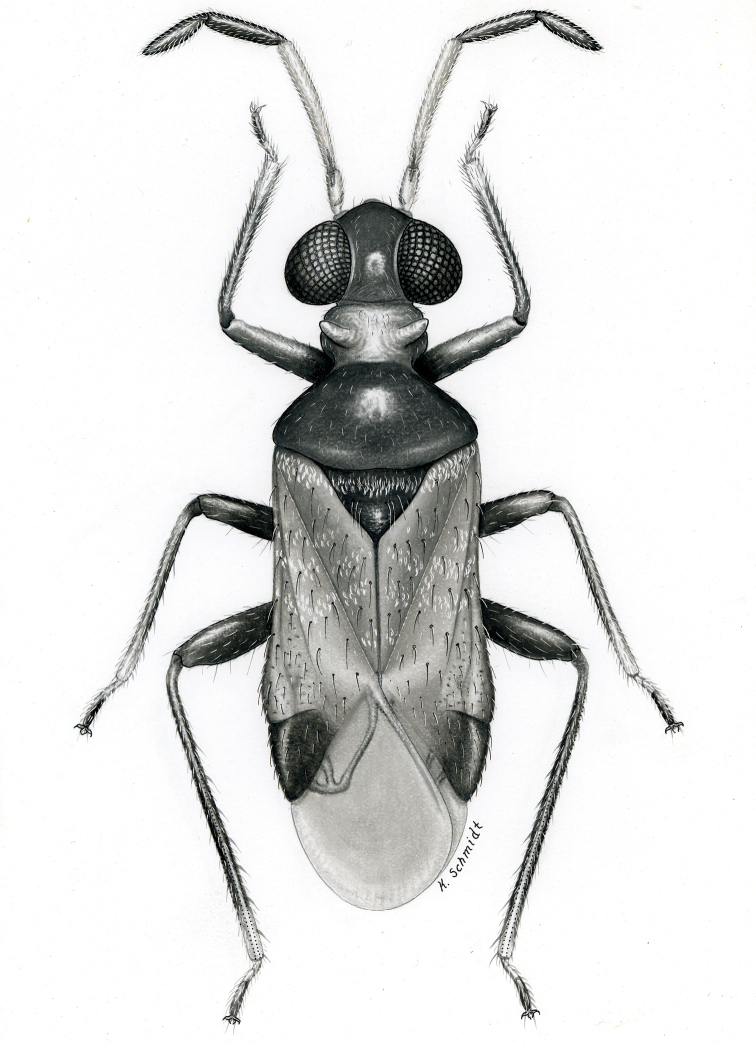
*Zanchisme mexicanus*, adult male.

**Figures 140–147.**
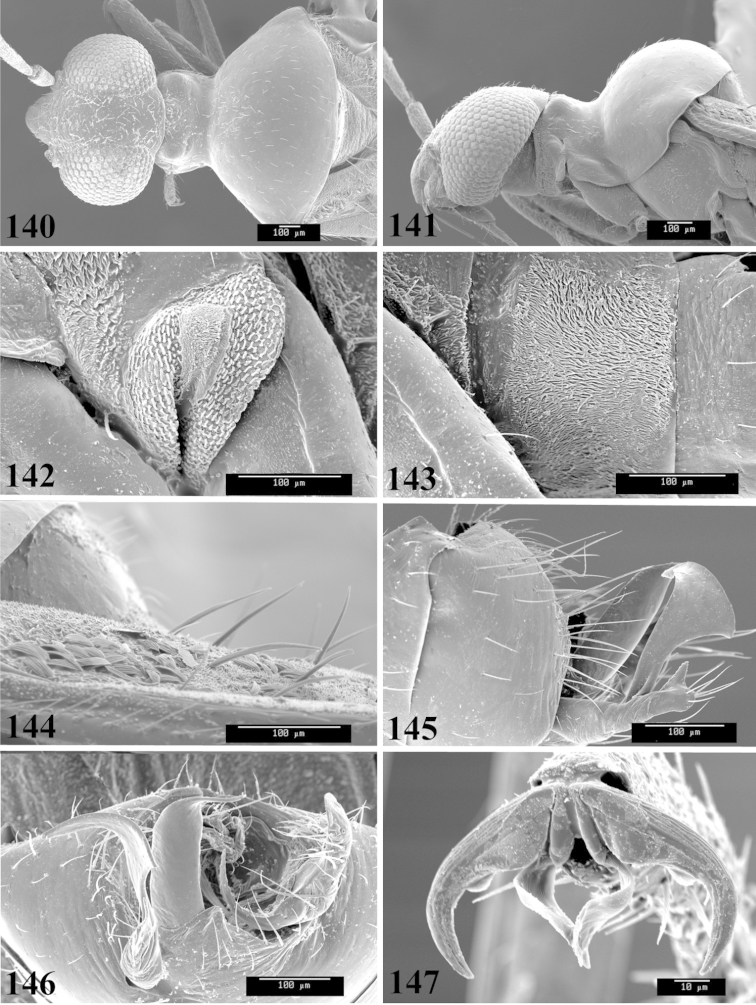
SEM photomicrographs of *Zanchisme illustris*, male. **140** head and pronotum, dorsal aspect **141** head and pronotum, lateral aspect **142** ostiolar evaporative area **143** glaucous patch (made of many tiny trichomes) at base of abdomen **144** bristle- and scale-like setae on hemelytron, lateral aspect **145** genital capsule, lateral aspect **146** genital capsule, caudal aspect **147** pretarsus.

**Figures 148–155.**
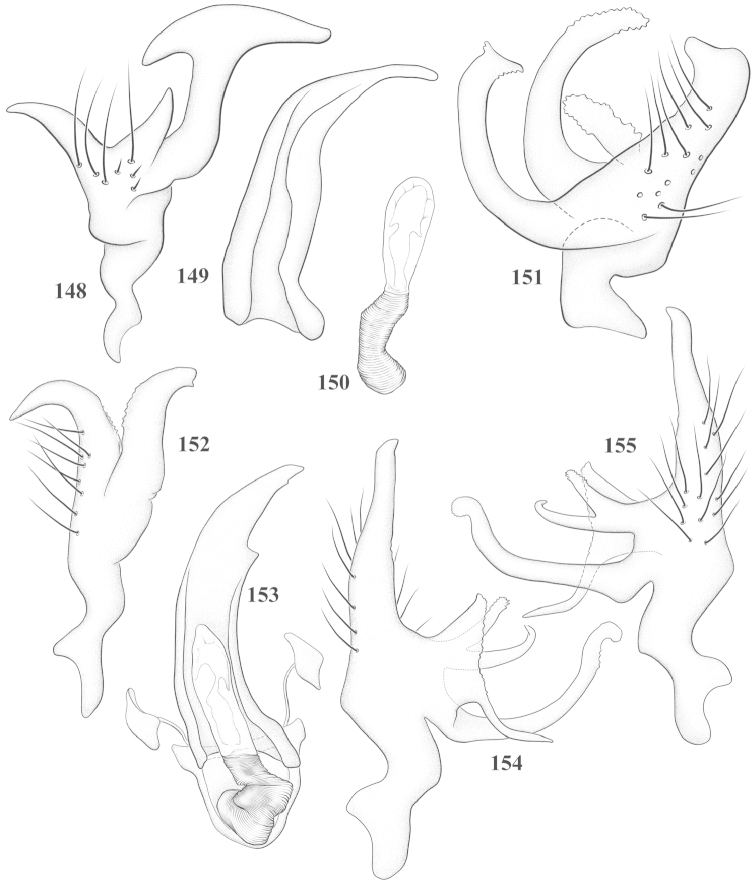
Male genitalia of *Ceratocapsidea* spp. **148–151** *Ceratocapsidea alayoi* **148** left paramere **149** phallotheca **150** endosoma, in part **151** right paramere, caudal aspect **152–155:** *Ceratocapsidea bahamaensis* **152** left paramere **153** endosoma and phallotheca **154** right paramere, anterior aspect **155** right paramere, caudal aspect.

**Figures 156–162.**
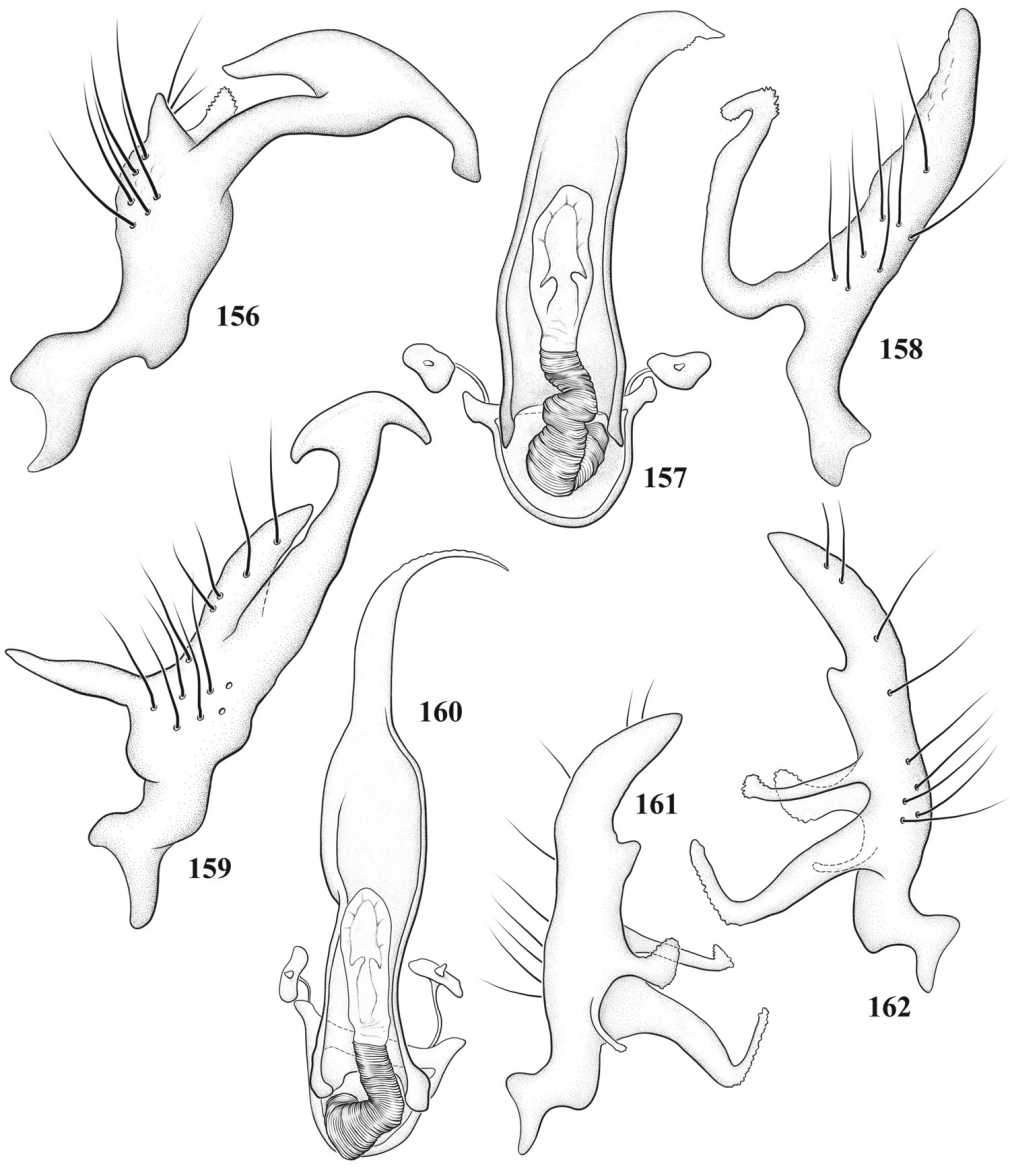
Male genitalia of *Ceratocapsidea* spp. **156–158:** *Ceratocapsus balli* **156** left paramere **157** phallotheca and endosoma **158** right paramere **159–162:** *Ceratocapsidea baranowskii* **159** left paramere **160** endosoma and phallotheca **161** right paramere, anterior aspect **162** right paramere, caudal aspect.

**Figures 163–170.**
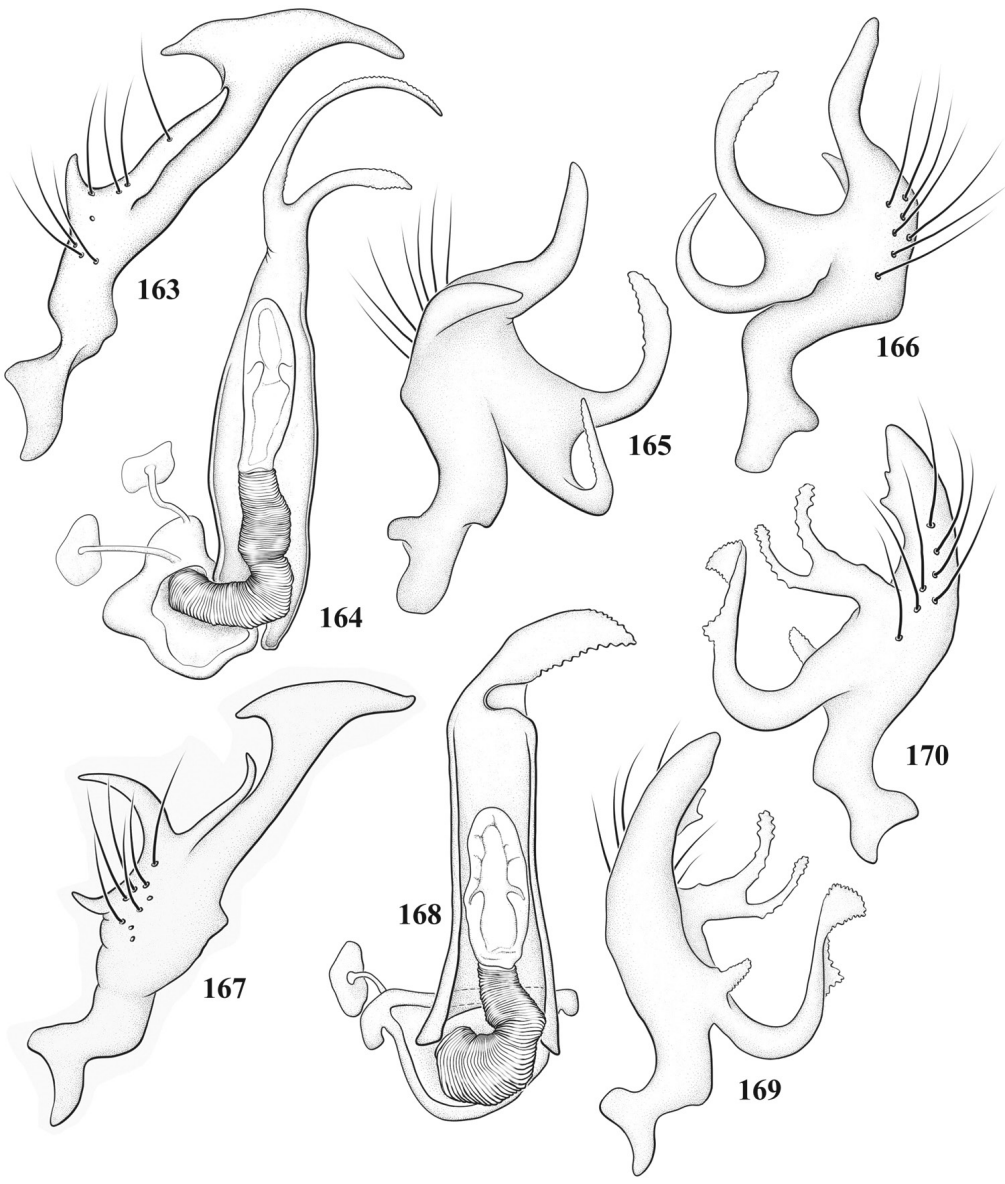
Male genitalia of *Ceratocapsidea* spp. **163–166:** *Ceratocapsidea complicata* **163** left paramere **164** phallotheca and endosoma **165** right paramere, anterior aspect **166** right paramere, caudal aspect **167–170:** *Ceratocapsidea consimilis* **167** left paramere **168** endosoma and phallotheca **169** right paramere, anterior aspect **170** right paramere, caudal aspect.

**Figures 171–177.**
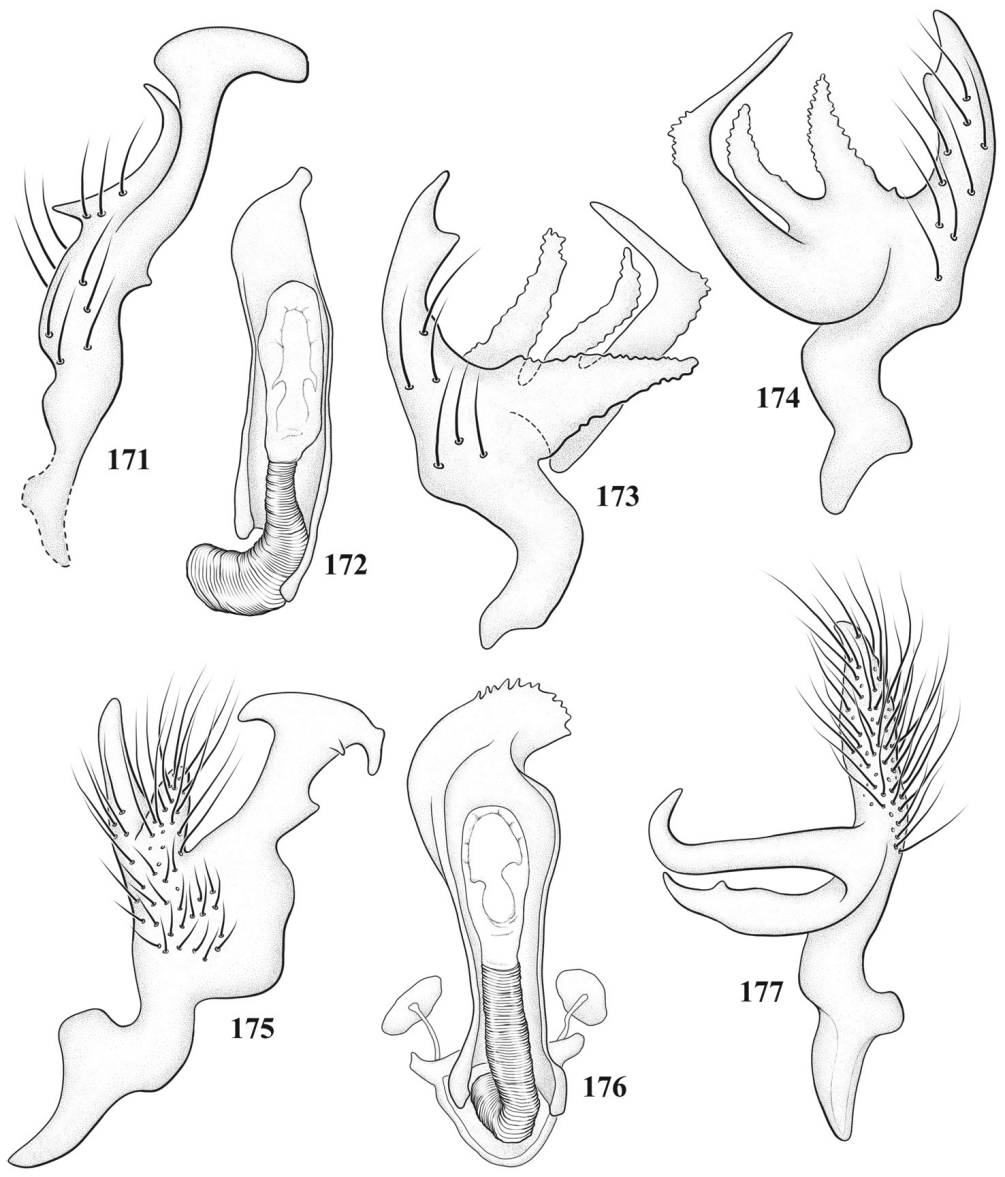
Male genitalia of *Ceratocapsidea* spp. **171–174:** *Ceratocapsidea cubana* **171** left paramere **172** phallotheca and endosoma **173** right paramere, anterior aspect **174** right paramere, caudal aspect **175–177:** *Ceratocapsidea dominicanensis* **175** left paramere **176** endosoma and phallotheca **177** right paramere, caudal aspect.

**Figures 178–185.**
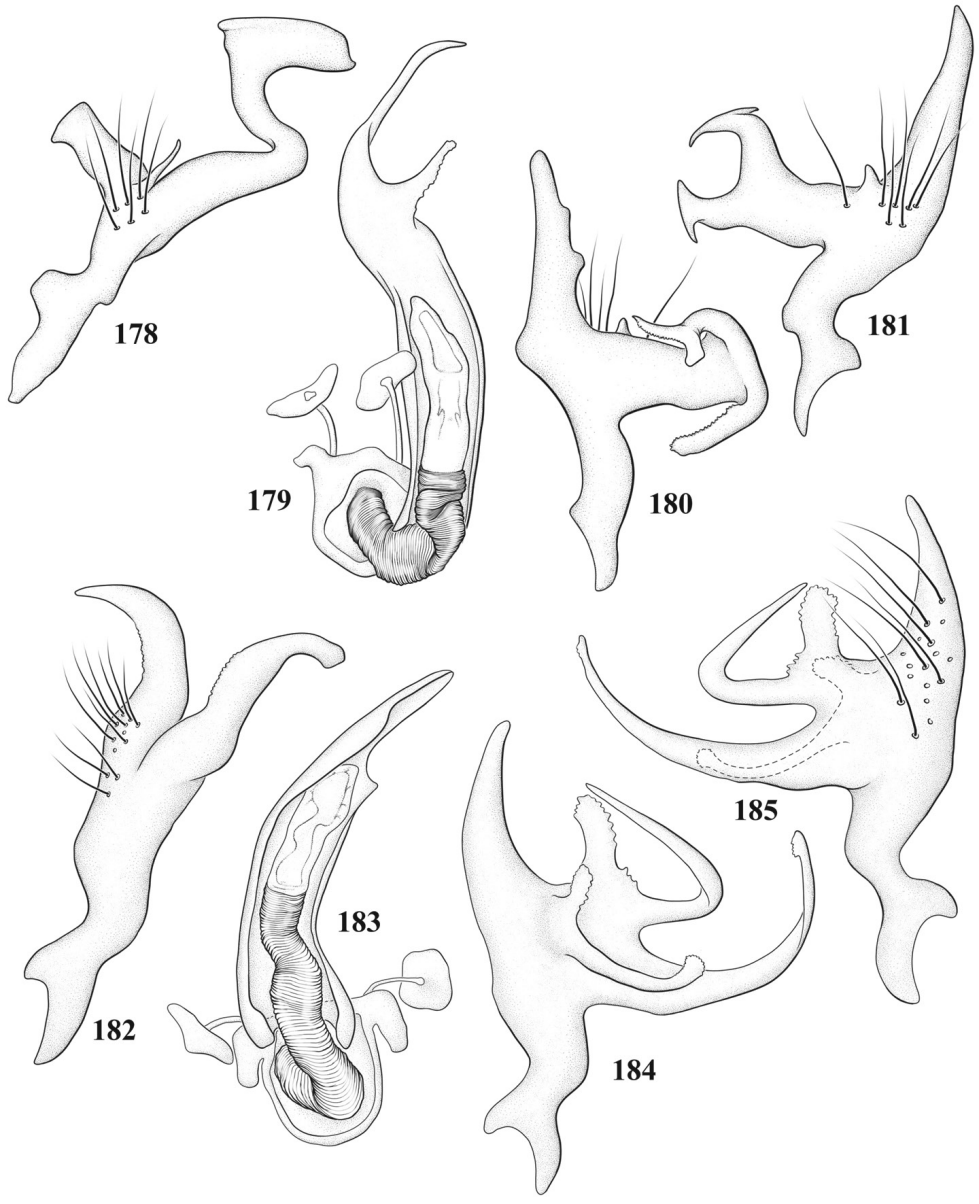
Male genitalia of *Ceratocapsidea* spp. **178–181:** *Ceratocapsus fusiformis* **178** left paramere **179** phallotheca and endosoma **180** right paramere, anterior aspect **181** right paramere, caudal aspect. **182–185:** *Ceratocapsidea holguinensis* **182** left paramere **183** endosoma and phallotheca **184** right paramere, anterior aspect **185** right paramere, caudal aspect.

**Figures 186–191.**
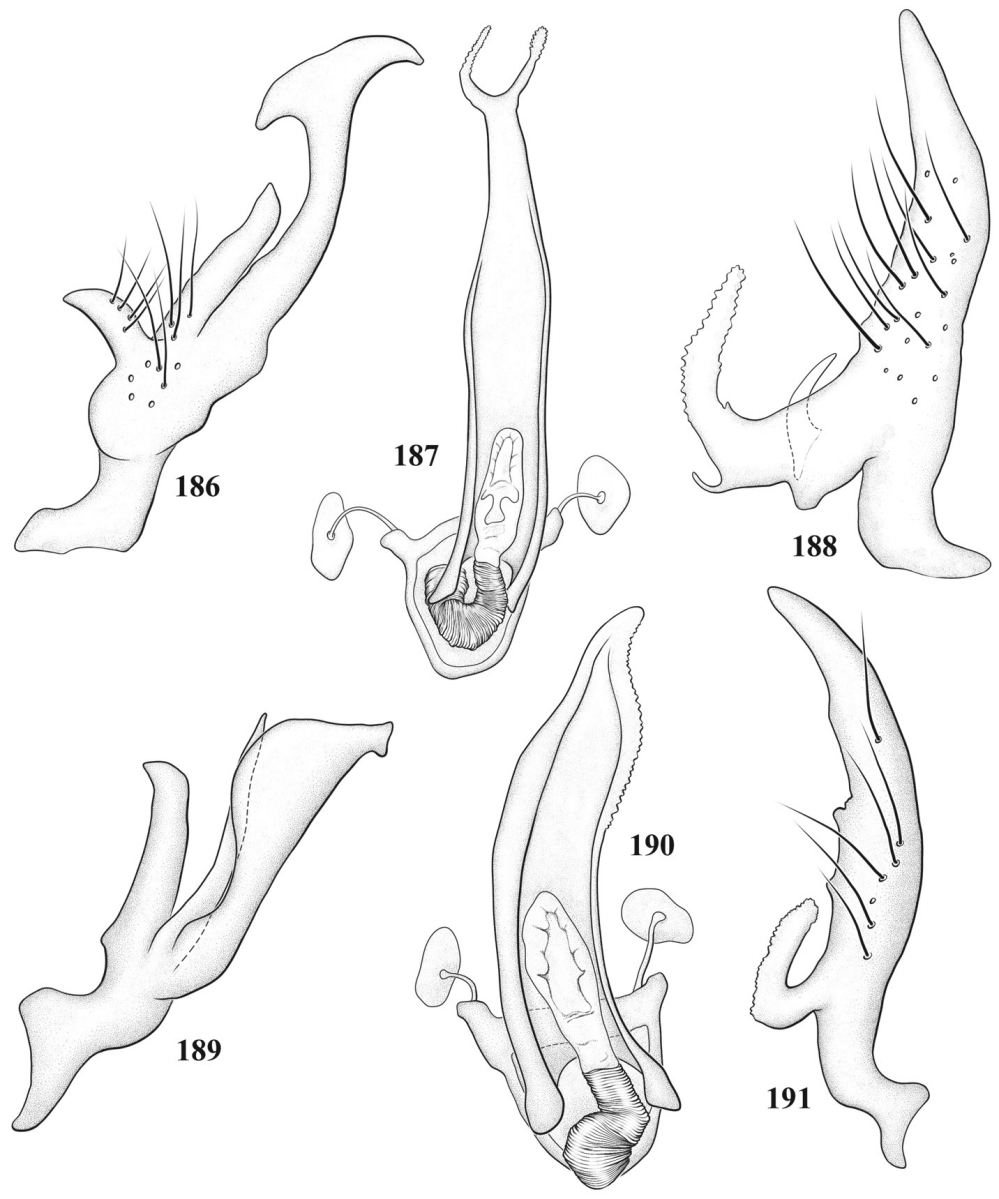
Male genitalia of *Ceratocapsidea* spp. **186–188:** *Ceratocapsidea nigropicea* **186** left paramere **187** phallotheca and endosoma **188** right paramere, caudal aspect **189–191:** *Ceratocapsidea rileyi* **189** left paramere **190** endosoma and phallotheca **191** right, caudal aspect.

**Figures 192–199.**
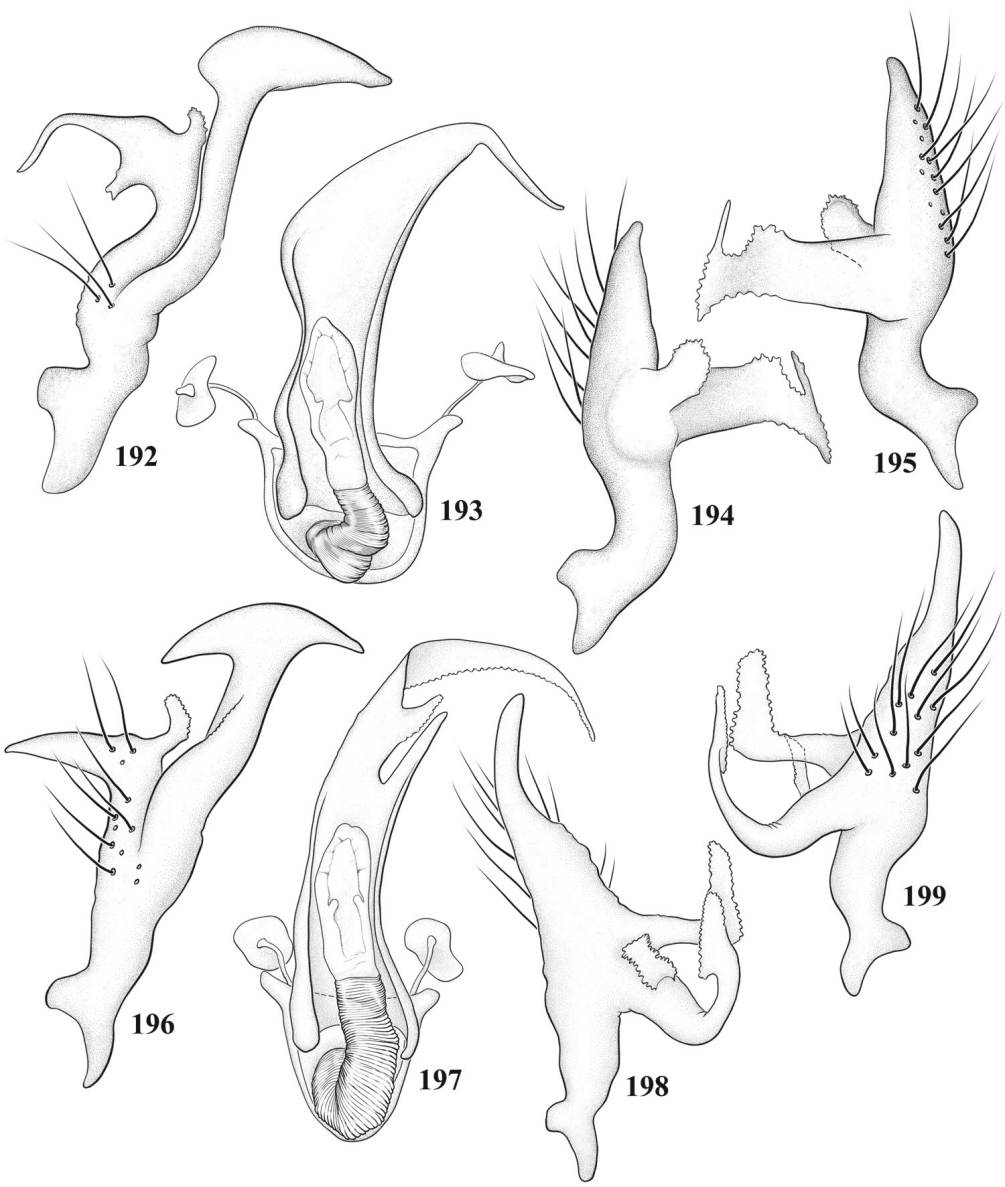
Male genitalia of *Ceratocapsidea* spp. **192–195:** *Ceratocapsidea rufistigma* **192** left paramere **193** phallotheca and endosoma **194** right paramere, anterior aspect **195** right paramere, caudal aspect **196–199:** *Ceratocapsidea taeniola* **196** left paramere **197** endosoma and phallotheca **198** right paramere, anterior aspect **199** right paramere, caudal aspect.

**Figures 200–207.**
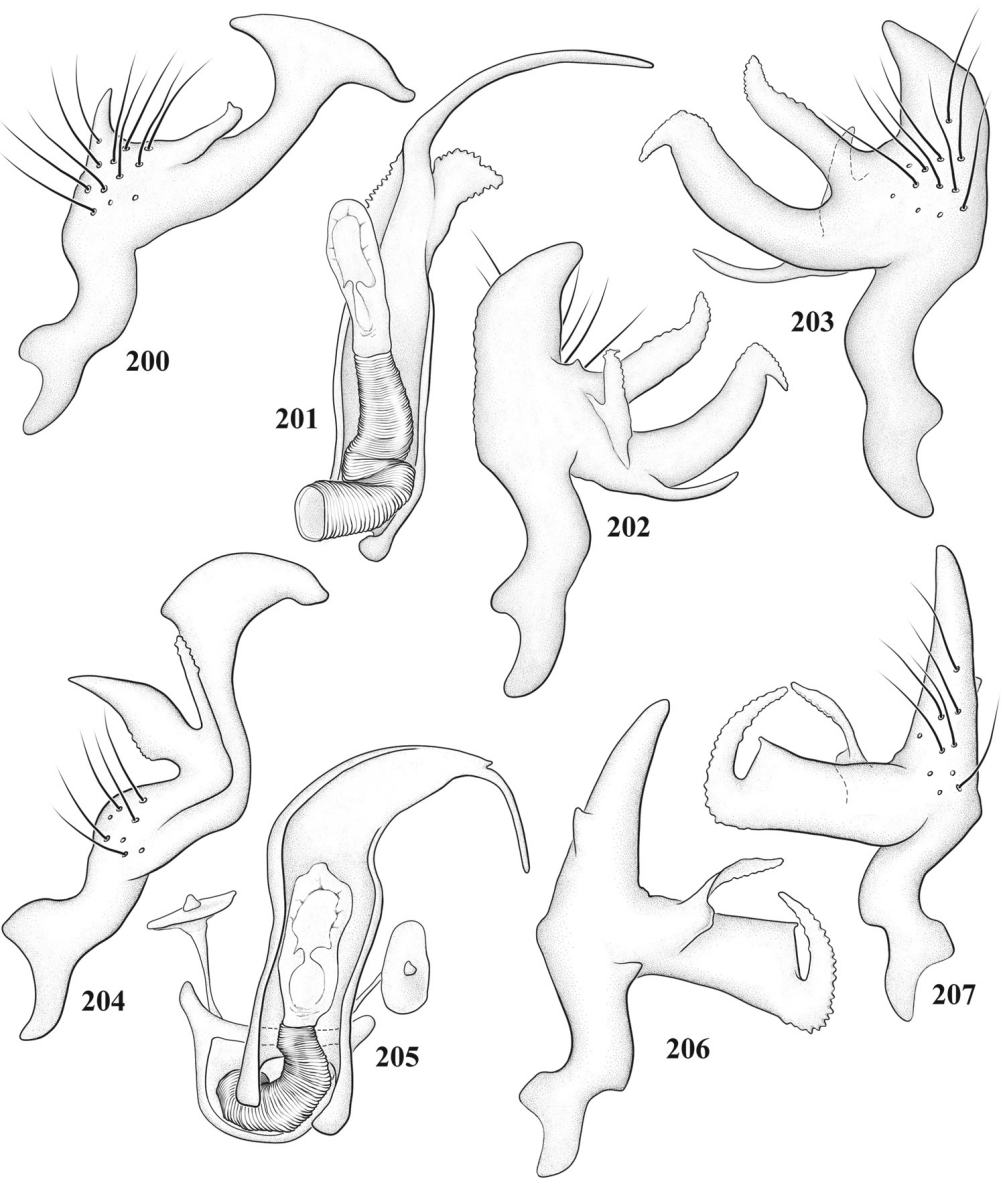
Male genitalia of *Ceratocapsidea* spp. **200–203:** *Ceratocapsidea texensis* **200** left paramere **201** phallotheca and endosoma **202** right paramere, anterior aspect **203** right paramere, caudal aspect **204–207:** *Ceratocapsidea transversa* **204** left paramere **205** endosoma and phallotheca **206** right paramere, anterior aspect **207** right paramere, caudal aspect.

**Figures 208–213.**
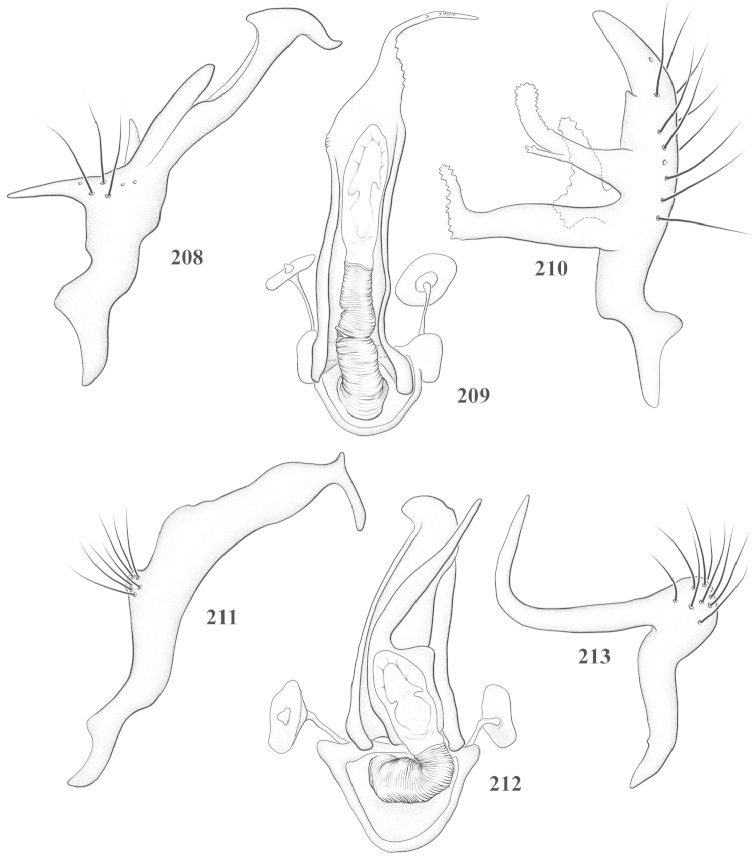
Male genitalia of *Ceratocapsidea* and *Pilophoropsidea* spp. **208–210:** *Ceratocapsidea variabilis* **208** left paramere **209** phallotheca and endosoma **210** right paramere, caudal aspect **211–213:** *Pilophoropsidea barberi* **211** left paramere **212** endosoma and phallotheca **213** right paramere, caudal aspect.

**Figures 214–219.**
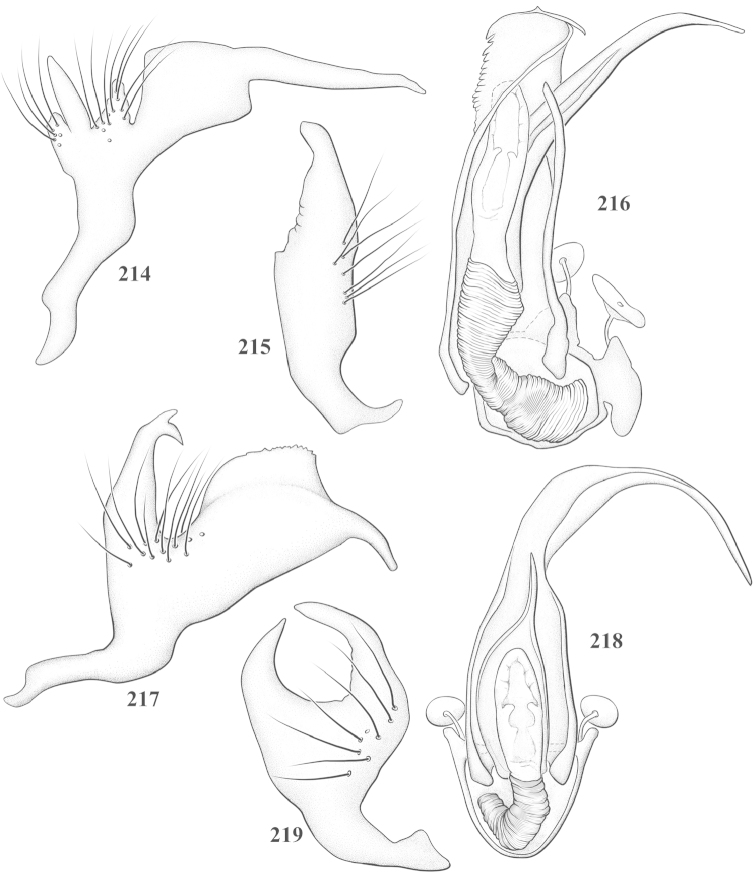
Male genitalia of *Pilophoropsidea* spp. **214–216:** *Pilophoropsidea brailovskyi* **214** left paramere **215** phallotheca and endosoma **216** right paramere, caudal aspect **217–219:** *Pilophoropsidea camela* **217** left paramere **218** endosoma and phallotheca **219** right paramere, caudal aspect.

**Figures 220–226.**
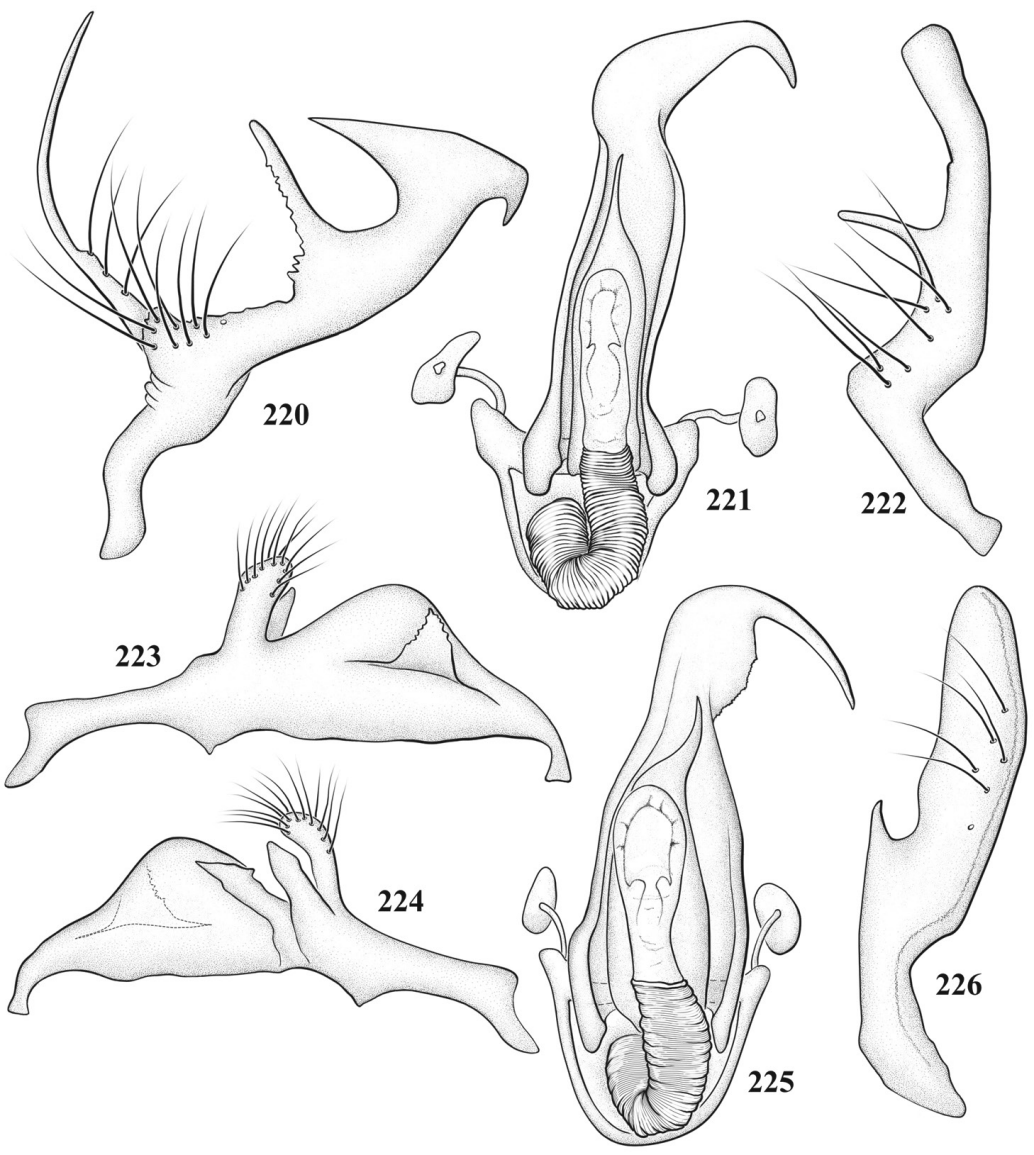
Male genitalia of *Pilophoropsidea* spp. **220–222:** *Pilophoropsidea cuneata* **220** left paramere **221** phallotheca and endosoma **222** right paramere, caudal aspect. **223–226:** *Pilophoropsidea dimidiata* **223** left paramere, caudal aspect **224** left paramere, anterior aspect **225** endosoma and phallotheca **226** right paramere, caudal aspect.

**Figures 227–233.**
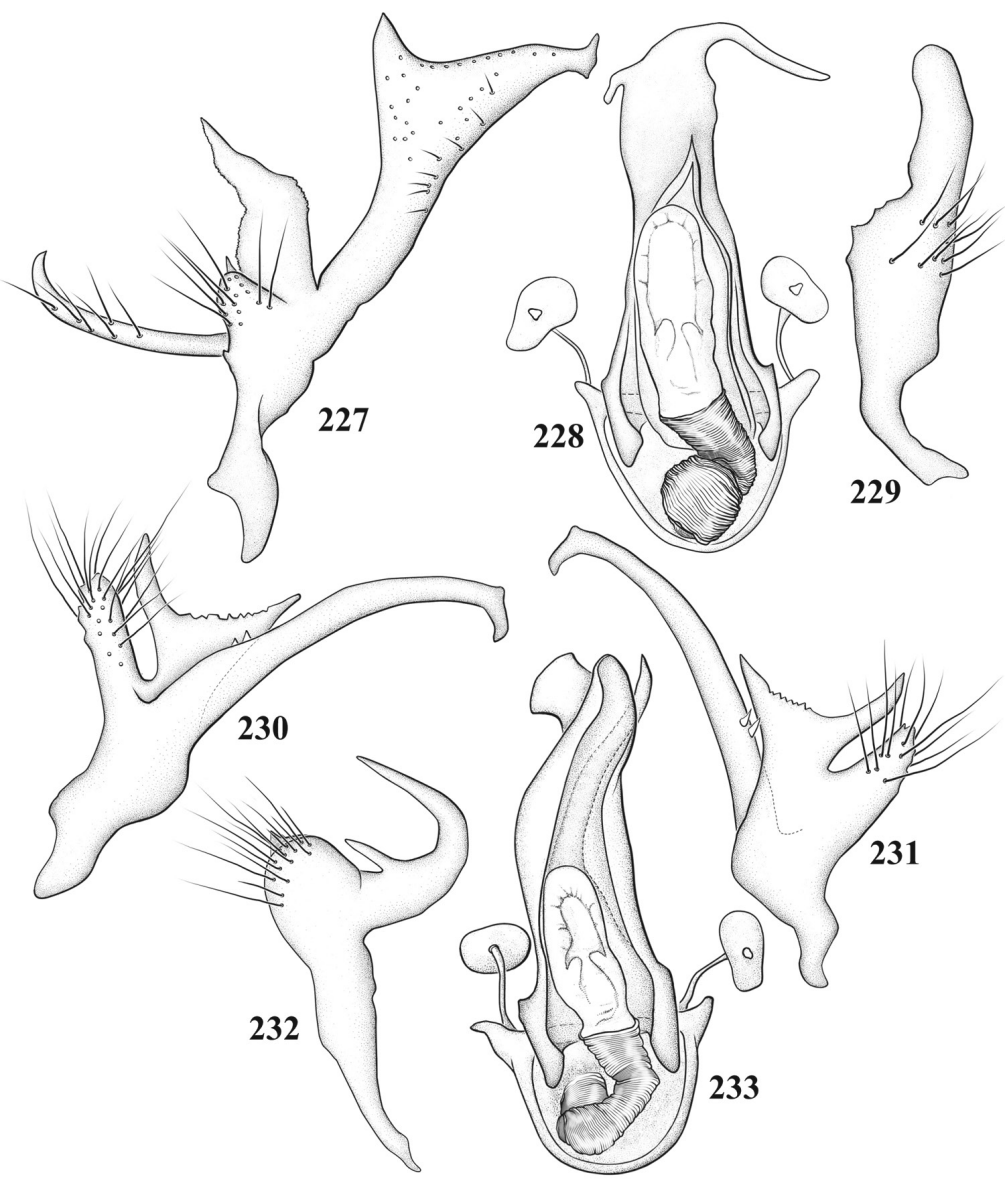
Male genitalia of *Pilophoropsidea* spp. **227–229:** *Pilophoropsidea fascipennis* **227** left paramere **228** phallotheca and endosoma **229** right paramere, caudal aspect **230–233:** *Pilophoropsidea fuscata* **230** left paramere, caudal aspect **231** left paramere, anterior aspect **232** endosoma and phallotheca **233** right paramere, caudal aspect.

**Figures 234–239.**
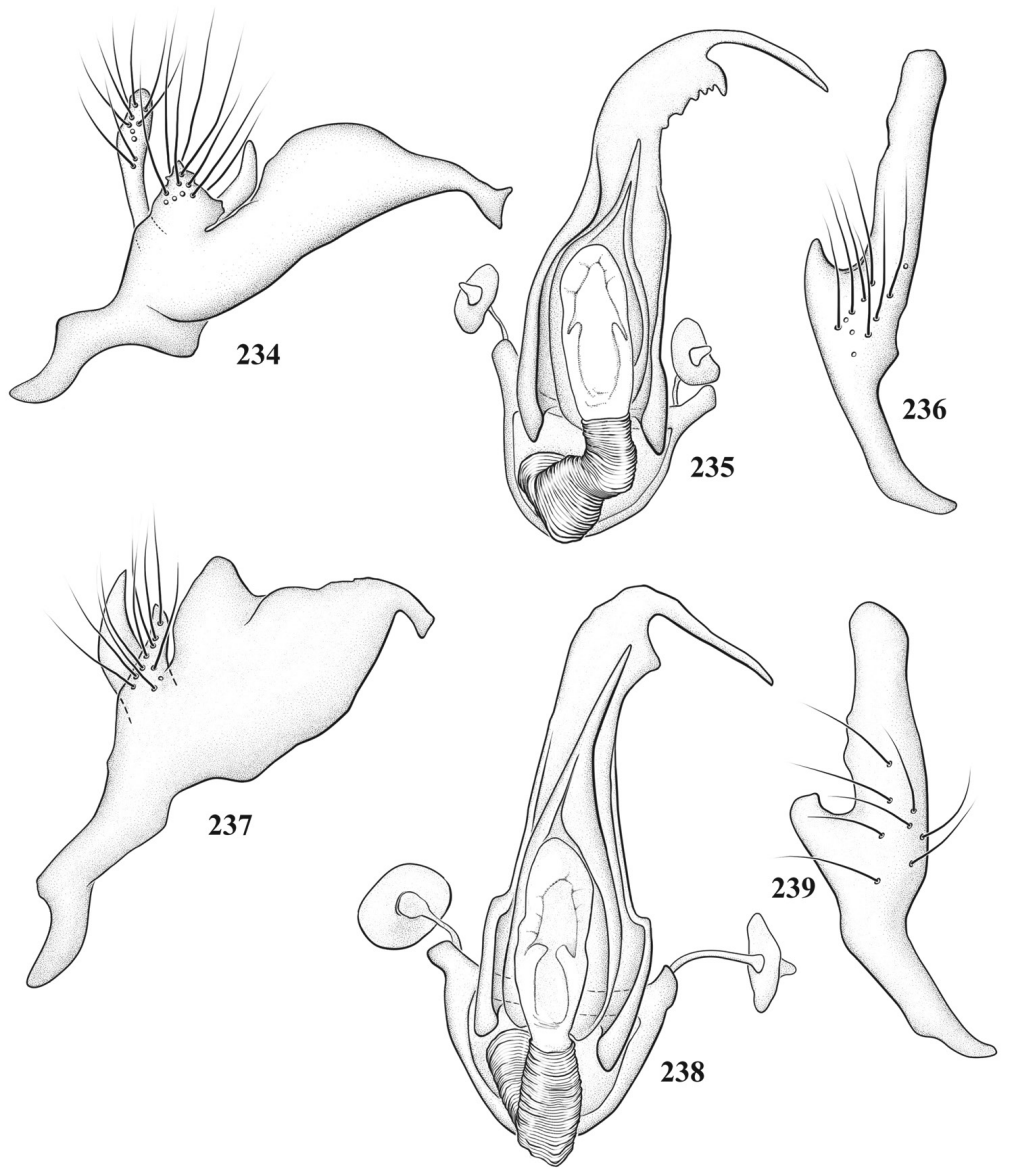
Male genitalia of *Pilophoropsidea* spp. **234–236:** *Pilophoropsidea keltoni* **234** left paramere **235** phallotheca and endosoma **236** right paramere, caudal aspect **237–239:** *Pilophoropsidea maxima* **237** left paramere **238** endosoma and phallotheca **239** right paramere, caudal aspect.

**Figures 240–245.**
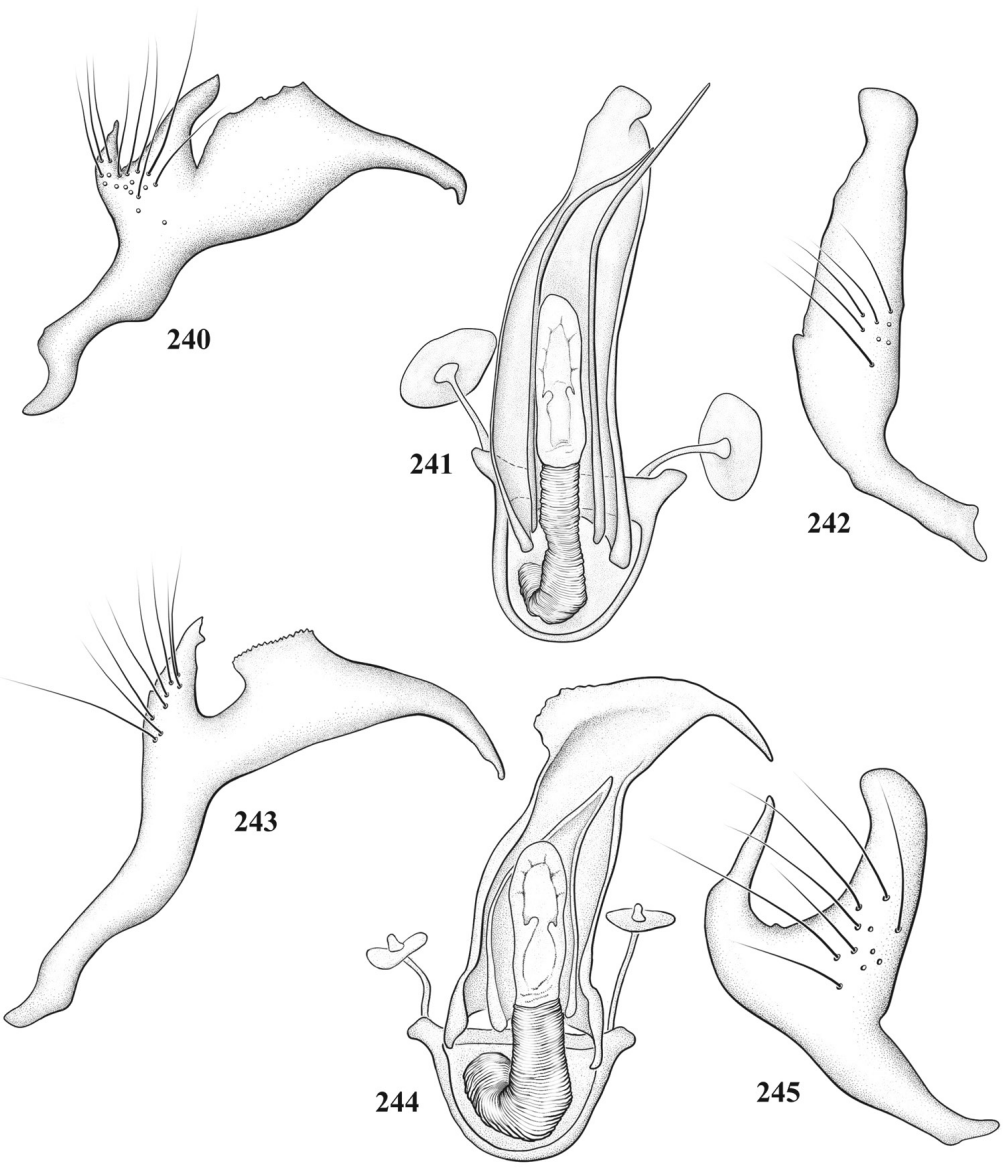
Male genitalia of *Pilophoropsidea* spp. **240–242:** *Pilophoropsidea pueblaensis* **240** left paramere **241** phallotheca and endosoma **242** right paramere, caudal aspect **243–245:** *Pilophoropsidea schaffneri* **243** left paramere **244** endosoma and phallotheca **245** right paramere, caudal aspect.

**Figures 246–253.**
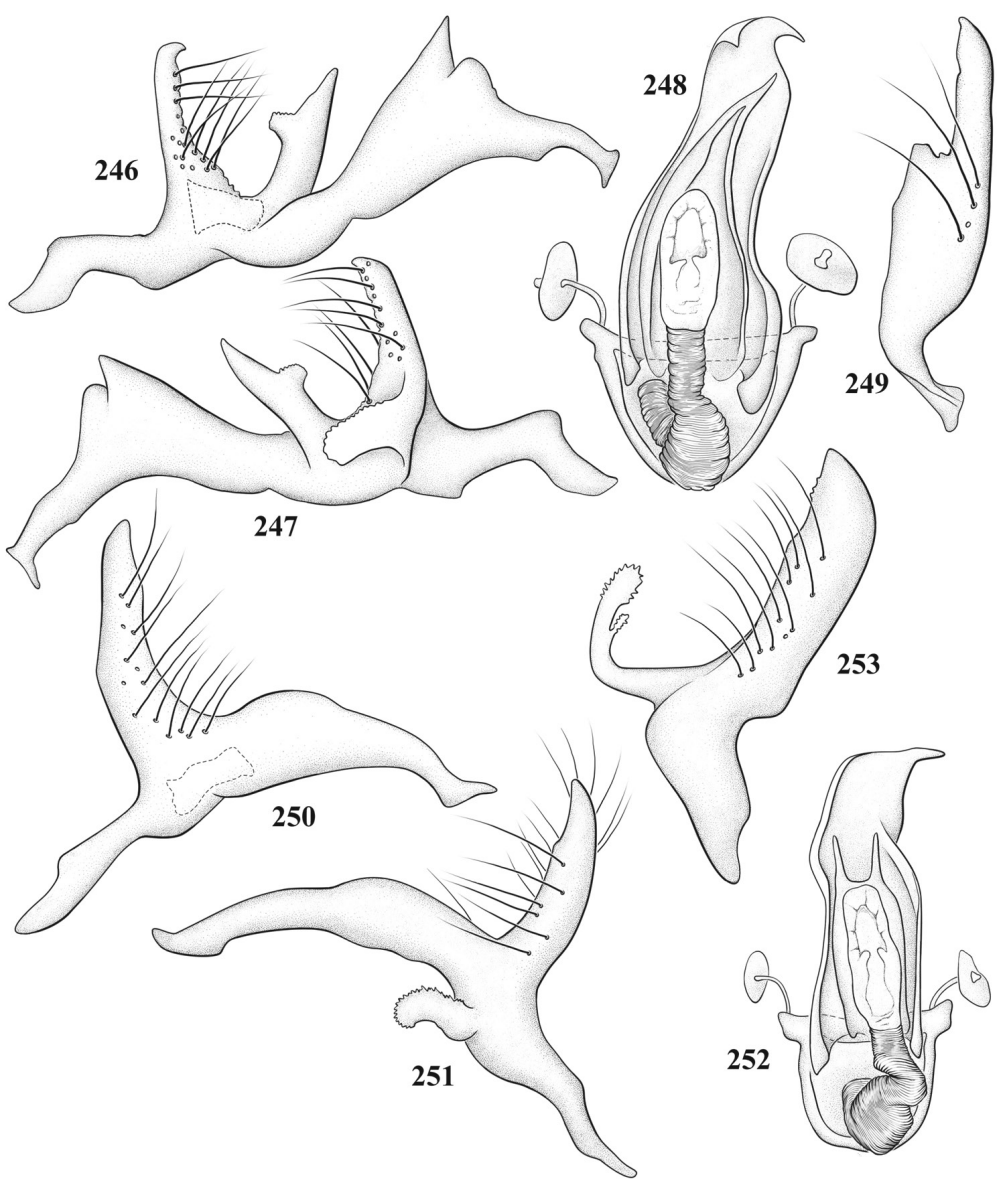
Male genitalia of *Pilophoropsidea* spp. **246–249:** *Pilophoropsidea serrata* **246** left paramere, caudal aspect **247** left paramere, anterior aspect **248** phallotheca and endosoma **249** right paramere, caudal aspect **250–253:** *Pilophoropsidea touchetae* **250** left paramere, caudal aspect **251** left paramere, anterior aspect **252** endosoma and phallotheca **253** right paramere, caudal aspect.

**Figures 254–259.**
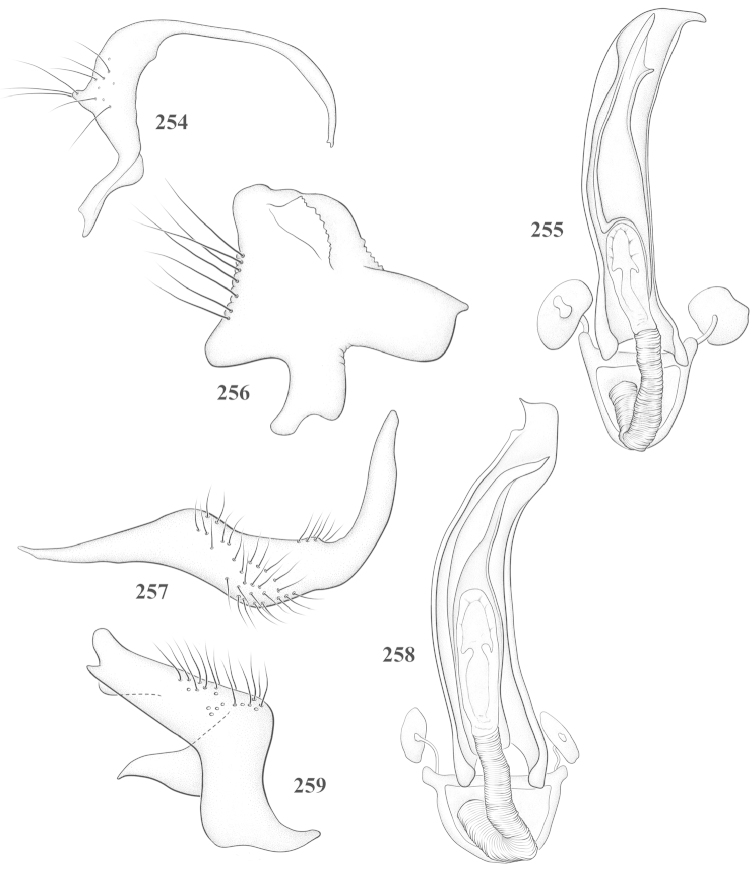
Male genitalia of *Pilophoropsidea* spp. **254–256:** *Pilophoropsidea truncata* **254** left paramere, caudal aspect **255** phallotheca and endosoma **256** right paramere, caudal aspect **257–259:** *Pilophoropsidea tuberculata* **257** left paramere, caudal aspect **258** endosoma and phallotheca **259** right paramere, caudal aspect.

**Figures 260–265.**
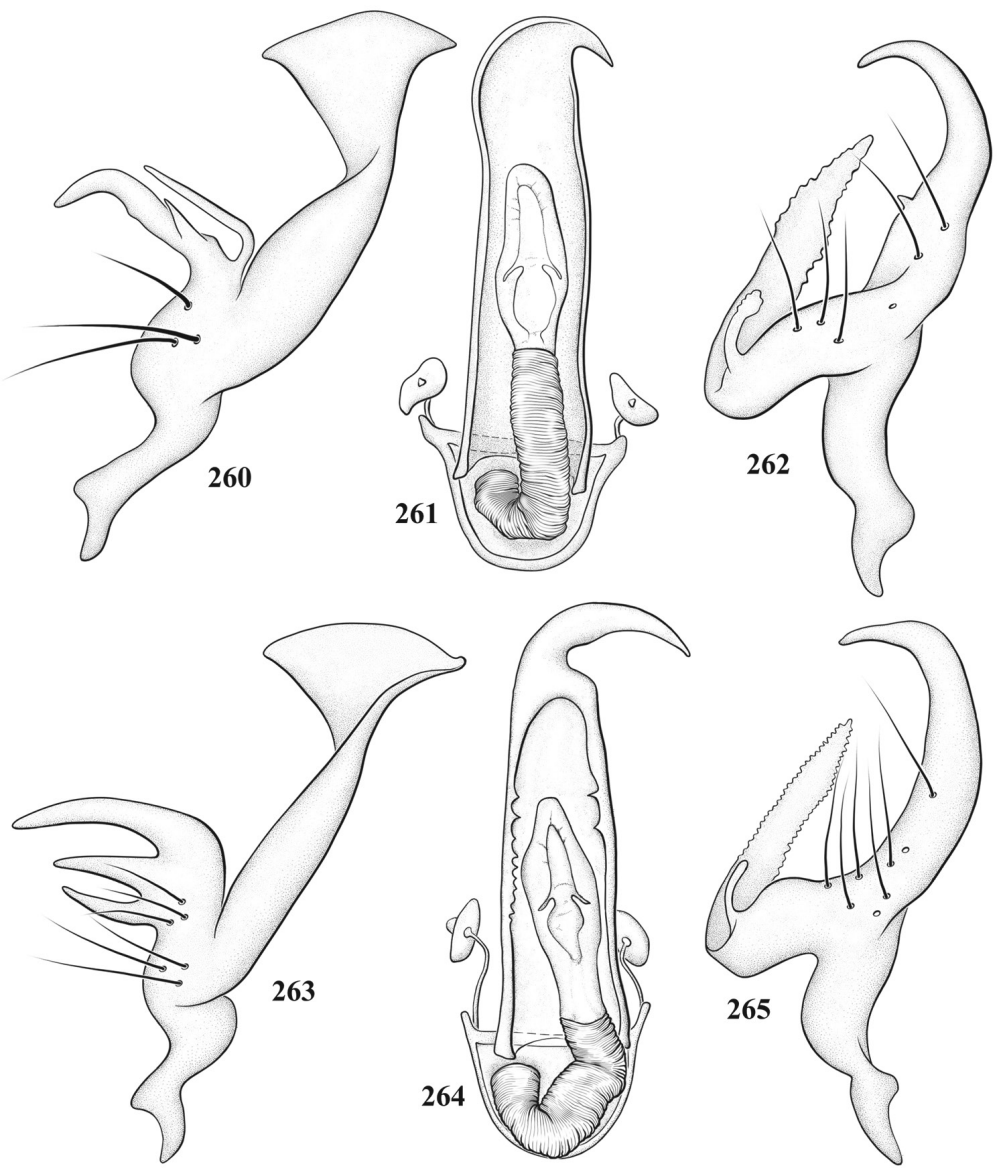
Male genitalia of *Pilophoropsis* spp. **260–262:** *Pilophoropsis bejeanae* **260** left paramere, caudal aspect **261** phallotheca and endosoma **262** right paramere, caudal aspect **263–265:** *Pilophoropsis brachyptera* **263** left paramere, caudal aspect **264** endosoma and phallotheca **265** right paramere, caudal aspect.

**Figures 266–271.**
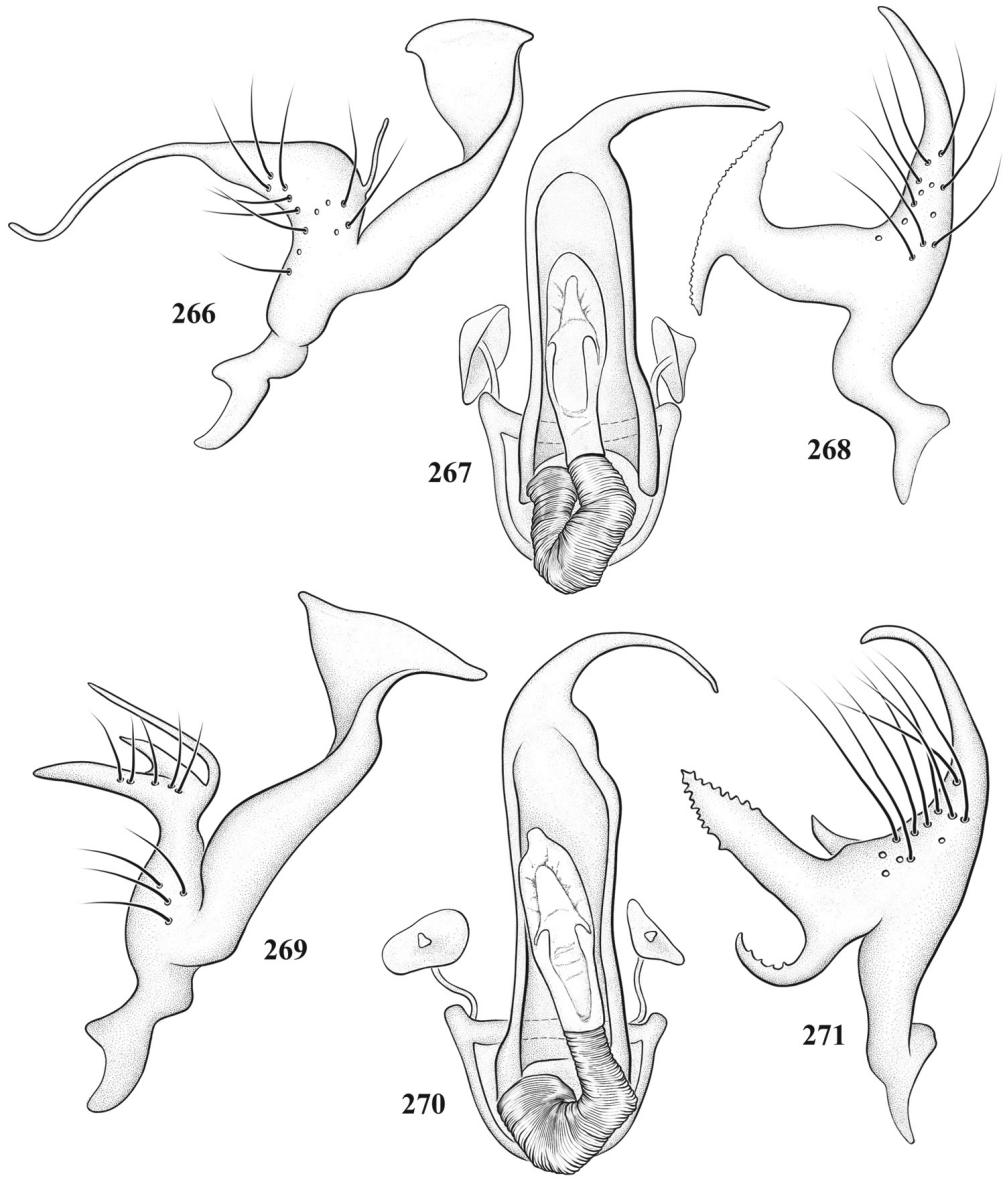
Male genitalia of *Pilophoropsidea* spp. **266–268:** *Pilophoropsis cunealis* **266** left paramere, caudal aspect **267** phallotheca and endosoma **268** right paramere, caudal aspect **269–271:** *Pilophoropsidea touchetae* **269** left paramere, caudal aspect **270** endosoma and phallotheca **271** right paramere, caudal aspect.

**Figures 272–279.**
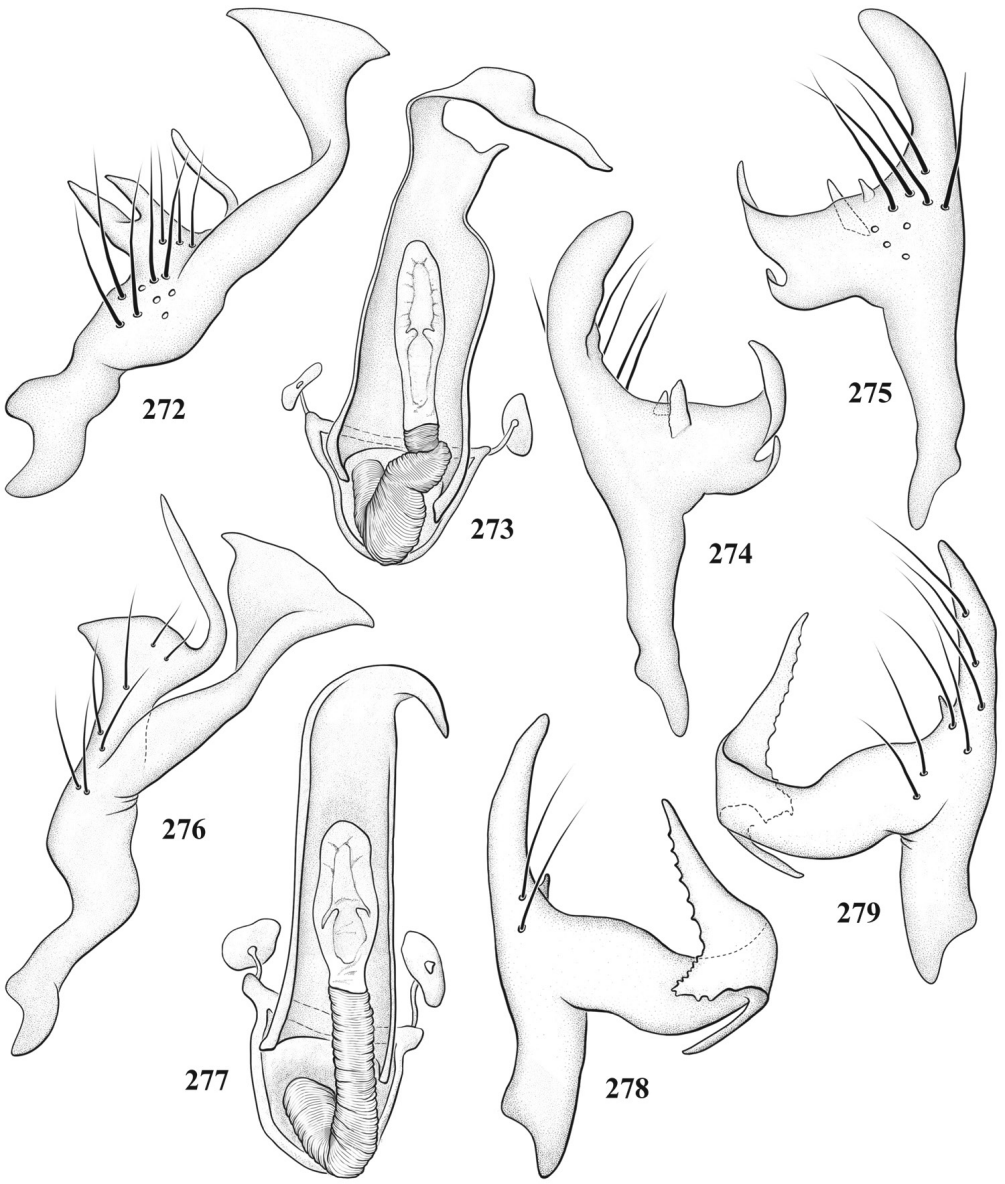
Male genitalia of *Pilophoropsidea* spp. **272–275:** *Pilophoropsis quercicola* **272** left paramere, caudal aspect **273** phallotheca and endosoma **274** right paramere, anterior aspect **275** right paramere, caudal aspect **276–279:** *Pilophoropsis texana* **276** left paramere, caudal aspect **277** endosoma and phallotheca **278** right paramere, anterior aspect **279** right paramere, caudal aspect.

**Figures 280–285.**
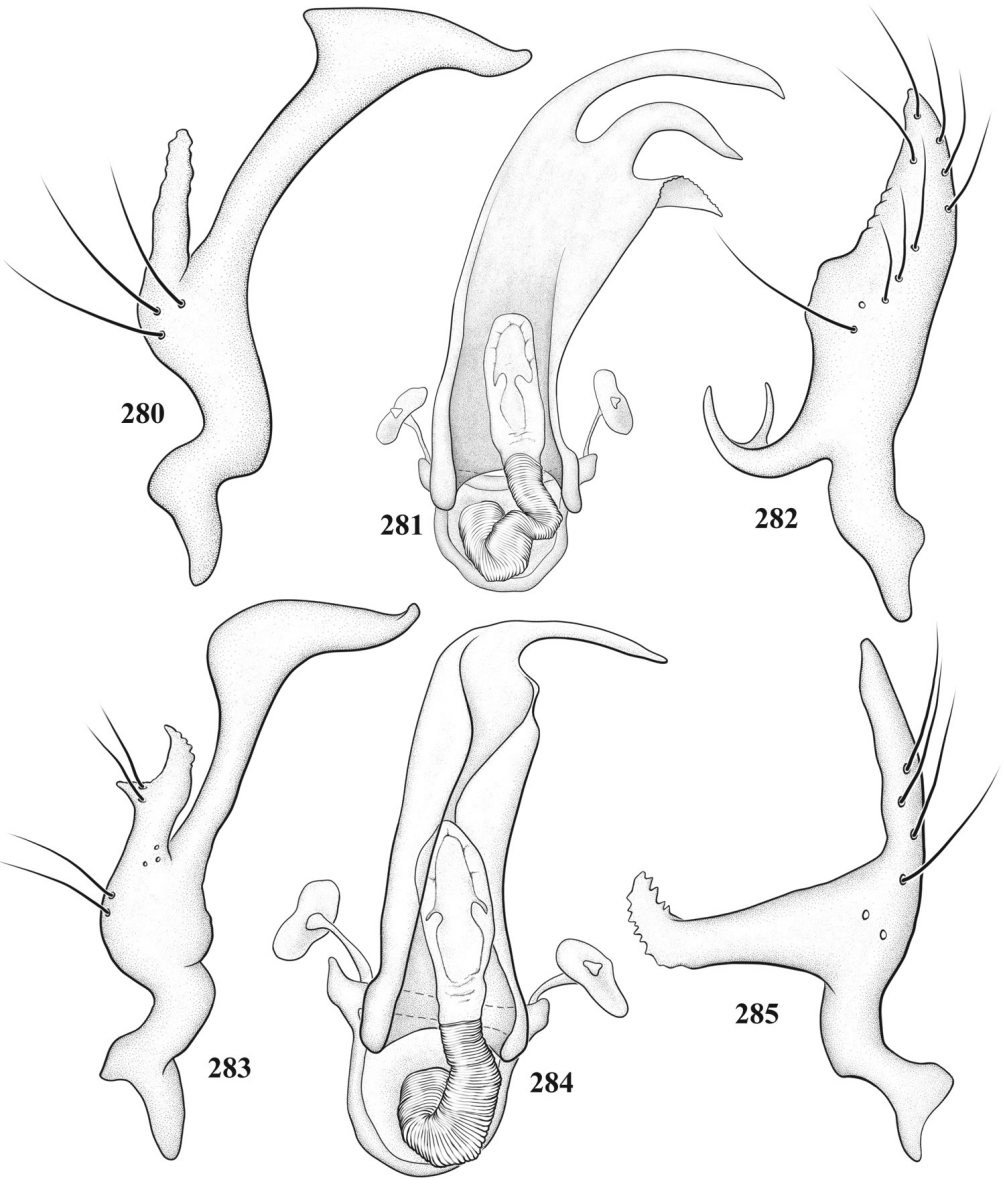
Male genitalia of *Marinonicoris* and *Pilophoropsita* spp. **280–282:** *Marinonicoris myrmecoides* **280** left paramere, caudal aspect **281** phallotheca and endosoma **282** right paramere, caudal aspect **283–285:** *Pilophoropsidea schaffneri* **283** left paramere, caudal aspect **284** endosoma and phallotheca **285** right paramere, caudal aspect.

**Figures 286–291.**
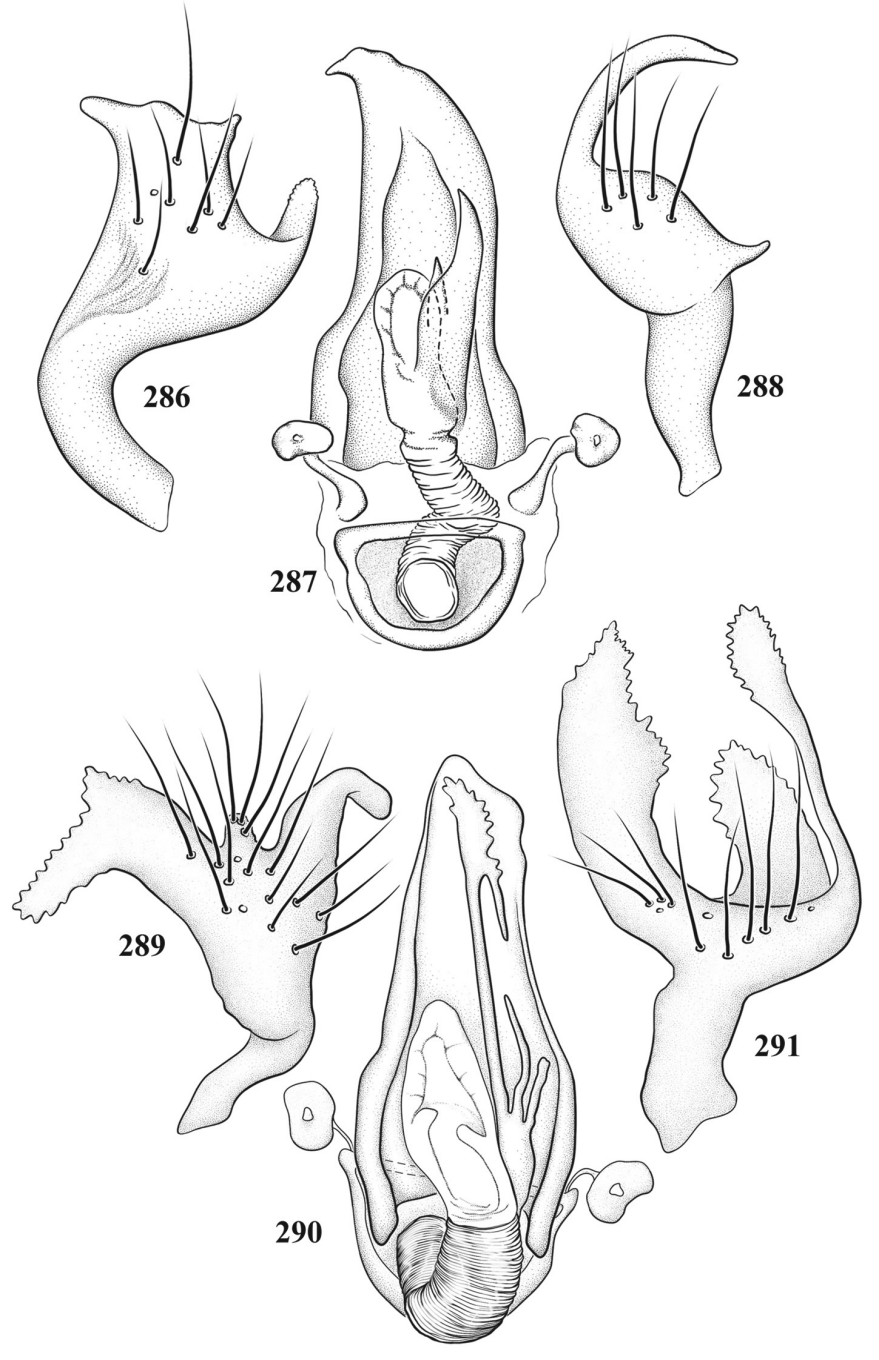
Male genitalia of *Renodaeus* spp. **286–288:** *Renodaeus gibbicollis* (redrawn after Carvalho and Becker 1959) **286** left paramere, caudal aspect **287** phallotheca and endosoma **288** right paramere, caudal aspect **289–291:** *Renodaeus mimeticus* **289** left paramere, caudal aspect **290** endosoma and phallotheca **291** right paramere, caudal aspect.

**Figures 292–297.**
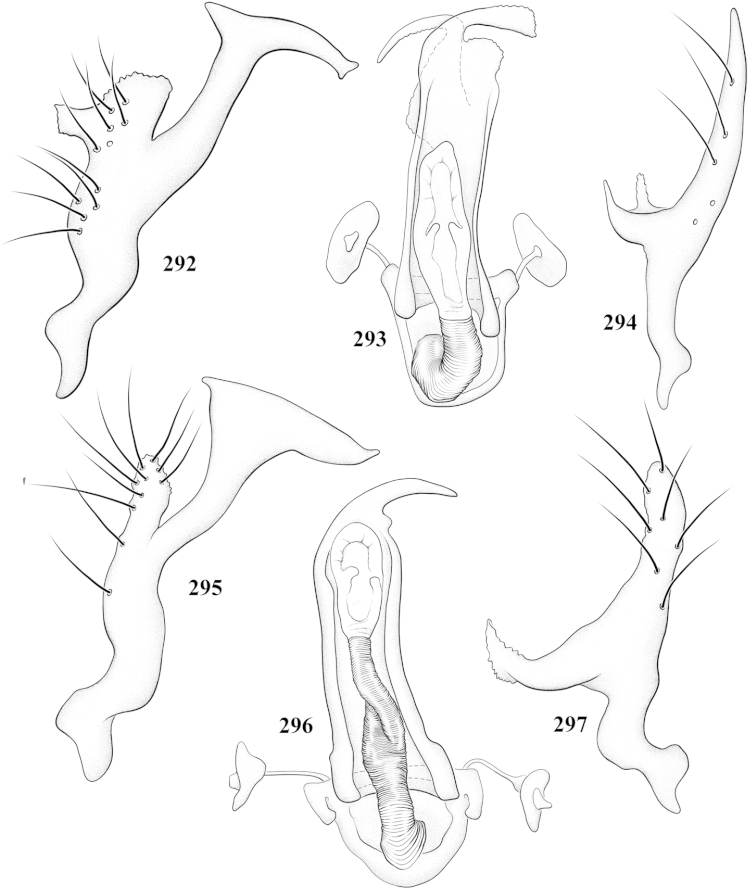
Male genitalia of *Zanchisme* spp. **292–294:** *Zanchisme dromedarius* **292** left paramere, caudal aspect **293** phallotheca and endosoma **294** right paramere, caudal aspect **295–297:** *Zanchisme illustris* **295** left paramere, caudal aspect **296** endosoma and phallotheca **297** right paramere, caudal aspect.

**Figures 298–303.**
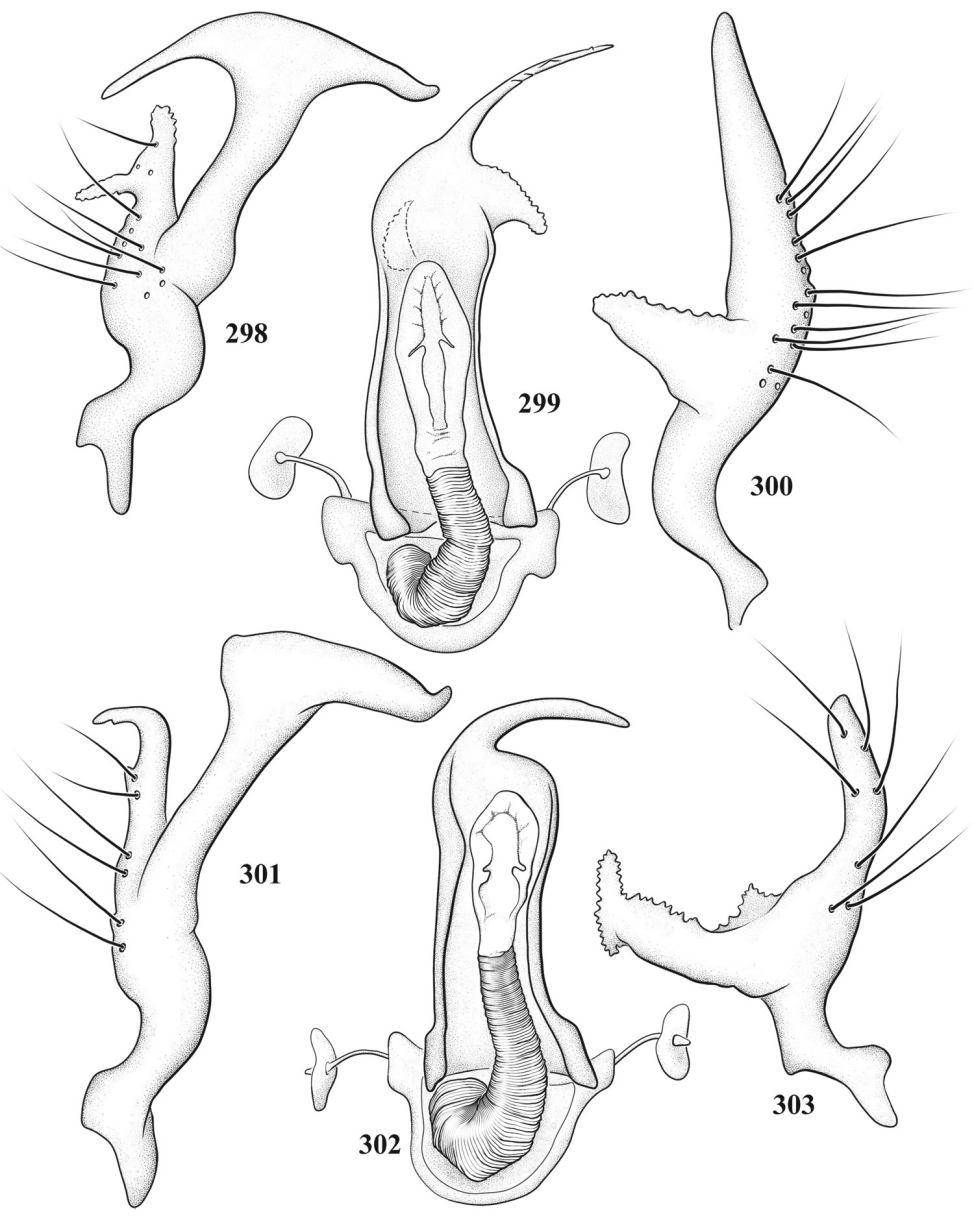
Male genitalia of *Zanchisme* spp. **298–300:** *Zanchisme inermis* **298** left paramere, caudal aspect **299** phallotheca and endosoma **300** right paramere, caudal aspect **301–303:** *Zanchisme mexicanus* **301** left paramere, caudal aspect **302** endosoma and phallotheca **303** right paramere, caudal aspect.

**Figures 304–306.**
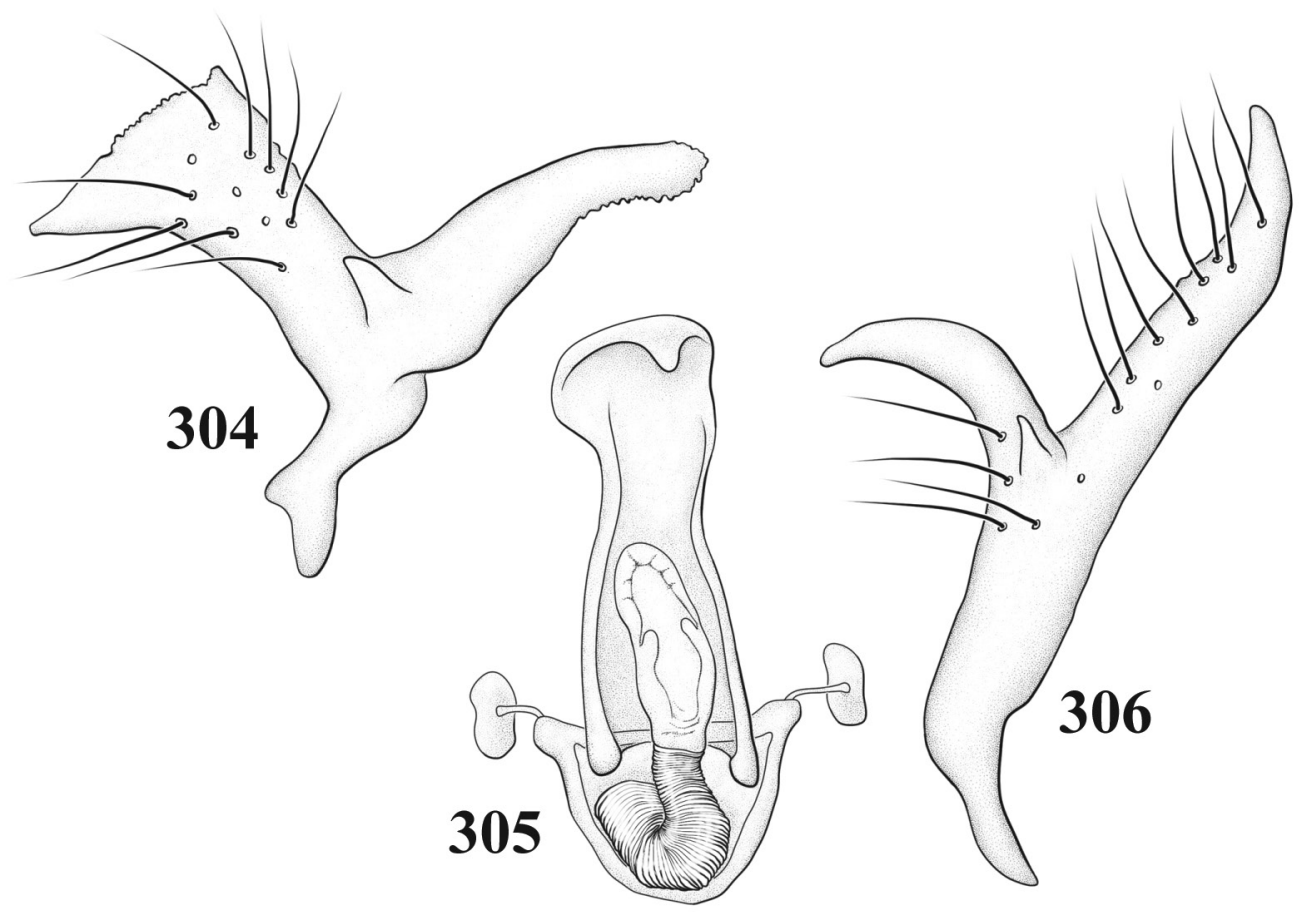
Male genitalia of *Zanchismeopsidea diegoi* **304** left paramere, caudal aspect **305** phallotheca and endosoma **306** right paramere, caudal aspect.

## Supplementary Material

XML Treatment for Ceratocapsidea

XML Treatment for Ceratocapsidea alayoi

XML Treatment for Ceratocapsidea bahamaensis

XML Treatment for Ceratocapsidea balli

XML Treatment for Ceratocapsidea baranowskii

XML Treatment for Ceratocapsidea complicata

XML Treatment for Ceratocapsidea consimilis

XML Treatment for Ceratocapsidea cubana

XML Treatment for Ceratocapsidea dominicanensis

XML Treatment for Ceratocapsidea fusiformis

XML Treatment for Ceratocapsidea holguinensis

XML Treatment for Ceratocapsidea nigropicea

XML Treatment for Ceratocapsidea rileyi

XML Treatment for Ceratocapsidea rufistigma

XML Treatment for Ceratocapsidea taeniola

XML Treatment for Ceratocapsidea texensis

XML Treatment for Ceratocapsidea transversa

XML Treatment for Ceratocapsidea variabilis

XML Treatment for Marinonicoris

XML Treatment for Marinonicoris myrmecoides

XML Treatment for Pilophoropsidea

XML Treatment for Pilophoropsidea barberi

XML Treatment for Pilophoropsidea brailovskyi

XML Treatment for Pilophoropsidea camela

XML Treatment for Pilophoropsidea cuneata

XML Treatment for Pilophoropsidea dimidiata

XML Treatment for Pilophoropsidea fascipennis

XML Treatment for Pilophoropsidea fuscata

XML Treatment for Pilophoropsidea keltoni

XML Treatment for Pilophoropsidea maxima

XML Treatment for Pilophoropsidea pueblaensis

XML Treatment for Pilophoropsidea schaffneri

XML Treatment for Pilophoropsidea serrata

XML Treatment for Pilophoropsidea touchetae

XML Treatment for Pilophoropsidea truncata

XML Treatment for Pilophoropsidea tuberculata

XML Treatment for Pilophoropsis

XML Treatment for Pilophoropsis bejeanae

XML Treatment for Pilophoropsis brachyptera

XML Treatment for Pilophoropsis cunealis

XML Treatment for Pilophoropsis nicholi

XML Treatment for Pilophoropsis quercicola

XML Treatment for Pilophoropsis texana

XML Treatment for Pilophoropsita

XML Treatment for Pilophoropsita schaffneri

XML Treatment for Renodaeus

XML Treatment for Renodaeus ficarius

XML Treatment for Renodaeus gibbicollis

XML Treatment for Renodaeus mimeticus

XML Treatment for Zanchisme

XML Treatment for Zanchisme dromedarius

XML Treatment for Zanchisme illustris

XML Treatment for Zanchisme inermis

XML Treatment for Zanchisme mexicanus

XML Treatment for Zanchismeopsidea

XML Treatment for Zanchismeopsidea diegoi

## Appendix I

Locality data for specimens in color habitus plates.

**Figures 24–35.** *Ceratocapsidea* spp. **24** *Ceratocapsidea alayoi*, ♂ (paratype: Cuba, Tortugilla, Guantánamo, Jul. 1975, L. B. Zayas) **25** *Ceratocapsidea bahamaensis*, ♂ (paratype: Bahama Isls., New Providence Isl., Nassau, 16 Apr. 1953, E. B. Hayden) **26** *Ceratocapsus balli*, ♂ (USA, Texas, Brazos Co., College Station, E. Riley estate, 18–19 May 2006, K. Menard) **27** *Ceratocapsus balli*, ♂ (lateral aspect) **28** *Ceratocapsus balli*, ♀ (USA, Florida, Volusia Co., Rt. 415, 2 mi. N of Osteen, 27 Apr. 1984, T. J. Henry, J. T. Polhemus, and A. G. Wheeler) **29** *Ceratocapsidea baranowskii*, ♂ (Jamaica, Par. of St. Andrew, 4000 ft., Holywell Forest Camp, 1 Jul. 1972, M. Winegar, blacklight) **30** *Ceratocapsidea complicata*, ♂ (USA, Texas, Llano Co., Ink Lake, Rt. 29 & Jct. to Dam Rd., 12 June 1997, T. J. Henry & J. C. Schaffner) **31** *Ceratocapsidea complicata*, ♀ (allotype: USA, Illinois, Herod, 24 Jul. 1930, Knight & Rose) **32** *Ceratocapsidea consimilis*, ♂ (Jamaica, St. James, Adelphi, 15 Aug. 1966, H. Howden) **33** *Ceratocapsidea cubana*, ♂ (lectotype: Cuba, Stål) **34** *Ceratocapsidea dominicanensis*, ♂ (holotype: Republica Dominicana, San Cristobal Prov., San Cristobal, 5 Aug. 1967, J. C. Schaffner) **35** C. dominicanensis, ♀ (paratype: Republica Dominicana, San Cristobal Prov., San Cristobal, 19 Sept. 1967, L. H. Rolston).

**Figures 36–47.** *Ceratocapsidea* spp. **36** *Ceratocapsus fusiformis*, ♂ (USA, Texas, Brazos Co., College Station, E. Riley Estate, 18–19 May 2006, K. Menard) **37** *Ceratocapsus fusiformis*, ♀ (USA: North Carolina, Mecklenburg Co., Rt. 51, 1 mi. W of Rt. 16, nr. Matthews, 2 Jul. 1976, A. G. Wheeler, Jr.) **38** *Ceratocapsidea holguinensis*, ♂ (Cuba, Soledad nr. Cienfuego, 6–20–VIII, N. Banks) **39** *Ceratocapsidea nigropicea*, ♂ (paralectotype: Jamaica, Mandeville, Apr. 1906, E. P. Van Duzee) **40** *Ceratocapsidea nigropicea*, ♀ (Jamaica, Ft. Clarence, 7 Dec. 1975, L. & C. W. O’Brien & Marshall) **41** *Ceratocapsidea rileyi*, ♂ (holotype: USA, Texas, Kenedy Co., Kenedy Ranch, Jaboncillos Pasture, sand dune area, 21 Apr. 2001, Raber, Riley, & Yoder, uv light) **42** *Ceratocapsidea rufistigma*, ♂ (USA, Florida, Marion Co., Rt. 40, 12 mi. E Lynne, 24 Apr. 1984, T. J. Henry & A. G. Wheeler, Jr, on *Pinus clausa*) **43** *Ceratocapsidea rufistigma*, ♀ (USA, Florida, Sanford, 24 Jul. 1927, E. D. Ball) **44** *Ceratocapsidea taeniola*, ♂ (holotype: Jamaica, Par. of St. Andrew, 4000, Holywell Forest Camp, 11 Aug. 1971, M. Winegar, blacklight) **45** *Ceratocapsidea texensis*, ♂ (holotype: USA, Texas, Cocan, 4 June 1933, P. W. Oman) **46** *Ceratocapsidea texensis*, ♀ (paratype: USA, Texas, Cocan, 4 June 1933, P. W. Oman) **47** *Ceratocapsidea transversa*, ♂ (Mexico, Nuevo León, Chipinque Mesa, 5400’, nr Monterrey, 8 Jul. 1963, H. & A. Howden).

**Figures 48–59.** *Ceratocapsidea* and *Pilophoropsidea* spp. Figure 48, *Ceratocapsidea variabilis*, ♂ (holotype: Jamaica, Par. of St. Andrew, 4000, ft., Holywell Forest Camp, 1 Sept. 1971, M. Winegar). *Pilophoropsidea* spp. **49–59:** 49, *Pilophoropsidea barberi*, ♂ (holotype: USA, Arizona, Huachuca Mtns., Jul. 1920) **50** *Pilophoropsidea brailovskyi*, ♂ (Mexico, Federal District, Temascaltepec, 9 Feb. 1979, H. Brailovsky) **51** *Pilophoropsidea camela*, ♂ (USA, Kentucky, Fulton Co., CR-430, nr Mississippi River, 2.1 mi. N of Sassafras Bridge, 19 June 2001, T. J. Henry & A. G. Wheeler, Jr., on *Toxicodendron radicans*) **52** *Pilophoropsidea camela*, ♂ lateral aspect **53** *Pilophoropsidea camela*, ♀ (USA, Oklahoma, Murray Co., Rt 77 at I-35, W of Dougherty, 316 m, 13 June 1999, T. J. Henry and A. G. Wheeler, Jr.) **54** *Pilophoropsidea camela* (lateral aspect) **55** *Pilophoropsidea cuneata*, ♂ (holotype: Mexico, Chiapas, 27 km. SW of Teopisca, 18 Sept. 1981, Clark & Coe) **56** *Pilophoropsidea dimidiata*, ♂ (Mexico, Durango, 9 mi. W La Ciudad Durango, 9000’, 1 Jul. 1964, L. A. Kelton) **57** *Pilophoropsidea fascipennis*, ♂ (USA, Arizona, Cochise Co., Chiricahua Mts., SWRS, 5 mi. SW Portal, el. 5300 ft, 22 Aug. 2000, J. C. Schaffner) **58** *Pilophoropsidea fuscata*, ♂ (paratype: USA, New Mexico, Sante Fe Co., 6 mi. E Tusque, 4 Jul. 1984, J. T. & D. A. Polhemus). 59, *Pilophoropsidea keltoni*, ♂ (Mexico, Durango, 3 mi. E El Salto, 8400’, 21 June 1964, L. A. Kelton).

**Figures 60–71.** *Pilophoropsidea* spp. **60** *Pilophoropsidea maxima*, ♂ (holotype: Mexico, Durange, 10 mi. W of El Salto, 9000’, 10 Aug. 1964, L. A. Kelton) **61** *Pilophoropsidea pueblaensis*, ♂ (holotype: Mexico, Puebla, Apulco, 13 mi. N Zacapoaxtla, 23 Jul. 1985, Jones & Schaffner, at light) **62** *Pilophoropsidea schaffneri*, ♂ (holotype: Mexico, San Luis Potosi, 28.5 mi. N of Huazache, 4 Jul. 1985, Jones & Schaffner) **63** *Pilophoropsidea schaffneri*, ♂ (holotype, lateral aspect). 64, *Pilophoropsidea schaffneri*, ♀ (paratype: Mexico, San Luis Potosi, 28.5 mi. N of Huazache, 4 Jul. 1985, Jones & Schaffner) **65** *Pilophoropsidea schaffneri*, ♀ (lateral aspect) **66** *Pilophoropsidea serrata*, ♂ (holotype: Mexico, Michoacan, 6 mi. N of Cheran, 7–8 Jul. 1985, Jones & Schaffner) **67** *Pilophoropsidea touchetae*, ♂ (holotype: Mexico, Puebla, 5.8 mi. NW of Telixtlahuaca, 21 Jul. 1987, Kovarik & Schaffner) **68** *Pilophoropsidea truncata*, ♂ (holotype: Mexico, Guerrero, 6.2 mi. SW of Xochipala, el. 5670 ft., 6 Jul. 1987, Kovarik & Schaffner) **69** *Pilophoropsidea truncata*, ♂ (lateral aspect) **70** *Pilophoropsidea tuberculata*, ♂ (holotype: Mexico, Guerrero, 6.2 mi. SW of Xochipala, el. 5670 ft., 6 Jul. 1987, Kovarik & Schaffner). 71, *Pilophoropsidea tuberculata*, ♀ (paratype: Mexico, Guerrero, 6.2 mi. SW of Xochipala, el. 5670 ft., 6 Jul. 1987, Kovarik & Schaffner).

**Figures 72–83.** *Pilophoropsidea* and *Pilophoropsis* spp. **72** *Pilophoropsidea tuberculata*, ♀ (lateral aspect). *Pilophoropsis* spp. **73–83:** **73** *Pilophoropsis bejeanae*, ♂ (holotype: Intercepted at Nogales, Arizona, on tomatoes from Mexico, 27 Dec. 1941) **74** *Pilophoropsis brachyptera*, ♂ (USA, Arizona, Pima Co., Lower Santa Rita Range, ne Sahuarita, 11 Apr. 1989, T. J. Henry & A. G. Wheeler, on Prosopis sp.) **75** *Pilophoropsis brachyptera*, ♀ (USA, Arizona, 4 mi. S of I-100, along R 82, 2 June 1997, T. J. Henry & A. G. Wheeler, on *Prosopis* sp.) **76** *Pilophoropsis cunealis*, ♂ (holotype: Mexico, Oaxaca, Puerto Escondido, 15 Jul. 1985, Jones & Schaffner, on *Acacia cochliacantha*) **77** *Pilophoropsis cunealis*, ♀ (paratype: Mexico, Oaxaca, Puerto Escondido, 15 Jul. 1985, Jones & Schaffner, on *Acacia cochliacantha*) **78** *Pilophoropsis cunealis*, ♀ (lateral aspect) **79** *Pilophoropsis nicholi*, ♂ (Mexico, Jalisco, Puerto Vallarta, 8 Dec. 1984, G. E. Bohart) **80** *Pilophoropsis nicholi*, ♀ (USA, Arizona, Santa Rita Mts., 16 May 1928, alt. 4000’, A. A. Nichol) **81** *nicholi*, ♀ (lateral aspect) **82** *Pilophoropsis quercicola*, ♂ (holotype: USA, Arizona, Cochise Co., Ash Canyon Rd., nr Rt. 92, S of Sierra Vista, 4 June 1997, T. J. Henry & A. G. Wheeler, on *Quercus longifolia*) **83** *Pilophoropsis quercicola*, ♀ (paratype: USA, Arizona, Cochise Co., Ash Canyon Rd., nr Rt. 92, S of Sierra Vista, 4 June 1997, T. J. Henry & A. G. Wheeler, on *Quercus longifolia*).

**Figures 84–94.** *Pilophoropsis*, *Pilophoropsita*, and *Renodaeus* spp. **84** *Pilophoropsis texana*, ♂ **85** *Pilophoropsis texana*, ♀ (holotype: USA, Texas, Brownsville, Esperanza Ranch, no date) **86–88:** **86** *Pilophoropsita schaffneri*, ♂ (paratype: Mexico, Oaxaca, 3.3 mi. E Rio Hondo, 5 Aug. 1980, Schaffner, Weaver, & Friedlander) **87** *Pilophoropsidea schaffneri*, ♂ (lateral aspect) **88** *Pilophoropsidea schaffneri*, ♀ (paratype: Mexico, Oaxaca, Puerto Escondido, 15 Jul. 1985, Jones & Schaffner) **89–92:** *Renodaeus* spp. **89** *Renodaeus ficarius* ♀ (lectotype: Guatemala, Cerro Zunil) **90** *Renodaeus mimeticus*, ♂ (Ecuador, Orellana Prov., Tiputini Biological Station, 216 m, 27 Oct. 1998, T. L. Erwin, et al., taken in insecticidal fogging) **91** *Renodaeus mimeticus*, ♂ (lateral aspect) **92** *Renodaeus mimeticus*, ♀ (Ecuador, Orellana Prov., Tiputini Biological Station, 216 m, 27 Oct. 1998, T. L. Erwin, et al., taken in insecticidal fogging) **93–94:** **93** *Marinonicoris myrmecoides*, ♂ (Argentina, Missiones, Dept. Concep., Sta. Marla, M. J. Viana) **94** *Marinonicoris myrmecoides*, ♀ ((Argentina, Missiones, Dept. Concep., Sta. Marla, M. J. Viana).

**Figures 95–106.** *Zanchisme* and *Zanchismeopsidea* spp. **95–104:** *Zanchisme* spp. **95** *Zanchisme dromidarus*, ♂; (Colombia, Bolivar, Cartogena, 8 Oct. 1971, G. E. Bohart) **96** *Zanchisme dromedarius*, ♂ (lateral aspect) **97** *Zanchisme dromedarius*, ♀ (Colombia, Mgd., Rio Frio, Mar. 1937, Darlington) **98** *Zanchisme inermis*, ♂ (Costa Rica, Guanacaste, S. of Caños, 9–14 Mar. 1990, F. D. Parker) **99** *Zanchisme inermis*, ♀ (Mexico, Guerrero, Barra Vieja, 5 Mar. 1986, H. Miranda) **100** *Zanchisme illustris*, ♂ (Jamaica, Ft. Clarence, 7 Dec. 1975, O’Brien & Marshall) **101** *Zanchisme illustris*, ♀ (Jamaica, Montego Bay, 15 Mar. 1911, no collector info.) **102** *Zanchisme mexicanus*, ♂ (Mexico, Chiapas, E of Cintalapa, 11 July 1991, R. W. Jones) **103** *Zanchisme mexicanus*, ♂ (lateral aspect) **104** *Zanchisme mexicanus*, ♀ (Panama, Canal Zone, Farfan Beach, 15 July 1976, W. E. Clark) **105** *Zanchisme mexicanus*, ♀ (lateral aspect) **105–106:** **104** *Zanchismeopsidea diegoi*, ♂ (holotype: Argentina, Santiago Del Estero, Añatuya, Mar. 1999, D. J. Carpintero) **105** *Zanchismeopsidea diegoi*, ♂ (lateral aspect).
